# Supplementary material for: Proteome efficiency of metabolic pathways in Escherichia coli increases along the nutrient flow
Source: mSystems. 2023 Oct 5;8(5):e00760-23. doi: 10.1128/msystems.00760-23 (PMC10654084; doi:10.1128/msystems.00760-23)
Supplement: Supplemental material — Fig. S1-S11 and Table S1-S6. [file msystems.00760-23-s0001.pdf]

Supplementary Figures and Tables for:

Proteome efficiency of metabolic pathways in *Escherichia coli* increases along the nutrient flow

Xiao-Pan Hu, Stefan Schroeder, Martin J. Lercher

This PDF includes Figure S1-S11 and Table S1-S6.

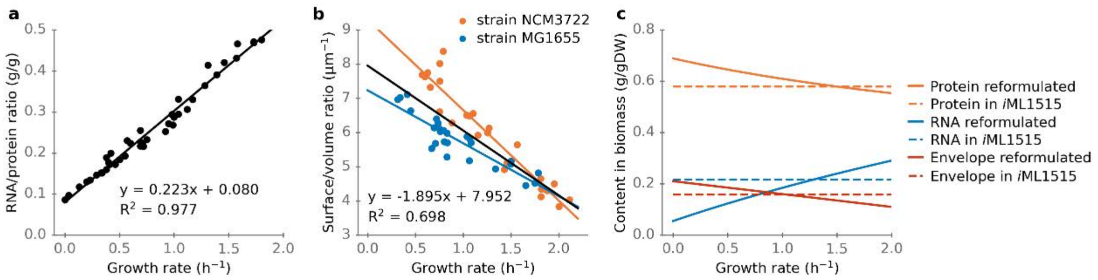

Fig. S1. Growth rate-dependent biomass composition. (**a**) Growth rate-dependent RNA/protein mass ratio; data from Ref.(1, 2). (**b**) Growth rate-dependent surface/volume ratio; data for wildtype strains in non-stressed growth conditions from Ref.(3). Surface/volume ratios are slightly different between the NCM3722 and MG1655 strains. Since the proteome data used in this study pertains to a third *E. coli* strain, BW25113, we used a regression model based on data from both strains (black line) to estimate the required production of cell envelope components. It should be noted that the re-estimated biomass composition here is not specific to any particular *E. coli* strain. **(c)** Growth rate-dependent biomass composition, reformulated considering the growth rate-dependent RNA/protein ratio in panel (a) and the surface/volume ratio in panel (b). The category "Envelope" includes murein, lipopolysaccharides, and lipids.

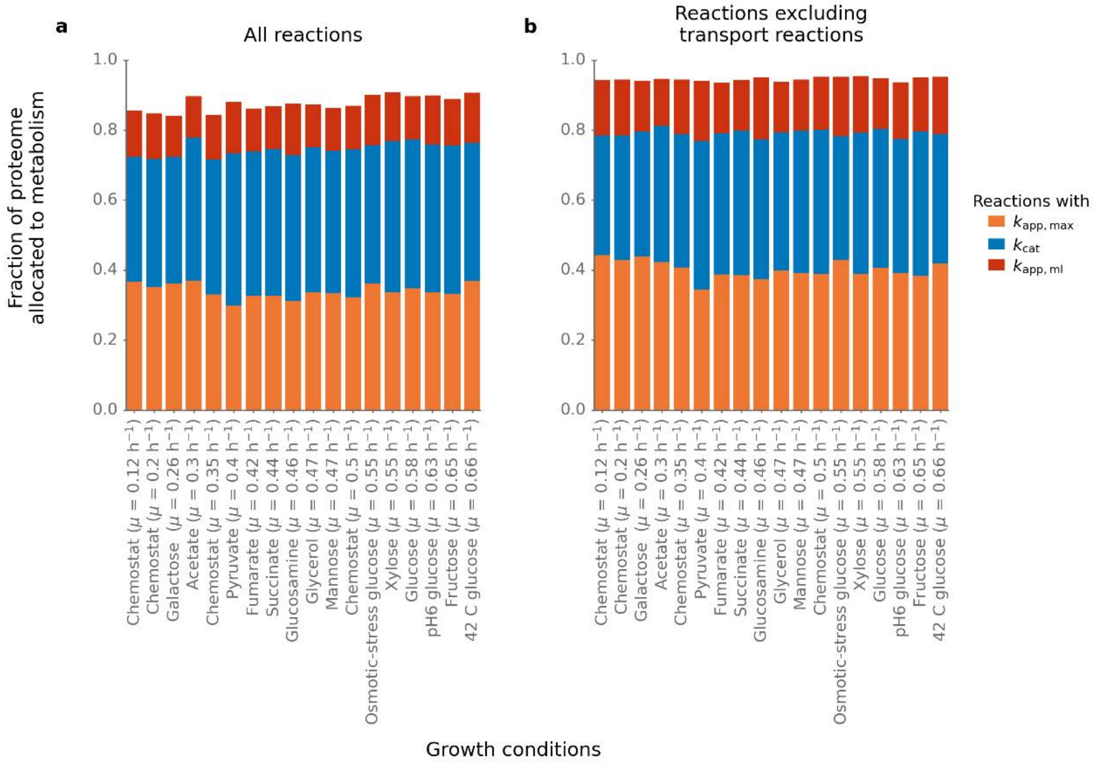

Fig. S2. Reactions with available enzyme turnover numbers cover more than 70% of the proteome fraction allocated to metabolism. Colors show the proteome fractions of enzymes with *in vivo* enzyme turnover number ($k_{app,max}$, in orange) or *in vitro* turnover number (k_{kcat}, in blue). (**a**) All reactions in iML1515. (**b**) All non-transport reactions in iML1515.

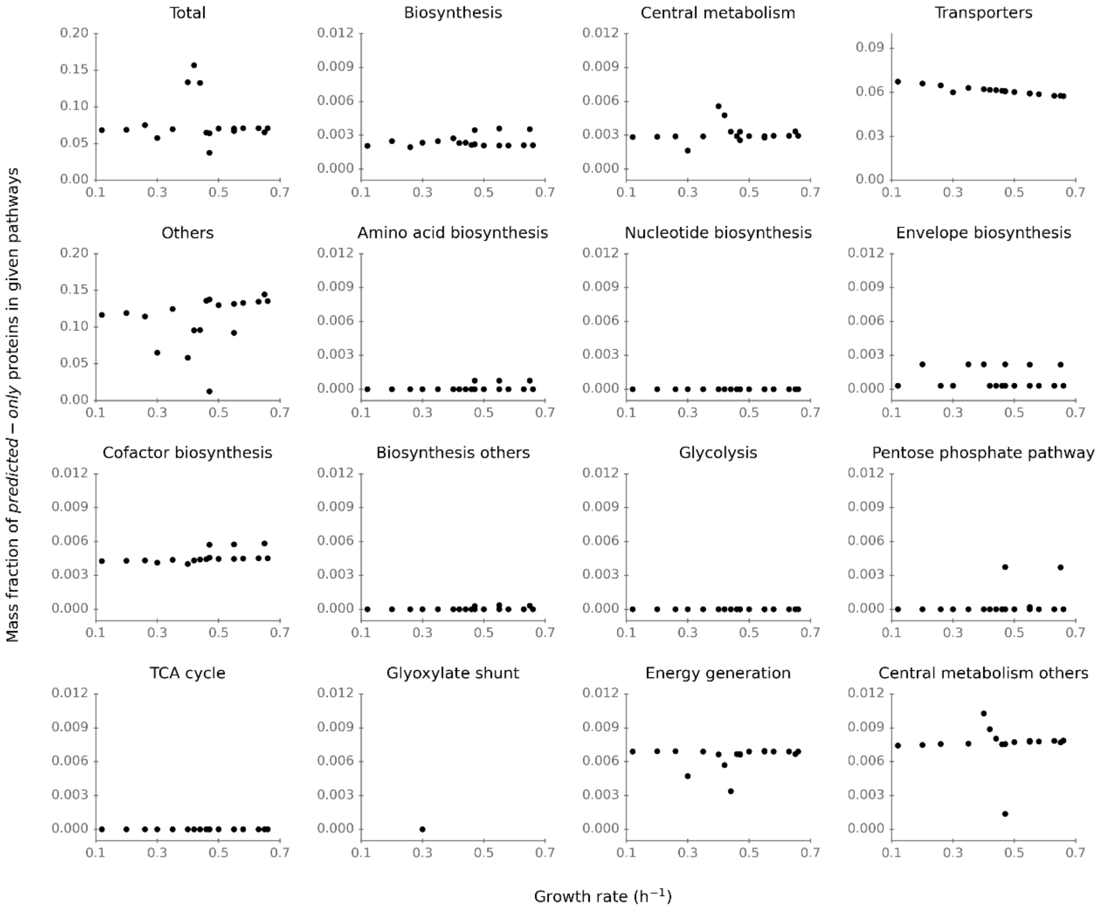

Fig. S3. The mass fraction of predicted-only proteins across pathways is generally low. Except for transporters and for proteins that cannot be assigned to particular pathways (others), the *predicted-only* proteins account for less than 1% of the predicted proteins in each pathway. We also note that the predictions of “others” and “transporters” are much lower than the measured data, and thus a relatively large fractions of predicted-only proteins in these two pathways does not change our conclusions.

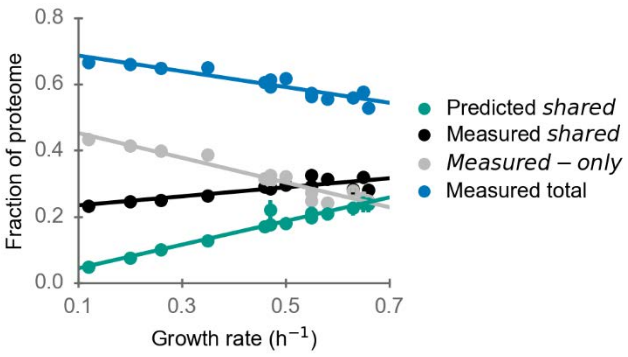

Fig. S4. Proteome efficiency of the whole metabolic network. With increasing growth rate, predicted and experimentally observed *shared* metabolic proteome fractions increase, while the *measured-only* metabolic proteome fraction decreases. Error bars extend from the 5th percentile to 95th percentile of 100 simulations, each with randomly perturbed turnover numbers.

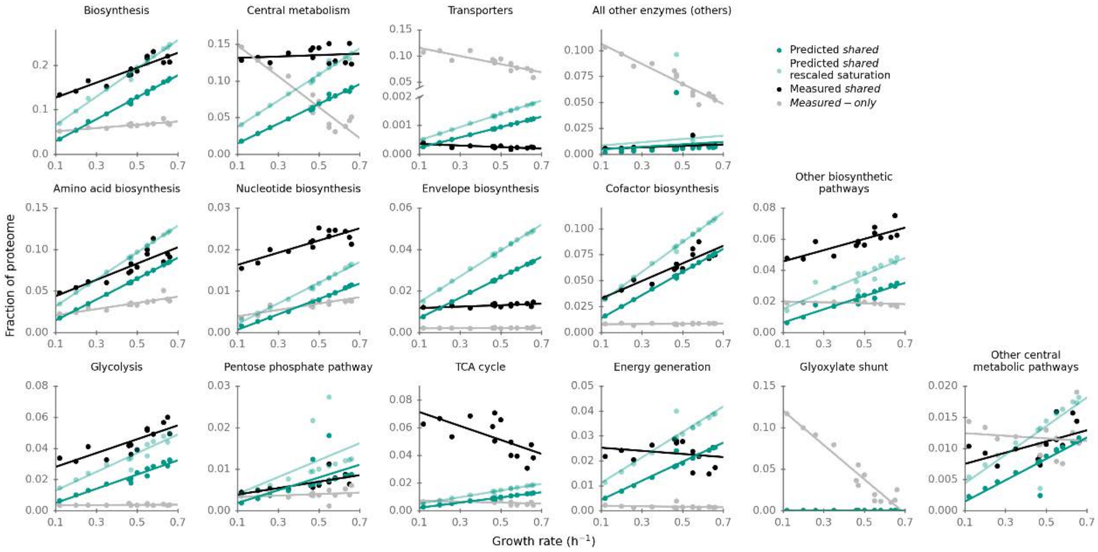

Fig. S5. Predictions of proteome allocated to pathways, including calculations based on growth rate-dependent expected enzyme saturation levels.

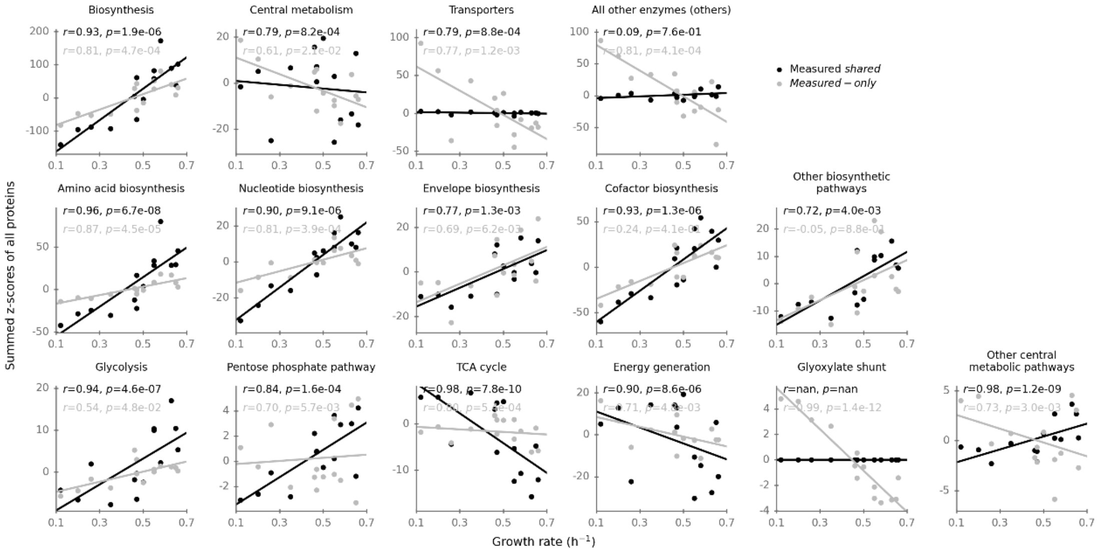

Fig. S6. Normalized proteome allocation trends of pathways based on z-scores. Pearson's *r* represents the correlation between the normalized proteome allocation and the total proteome fraction. The normalized proteome allocation of *shared* proteins is correlated with the total proteome fraction for all pathways; the only exception is "others", where most of the proteins are *measured-only* proteins.

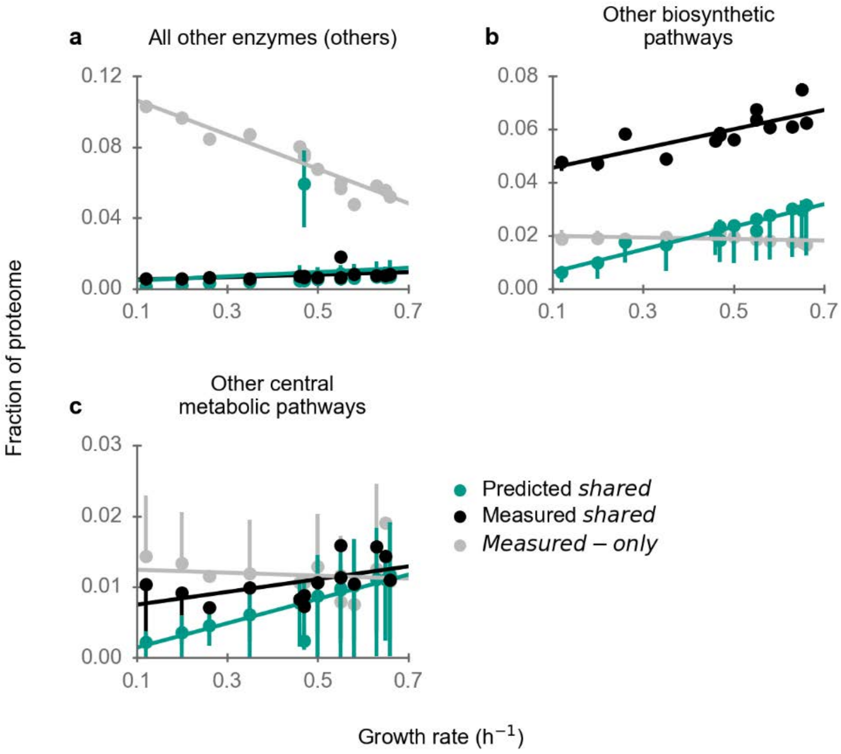

Fig. S7. The proteome efficiency of other pathways. (**a**) Combined proteome fraction of all enzymes in the *i*ML1515 model that are not part of the three pathway sets in Fig. 1b-1d ("others"). While the abundance of those proteins predicted to be active under optimality is similar to the expected abundance ($GMFE_{pathway}$ = 1.79), a much higher proteome fraction is allocated to *measured-only* proteins. (**b**) Proteome investment into those biosynthetic proteins that are not part of the four pathway sets in Fig. 2. While predicted and observed concentrations of *shared* enzymes are strongly correlated ($r_{pathway}^2$ = 0.60; Table 1) and the abundance of individual enzymes can be well explained by the predictions ($r_{individual}^2$ = 0.46), the corresponding values differ on average by almost 3-fold ($GMFE_{pathway}$ = 2.91). (**c**) Proteome investment into enzymes of central metabolism that are not part of the five pathway sets in Fig. 3. The prediction increases with growth rate, whereas the measured data is largely independent of growth rate. Error bars extend from the 5th percentile to 95th percentile of 100 simulations, each with randomly perturbed turnover numbers.

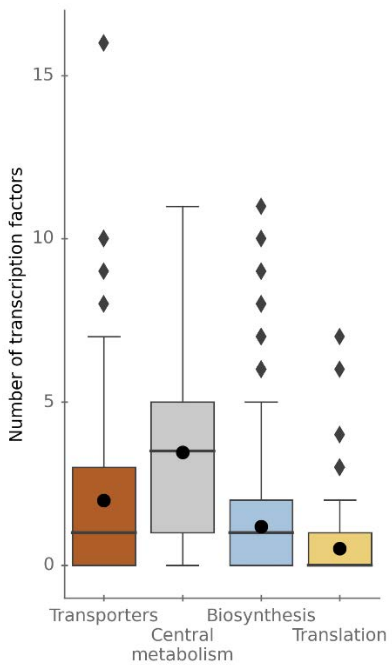

Fig. S8. Genes in more central cellular processes are regulated by fewer transcription factors than genes in peripheral processes. Genes encoding enzymes in biosynthesis pathways are regulated by fewer transcription factors than genes encoding transporters (one-sided Wilcoxon rank sum tests: $p < 10^{-10}$) and central metabolism ($p < 10^{-10}$). Genes in translation (here, all genes annotated with COG category "J"(4)) are also regulated by fewer transcription factors than genes in transporters ($p < 10^{-10}$) and central metabolism ($p < 10^{-10}$). Lines within boxes mark medians, dots indicate means. Boxes indicate 25% and 75% quantiles, whiskers extend from the boxes by 1.5x the interquartile range, diamonds are outliers.

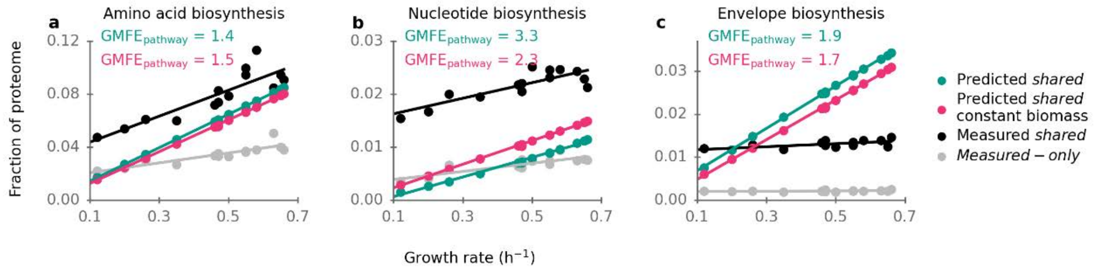

Fig. S9. Comparison of predictions assuming growth rate-dependent biomass composition versus constant biomass composition. (a) Amino acid biosynthesis pathway, (b) nucleotide biosynthesis pathway, and (c) cell envelope biosynthesis pathway. The composition of constant biomass was obtained from *i*ML1515 model.

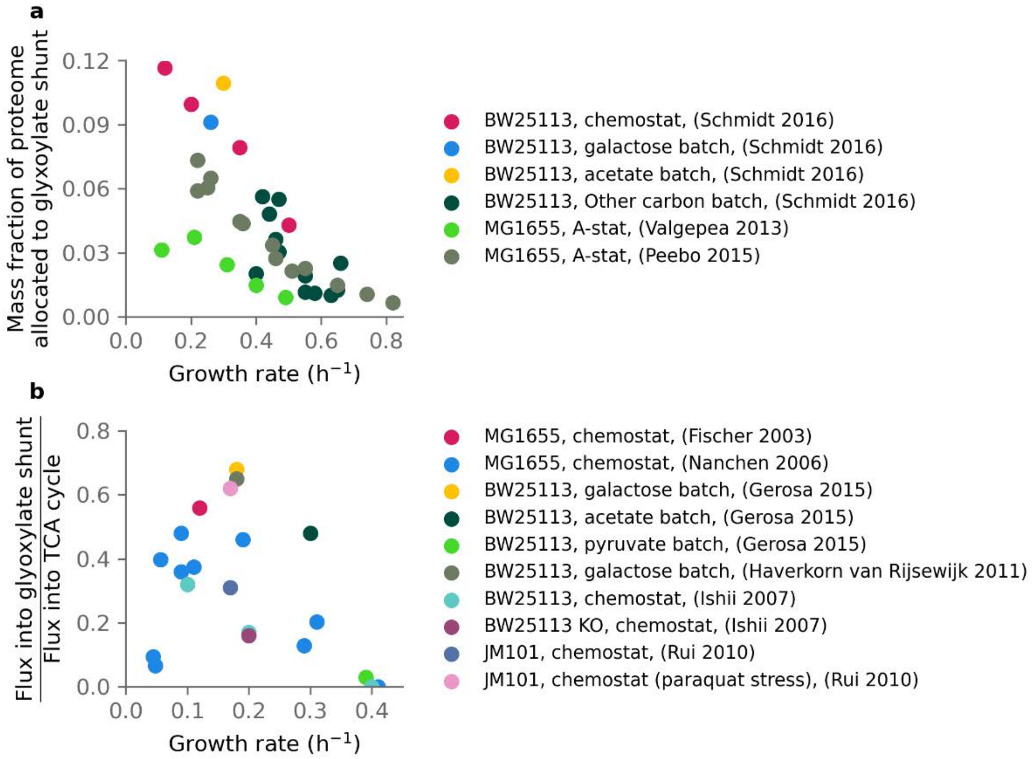

Fig. S10. Growth rate-dependent activity of the glyoxylate shunt. (**a**) Growth rate-dependent proteome allocation to the glyoxylate shunt, data from Refs. (5–7). (**b**) Growth rate-dependence of the flux through the glyoxylate shunt relative as a fraction of the flux into the TCA cycle. As the ^{13}C metabolic flux analysis usually lumps linear sequential reaction sequences into a single reaction, flux into the TCA cycle is quantified through ^{13}C measures of the flux of acetyl-CoA and oxaloacetate to isocitrate. The flux into the glyoxylate shunt is quantified through ^{13}C measures of the flux catalyzed by Isocitrate lyase (isocitrate -> succinate + glyoxylate). Data from Refs. (8–13). Both protein abundance and flux decrease with increasing growth rate. The glyoxylate shunt's high protein abundance and large flux at low growth rates mark it as an important alternative to the TCA cycle in these conditions.

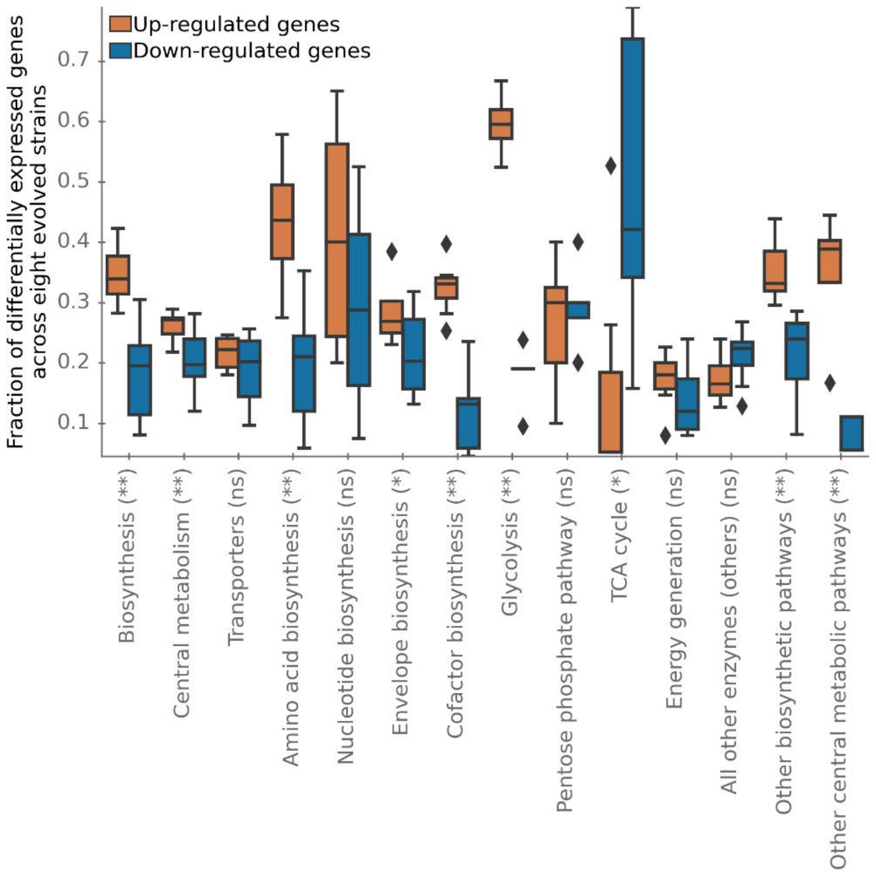

Fig. S11. Fraction of differentially expressed genes in evolved strains grown on glucose minimal medium across pathways. *P*-values of two-sided paired *t*-tests (n = 8) are denoted in brackets as follows: ** for $p < 0.01$, * for $p < 0.1$, and ns for $p \geq 0.1$ (non-significant).

Supplementary References

1. Scott M, Gunderson CW, Mateescu EM, Zhang Z, Hwa T. 2010. Interdependence of cell growth and gene expression: origins and consequences. Science 330:1099–102.

2. Dai X, Zhu M, Warren M, Balakrishnan R, Patsalo V, Okano H, Williamson JR, Fredrick K, Wang Y-P, Hwa T. 2016. Reduction of translating ribosomes enables Escherichia coli to maintain elongation rates during slow growth. Nat Microbiol 2:16231.

3. Si F, Li D, Cox SE, Sauls JT, Azizi O, Sou C, Schwartz AB, Erickstad MJ, Jun Y, Li X, Jun S. 2017. Invariance of Initiation Mass and Predictability of Cell Size in Escherichia coli. Curr Biol 27:1278–1287.

4. Galperin MY, Wolf YI, Makarova KS, Vera Alvarez R, Landsman D, Koonin EV. 2021. COG database update: focus on microbial diversity, model organisms, and widespread pathogens. Nucleic Acids Res 49:D274–D281.

5. Schmidt A, Kochanowski K, Vedelaar S, Ahrne E, Volkmer B, Callipo L, Knoops K, Bauer M, Aebersold R, Heinemann M. 2016. The quantitative and condition-dependent Escherichia coli proteome. Nat Biotechnol 34:104–110.

6. Valgepea K, Adamberg K, Seiman A, Vilu R. 2013. Escherichia coli achieves faster growth by increasing catalytic and translation rates of proteins. Mol Biosyst 9:2344–2358.

7. Peebo K, Valgepea K, Maser A, Nahku R, Adamberg K, Vilu R. 2015. Proteome reallocation in Escherichia coli with increasing specific growth rate. Mol BioSyst 11:1184–1193.

8. Fischer E, Sauer U. 2003. A Novel Metabolic Cycle Catalyzes Glucose Oxidation and Anaplerosis in Hungry Escherichia coli. J Biol Chem 278:46446–46451.

9. Nanchen A, Schicker A, Sauer U. 2006. Nonlinear Dependency of Intracellular Fluxes on Growth Rate in Miniaturized Continuous Cultures of Escherichia coli. Appl Environ Microbiol 72:1164–1172.

10. Gerosa L, Haverkorn Van Rijsewijk BRB, Christodoulou D, Kochanowski K, Schmidt TSB, Noor E, Sauer U. 2015. Pseudo-transition Analysis Identifies the Key Regulators of Dynamic Metabolic Adaptations from Steady-State Data. Cell Syst 1:270–282.

11. Haverkorn van Rijsewijk BRB, Nanchen A, Nallet S, Kleijn RJ, Sauer U. 2011. Large-scale 13C-flux analysis reveals distinct transcriptional control of respiratory and fermentative metabolism in Escherichia coli. Mol Syst Biol 7:477.

12. Ishii N, Nakahigashi K, Baba T, Robert M, Soga T, Kanai A, Hirasawa T, Naba M, Hirai K, Hoque A, Ho PY, Kakazu Y, Sugawara K, Igarashi S, Harada S, Masuda T, Sugiyama N, Togashi T, Hasegawa M, Takai Y, Yugi K, Arakawa K, Iwata N, Toya Y, Nakayama Y, Nishioka T, Shimizu K, Mori H, Tomita M. 2007. Multiple high-throughput analyses monitor the response of E. coli to perturbations. Science 316:593–7.

13. Rui B, Shen T, Zhou H, Liu J, Chen J, Pan X, Liu H, Wu J, Zheng H, Shi Y. 2010. A systematic investigation of Escherichia coli central carbon metabolism in response to superoxide stress. BMC Syst Biol 4:122.

Table S1: Stoichiometry of components in biomass objective function in the studied conditions

Metabolite	Chemostat glucose-limited (μ=0.12 per h)	Chemostat glucose-limited (μ=0.2	Galactose (μ=0.26 per h)	Acetate (μ=0.3 per h)	Chemostat glucose-limited (μ=0.35	Pyruvate (μ=0.4 per h)	Fumarate (μ=0.42 per h)	Succinate (μ=0.44 per h)	Glucosamine (μ=0.46 per h)	Glycerol (μ=0.47 per h)	Mannose (μ=0.47 per h)	Chemostat glucose-limited (μ=0.5	Osmotic-stress glucose (μ=0.55 per h)	Xylose (μ=0.55 per h)	Glucose (μ=0.58 per h)	pH6 glucose (μ=0.63 per h)	Fructose (μ=0.65 per h)	42°C glucose (μ=0.66 per h)
ala__L[c]	-0.60029	-0.59392	-0.58928	-0.58625	-0.58252	-0.57886	-0.57742	-0.57598	-0.57456	-0.57386	-0.57386	-0.57175	-0.56829	-0.56829	-0.56625	-0.5629	-0.56157	-0.56091
arg__L[c]	-0.345659	-0.34199	-0.33932	-0.33757	-0.33542	-0.33332	-0.33249	-0.33166	-0.33084	-0.33044	-0.33044	-0.32922	-0.32723	-0.32723	-0.32606	-0.32413	-0.32336	-0.32298
asn__L[c]	-0.281694	-0.27871	-0.27653	-0.2751	-0.27335	-0.27164	-0.27096	-0.27029	-0.26962	-0.26929	-0.26929	-0.2683	-0.26668	-0.26668	-0.26572	-0.26415	-0.26352	-0.26322
asp__L[c]	-0.281694	-0.27871	-0.27653	-0.2751	-0.27335	-0.27164	-0.27096	-0.27029	-0.26962	-0.26929	-0.26929	-0.2683	-0.26668	-0.26668	-0.26572	-0.26415	-0.26352	-0.26322
cys__L[c]	-0.107019	-0.10588	-0.10506	-0.10452	-0.10385	-0.1032	-0.10294	-0.10269	-0.10243	-0.10231	-0.10231	-0.10193	-0.10132	-0.10132	-0.10095	-0.10035	-0.10012	-0.1
gln__L[c]	-0.307525	-0.30426	-0.30189	-0.30033	-0.29842	-0.29655	-0.29581	-0.29507	-0.29434	-0.29398	-0.29398	-0.2929	-0.29113	-0.29113	-0.29009	-0.28837	-0.28769	-0.28735
glu__L[c]	-0.307525	-0.30426	-0.30189	-0.30033	-0.29842	-0.29655	-0.29581	-0.29507	-0.29434	-0.29398	-0.29398	-0.2929	-0.29113	-0.29113	-0.29009	-0.28837	-0.28769	-0.28735
gly[c]	-0.715921	-0.70833	-0.70279	-0.69917	-0.69472	-0.69036	-0.68864	-0.68693	-0.68524	-0.68439	-0.68439	-0.68188	-0.67776	-0.67776	-0.67533	-0.67132	-0.66974	-0.66896
his__L[c]	-0.11071	-0.10954	-0.10868	-0.10812	-0.10743	-0.10676	-0.10649	-0.10623	-0.10596	-0.10583	-0.10583	-0.10545	-0.10481	-0.10481	-0.10443	-0.10381	-0.10357	-0.10345
ile__L[c]	-0.339508	-0.33591	-0.33328	-0.33157	-0.32946	-0.32739	-0.32657	-0.32576	-0.32496	-0.32456	-0.32456	-0.32337	-0.32141	-0.32141	-0.32026	-0.31836	-0.31761	-0.31724
leu__L[c]	-0.526485	-0.5209	-0.51683	-0.51417	-0.5109	-0.50769	-0.50642	-0.50517	-0.50392	-0.5033	-0.5033	-0.50145	-0.49842	-0.49842	-0.49663	-0.49369	-0.49253	-0.49195
lys__L[c]	-0.401013	-0.39676	-0.39366	-0.39163	-0.38914	-0.3867	-0.38573	-0.38478	-0.38383	-0.38335	-0.38335	-0.38195	-0.37964	-0.37964	-0.37827	-0.37603	-0.37515	-0.37471
met__L[c]	-0.179595	-0.17769	-0.1763	-0.17539	-0.17428	-0.17318	-0.17275	-0.17232	-0.1719	-0.17169	-0.17169	-0.17106	-0.17002	-0.17002	-0.16941	-0.16841	-0.16801	-0.16781
phe__L[c]	-0.216498	-0.2142	-0.21253	-0.21143	-0.21009	-0.20877	-0.20825	-0.20773	-0.20722	-0.20696	-0.20696	-0.20621	-0.20496	-0.20496	-0.20422	-0.20301	-0.20253	-0.2023
pro__L[c]	-0.258322	-0.25558	-0.25358	-0.25228	-0.25067	-0.2491	-0.24848	-0.24786	-0.24725	-0.24695	-0.24695	-0.24604	-0.24455	-0.24455	-0.24367	-0.24223	-0.24166	-0.24138
ser__L[c]	-0.252172	-0.2495	-0.24755	-0.24627	-0.24471	-0.24317	-0.24256	-0.24196	-0.24136	-0.24107	-0.24107	-0.24018	-0.23873	-0.23873	-0.23787	-0.23646	-0.23591	-0.23563
thr__L[c]	-0.296455	-0.29331	-0.29102	-0.28952	-0.28768	-0.28587	-0.28516	-0.28445	-0.28375	-0.2834	-0.2834	-0.28236	-0.28065	-0.28065	-0.27965	-0.27799	-0.27733	-0.27701
trp__L[c]	-0.066426	-0.06572	-0.06521	-0.06487	-0.06446	-0.06405	-0.06389	-0.06374	-0.06358	-0.0635	-0.0635	-0.06327	-0.06289	-0.06289	-0.06266	-0.06229	-0.06214	-0.06207
tyr__L[c]	-0.161143	-0.15943	-0.15819	-0.15737	-0.15637	-0.15539	-0.155	-0.15462	-0.15424	-0.15405	-0.15405	-0.15348	-0.15255	-0.15255	-0.15201	-0.15111	-0.15075	-0.15057
val__L[c]	-0.494502	-0.48926	-0.48543	-0.48293	-0.47986	-0.47685	-0.47566	-0.47448	-0.47331	-0.47273	-0.47273	-0.47099	-0.46814	-0.46814	-0.46646	-0.4637	-0.46261	-0.46206
ctp[c]	-0.044679	-0.05159	-0.05669	-0.06004	-0.06419	-0.06828	-0.06991	-0.07152	-0.07313	-0.07394	-0.07394	-0.07633	-0.08029	-0.08029	-0.08264	-0.08653	-0.08807	-0.08884
atp[c]	-75.43574	-75.4448	-75.4515	-75.4559	-75.4613	-75.4666	-75.4688	-75.4709	-75.473	-75.4741	-75.4741	-75.4772	-75.4824	-75.4824	-75.4854	-75.4905	-75.4926	-75.4936
gtp[c]	-0.071982	-0.08312	-0.09133	-0.09673	-0.10341	-0.11001	-0.11263	-0.11523	-0.11783	-0.11912	-0.11912	-0.12298	-0.12935	-0.12935	-0.13314	-0.1394	-0.14189	-0.14313
utp[c]	-0.048225	-0.05569	-0.06118	-0.0648	-0.06928	-0.0737	-0.07546	-0.0772	-0.07894	-0.0798	-0.0798	-0.08239	-0.08666	-0.08666	-0.0892	-0.09339	-0.09506	-0.09589
kdo2lipid4[e]	-0.025125	-0.02463	-0.02426	-0.02402	-0.02371	-0.0234	-0.02328	-0.02315	-0.02303	-0.02297	-0.02297	-0.02278	-0.02247	-0.02247	-0.02229	-0.02198	-0.02186	-0.0218
murein5px4p	-0.017942	-0.01759	-0.01733	-0.01715	-0.01693	-0.01671	-0.01662	-0.01653	-0.01645	-0.0164	-0.0164	-0.01627	-0.01605	-0.01605	-0.01592	-0.0157	-0.01561	-0.01557
pe160[p]	-0.082407	-0.08079	-0.07958	-0.07877	-0.07776	-0.07675	-0.07634	-0.07594	-0.07553	-0.07533	-0.07533	-0.07473	-0.07371	-0.07371	-0.07311	-0.0721	-0.07169	-0.07149
pe161[p]	-0.097129	-0.09522	-0.09379	-0.09284	-0.09165	-0.09046	-0.08998	-0.0895	-0.08903	-0.08879	-0.08879	-0.08807	-0.08688	-0.08688	-0.08617	-0.08498	-0.0845	-0.08426
10fthf[c]	-0.000223	-0.00022	-0.00022	-0.00022	-0.00022	-0.00022	-0.00022	-0.00022	-0.00022	-0.00022	-0.00022	-0.00022	-0.00022	-0.00022	-0.00022	-0.00022	-0.00022	-0.00022
2fe2s[c]	-0.000026	-2.6E-05	-2.6E-05	-2.6E-05	-2.6E-05	-2.6E-05	-2.6E-05	-2.6E-05	-2.6E-05	-2.6E-05	-2.6E-05	-2.6E-05	-2.6E-05	-2.6E-05	-2.6E-05	-2.6E-05	-2.6E-05	-2.6E-05
2ohph[c]	-0.000223	-0.00022	-0.00022	-0.00022	-0.00022	-0.00022	-0.00022	-0.00022	-0.00022	-0.00022	-0.00022	-0.00022	-0.00022	-0.00022	-0.00022	-0.00022	-0.00022	-0.00022
4fe4s[c]	-0.00026	-0.00026	-0.00026	-0.00026	-0.00026	-0.00026	-0.00026	-0.00026	-0.00026	-0.00026	-0.00026	-0.00026	-0.00026	-0.00026	-0.00026	-0.00026	-0.00026	-0.00026
amet[c]	-0.000223	-0.00022	-0.00022	-0.00022	-0.00022	-0.00022	-0.00022	-0.00022	-0.00022	-0.00022	-0.00022	-0.00022	-0.00022	-0.00022	-0.00022	-0.00022	-0.00022	-0.00022
btn[c]	-0.000002	-2E-06	-2E-06	-2E-06	-2E-06	-2E-06	-2E-06	-2E-06	-2E-06	-2E-06	-2E-06	-2E-06	-2E-06	-2E-06	-2E-06	-2E-06	-2E-06	-2E-06
ca2[c]	-0.005205	-0.00521	-0.00521	-0.00521	-0.00521	-0.00521	-0.00521	-0.00521	-0.00521	-0.00521	-0.00521	-0.00521	-0.00521	-0.00521	-0.00521	-0.00521	-0.00521	-0.00521
cl[c]	-0.005205	-0.00521	-0.00521	-0.00521	-0.00521	-0.00521	-0.00521	-0.00521	-0.00521	-0.00521	-0.00521	-0.00521	-0.00521	-0.00521	-0.00521	-0.00521	-0.00521	-0.00521
coa[c]	-0.000576	-0.00058	-0.00058	-0.00058	-0.00058	-0.00058	-0.00058	-0.00058	-0.00058	-0.00058	-0.00058	-0.00058	-0.00058	-0.00058	-0.00058	-0.00058	-0.00058	-0.00058
cobalt2[c]	-0.000025	-2.5E-05	-2.5E-05	-2.5E-05	-2.5E-05	-2.5E-05	-2.5E-05	-2.5E-05	-2.5E-05	-2.5E-05	-2.5E-05	-2.5E-05	-2.5E-05	-2.5E-05	-2.5E-05	-2.5E-05	-2.5E-05	-2.5E-05
cu2[c]	-0.000709	-0.00071	-0.00071	-0.00071	-0.00071	-0.00071	-0.00071	-0.00071	-0.00071	-0.00071	-0.00071	-0.00071	-0.00071	-0.00071	-0.00071	-0.00071	-0.00071	-0.00071
datp[c]	-0.026166	-0.02617	-0.02617	-0.02617	-0.02617	-0.02617	-0.02617	-0.02617	-0.02617	-0.02617	-0.02617	-0.02617	-0.02617	-0.02617	-0.02617	-0.02617	-0.02617	-0.02617
dctp[c]	-0.027017	-0.02702	-0.02702	-0.02702	-0.02702	-0.02702	-0.02702	-0.02702	-0.02702	-0.02702	-0.02702	-0.02702	-0.02702	-0.02702	-0.02702	-0.02702	-0.02702	-0.02702
dgtp[c]	-0.027017	-0.02702	-0.02702	-0.02702	-0.02702	-0.02702	-0.02702	-0.02702	-0.02702	-0.02702	-0.02702	-0.02702	-0.02702	-0.02702	-0.02702	-0.02702	-0.02702	-0.02702
dttp[c]	-0.026166	-0.02617	-0.02617	-0.02617	-0.02617	-0.02617	-0.02617	-0.02617	-0.02617	-0.02617	-0.02617	-0.02617	-0.02617	-0.02617	-0.02617	-0.02617	-0.02617	-0.02617
fad[c]	-0.000223	-0.00022	-0.00022	-0.00022	-0.00022	-0.00022	-0.00022	-0.00022	-0.00022	-0.00022	-0.00022	-0.00022	-0.00022	-0.00022	-0.00022	-0.00022	-0.00022	-0.00022
fe2[c]	-0.006715	-0.00672	-0.00672	-0.00672	-0.00672	-0.00672	-0.00672	-0.00672	-0.00672	-0.00672	-0.00672	-0.00672	-0.00672	-0.00672	-0.00672	-0.00672	-0.00672	-0.00672
fe3[c]	-0.007808	-0.00781	-0.00781	-0.00781	-0.00781	-0.00781	-0.00781	-0.00781	-0.00781	-0.00781	-0.00781	-0.00781	-0.00781	-0.00781	-0.00781	-0.00781	-0.00781	-0.00781
k[c]	-0.195193	-0.19519	-0.19519	-0.19519	-0.19519	-0.19519	-0.19519	-0.19519	-0.19519	-0.19519	-0.19519	-0.19519	-0.19519	-0.19519	-0.19519	-0.19519	-0.19519	-0.19519
mg2[c]	-0.008675	-0.00868	-0.00868	-0.00868	-0.00868	-0.00868	-0.00868	-0.00868	-0.00868	-0.00868	-0.00868	-0.00868	-0.00868	-0.00868	-0.00868	-0.00868	-0.00868	-0.00868
mlthf[c]	-0.000223	-0.00022	-0.00022	-0.00022	-0.00022	-0.00022	-0.00022	-0.00022	-0.00022	-0.00022	-0.00022	-0.00022	-0.00022	-0.00022	-0.00022	-0.00022	-0.00022	-0.00022
mn2[c]	-0.000691	-0.00069	-0.00069	-0.00069	-0.00069	-0.00069	-0.00069	-0.00069	-0.00069	-0.00069	-0.00069	-0.00069	-0.00069	-0.00069	-0.00069	-0.00069	-0.00069	-0.00069
mobd[c]	-0.000007	-7E-06	-7E-06	-7E-06	-7E-06	-7E-06	-7E-06	-7E-06	-7E-06	-7E-06	-7E-06	-7E-06	-7E-06	-7E-06	-7E-06	-7E-06	-7E-06	-7E-06
nad[c]	-0.001831	-0.00183	-0.00183	-0.00183	-0.00183	-0.00183	-0.00183	-0.00183	-0.00183	-0.00183	-0.00183	-0.00183	-0.00183	-0.00183	-0.00183	-0.00183	-0.00183	-0.00183
nadp[c]	-0.000447	-0.00045	-0.00045	-0.00045	-0.00045	-0.00045	-0.00045	-0.00045	-0.00045	-0.00045	-0.00045	-0.00045	-0.00045	-0.00045	-0.00045	-0.00045	-0.00045	-0.00045
nh4[c]	-0.013013	-0.01301	-0.01301	-0.01301	-0.01301	-0.01301	-0.01301	-0.01301	-0.01301	-0.01301	-0.01301	-0.01301	-0.01301	-0.01301	-0.01301	-0.01301	-0.01301	-0.01301
ni2[c]	-0.000323	-0.00032	-0.00032	-0.00032	-0.00032	-0.00032	-0.00032	-0.00032	-0.00032	-0.00032	-0.00032	-0.00032	-0.00032	-0.00032	-0.00032	-0.00032	-0.00032	-0.00032
pheme[c]	-0.000223	-0.00022	-0.00022	-0.00022	-0.00022	-0.00022	-0.00022	-0.00022	-0.00022	-0.00022	-0.00022	-0.00022	-0.00022	-0.00022	-0.00022	-0.00022	-0.00022	-0.00022
pydx5p[c]	-0.000223	-0.00022	-0.00022	-0.00022	-0.00022	-0.00022	-0.00022	-0.00022	-0.00022	-0.00022	-0.00022	-0.00022	-0.00022	-0.00022	-0.00022	-0.00022	-0.00022	-0.00022
ribflv[c]	-0.000223	-0.00022	-0.00022	-0.00022	-0.00022	-0.00022	-0.00022	-0.00022	-0.00022	-0.00022	-0.00022	-0.00022	-0.00022	-0.00022	-0.00022	-0.00022	-0.00022	-0.00022
sheme[c]	-0.000223	-0.00022	-0.00022	-0.00022	-0.00022	-0.00022	-0.00022	-0.00022	-0.00022	-0.00022	-0.00022	-0.00022	-0.00022	-0.00022	-0.00022	-0.00022	-0.00022	-0.00022
so4[c]	-0.004338	-0.00434	-0.00434	-0.00434	-0.00434	-0.00434	-0.00434	-0.00434	-0.00434	-0.00434	-0.00434	-0.00434	-0.00434	-0.00434	-0.00434	-0.00434	-0.00434	-0.00434
succoa[c]	-0.000098	-9.8E-05	-9.8E-05	-9.8E-05	-9.8E-05	-9.8E-05	-9.8E-05	-9.8E-05	-9.8E-05	-9.8E-05	-9.8E-05	-9.8E-05	-9.8E-05	-9.8E-05	-9.8E-05	-9.8E-05	-9.8E-05	-9.8E-05
thf[c]	-0.000223	-0.00022	-0.00022	-0.00022	-0.00022	-0.00022	-0.00022	-0.00022	-0.00022	-0.00022	-0.00022	-0.00022	-0.00022	-0.00022	-0.00022	-0.00022	-0.00022	-0.00022
thmpp[c]	-0.000223	-0.00022	-0.00022	-0.00022	-0.00022	-0.00022	-0.00022	-0.00022	-0.00022	-0.00022	-0.00022	-0.00022	-0.00022	-0.00022	-0.00022	-0.00022	-0.00022	-0.00022
udcpdp[c]	-0.000055	-5.5E-05	-5.5E-05	-5.5E-05	-5.5E-05	-5.5E-05	-5.5E-05	-5.5E-05	-5.5E-05	-5.5E-05	-5.5E-05	-5.5E-05	-5.5E-05	-5.5E-05	-5.5E-05	-5.5E-05	-5.5E-05	-5.5E-05
zn2[c]	-0.000341	-0.00034	-0.00034	-0.00034	-0.00034	-0.00034	-0.00034	-0.00034	-0.00034	-0.00034	-0.00034	-0.00034	-0.00034	-0.00034	-0.00034	-0.00034	-0.00034	-0.00034
h2o[c]	-69.12707	-69.1934	-69.2417	-69.2733	-69.3121	-69.3502	-69.3652	-69.3801	-69.395	-69.4023	-69.4023	-69.4242	-69.4602	-69.4602	-69.4815	-69.5164	-69.5302	-69.5371
h[c]	75.37723	75.37723	75.37723	75.37723	75.37723	75.37723	75.37723	75.37723	75.37723	75.37723	75.37723	75.37723	75.37723	75.37723	75.37723	75.37723	75.37723	75.37723
adp[c]	75.37723	75.37723	75.37723	75.37723	75.37723	75.37723	75.37723	75.37723	75.37723	75.37723	75.37723	75.37723	75.37723	75.37723	75.37723	75.37723	75.37723	75.37723
pi[c]	75.37323	75.37323	75.37323	75.37323	75.37323	75.37323	75.37323	75.37323	75.37323	75.37323	75.37323	75.37323	75.37323	75.37323	75.37323	75.37323	75.37323	75.37323
ppi[c]	0.445997	0.476193	0.498384	0.512968	0.530969	0.548721	0.555755	0.56275	0.569708	0.573172	0.573172	0.583511	0.600562	0.600562	0.610685	0.627384	0.634004	0.637302

Table S2: Enzyme turnover numbers and their sources

Reaction id	value	direction	source [1]
DHORTS	14.30024	-1	$k_{app,max}$
OMPDC	58.99106	1	$k_{app,max}$
G5SD	9.681925	1	$k_{app,max}$
CS	31.49134	1	$k_{app,max}$
ICDHyr	10.0849	1	$k_{app,max}$
APRAUR	0.076305	1	$k_{app,max}$
DB4PS	0.091319	1	$k_{app,max}$
RBFK	0.086318	1	$k_{app,max}$
GLYK	1.422746	1	$k_{app,max}$
ASPTA	21.5778	-1	$k_{app,max}$
RBFSb	0.024042	1	$k_{app,max}$
SHCHD2	0.131828	1	$k_{app,max}$
CPPPGO	0.696853	1	$k_{app,max}$
ILETA	8.458744	-1	$k_{app,max}$
VALTA	19.05329	-1	$k_{app,max}$
ORPT	80.85754	-1	$k_{app,max}$
RBFSa	0.07372	1	$k_{app,max}$
PFK_3	108.1692	1	$k_{app,max}$
DHAD2	12.76649	1	$k_{app,max}$
PSCVT	8.659949	1	$k_{app,max}$
ANPRT	3.173382	1	$k_{app,max}$
CHORS	32.64963	1	$k_{app,max}$
IGPS	3.37822	1	$k_{app,max}$
DMATT	0.585096	1	$k_{app,max}$
GRTT	0.585096	1	$k_{app,max}$
UPP3S	0.912843	1	$k_{app,max}$
UPPDC1	0.068513	1	$k_{app,max}$
MOAT	10.66652	1	$k_{app,max}$
UDCPDPS	0.062705	1	$k_{app,max}$
USHD	16.5942	1	$k_{app,max}$
ENO	58.18488	1	$k_{app,max}$
MGSA	805.9263	1	$k_{app,max}$
IMPC	11.8847	-1	$k_{app,max}$
IMPD	1.399624	1	$k_{app,max}$
LPADSS	8.700117	1	$k_{app,max}$
GLUTRS	0.120344	1	$k_{app,max}$
TDSK	16.71266	1	$k_{app,max}$
PGI	129.9447	1	$k_{app,max}$
TMPPP	0.021426	1	$k_{app,max}$
PGK	114.571	-1	$k_{app,max}$
PGL	588.7476	1	$k_{app,max}$
DHQTi	40.31471	1	$k_{app,max}$
IPMD	9.643575	1	$k_{app,max}$
AIRC3	30.13264	-1	$k_{app,max}$
ADSL2r	10.24187	1	$k_{app,max}$
TMDS	4.639885	1	$k_{app,max}$
PPCDC	0.073955	1	$k_{app,max}$
DHAD1	38.41795	1	$k_{app,max}$
FMNAT	0.086318	1	$k_{app,max}$
OHPBAT	0.001678	1	$k_{app,max}$
KARA1	3.787365	-1	$k_{app,max}$
KARA2	1.258562	1	$k_{app,max}$
NADK	2.640962	1	$k_{app,max}$
SUCBZS	0.611389	1	$k_{app,max}$
DHBD	0.936895	1	$k_{app,max}$
ICHORT	0.113636	1	$k_{app,max}$
GLUTRR	12.14911	1	$k_{app,max}$
HEMEOS	0.445764	1	$k_{app,max}$
ADMDC	2.161437	1	$k_{app,max}$
LGTHL	64.4189	1	$k_{app,max}$
ARGSS	1.754436	1	$k_{app,max}$
AGPR	12.40352	-1	$k_{app,max}$
SPMS	0.993811	1	$k_{app,max}$
G1SAT	0.168925	1	$k_{app,max}$
KDOCT2	1.446841	1	$k_{app,max}$
EDTXS1	6.821223	1	$k_{app,max}$
DXPS	1.027865	1	$k_{app,max}$
CDPMEK	4.086811	1	$k_{app,max}$
EDTXS2	3.793857	1	$k_{app,max}$
MECDPS	3.386297	1	$k_{app,max}$
PGAMT	2.249193	-1	$k_{app,max}$
ICL	4.849202	1	$k_{app,max}$
UAGDP	7.491129	1	$k_{app,max}$

[1] Note

$k_{app,max}$: in vivo enzyme turnover number estimated from experimental data by Heckmann *et al.* Ref. (31).

$k_{app,ml}$: in vivo enzyme turnover number estimated from machine learning by Heckmann *et al.* Ref. (31).

k_{cat}: in vitro enzyme turnover number from ccFBA by Desouki Ref. (27).

transporter: transport reactions with no in vivo or in vitro parameters, set to 65 s-1 according to Heckmann *et al.* Ref. (31).

UAGCVT	1.397778	1	$k_{app,max}$
GLUR	32.76217	-1	$k_{app,max}$
NNDPR	0.446503	1	$k_{app,max}$
DMQMT	0.021907	1	$k_{app,max}$
HPPK2	0.31375	1	$k_{app,max}$
PTPATi	1.286563	1	$k_{app,max}$
DXPRIi	3.079024	1	$k_{app,max}$
GTPCII2	0.068128	1	$k_{app,max}$
OHPHM	0.021907	1	$k_{app,max}$
GHMT2r	4.439337	1	$k_{app,max}$
DHORD2	38.53068	1	$k_{app,max}$
SDPDS	102.2286	1	$k_{app,max}$
ADCL	1.553082	1	$k_{app,max}$
TDPDRE	3.910464	1	$k_{app,max}$
TDPDRR	1.226032	1	$k_{app,max}$
UAPGR	5.838932	1	$k_{app,max}$
CTPS2	4.258782	1	$k_{app,max}$
PAPPT3	15.59233	1	$k_{app,max}$
UAGPT3	6.150296	1	$k_{app,max}$
GMHEPPA	23.31693	1	$k_{app,max}$
S7PI	1.63139	1	$k_{app,max}$
AGMHE	0.934696	1	$k_{app,max}$
GMHEPAT	1.806171	1	$k_{app,max}$
FADRx	0.088143	1	$k_{app,max}$
ACODA	10.97911	1	$k_{app,max}$
FCLT	1.301035	1	$k_{app,max}$
HBZOPT	0.567372	1	$k_{app,max}$
PNTK	0.227027	1	$k_{app,max}$
ADSS	1.84019	1	$k_{app,max}$
OCTDPS	1.287727	1	$k_{app,max}$
DPCOAK	1.333897	1	$k_{app,max}$
ASNS2	8.972534	1	$k_{app,max}$
KDOPS	0.763831	1	$k_{app,max}$
KDOPP	18.7249	1	$k_{app,max}$
DMPPS	0.148089	1	$k_{app,max}$
IPDPS	0.888064	1	$k_{app,max}$
DAPE	55.54165	1	$k_{app,max}$
DHDPRy	8.859222	1	$k_{app,max}$
HSK	151.8686	1	$k_{app,max}$
SDPTA	6.698489	-1	$k_{app,max}$
THRS	28.64601	1	$k_{app,max}$
HSST	8.539119	1	$k_{app,max}$
PPNDH	17.86531	1	$k_{app,max}$
DTMPK	7.94476	1	$k_{app,max}$
RHCCE	0.015614	1	$k_{app,max}$
G3PD2	42.97261	-1	$k_{app,max}$
GMPS2	2.395951	1	$k_{app,max}$
GALUi	1.309006	1	$k_{app,max}$
U23GAAT	1.39172	1	$k_{app,max}$
UAAGDS	4.160067	1	$k_{app,max}$
UAMAS	2.786797	1	$k_{app,max}$
UAMAGS	4.523614	1	$k_{app,max}$
P5CR	23.66027	1	$k_{app,max}$
DHPPDA2	0.076305	1	$k_{app,max}$
ASP1DC	0.041348	1	$k_{app,max}$
UGMDDS	8.68655	1	$k_{app,max}$
DBTS	0.003799	1	$k_{app,max}$
UHGADA	12.98895	1	$k_{app,max}$
ATPPRT	3.754474	1	$k_{app,max}$
HISTD	3.179596	1	$k_{app,max}$
DHDPS	7.126217	1	$k_{app,max}$
G1PACT	7.491129	1	$k_{app,max}$
GF6PTA	2.021826	1	$k_{app,max}$
IGPDH	3.333692	1	$k_{app,max}$
PRPPS	17.03327	1	$k_{app,max}$
GLUCYS	0.016512	1	$k_{app,max}$
PRATPP	9.319031	1	$k_{app,max}$
PRMICI	13.48006	1	$k_{app,max}$
GLUPRT	18.65284	1	$k_{app,max}$
SERAT	21.21567	1	$k_{app,max}$
NADS1	0.122167	1	$k_{app,max}$
BPNT	84.84513	1	$k_{app,max}$
ASAD	18.63376	-1	$k_{app,max}$
DAPDC	55.701	1	$k_{app,max}$
GARFT	57.1662	1	$k_{app,max}$
PPND	19.97671	1	$k_{app,max}$

METAT	0.054295	1	$k_{app,max}$
PRAMPC	9.319031	1	$k_{app,max}$
TMPK	0.206249	1	$k_{app,max}$
PPBNGS	0.158583	1	$k_{app,max}$
PPNCL2	0.073955	1	$k_{app,max}$
PRAGSr	13.68985	1	$k_{app,max}$
PRFGS	10.23022	1	$k_{app,max}$
NNATr	5.322368	1	$k_{app,max}$
PSERT	37.41342	1	$k_{app,max}$
AIRC2	48.17105	1	$k_{app,max}$
GAPD	35.76496	1	$k_{app,max}$
HEX1	305.0672	1	$k_{app,max}$
GND	66.7612	1	$k_{app,max}$
MOAT2	10.66652	1	$k_{app,max}$
G6PDH2r	163.6787	1	$k_{app,max}$
ARGSL	8.567866	1	$k_{app,max}$
GLU5K	12.22192	1	$k_{app,max}$
IPPS	8.606867	1	$k_{app,max}$
MOHMT	0.029624	1	$k_{app,max}$
AHCYSNS	0.052337	1	$k_{app,max}$
SERASr	0.209118	1	$k_{app,max}$
CHRPL	0.543619	1	$k_{app,max}$
FBA3	29.27115	1	$k_{app,max}$
PMPK	0.03897	1	$k_{app,max}$
PSP_L	2693.142	1	$k_{app,max}$
ADK3	14.77674	1	$k_{app,max}$
ADSK	85.07205	1	$k_{app,max}$
SHSL1	7.32263	1	$k_{app,max}$
GMHEPK	1.806171	1	$k_{app,max}$
GTPCI	0.009085	1	$k_{app,max}$
CYTK1	14.9248	1	$k_{app,max}$
GK1	37.5363	1	$k_{app,max}$
DHFS	0.119841	1	$k_{app,max}$
ASPO6	1.248945	1	$k_{app,max}$
PRASCSi	5.514622	1	$k_{app,max}$
DHPS2	0.371229	1	$k_{app,max}$
MTHFC	40.91791	1	$k_{app,max}$
MTHFD	40.91791	1	$k_{app,max}$
QULNS	0.724281	1	$k_{app,max}$
GLCTR1	17.76051	1	$k_{app,max}$
GLCTR3	10.85577	1	$k_{app,max}$
HEPT4	6.874304	1	$k_{app,max}$
CBLAT	0.204043	1	$k_{app,max}$
NH4tpp	8586.02	1	$k_{app,max}$
GLYCLTt4pp	0.661979	1	$k_{app,max}$
ACGK	6.482128	1	$k_{app,max}$
HMBS	0.194692	1	$k_{app,max}$
MEPCT	13.96947	1	$k_{app,max}$
DAAD	0.212326	1	$k_{app,max}$
GLCt2pp	266.8132	1	$k_{app,max}$
HSTPT	5.95604	1	$k_{app,max}$
THDPS	1.677663	1	$k_{app,max}$
GLYOX	2609.268	1	$k_{app,max}$
PRAIi	3.37822	1	$k_{app,max}$
MTHFR2	3.720112	1	$k_{app,max}$
MTAN	0.444461	1	$k_{app,max}$
AMAOTr	0.000662	1	$k_{app,max}$
GTHS	0.016597	1	$k_{app,max}$
ACGS	25.92218	1	$k_{app,max}$
HEPT1	15.56121	1	$k_{app,max}$
HEPT2	4.951763	1	$k_{app,max}$
MOAT3C	15.80545	1	$k_{app,max}$
RHAT1	19.01035	1	$k_{app,max}$
GALT1	9.122473	1	$k_{app,max}$
GLCTR2	17.99057	1	$k_{app,max}$
HEPK1	9.747015	1	$k_{app,max}$
HEPT3	12.10135	1	$k_{app,max}$
HEPK2	12.15066	1	$k_{app,max}$
SHCHF	0.131828	1	$k_{app,max}$
SUCBZL	1.396733	1	$k_{app,max}$
PRAIS	14.58303	1	$k_{app,max}$
PANTS	0.028019	1	$k_{app,max}$
GCALDD	0.042906	1	$k_{app,max}$
HISTP	3.333692	1	$k_{app,max}$
KAS15	14.98418	1	$k_{app,max}$
T2DECAI	2.047433	1	$k_{app,max}$

3OAS161	1.876475	1	$k_{app,max}$
DHQS	38.1248	1	$k_{app,max}$
EAR161x	1.491904	1	$k_{app,max}$
DASYN160	111.4841	1	$k_{app,max}$
DASYN161	86.71167	1	$k_{app,max}$
DASYN181	44.69031	1	$k_{app,max}$
PGCD	37.46429	1	$k_{app,max}$
PG161abcpp	0.832095	1	$k_{app,max}$
3OAS141	1.876475	1	$k_{app,max}$
3OAR121	3.763232	1	$k_{app,max}$
ADSL1r	4.036562	1	$k_{app,max}$
AICART	11.8847	1	$k_{app,max}$
PG160abcpp	1.069765	1	$k_{app,max}$
DHBS	0.509428	1	$k_{app,max}$
PPPGO3	0.184466	1	$k_{app,max}$
OMBZLM	0.013342	1	$k_{app,max}$
EAR141x	1.491904	1	$k_{app,max}$
EAR181x	0.507402	1	$k_{app,max}$
TPI	193.9066	1	$k_{app,max}$
PERD	0.016316	1	$k_{app,max}$
5DOAN	0.029921	1	$k_{app,max}$
3OAR120	4.480061	1	$k_{app,max}$
SO4t2pp	248.6991	1	$k_{app,max}$
DHNAOT4	0.356291	1	$k_{app,max}$
PSSA161	2.879332	1	$k_{app,max}$
PSD160	5.497192	1	$k_{app,max}$
PSD161	4.275621	1	$k_{app,max}$
COLIPAabcpp	0.808873	1	$k_{app,max}$
3OAR181	1.27989	1	$k_{app,max}$
EAR140x	1.350815	1	$k_{app,max}$
3OAS181	1.316407	1	$k_{app,max}$
AGPAT160	30.0267	1	$k_{app,max}$
PGSA160	12.65921	1	$k_{app,max}$
3OAR140	4.265518	1	$k_{app,max}$
3OAR160	3.192801	1	$k_{app,max}$
PGSA161	9.846652	1	$k_{app,max}$
3OAR141	3.763232	1	$k_{app,max}$
3OAR161	3.763232	1	$k_{app,max}$
EAR121x	1.491904	1	$k_{app,max}$
PE160abcpp	3.155509	1	$k_{app,max}$
PE161abcpp	2.454306	1	$k_{app,max}$
PG181abcpp	0.428799	1	$k_{app,max}$
PGSA181	5.074589	1	$k_{app,max}$
PSSA160	3.701974	1	$k_{app,max}$
PSSA181	1.484009	1	$k_{app,max}$
AGPAT181	12.03672	1	$k_{app,max}$
3OAR60	8.243372	1	$k_{app,max}$
3OAR80	8.243372	1	$k_{app,max}$
EAR100x	1.776085	1	$k_{app,max}$
EAR120x	1.776085	1	$k_{app,max}$
EAR160x	1.265761	1	$k_{app,max}$
3OAS121	1.876475	1	$k_{app,max}$
3OAR40	8.243372	1	$k_{app,max}$
3OAR100	8.243293	1	$k_{app,max}$
AGPAT161	23.3546	1	$k_{app,max}$
EAR40x	3.268021	1	$k_{app,max}$
PSD181	2.203657	1	$k_{app,max}$
EAR60x	3.268021	1	$k_{app,max}$
EAR80x	3.268021	1	$k_{app,max}$
PE181abcpp	1.264962	1	$k_{app,max}$
ZN2tpp	0.431349	1	$k_{app,max}$
CAt6pp	1.058014	-1	$k_{app,max}$
AMMQLT8	0.013342	1	$k_{app,max}$
DALAt2pp	3.342165	1	$k_{app,max}$
FE2tpp	10.82699	1	$k_{app,max}$
MN2tpp	0.87601	1	$k_{app,max}$
MALCOAMT	0.002455	1	$k_{app,max}$
CU2tpp	0.897312	1	$k_{app,max}$
OGMEACPS	9.57E-05	1	$k_{app,max}$
OGMEACPR	5.26E-05	1	$k_{app,max}$
OPMEACPS	2.62E-05	1	$k_{app,max}$
MPTAT	0.077786	1	$k_{app,max}$
OPMEACPD	9.10E-05	1	$k_{app,max}$
EPMEACPR	2.09E-05	1	$k_{app,max}$
AOXSr2	0.001779	1	$k_{app,max}$
OGMEACPD	9.10E-05	1	$k_{app,max}$

EGMEACPR	2.09E-05	1	$k_{app,max}$
OPMEACPR	5.26E-05	1	$k_{app,max}$
PMEACPE	0.002756	1	$k_{app,max}$
ICYSDS	0.021592	1	$k_{app,max}$
I4FE4SR	0.01136	1	$k_{app,max}$
BMOCOS	0.010176	1	$k_{app,max}$
FESR	4.942637	1	$k_{app,max}$
BTS5	0.000739	1	$k_{app,max}$
LIPOS	0.000242	1	$k_{app,max}$
MPTSS	0.380772	1	$k_{app,max}$
LIPOCT	0.002578	1	$k_{app,max}$
VPAMTr	49.25294	-1	$k_{app,max}$
DHNPA2r	2.917904	1	$k_{app,max}$
SEPHCHCS	1.474107	1	$k_{app,max}$
DHNCOAS	0.052986	1	$k_{app,max}$
DHNCOAT	0.683201	1	$k_{app,max}$
TYRL	0.014337	1	$k_{app,max}$
MOCOS	0.011404	1	$k_{app,max}$
CYTDK2	3.633271	1	$k_{app,ml}$
NDPK5	5.004318	1	$k_{app,ml}$
NDPK5	3.760914	-1	$k_{app,ml}$
NDPK6	0.096211	1	$k_{app,ml}$
NDPK6	4.9978	-1	$k_{app,ml}$
NDPK8	3.948568	1	$k_{app,ml}$
NDPK8	2.493469	-1	$k_{app,ml}$
PYNP2r	6.931895	1	$k_{app,ml}$
PYNP2r	3.136084	-1	$k_{app,ml}$
ICDHyr	3.74751	-1	$k_{app,ml}$
ALATA_L2	3.880836	1	$k_{app,ml}$
DURIPP	3.250846	1	$k_{app,ml}$
ACALD	16.37094	1	$k_{app,ml}$
ACALD	8.194523	-1	$k_{app,ml}$
PTRCTA	3.899522	1	$k_{app,ml}$
CYSDS	2.611557	1	$k_{app,ml}$
MAN6PI	2.817264	-1	$k_{app,ml}$
TRPAS2	3.364753	-1	$k_{app,ml}$
ALATA_L	12.19887	-1	$k_{app,ml}$
PPM	2.86429	1	$k_{app,ml}$
ACP1_FMN	3.891823	1	$k_{app,ml}$
NDP3	5.563382	1	$k_{app,ml}$
CDPPH	5.226493	1	$k_{app,ml}$
NDP7	5.226493	1	$k_{app,ml}$
ALATA_D2	3.880836	1	$k_{app,ml}$
PMDPHT	0.188859	1	$k_{app,ml}$
GTHOr	2.287207	-1	$k_{app,ml}$
GLYCTO2	1.57191	1	$k_{app,ml}$
GLYCTO3	1.484281	1	$k_{app,ml}$
IPPMIb	3.659187	1	$k_{app,ml}$
IPPMIb	8.523155	-1	$k_{app,ml}$
GLYCTO4	1.484013	1	$k_{app,ml}$
ACHBS	7.395987	1	$k_{app,ml}$
ACLS	29.79637	1	$k_{app,ml}$
G3PD5	2.686021	1	$k_{app,ml}$
G1PP	0.056349	1	$k_{app,ml}$
TMDPP	2.991571	-1	$k_{app,ml}$
ECOAH6	4.236997	1	$k_{app,ml}$
ECOAH6	2.418356	-1	$k_{app,ml}$
ENO	53.81454	-1	$k_{app,ml}$
FBA	42.12004	-1	$k_{app,ml}$
HCO3E	6.099103	-1	$k_{app,ml}$
IMPC	4.277784	1	$k_{app,ml}$
ECOAH4	5.827645	1	$k_{app,ml}$
ECOAH4	3.17966	-1	$k_{app,ml}$
NTPP1	23.34322	1	$k_{app,ml}$
URIK1	3.576691	1	$k_{app,ml}$
URIK2	3.633271	1	$k_{app,ml}$
NTPP2	23.34322	1	$k_{app,ml}$
NTPTP2	29.25939	1	$k_{app,ml}$
CYTDK1	3.576691	1	$k_{app,ml}$
AIRC3	7.516071	1	$k_{app,ml}$
TALA	39.83541	1	$k_{app,ml}$
ADSL2r	3.193393	-1	$k_{app,ml}$
TKT1	6.0799	-1	$k_{app,ml}$
RNDR1	0.923364	1	$k_{app,ml}$
RNDR4	0.00941	1	$k_{app,ml}$
LYSAM	3.126748	1	$k_{app,ml}$

LYSAM	3.126748	-1	$k_{app,ml}$
FTHFD	46.06887	1	$k_{app,ml}$
IPPMIa	3.336187	1	$k_{app,ml}$
IPPMIa	8.230083	-1	$k_{app,ml}$
G3PD7	2.589234	1	$k_{app,ml}$
EDD	22.88964	1	$k_{app,ml}$
NADH9	2.403306	1	$k_{app,ml}$
ADK4	4.506135	1	$k_{app,ml}$
ADK4	4.281521	-1	$k_{app,ml}$
INSK	3.979366	1	$k_{app,ml}$
OHPBAT	0.06708	-1	$k_{app,ml}$
NADDP	2.587898	1	$k_{app,ml}$
HEX4	3.469276	1	$k_{app,ml}$
KARA2	2.607869	-1	$k_{app,ml}$
ICHORS_copy2	7.732491	1	$k_{app,ml}$
ICHORS_copy2	6.314789	-1	$k_{app,ml}$
DHBD	2.377994	-1	$k_{app,ml}$
RBP4E	3.032085	-1	$k_{app,ml}$
TARTD	3.088357	1	$k_{app,ml}$
PDXPP	4.055824	1	$k_{app,ml}$
HACD6	3.339417	1	$k_{app,ml}$
HACD6	2.576447	-1	$k_{app,ml}$
ECOAH1	5.435808	1	$k_{app,ml}$
ECOAH2	5.843902	1	$k_{app,ml}$
ECOAH2	3.217962	-1	$k_{app,ml}$
AGPR	3.582858	1	$k_{app,ml}$
ECOAH3	5.841133	1	$k_{app,ml}$
ECOAH3	3.218444	-1	$k_{app,ml}$
ALTRH	2.85554	1	$k_{app,ml}$
GUI1	2.725522	1	$k_{app,ml}$
GUI1	2.725522	-1	$k_{app,ml}$
EDTXS3	3.892001	1	$k_{app,ml}$
PGAMT	3.095659	1	$k_{app,ml}$
EDTXS4	4.834386	1	$k_{app,ml}$
PMANM	2.995768	-1	$k_{app,ml}$
UAG2E	3.055023	-1	$k_{app,ml}$
GLYCL	8.970031	1	$k_{app,ml}$
CMPN	11.29015	1	$k_{app,ml}$
TRE6PH	9.31694	1	$k_{app,ml}$
ICHORS_copy1	7.712237	1	$k_{app,ml}$
HACD4	3.353535	1	$k_{app,ml}$
HACD4	2.35958	-1	$k_{app,ml}$
CINNDO	2.766831	1	$k_{app,ml}$
DHPPD	2.733627	1	$k_{app,ml}$
HACD5	3.60068	1	$k_{app,ml}$
HACD5	2.500709	-1	$k_{app,ml}$
DHCIND	2.733627	1	$k_{app,ml}$
HACD7	3.281245	1	$k_{app,ml}$
HACD7	2.536963	-1	$k_{app,ml}$
CITL	6.392752	1	$k_{app,ml}$
GOFUCR	2.275287	1	$k_{app,ml}$
DNMPPA	2.750124	1	$k_{app,ml}$
SADT2	4.711474	1	$k_{app,ml}$
OPHBDC	1.522857	1	$k_{app,ml}$
AACPS1	0.473365	1	$k_{app,ml}$
GHMT2r	4.716639	-1	$k_{app,ml}$
LCARS	2.751624	1	$k_{app,ml}$
LCARS	18.49761	-1	$k_{app,ml}$
ALCD19	11.22635	1	$k_{app,ml}$
ALCD19	3.933228	-1	$k_{app,ml}$
RMPA	3.08298	-1	$k_{app,ml}$
SBTPD	2.728479	1	$k_{app,ml}$
SBTPD	2.501926	-1	$k_{app,ml}$
PFK_2	6.244474	1	$k_{app,ml}$
GLTPD	2.635642	1	$k_{app,ml}$
GLTPD	2.59837	-1	$k_{app,ml}$
ACACCT	2.846615	1	$k_{app,ml}$
BUTCT	2.752224	1	$k_{app,ml}$
TGBPA	3.29865	-1	$k_{app,ml}$
MANAO	2.559645	1	$k_{app,ml}$
MANAO	2.955656	-1	$k_{app,ml}$
TAGURr	2.578188	1	$k_{app,ml}$
TAGURr	2.951025	-1	$k_{app,ml}$
2DGULRx	2.17844	1	$k_{app,ml}$
DKGLCNR2y	2.448182	1	$k_{app,ml}$
2DGULRy	2.448182	1	$k_{app,ml}$

DHAPT	1.008448	1	$k_{app,ml}$
UMPK	4.601321	-1	$k_{app,ml}$
RZ5PP	5.385396	1	$k_{app,ml}$
DDPGALA	3.250094	-1	$k_{app,ml}$
DURIK1	6.997138	1	$k_{app,ml}$
G3PD6	2.589234	1	$k_{app,ml}$
NDPK7	4.9978	-1	$k_{app,ml}$
GLYAT	10.53765	1	$k_{app,ml}$
GLYAT	5.520045	-1	$k_{app,ml}$
ADNUC	3.447988	1	$k_{app,ml}$
F6PA	6.249798	1	$k_{app,ml}$
F6PA	11.29118	-1	$k_{app,ml}$
CRNBTCT	3.236285	1	$k_{app,ml}$
CRNBTCT	3.236285	-1	$k_{app,ml}$
CRNCBCT	3.236285	1	$k_{app,ml}$
CRNCBCT	3.236285	-1	$k_{app,ml}$
CRNCDH	4.078269	1	$k_{app,ml}$
CRNCDH	4.078269	-1	$k_{app,ml}$
IDOND	2.670143	1	$k_{app,ml}$
IDOND	17.8758	-1	$k_{app,ml}$
IDOND2	17.11058	1	$k_{app,ml}$
NADN	21.87028	1	$k_{app,ml}$
GLYCLTDx	2.401812	1	$k_{app,ml}$
NDPK2	19.10756	1	$k_{app,ml}$
NDPK2	6.490113	-1	$k_{app,ml}$
NDPK3	11.67335	1	$k_{app,ml}$
NDPK3	4.9978	-1	$k_{app,ml}$
HPYRRy	12.34104	1	$k_{app,ml}$
NDPK4	4.9978	-1	$k_{app,ml}$
HETZK	3.805523	1	$k_{app,ml}$
MCOATA	2.622024	-1	$k_{app,ml}$
AST	3.243138	1	$k_{app,ml}$
SGDS	6.736857	1	$k_{app,ml}$
CYNTAH	2.868002	1	$k_{app,ml}$
SDPTA	4.120056	1	$k_{app,ml}$
TMAOR2	2.26961	1	$k_{app,ml}$
DGK1	3.378429	-1	$k_{app,ml}$
DTMPK	3.421685	-1	$k_{app,ml}$
NDPK1	3.292598	-1	$k_{app,ml}$
DDPA	10.95666	1	$k_{app,ml}$
NMNDA	20.73942	1	$k_{app,ml}$
PGMT	22.12188	-1	$k_{app,ml}$
FRUK	63.07576	1	$k_{app,ml}$
TDPADGAT	4.807875	1	$k_{app,ml}$
MALS	32.55933	1	$k_{app,ml}$
X5PL3E	3.45401	1	$k_{app,ml}$
DOGULNR	3.051568	1	$k_{app,ml}$
BETALDHx	2.585938	1	$k_{app,ml}$
MLTG3	0.272841	1	$k_{app,ml}$
DKGLCNR1	2.508822	1	$k_{app,ml}$
NADTRHD	24.51586	1	$k_{app,ml}$
AACPS2	2.004406	1	$k_{app,ml}$
FMNRx2	2.27405	1	$k_{app,ml}$
ARAI	2.740122	-1	$k_{app,ml}$
FCI	2.646269	1	$k_{app,ml}$
FCI	2.646269	-1	$k_{app,ml}$
FCLK	2.90671	1	$k_{app,ml}$
FCLPA	3.027816	1	$k_{app,ml}$
FCLPA	2.841457	-1	$k_{app,ml}$
GSNK	4.019751	1	$k_{app,ml}$
AACPS3	1.470615	1	$k_{app,ml}$
AACPS4	1.572154	1	$k_{app,ml}$
UGLYCH	5.374962	1	$k_{app,ml}$
NTPP3	8.841867	1	$k_{app,ml}$
NTPP6	27.48631	1	$k_{app,ml}$
NTPP7	5.451964	1	$k_{app,ml}$
OP4ENH	4.119825	1	$k_{app,ml}$
HOPNTAL	3.265266	1	$k_{app,ml}$
NTPP8	8.705841	1	$k_{app,ml}$
3HPPPNH	2.676857	1	$k_{app,ml}$
PPCSCT	18.76042	1	$k_{app,ml}$
AKGDH	40.88314	1	$k_{app,ml}$
HPYRRx	0.352116	1	$k_{app,ml}$
GLYCDx	4.506491	1	$k_{app,ml}$
DCYTD	3.76805	1	$k_{app,ml}$
AMANK	3.752667	1	$k_{app,ml}$

UAGAAT	2.99576	-1	$k_{app,ml}$
GART	6.381109	1	$k_{app,ml}$
PRPPS	16.77529	-1	$k_{app,ml}$
H2SO	3.487541	1	$k_{app,ml}$
PRMICI	3.676523	-1	$k_{app,ml}$
GMPR	2.610772	1	$k_{app,ml}$
ASPK	3.322316	-1	$k_{app,ml}$
NTRIR2x	3.284036	1	$k_{app,ml}$
GARFT	13.45849	-1	$k_{app,ml}$
PHETA1	4.269688	1	$k_{app,ml}$
PHETA1	11.59656	-1	$k_{app,ml}$
ASCBPL	6.13234	1	$k_{app,ml}$
ADOCBLS	4.193462	1	$k_{app,ml}$
ADOCBIK	2.721728	1	$k_{app,ml}$
MCITL2	3.450673	1	$k_{app,ml}$
MCITL2	3.298899	-1	$k_{app,ml}$
DXYLK	2.892534	1	$k_{app,ml}$
MDH3	1.502434	1	$k_{app,ml}$
SOTA	3.569773	1	$k_{app,ml}$
DHCINDO	3.193946	1	$k_{app,ml}$
SGSAD	2.418219	1	$k_{app,ml}$
HKNDDH	10.07529	1	$k_{app,ml}$
HKNTDH	9.829484	1	$k_{app,ml}$
PTA2	1.524221	1	$k_{app,ml}$
MDH2	15.33422	1	$k_{app,ml}$
ACOTA	6.936054	-1	$k_{app,ml}$
GPDDA5	13.05643	1	$k_{app,ml}$
PDX5PS	0.091514	1	$k_{app,ml}$
ENTCS	1.514259	1	$k_{app,ml}$
SULR	4.395033	1	$k_{app,ml}$
MANPGH	5.755779	1	$k_{app,ml}$
URDGLYCD	3.287935	1	$k_{app,ml}$
CBMKr	12.49795	1	$k_{app,ml}$
CBMKr	4.845883	-1	$k_{app,ml}$
PRAGSr	4.628824	-1	$k_{app,ml}$
AMANAPEr	4.245241	1	$k_{app,ml}$
AMANAPEr	4.245241	-1	$k_{app,ml}$
ACM6PH	2.703964	1	$k_{app,ml}$
NNATr	3.476746	-1	$k_{app,ml}$
MAN1PT2	4.021793	1	$k_{app,ml}$
AP5AH	11.17604	1	$k_{app,ml}$
GAPD	31.49729	-1	$k_{app,ml}$
MCITS	2.829793	1	$k_{app,ml}$
DM_amob_c	1.484089	1	$k_{app,ml}$
PPM2	2.709613	-1	$k_{app,ml}$
G6PDH2r	7.322026	-1	$k_{app,ml}$
ARGSL	5.957479	-1	$k_{app,ml}$
NADH5	2.403306	1	$k_{app,ml}$
MLTG2	5.614094	1	$k_{app,ml}$
MLTP3	2.619613	1	$k_{app,ml}$
MLTP3	1.569838	-1	$k_{app,ml}$
SPODM	1.887779	1	$k_{app,ml}$
TMK	3.529747	1	$k_{app,ml}$
NTPP4	14.51541	1	$k_{app,ml}$
GMPS	6.336127	1	$k_{app,ml}$
CAT	4.358878	1	$k_{app,ml}$
SERASr	2.660067	-1	$k_{app,ml}$
PPPNDO	2.766831	1	$k_{app,ml}$
UACMAMO	2.462367	1	$k_{app,ml}$
FBA3	6.447201	-1	$k_{app,ml}$
LCADi	2.385278	1	$k_{app,ml}$
ACACT7r	9.086134	1	$k_{app,ml}$
ACACT7r	2.967914	-1	$k_{app,ml}$
ADK3	4.014544	-1	$k_{app,ml}$
ACACT2r	10.82815	1	$k_{app,ml}$
ACACT2r	3.631752	-1	$k_{app,ml}$
BSORy	2.362444	1	$k_{app,ml}$
DMSOR2	2.26961	1	$k_{app,ml}$
ACACT3r	8.939981	1	$k_{app,ml}$
ACACT3r	2.697898	-1	$k_{app,ml}$
ACACT4r	11.08831	1	$k_{app,ml}$
ACACT4r	3.64321	-1	$k_{app,ml}$
HSDy	10.56054	-1	$k_{app,ml}$
ACMAMUT	3.365882	1	$k_{app,ml}$
SSALy	12.18545	1	$k_{app,ml}$
DDGALK	3.806318	1	$k_{app,ml}$

AB6PGH	3.909703	1	$k_{app,ml}$
AHGDx	4.427314	1	$k_{app,ml}$
AHGDx	2.770437	-1	$k_{app,ml}$
3HCINNMH	2.676857	1	$k_{app,ml}$
BSORx	2.895214	1	$k_{app,ml}$
GP4GH	12.84317	1	$k_{app,ml}$
AADDGT	3.399368	1	$k_{app,ml}$
SPODMpp	3.15388	1	$k_{app,ml}$
PHYTSpp	12.8032	1	$k_{app,ml}$
PPTHpp	4.950185	1	$k_{app,ml}$
THD2pp	3.840832	1	$k_{app,ml}$
TMAOR2pp	2.447084	1	$k_{app,ml}$
FDMO	2.330453	1	$k_{app,ml}$
CHOLD	3.420031	1	$k_{app,ml}$
TKT2	3.049336	-1	$k_{app,ml}$
CYTK1	4.524805	-1	$k_{app,ml}$
DMSOR1pp	2.464278	1	$k_{app,ml}$
CYTK2	3.25772	-1	$k_{app,ml}$
ECOAH8	1.932477	1	$k_{app,ml}$
ECOAH8	2.418335	-1	$k_{app,ml}$
GLCDpp	4.155313	1	$k_{app,ml}$
HYD2pp	2.435902	1	$k_{app,ml}$
GK1	5.679219	-1	$k_{app,ml}$
HACD1	3.683205	1	$k_{app,ml}$
HACD1	2.561001	-1	$k_{app,ml}$
HACD2	3.581255	1	$k_{app,ml}$
HACD2	2.501425	-1	$k_{app,ml}$
ECOAH5	5.841235	1	$k_{app,ml}$
ECOAH5	3.218489	-1	$k_{app,ml}$
CYTBDpp	2.540151	1	$k_{app,ml}$
CYTBD2pp	3.52715	1	$k_{app,ml}$
ASPO3	1.043996	1	$k_{app,ml}$
GLYOX3	8.840878	1	$k_{app,ml}$
FDMO3	2.330453	1	$k_{app,ml}$
O16GLCT1	3.027211	1	$k_{app,ml}$
O16AP2pp	2.563836	1	$k_{app,ml}$
O16AP3pp	2.563836	1	$k_{app,ml}$
ACACT5r	8.939981	1	$k_{app,ml}$
ACACT5r	2.697898	-1	$k_{app,ml}$
O16A4Lpp	3.819881	1	$k_{app,ml}$
MALTATr	3.703914	1	$k_{app,ml}$
MALTATr	3.703914	-1	$k_{app,ml}$
DHBSH	7.098512	1	$k_{app,ml}$
MALDDH	2.52877	1	$k_{app,ml}$
ENTERES	17.49672	1	$k_{app,ml}$
TRSARr	4.313351	1	$k_{app,ml}$
TRSARr	34.25961	-1	$k_{app,ml}$
MTHFC	10.24086	-1	$k_{app,ml}$
MTHFD	8.114772	-1	$k_{app,ml}$
LYXI	2.791214	1	$k_{app,ml}$
LADGMDH	7.882974	1	$k_{app,ml}$
ALKP	4.550678	1	$k_{app,ml}$
ALAGLUE	2.794805	1	$k_{app,ml}$
ALAGLUE	2.794805	-1	$k_{app,ml}$
FE3HOXR2	3.487541	1	$k_{app,ml}$
ACACT6r	9.712107	1	$k_{app,ml}$
ACACT6r	3.645638	-1	$k_{app,ml}$
GGGABAH	4.619979	1	$k_{app,ml}$
FRULYSK	3.417939	1	$k_{app,ml}$
FDMO6	2.330453	1	$k_{app,ml}$
LIPAabcpp	2.50442	1	$k_{app,ml}$
LIPACabcpp	2.50442	1	$k_{app,ml}$
ECAP1pp	2.797934	1	$k_{app,ml}$
GGGABADr	2.886939	1	$k_{app,ml}$
GGGABADr	2.285701	-1	$k_{app,ml}$
ECAP2pp	2.546605	1	$k_{app,ml}$
ECA4OALpp	3.819881	1	$k_{app,ml}$
FE3Ri	2.640478	1	$k_{app,ml}$
FE3HOXR3	3.487541	1	$k_{app,ml}$
FEENTERR1	3.487541	1	$k_{app,ml}$
FEENTERR3	3.487541	1	$k_{app,ml}$
LA4NTpp	2.785555	1	$k_{app,ml}$
AGM4PA	3.051655	1	$k_{app,ml}$
RNDR4b	0.078608	1	$k_{app,ml}$
UDPGALPpp	5.821825	1	$k_{app,ml}$
UACGAMPpp	5.821825	1	$k_{app,ml}$

UACGALPpp	5.821825	1	$k_{app,ml}$
UGLCURPpp	5.821825	1	$k_{app,ml}$
GUR1PPpp	7.098512	1	$k_{app,ml}$
ACGAL1PPpp	21.87028	1	$k_{app,ml}$
AM4PCP	3.733613	1	$k_{app,ml}$
AM3PA	3.051655	1	$k_{app,ml}$
UM4PCP	3.733613	1	$k_{app,ml}$
CBLAT	3.411688	-1	$k_{app,ml}$
NTRIR4pp	2.517789	1	$k_{app,ml}$
NTRIR3pp	2.517789	1	$k_{app,ml}$
AGM3PA	3.051655	1	$k_{app,ml}$
ACANTHAT	3.407813	1	$k_{app,ml}$
FECRMR1	3.487541	1	$k_{app,ml}$
FECRMR2	3.487541	1	$k_{app,ml}$
TDP	4.813343	1	$k_{app,ml}$
3PEPTabcpp	2.526631	1	$k_{app,ml}$
4PEPTabcpp	2.525389	1	$k_{app,ml}$
AGM3Pt2pp	3.755548	1	$k_{app,ml}$
AGM4Pt2pp	3.755548	1	$k_{app,ml}$
AGMt2pp	3.755548	1	$k_{app,ml}$
ARBTNR1	3.487541	1	$k_{app,ml}$
ARBTNR2	3.487541	1	$k_{app,ml}$
METSOXR1	2.295099	1	$k_{app,ml}$
METSOXR2	5.168006	1	$k_{app,ml}$
DAPAL	3.73781	1	$k_{app,ml}$
AMPTASECG	11.68583	1	$k_{app,ml}$
ARBTNR3	3.487541	1	$k_{app,ml}$
FEOXAMR1	3.847276	1	$k_{app,ml}$
FEOXAMR2	3.847276	1	$k_{app,ml}$
FEOXAMR3	3.847276	1	$k_{app,ml}$
PTHRpp	4.226436	1	$k_{app,ml}$
FEENTERR2	3.487541	1	$k_{app,ml}$
FE3HOXR1	3.487541	1	$k_{app,ml}$
FECRMR3	3.487541	1	$k_{app,ml}$
FDMO2	2.330453	1	$k_{app,ml}$
LALGP	3.67872	1	$k_{app,ml}$
AGM4PApp	3.143364	1	$k_{app,ml}$
ARABDI	2.646269	1	$k_{app,ml}$
ARABDI	2.646269	-1	$k_{app,ml}$
R5PPpp	4.226436	1	$k_{app,ml}$
R1PK	12.56464	1	$k_{app,ml}$
GRXR	6.532341	1	$k_{app,ml}$
RNDR1b	0.292833	1	$k_{app,ml}$
R15BPK	12.72596	1	$k_{app,ml}$
RNDR2b	0.890565	1	$k_{app,ml}$
FACOAE181	8.936187	1	$k_{app,ml}$
FACOAE160	11.48934	1	$k_{app,ml}$
FACOAE140	3.671227	1	$k_{app,ml}$
CPGNR1	3.487541	1	$k_{app,ml}$
CPGNR2	3.487541	1	$k_{app,ml}$
CPGNR3	3.487541	1	$k_{app,ml}$
AAMYL	6.124081	1	$k_{app,ml}$
AAMYLpp	4.282791	1	$k_{app,ml}$
GGPTRCS	2.435125	1	$k_{app,ml}$
23PDE2pp	3.540425	1	$k_{app,ml}$
23PDE4pp	3.540425	1	$k_{app,ml}$
MN6PP	4.896934	1	$k_{app,ml}$
3KGK	2.823988	1	$k_{app,ml}$
F6PP	4.692017	1	$k_{app,ml}$
FRULYSDG	10.78971	1	$k_{app,ml}$
FRULYSDG	3.768395	-1	$k_{app,ml}$
GDPMNH	3.105539	1	$k_{app,ml}$
GDPDPK	2.457914	1	$k_{app,ml}$
DNTPPA	1.573331	1	$k_{app,ml}$
NTPP11	4.533803	1	$k_{app,ml}$
ALLPI	4.119161	1	$k_{app,ml}$
ALLPI	4.119161	-1	$k_{app,ml}$
ALLK	3.550841	1	$k_{app,ml}$
ALLULPE	4.154883	1	$k_{app,ml}$
ALLULPE	3.402488	-1	$k_{app,ml}$
NADPHQR2	3.564066	1	$k_{app,ml}$
NADPHQR3	3.564066	1	$k_{app,ml}$
NADPHQR4	3.564066	1	$k_{app,ml}$
GLBRAN2	2.464644	1	$k_{app,ml}$
ASR	2.47837	1	$k_{app,ml}$
XTSNH	3.447988	1	$k_{app,ml}$

ECAtpp	9.168891	1	$k_{app,ml}$
O16AT	5.35852	1	$k_{app,ml}$
O16GALFT	3.460466	1	$k_{app,ml}$
O16GLCT2	3.487541	1	$k_{app,ml}$
O16AUNDtpp	8.982407	1	$k_{app,ml}$
O16AP1pp	2.867069	1	$k_{app,ml}$
AGM4PCP	3.733613	1	$k_{app,ml}$
4PCP	3.733613	1	$k_{app,ml}$
4PCPpp	3.280727	1	$k_{app,ml}$
FALDH2	2.672188	-1	$k_{app,ml}$
CBIAT	3.411688	-1	$k_{app,ml}$
PPK2	2.665989	1	$k_{app,ml}$
DHPTPE	5.442813	1	$k_{app,ml}$
DHPTPE	5.442813	-1	$k_{app,ml}$
PPDOy	2.810607	1	$k_{app,ml}$
PPA2	1.569796	1	$k_{app,ml}$
GNK	26.65476	1	$k_{app,ml}$
3NTD7pp	3.487722	1	$k_{app,ml}$
3NTD4pp	3.80098	1	$k_{app,ml}$
RMK	2.881141	1	$k_{app,ml}$
FHL	2.524572	1	$k_{app,ml}$
HYPOE	21.87028	1	$k_{app,ml}$
NTPP5	15.04929	1	$k_{app,ml}$
ACONMT	3.880165	1	$k_{app,ml}$
NADPPPS	7.680184	1	$k_{app,ml}$
THRD_L	27.91808	1	$k_{app,ml}$
NAMNPP	2.633579	1	$k_{app,ml}$
SELNPS	5.118439	1	$k_{app,ml}$
MTRPOX	2.482688	1	$k_{app,ml}$
AGM4PCPpp	3.280727	1	$k_{app,ml}$
CRNCAR	8.097315	1	$k_{app,ml}$
CRNCAR	8.097315	-1	$k_{app,ml}$
RNDR2	1.810068	1	$k_{app,ml}$
ANHMK	3.539172	1	$k_{app,ml}$
DHFR	2.448391	-1	$k_{app,ml}$
NMNN	11.29015	1	$k_{app,ml}$
XAND	2.26533	1	$k_{app,ml}$
HACD3	3.592268	1	$k_{app,ml}$
HACD3	2.493227	-1	$k_{app,ml}$
NNDMBRT	4.321261	1	$k_{app,ml}$
THRA2	66.09963	1	$k_{app,ml}$
PPK	2.665989	1	$k_{app,ml}$
SADH	14.35947	1	$k_{app,ml}$
E4PD	0.038994	-1	$k_{app,ml}$
IG3PS	5.01309	1	$k_{app,ml}$
PDH	71.8999	1	$k_{app,ml}$
DADK	4.360949	1	$k_{app,ml}$
DADK	2.375471	-1	$k_{app,ml}$
HYD3pp	2.435902	1	$k_{app,ml}$
AACPS5	1.294524	1	$k_{app,ml}$
MLTG4	0.272841	1	$k_{app,ml}$
UDPGDC	5.371394	1	$k_{app,ml}$
AMAOTr	3.170838	-1	$k_{app,ml}$
MMCD	4.067714	1	$k_{app,ml}$
UDPKAAT	3.291144	1	$k_{app,ml}$
UDPKAAT	3.65951	-1	$k_{app,ml}$
ULA4NFT	3.740215	1	$k_{app,ml}$
DKGLCNR2x	2.17844	1	$k_{app,ml}$
UPLA4FNT	4.922288	1	$k_{app,ml}$
UPLA4FNF	11.21595	1	$k_{app,ml}$
ULA4Ntppi	7.353592	1	$k_{app,ml}$
MCITD	3.417475	1	$k_{app,ml}$
GLCATr	3.703914	1	$k_{app,ml}$
GLCATr	3.703914	-1	$k_{app,ml}$
ALR2	2.438322	1	$k_{app,ml}$
ALAALAr	2.462285	-1	$k_{app,ml}$
HXAND	2.26533	1	$k_{app,ml}$
G2PPpp	4.226436	1	$k_{app,ml}$
ALDD3y	2.909703	1	$k_{app,ml}$
R5PP	3.409544	1	$k_{app,ml}$
CYSSADS	2.872551	1	$k_{app,ml}$
CPPPGO2	2.407467	1	$k_{app,ml}$
AGM3PH	2.831628	1	$k_{app,ml}$
AGM3PApp	3.143364	1	$k_{app,ml}$
LALDO3	2.75933	1	$k_{app,ml}$
NTP10	12.81817	1	$k_{app,ml}$

GDMANE	2.644533	1	$k_{app,ml}$
LPLIPAL2ATG1	3.251202	1	$k_{app,ml}$
PLIPA1E140pp	2.514084	1	$k_{app,ml}$
LPLIPAL2E140	3.055662	1	$k_{app,ml}$
PLIPA1E160pp	3.462475	1	$k_{app,ml}$
GALKr	10.22032	-1	$k_{app,ml}$
PLIPA1E161pp	2.514084	1	$k_{app,ml}$
AACPS8	2.004406	1	$k_{app,ml}$
AACPS9	0.00694	1	$k_{app,ml}$
LPLIPAL2E180	3.055662	1	$k_{app,ml}$
LPLIPAL2G161	3.055662	1	$k_{app,ml}$
LPLIPAL2G181	3.055662	1	$k_{app,ml}$
HXCT	2.752224	1	$k_{app,ml}$
AACPS6	2.004406	1	$k_{app,ml}$
2AGPEAT160	2.044477	1	$k_{app,ml}$
AACPS7	0.900429	1	$k_{app,ml}$
DURADx	2.372947	1	$k_{app,ml}$
DURADx	2.477274	-1	$k_{app,ml}$
ECOAH7	4.864677	1	$k_{app,ml}$
ECOAH7	2.713135	-1	$k_{app,ml}$
GALCTND	3.347427	1	$k_{app,ml}$
QMO2	1.483889	1	$k_{app,ml}$
QMO3	1.896808	1	$k_{app,ml}$
LPLIPAL2E141	3.055662	1	$k_{app,ml}$
LPLIPAL2E161	3.055662	1	$k_{app,ml}$
LDH_D	5.601872	1	$k_{app,ml}$
LPLIPAL2E181	3.055662	1	$k_{app,ml}$
YUMPS	3.840615	1	$k_{app,ml}$
YUMPS	3.606845	-1	$k_{app,ml}$
LPLIPAL2G180	3.055662	1	$k_{app,ml}$
LPLIPAL2G141	3.055662	1	$k_{app,ml}$
IPDDI	0.577649	-1	$k_{app,ml}$
T2DECAI	2.653291	-1	$k_{app,ml}$
2AGPGAT180	2.044477	1	$k_{app,ml}$
2AGPGAT181	2.044477	1	$k_{app,ml}$
2AGPEAT140	2.044477	1	$k_{app,ml}$
2AGPEAT120	2.044477	1	$k_{app,ml}$
CDAPPA161	5.716151	1	$k_{app,ml}$
2AGPEAT161	2.044477	1	$k_{app,ml}$
2AGPEAT141	2.044477	1	$k_{app,ml}$
2AGPGAT160	2.044477	1	$k_{app,ml}$
PGSA180	5.363519	1	$k_{app,ml}$
PSSA140	2.765518	1	$k_{app,ml}$
2AGPGAT141	2.044477	1	$k_{app,ml}$
FACOAE180	7.34444	1	$k_{app,ml}$
LPLIPAL2ATG1	3.251202	1	$k_{app,ml}$
LPLIPAL2ATG1	3.251202	1	$k_{app,ml}$
LPLIPAL2ATG1	3.251202	1	$k_{app,ml}$
LPLIPAL2ATG1	3.251202	1	$k_{app,ml}$
LPLIPAL2ATG1	3.251202	1	$k_{app,ml}$
LPLIPAL2ATG1	3.251202	1	$k_{app,ml}$
FACOAE100	7.34444	1	$k_{app,ml}$
FACOAE80	0.001362	1	$k_{app,ml}$
FACOAE60	7.34444	1	$k_{app,ml}$
CLPNS180pp	3.757662	1	$k_{app,ml}$
CLPNS180pp	2.569282	-1	$k_{app,ml}$
PLIPA1E120pp	2.514084	1	$k_{app,ml}$
CLPNS181pp	0.194469	1	$k_{app,ml}$
CLPNS181pp	2.569282	-1	$k_{app,ml}$
PLIPA1G120pp	2.514084	1	$k_{app,ml}$
FACOAE141	7.34444	1	$k_{app,ml}$
CLPNS120pp	3.757662	1	$k_{app,ml}$
CLPNS120pp	2.569282	-1	$k_{app,ml}$
ALPATE160pp	2.404651	1	$k_{app,ml}$
FACOAE161	10.52334	1	$k_{app,ml}$
ALPATG160pp	2.404651	1	$k_{app,ml}$
2AGPGAT120	2.044477	1	$k_{app,ml}$
LPLIPAL2ATE1	3.251202	1	$k_{app,ml}$
ALAALAD	3.76856	1	$k_{app,ml}$
2AGPEAT180	2.044477	1	$k_{app,ml}$
LPLIPAL2ATE1	3.251202	1	$k_{app,ml}$
LPLIPAL2ATE1	3.251202	1	$k_{app,ml}$
LPLIPAL2ATE1	3.251202	1	$k_{app,ml}$
LPLIPAL2ATE1	3.251202	1	$k_{app,ml}$
2AGPEAT181	2.044477	1	$k_{app,ml}$
2AGPGAT140	2.044477	1	$k_{app,ml}$

PLIPA1E181pp	2.514084	1	$k_{app,ml}$
PLIPA1G180pp	2.514084	1	$k_{app,ml}$
LPLIPAL2ATE1	3.251202	1	$k_{app,ml}$
LPLIPAL2ATE1	3.251202	1	$k_{app,ml}$
PLIPA1G181pp	2.514084	1	$k_{app,ml}$
2AGPGAT161	2.044477	1	$k_{app,ml}$
AMPTASEPG	11.68583	1	$k_{app,ml}$
ADSL1r	3.15405	-1	$k_{app,ml}$
PGPP140pp	4.251784	1	$k_{app,ml}$
PGPP141pp	4.251784	1	$k_{app,ml}$
PGPP160pp	4.251784	1	$k_{app,ml}$
PGPP180pp	4.251784	1	$k_{app,ml}$
CLPNH141pp	7.098512	1	$k_{app,ml}$
CLPNH160pp	7.098512	1	$k_{app,ml}$
CLPNH161pp	7.098512	1	$k_{app,ml}$
AICART	3.406534	-1	$k_{app,ml}$
CLPNH181pp	7.098512	1	$k_{app,ml}$
PLIPA1E180pp	3.462475	1	$k_{app,ml}$
PLIPA1G140pp	2.514084	1	$k_{app,ml}$
PLIPA1G161pp	2.514084	1	$k_{app,ml}$
CLPNS160pp	0.389207	1	$k_{app,ml}$
CLPNS160pp	2.569282	-1	$k_{app,ml}$
CLPNS161pp	0.326188	1	$k_{app,ml}$
CLPNS161pp	2.569282	-1	$k_{app,ml}$
PAPA120pp	7.234931	1	$k_{app,ml}$
PAPA140pp	7.234931	1	$k_{app,ml}$
GGPTRCO	4.320293	1	$k_{app,ml}$
PAPA141pp	7.234931	1	$k_{app,ml}$
PAPA160pp	7.234931	1	$k_{app,ml}$
PAPA161pp	7.234931	1	$k_{app,ml}$
PAPA180pp	7.234931	1	$k_{app,ml}$
HPYRI	4.277075	1	$k_{app,ml}$
HPYRI	31.1465	-1	$k_{app,ml}$
HYD1pp	2.418675	1	$k_{app,ml}$
AACTOOR	2.496258	1	$k_{app,ml}$
ECAP3pp	2.546605	1	$k_{app,ml}$
TPRDCOAS	7.748391	1	$k_{app,ml}$
ACGAM1PPpp	21.87028	1	$k_{app,ml}$
THIORDXi	5.927244	1	$k_{app,ml}$
PDX5PO2	1.510259	1	$k_{app,ml}$
MDDCP1pp	1.353983	1	$k_{app,ml}$
GUI2	2.725522	1	$k_{app,ml}$
GUI2	2.725522	-1	$k_{app,ml}$
PLIPA1A181pp	2.514084	1	$k_{app,ml}$
PGM	65.56609	1	$k_{app,ml}$
LPLIPAL2A141	2.809181	1	$k_{app,ml}$
MLTG5	0.272841	1	$k_{app,ml}$
G3PAT141	2.571933	1	$k_{app,ml}$
2DOXG6PP	4.038472	1	$k_{app,ml}$
OXAMTC	3.487541	1	$k_{app,ml}$
UDCPPtppi	7.098512	1	$k_{app,ml}$
AGMH	2.831628	1	$k_{app,ml}$
G3PAT181	2.180277	1	$k_{app,ml}$
MPTG	1.881249	1	$k_{app,ml}$
MPTG2	0.156749	1	$k_{app,ml}$
MCTP1App	1.791982	1	$k_{app,ml}$
MCTP2App	0.216502	1	$k_{app,ml}$
CDAPPA160	5.716151	1	$k_{app,ml}$
CDAPPA141	5.716151	1	$k_{app,ml}$
MCTP1Bpp	0.320418	1	$k_{app,ml}$
PERD	0.078893	-1	$k_{app,ml}$
PGSA141	5.363519	1	$k_{app,ml}$
DM_5drib_c	1.525218	1	$k_{app,ml}$
3OAR120	3.967411	-1	$k_{app,ml}$
3HAD80	2.407494	1	$k_{app,ml}$
3HAD120	2.407494	1	$k_{app,ml}$
DSBGGT	3.246589	1	$k_{app,ml}$
EAR180x	2.290886	1	$k_{app,ml}$
AGPAT120	6.658908	1	$k_{app,ml}$
AMPMS2	1.542061	1	$k_{app,ml}$
DM_oxam_c	7.098512	1	$k_{app,ml}$
ALR4x	2.499005	1	$k_{app,ml}$
PGPP120	4.251784	1	$k_{app,ml}$
DAGK120	5.427744	1	$k_{app,ml}$
PSSA180	2.765518	1	$k_{app,ml}$
PSSA141	2.765518	1	$k_{app,ml}$

PSD180	3.061142	1	$k_{app,ml}$
LPLIPAL1G141	3.324473	1	$k_{app,ml}$
LPLIPAL1G180	3.171706	1	$k_{app,ml}$
LPLIPAL1G181	3.324473	1	$k_{app,ml}$
ACONTb	14.12519	-1	$k_{app,ml}$
PLIPA2A140pp	3.462475	1	$k_{app,ml}$
PLIPA2A141pp	3.462475	1	$k_{app,ml}$
PLIPA2A180pp	3.462475	1	$k_{app,ml}$
PLIPA2E141pp	2.514084	1	$k_{app,ml}$
PLIPA2E181pp	2.514084	1	$k_{app,ml}$
PLIPA2G140pp	2.514084	1	$k_{app,ml}$
3HAD161	2.407494	1	$k_{app,ml}$
PLIPA2G160pp	2.514084	1	$k_{app,ml}$
CU1Opp	2.852558	1	$k_{app,ml}$
PLIPA2G181pp	2.514084	1	$k_{app,ml}$
FADRx2	13.29396	1	$k_{app,ml}$
LPLIPAL1E141	3.324473	1	$k_{app,ml}$
LPLIPAL1E160	3.324473	1	$k_{app,ml}$
LPLIPAL1E180	3.324473	1	$k_{app,ml}$
3HAD121	2.407494	1	$k_{app,ml}$
LPLIPAL1E181	3.324473	1	$k_{app,ml}$
LPLIPAL1G160	3.324473	1	$k_{app,ml}$
LPLIPAL1G161	3.324473	1	$k_{app,ml}$
GTHRDHpp	2.907945	1	$k_{app,ml}$
MDDCP2pp	0.082167	1	$k_{app,ml}$
LPLIPAL1G120	3.324473	1	$k_{app,ml}$
PLIPA2E140pp	2.514084	1	$k_{app,ml}$
PLIPA2E160pp	2.514084	1	$k_{app,ml}$
PLIPA2A161pp	2.514084	1	$k_{app,ml}$
PLIPA2A181pp	2.514084	1	$k_{app,ml}$
MDDCP3pp	0.235402	1	$k_{app,ml}$
G3PAT140	2.571933	1	$k_{app,ml}$
G3PAT160	2.853299	1	$k_{app,ml}$
G3PAT161	2.619723	1	$k_{app,ml}$
AGPAT140	6.658908	1	$k_{app,ml}$
MLDCP2Bpp	3.160439	1	$k_{app,ml}$
3OAR140	3.967411	-1	$k_{app,ml}$
CDAPPA140	5.716151	1	$k_{app,ml}$
MLDCP1App	0.103874	1	$k_{app,ml}$
3OAR160	3.967411	-1	$k_{app,ml}$
3HAD181	2.407494	1	$k_{app,ml}$
PGPP160	2.609757	1	$k_{app,ml}$
3HAD141	2.407494	1	$k_{app,ml}$
PGPP180	4.251784	1	$k_{app,ml}$
PGPP161	2.603426	1	$k_{app,ml}$
PGPP181	1.266994	1	$k_{app,ml}$
PSD140	3.061142	1	$k_{app,ml}$
MICITDr	2.729069	1	$k_{app,ml}$
MICITDr	2.729069	-1	$k_{app,ml}$
CDAPPA180	5.716151	1	$k_{app,ml}$
AGM4PH	2.831628	1	$k_{app,ml}$
NTP12	12.44237	1	$k_{app,ml}$
CDAPPA181	5.716151	1	$k_{app,ml}$
PGSA140	5.363519	1	$k_{app,ml}$
LPLIPAL1A141	3.324473	1	$k_{app,ml}$
LPLIPAL1A180	3.324473	1	$k_{app,ml}$
LPLIPAL1E140	3.324473	1	$k_{app,ml}$
LPLIPAL1E161	3.324473	1	$k_{app,ml}$
LPLIPAL1G140	3.324473	1	$k_{app,ml}$
PSD141	3.061142	1	$k_{app,ml}$
MLDCP2App	3.160439	1	$k_{app,ml}$
AM4PA	3.051655	1	$k_{app,ml}$
PGPP120pp	4.251784	1	$k_{app,ml}$
PGPP181pp	4.251784	1	$k_{app,ml}$
CLPNH120pp	7.098512	1	$k_{app,ml}$
PAPA181pp	7.234931	1	$k_{app,ml}$
CLPNH140pp	7.098512	1	$k_{app,ml}$
PGPP140	4.251784	1	$k_{app,ml}$
PGPP141	4.251784	1	$k_{app,ml}$
MDDCP5pp	3.133974	1	$k_{app,ml}$
MLTGY3pp	2.725129	1	$k_{app,ml}$
MDDCP4pp	0.532646	1	$k_{app,ml}$
CLPNS140pp	3.757662	1	$k_{app,ml}$
CLPNS140pp	2.569282	-1	$k_{app,ml}$
CLPNS141pp	3.757662	1	$k_{app,ml}$
CLPNS141pp	2.569282	-1	$k_{app,ml}$

G3PAT180	2.571933	1	$k_{app,ml}$
PLIPA1G160pp	2.514084	1	$k_{app,ml}$
PLIPA1G141pp	2.514084	1	$k_{app,ml}$
LPLIPAL2E160	3.055662	1	$k_{app,ml}$
LPLIPAL2G140	3.055662	1	$k_{app,ml}$
LPLIPAL2G160	3.055662	1	$k_{app,ml}$
PAPA160	7.234931	1	$k_{app,ml}$
PAPA180	7.234931	1	$k_{app,ml}$
PAPA181	7.234931	1	$k_{app,ml}$
LPLIPAL2G120	3.055662	1	$k_{app,ml}$
PETNT161pp	2.758737	1	$k_{app,ml}$
DAGK141	5.427744	1	$k_{app,ml}$
DAGK161	5.427744	1	$k_{app,ml}$
PGPP161pp	4.251784	1	$k_{app,ml}$
MDDEP3pp	0.230219	1	$k_{app,ml}$
MDDEP4pp	3.244021	1	$k_{app,ml}$
PAPA140	7.234931	1	$k_{app,ml}$
MLTGY1pp	2.725129	1	$k_{app,ml}$
3HAD40	2.407494	1	$k_{app,ml}$
PLIPA1E141pp	2.514084	1	$k_{app,ml}$
PAPA141	7.234931	1	$k_{app,ml}$
PAPA161	7.234931	1	$k_{app,ml}$
DSBAO1	3.832589	1	$k_{app,ml}$
DSBAO2	3.832589	1	$k_{app,ml}$
DSBDR	2.587281	1	$k_{app,ml}$
PLIPA2A120pp	3.462475	1	$k_{app,ml}$
PLIPA2A160pp	3.462475	1	$k_{app,ml}$
CLPNH180pp	7.098512	1	$k_{app,ml}$
3OAR60	3.967411	-1	$k_{app,ml}$
3OAR80	3.967411	-1	$k_{app,ml}$
MLDCP3App	0.174891	1	$k_{app,ml}$
MLDCP1Bpp	3.160439	1	$k_{app,ml}$
LPLIPAL2E120	3.055662	1	$k_{app,ml}$
ATHRDHr	2.400645	1	$k_{app,ml}$
ATHRDHr	16.03101	-1	$k_{app,ml}$
LSERDHr	2.452173	-1	$k_{app,ml}$
3OAR40	2.660884	-1	$k_{app,ml}$
3OAR100	3.967411	-1	$k_{app,ml}$
DSERDHr	3.00543	1	$k_{app,ml}$
DSERDHr	4.674328	-1	$k_{app,ml}$
3HAD60	2.745113	1	$k_{app,ml}$
3HAD100	2.407494	1	$k_{app,ml}$
3HAD140	2.745113	1	$k_{app,ml}$
3HAD160	2.745113	1	$k_{app,ml}$
PSD120	3.061142	1	$k_{app,ml}$
AGPAT180	6.658908	1	$k_{app,ml}$
AGPAT141	6.658908	1	$k_{app,ml}$
PAPA120	7.234931	1	$k_{app,ml}$
MDDEP1pp	0.192692	1	$k_{app,ml}$
MDDEP2pp	3.244021	1	$k_{app,ml}$
MLDEP1pp	1.717065	1	$k_{app,ml}$
DAGK180	5.427744	1	$k_{app,ml}$
MLDEP2pp	7.648413	1	$k_{app,ml}$
MLTGY2pp	2.725129	1	$k_{app,ml}$
PLIPA2E120pp	2.514084	1	$k_{app,ml}$
PLIPA2G141pp	2.514084	1	$k_{app,ml}$
MLTGY4pp	2.725129	1	$k_{app,ml}$
PLIPA2G161pp	2.514084	1	$k_{app,ml}$
LPLIPAL1A120	3.324473	1	$k_{app,ml}$
LPLIPAL1A140	3.324473	1	$k_{app,ml}$
LPLIPAL1A181	3.324473	1	$k_{app,ml}$
LPLIPAL1E120	3.324473	1	$k_{app,ml}$
3OAR180	2.660884	-1	$k_{app,ml}$
G3PAT120	2.571933	1	$k_{app,ml}$
PGSA120	5.363519	1	$k_{app,ml}$
3HAD180	2.407494	1	$k_{app,ml}$
CDAPPA120	5.716151	1	$k_{app,ml}$
OMMBLHX3	1.507884	1	$k_{app,ml}$
PSSA120	2.765518	1	$k_{app,ml}$
UM4PL	2.563139	1	$k_{app,ml}$
2MAHMP	3.462365	1	$k_{app,ml}$
NADHPO	4.510757	1	$k_{app,ml}$
ACOAD7f	2.822655	1	$k_{app,ml}$
ACOAD7f	5.366348	-1	$k_{app,ml}$
ACOAD8f	2.822655	1	$k_{app,ml}$
ACOAD8f	2.822655	-1	$k_{app,ml}$

PLIPA1A120pp	3.462475	1	$k_{app,ml}$
ACOAD5f	3.666903	1	$k_{app,ml}$
ACOAD5f	8.701169	-1	$k_{app,ml}$
PLIPA1A140pp	3.462475	1	$k_{app,ml}$
PLIPA1A141pp	3.462475	1	$k_{app,ml}$
PLIPA1A160pp	3.462475	1	$k_{app,ml}$
PLIPA1A161pp	2.514084	1	$k_{app,ml}$
PLIPA1A180pp	3.462475	1	$k_{app,ml}$
LPLIPAL2A120	2.809181	1	$k_{app,ml}$
ACOAD3f	3.639979	1	$k_{app,ml}$
ACOAD3f	8.652383	-1	$k_{app,ml}$
ACOAD2f	2.822655	1	$k_{app,ml}$
ACOAD2f	7.085496	-1	$k_{app,ml}$
ACOAD4f	2.822655	1	$k_{app,ml}$
ACOAD4f	7.085496	-1	$k_{app,ml}$
ACOAD6f	7.639588	-1	$k_{app,ml}$
KDUI	11.50452	1	$k_{app,ml}$
CTBTCAL2	2.501161	1	$k_{app,ml}$
OBTFL	2.620728	1	$k_{app,ml}$
FA161ACPHi	55.65879	1	$k_{app,ml}$
BGLA1	4.74562	1	$k_{app,ml}$
FA141ACPHi	55.65879	1	$k_{app,ml}$
FA160ACPHi	55.65879	1	$k_{app,ml}$
GDPMNP	16.35714	1	$k_{app,ml}$
OPHHX3	1.507884	1	$k_{app,ml}$
FA120ACPHi	55.65879	1	$k_{app,ml}$
FA80ACPHi	55.65879	1	$k_{app,ml}$
FA140ACPHi	55.65879	1	$k_{app,ml}$
FA100ACPHi	55.65879	1	$k_{app,ml}$
CRNDCAL2	2.501161	1	$k_{app,ml}$
CRNCAL2	2.501161	1	$k_{app,ml}$
PLIPA2E161pp	2.514084	1	$k_{app,ml}$
PLIPA2E180pp	2.514084	1	$k_{app,ml}$
FRULYSE	3.410595	1	$k_{app,ml}$
FRULYSE	3.410595	-1	$k_{app,ml}$
PLIPA2G120pp	2.514084	1	$k_{app,ml}$
PLIPA2G180pp	2.127453	1	$k_{app,ml}$
LPLIPAL1A161	3.324473	1	$k_{app,ml}$
LPLIPAL1A160	3.324473	1	$k_{app,ml}$
LPLIPAL2A140	2.809181	1	$k_{app,ml}$
LPLIPAL2A160	2.809181	1	$k_{app,ml}$
LPLIPAL2A161	2.809181	1	$k_{app,ml}$
LPLIPAL2A180	2.809181	1	$k_{app,ml}$
LPLIPAL2A181	2.809181	1	$k_{app,ml}$
DM_aacald_c	7.098512	1	$k_{app,ml}$
UM3PL	2.563139	1	$k_{app,ml}$
GALCTLO	2.881793	1	$k_{app,ml}$
POR5	3.980006	1	$k_{app,ml}$
POR5	6.197212	-1	$k_{app,ml}$
HACD8	11.80702	1	$k_{app,ml}$
HACD8	8.339206	-1	$k_{app,ml}$
CTECOAI6	6.982129	-1	$k_{app,ml}$
SELGTHR	3.487541	1	$k_{app,ml}$
SELGTHR3	2.723451	1	$k_{app,ml}$
THRA	4.288814	1	$k_{app,ml}$
DGUNC	3.786562	1	$k_{app,ml}$
XYLK2	2.797534	1	$k_{app,ml}$
GLDBRAN2	2.334182	1	$k_{app,ml}$
S2FE2SR	2.15723	1	$k_{app,ml}$
CDGUNPD	3.530083	1	$k_{app,ml}$
ACACT8r	2.697898	1	$k_{app,ml}$
ACACT8r	3.686624	-1	$k_{app,ml}$
CTECOAI7	6.982129	1	$k_{app,ml}$
CTECOAI7	11.53259	-1	$k_{app,ml}$
3AMACHYD	33.13717	1	$k_{app,ml}$
FDH4pp	25.79707	1	$k_{app,ml}$
FDH5pp	0.00855	1	$k_{app,ml}$
ACPPAT140	2.410531	1	$k_{app,ml}$
APG3PAT140	4.890185	1	$k_{app,ml}$
LDGUNPD	3.321879	1	$k_{app,ml}$
APG3PAT141	4.890185	1	$k_{app,ml}$
MOADSUx	1.495763	1	$k_{app,ml}$
APG3PAT181	3.416439	1	$k_{app,ml}$
APH140	3.12568	1	$k_{app,ml}$
APH160	3.12568	1	$k_{app,ml}$
SCYSDS	3.198283	1	$k_{app,ml}$

S2FE2SS	2.059549	1	$k_{app,ml}$
S4FE4SR	2.467245	1	$k_{app,ml}$
CCGS	2.838964	1	$k_{app,ml}$
REPHACCOAI	4.078866	1	$k_{app,ml}$
REPHACCOAI	4.078866	-1	$k_{app,ml}$
ACPPAT141	2.410531	1	$k_{app,ml}$
ACPPAT161	2.087464	1	$k_{app,ml}$
ACPPAT180	2.410531	1	$k_{app,ml}$
APG3PAT160	4.541992	1	$k_{app,ml}$
APG3PAT161	4.15706	1	$k_{app,ml}$
APG3PAT180	4.890185	1	$k_{app,ml}$
MECDPDH5	1.869982	1	$k_{app,ml}$
CDGR	2.505639	1	$k_{app,ml}$
RNTR1c2	2.218289	1	$k_{app,ml}$
RNTR2c2	2.260405	1	$k_{app,ml}$
S2FE2ST	2.68241	1	$k_{app,ml}$
BWCOS	2.326042	1	$k_{app,ml}$
RNTR3c2	1.99679	1	$k_{app,ml}$
RNTR4c2	1.498421	1	$k_{app,ml}$
BWCOGDS1	3.699292	1	$k_{app,ml}$
BWCOGDS2	3.699292	1	$k_{app,ml}$
CDGS	3.322615	1	$k_{app,ml}$
DHNPTE	4.361322	1	$k_{app,ml}$
DHNPTE	4.361322	-1	$k_{app,ml}$
DHMPTR	2.523332	1	$k_{app,ml}$
I2FE2SS	1.504195	1	$k_{app,ml}$
I2FE2ST	1.493889	1	$k_{app,ml}$
OXCOAHDH	2.613249	1	$k_{app,ml}$
DHACOAH	4.178044	1	$k_{app,ml}$
DHACOAH	4.178044	-1	$k_{app,ml}$
I2FE2SS2	1.502792	1	$k_{app,ml}$
MSAR	2.427211	1	$k_{app,ml}$
I4FE4ST	1.517756	1	$k_{app,ml}$
S4FE4ST	2.68241	1	$k_{app,ml}$
FOMETRi	3.875567	1	$k_{app,ml}$
S2FE2SS2	2.059549	1	$k_{app,ml}$
SELGTHR2	2.723451	1	$k_{app,ml}$
GDPTPDP	3.524073	1	$k_{app,ml}$
COLIPAKpp	3.829001	1	$k_{app,ml}$
ACPPAT120	2.410531	1	$k_{app,ml}$
CPMPS	0.099392	1	$k_{app,ml}$
LIPATPT	3.449978	1	$k_{app,ml}$
LIPAMPL	2.586752	1	$k_{app,ml}$
ALDD19xr	4.68611	-1	$k_{app,ml}$
MOGDS	1.489004	1	$k_{app,ml}$
CPH4S	3.212318	1	$k_{app,ml}$
WCOS	2.326042	1	$k_{app,ml}$
SHCHCS3	1.552127	1	$k_{app,ml}$
VPAMTr	4.407651	1	$k_{app,ml}$
PYK4	1.48396	1	$k_{app,ml}$
NADPHXD	2.314239	1	$k_{app,ml}$
NADPHXE	3.527344	1	$k_{app,ml}$
NADPHXE	3.527344	-1	$k_{app,ml}$
DM_4crsol_c	1.524038	1	$k_{app,ml}$
3OXCOAT	3.170324	1	$k_{app,ml}$
URACPAH	5.20523	1	$k_{app,ml}$
FE3DHBZS3R	3.665524	1	$k_{app,ml}$
DXYLTD	3.444396	1	$k_{app,ml}$
DXYLTD	3.444396	-1	$k_{app,ml}$
2DDARAA	3.116683	1	$k_{app,ml}$
2DDARAA	2.689897	-1	$k_{app,ml}$
2DGULRGx	2.17844	1	$k_{app,ml}$
2DGULRGy	2.448182	1	$k_{app,ml}$
AP4AS	2.655165	1	$k_{app,ml}$
GNP	1.572874	1	$k_{app,ml}$
ALLTAMH2	6.517188	1	$k_{app,ml}$
UGCIAMH	10.07596	1	$k_{app,ml}$
POAACR	2.992232	1	$k_{app,ml}$
METGLCUR	3.104906	1	$k_{app,ml}$
ACOAD1fr	9.260675	1	$k_{app,ml}$
FEENTERES	7.485505	1	$k_{app,ml}$
FEENTERR4	3.665524	1	$k_{app,ml}$
ACPPAT160	2.23661	1	$k_{app,ml}$
ALR2x	2.731465	1	$k_{app,ml}$
OMPHHXy	1.508531	1	$k_{app,ml}$
13PPDH2	2.791839	1	$k_{app,ml}$

13PPDH2	3.283804	-1	$k_{app,ml}$
APCS	2.507151	1	$k_{app,ml}$
ACPPAT181	1.990575	1	$k_{app,ml}$
SQVOSI	6.062962	1	$k_{app,ml}$
6D6SPA	9.53266	1	$k_{app,ml}$
ThDPAT	3.651278	1	$k_{app,ml}$
GHBDHx	2.477119	1	$k_{app,ml}$
GHBDHx	2.757048	-1	$k_{app,ml}$
APPLDHr	2.499005	1	$k_{app,ml}$
APPLDHr	2.827125	-1	$k_{app,ml}$
APH141	3.12568	1	$k_{app,ml}$
AI2K	1.660644	1	$k_{app,ml}$
G2PP	45.26527	1	$k_{app,ml}$
APH161	3.12568	1	$k_{app,ml}$
DHNPA2r	5.202772	-1	$k_{app,ml}$
SHGO	4.325558	1	$k_{app,ml}$
ACOXT	5.120754	1	$k_{app,ml}$
ACOXT	5.120754	-1	$k_{app,ml}$
2HESR	3.911573	1	$k_{app,ml}$
CHOLID	3.274975	1	$k_{app,ml}$
NADHXD	2.317967	1	$k_{app,ml}$
4ABZGLUH	4.851053	1	$k_{app,ml}$
4ABZGLUH	2.580011	-1	$k_{app,ml}$
APH180	3.12568	1	$k_{app,ml}$
NADHXE	3.527344	1	$k_{app,ml}$
NADHXE	3.527344	-1	$k_{app,ml}$
PHDA	4.501681	1	$k_{app,ml}$
TEO2M	3.591815	1	$k_{app,ml}$
PACOAT	5.182569	1	$k_{app,ml}$
PYK2	1.483969	1	$k_{app,ml}$
PYK3	1.484309	1	$k_{app,ml}$
APH181	3.12568	1	$k_{app,ml}$
DHPTDNR	2.959104	1	$k_{app,ml}$
DHPTDNRN	2.301584	1	$k_{app,ml}$
PCNO	2.794064	1	$k_{app,ml}$
PCNO	2.719302	-1	$k_{app,ml}$
PYK6	1.484091	1	$k_{app,ml}$
HPACOAT	5.182569	1	$k_{app,ml}$
DC6PDA	8.290936	1	$k_{app,ml}$
5DGLCNR	33.42809	1	$k_{app,ml}$
PAI2I	1.941962	1	$k_{app,ml}$
CURR	2.671109	1	$k_{app,ml}$
LKDRA	3.122071	1	$k_{app,ml}$
LKDRA	2.912668	-1	$k_{app,ml}$
METNA	3.817243	1	$k_{app,ml}$
METNA	3.817243	-1	$k_{app,ml}$
5DKGR	32.2425	1	$k_{app,ml}$
5DKGR	2.874367	-1	$k_{app,ml}$
XYHDL	2.822777	1	$k_{app,ml}$
E4PP	3.4766	1	$k_{app,ml}$
PUACGAMS	2.664868	1	$k_{app,ml}$
PUACGAMS	3.220147	-1	$k_{app,ml}$
RU5PP	3.226787	1	$k_{app,ml}$
6D6SFK	3.439494	1	$k_{app,ml}$
3SLAR	2.702708	1	$k_{app,ml}$
MOX	1.488573	1	$k_{app,ml}$
ARHGDx	4.789896	1	$k_{app,ml}$
ARHGDx	4.750349	-1	$k_{app,ml}$
CPL	11.76008	1	$k_{app,ml}$
CYSTA	2.456687	1	$k_{app,ml}$
FE3DCITR5	4.112798	1	$k_{app,ml}$
4HTHRA	8.34468	1	$k_{app,ml}$
4HTHRA	1.505022	-1	$k_{app,ml}$
I2FE2SR	1.485261	1	$k_{app,ml}$
GAPP	12.69453	1	$k_{app,ml}$
INOSTO	2.437373	1	$k_{app,ml}$
INOSTO	2.866705	-1	$k_{app,ml}$
THZPSN3	0.037488	1	$k_{app,ml}$
HPYRP	3.857914	1	$k_{app,ml}$
PSURIK	3.356983	1	$k_{app,ml}$
PAI2T	1.575669	1	$k_{app,ml}$
APG3PAT120	4.890185	1	$k_{app,ml}$
QUINDHyi	4.070744	1	$k_{app,ml}$
DHCURR	2.671109	1	$k_{app,ml}$
RHMND	4.664837	1	$k_{app,ml}$
RHMND	4.664837	-1	$k_{app,ml}$

3HBZCT	3.283962	1	$k_{app,ml}$
CXSAMS	4.072984	1	$k_{app,ml}$
PNSPA	4.831707	1	$k_{app,ml}$
4HTHRK	1.512763	1	$k_{app,ml}$
AMPNTAT	3.693533	1	$k_{app,ml}$
APH120	3.12568	1	$k_{app,ml}$
SQGH	2.964042	1	$k_{app,ml}$
QUINDH	3.095731	1	$k_{app,ml}$
PETNT181pp	2.758737	1	$k_{app,ml}$
ARMEPNS	2.604305	1	$k_{app,ml}$
LCARSyi	16.67119	1	$k_{app,ml}$
MOCDS	1.491555	1	$k_{app,ml}$
RPNTPH	6.806072	1	$k_{app,ml}$
PRCPD	8.589033	1	$k_{app,ml}$
UDPGPT	2.703733	1	$k_{app,ml}$
UDPGPT	2.703733	-1	$k_{app,ml}$
OMMBLHXy	2.28037	1	$k_{app,ml}$
OPHHXy	2.585025	1	$k_{app,ml}$
PROD3	2.562292	1	$k_{app,ml}$
F1PP	1.483336	1	$k_{app,ml}$
ACP1p	2.77876	1	$k_{app,ml}$
3HPADHi	2.464206	1	$k_{app,ml}$
2HPTCOAT	6.153794	1	$k_{app,ml}$
MC6PH	6.1778	1	$k_{app,ml}$
FORCT	3.793628	1	$k_{app,ml}$
FORCT	3.793628	-1	$k_{app,ml}$
PACCOAE	2.314845	1	$k_{app,ml}$
OXDHCOAT	2.665219	1	$k_{app,ml}$
HADPCOADH3	2.508011	1	$k_{app,ml}$
HADPCOADH3	2.915597	-1	$k_{app,ml}$
DM_mththf_c	1.508031	1	$k_{app,ml}$
PYROX	2.79138	1	$k_{app,ml}$
BMOGDS1	3.699292	1	$k_{app,ml}$
MPTS	1.490435	1	$k_{app,ml}$
BMOGDS2	3.699292	1	$k_{app,ml}$
OCTNLL	1.483907	1	$k_{app,ml}$
XPPT	131	1	k_{cat}
HXPRT	7.89946	1	k_{cat}
SHK3Dr	31.35933	1	k_{cat}
SHK3Dr	11445.67	-1	k_{cat}
DHORTS	40.62444	1	k_{cat}
DURIPP	145	-1	k_{cat}
ACS	20.26667	1	k_{cat}
MAN6PI	64.4952	1	k_{cat}
PPA	108.0008	1	k_{cat}
TRPAS2	42	1	k_{cat}
PPCK	65	1	k_{cat}
ME1	134.4	1	k_{cat}
ALAR	27.6	1	k_{cat}
ALAR	6	-1	k_{cat}
ALATA_L	17.1	1	k_{cat}
XYLK	3427.5	1	k_{cat}
RBK	10.5	1	k_{cat}
PPM	173	-1	k_{cat}
ASPTA	82.47143	1	k_{cat}
FBP	19.1	1	k_{cat}
GLGC	370	1	k_{cat}
PYK	135.6429	1	k_{cat}
A5PISO	172	1	k_{cat}
A5PISO	130	-1	k_{cat}
GTHOr	441.95	1	k_{cat}
ILETA	48	1	k_{cat}
DHORD5	49.64	1	k_{cat}
VALTA	19	1	k_{cat}
ORPT	1.95	1	k_{cat}
TRPS2	4.45194	1	k_{cat}
PSCVT	1.94667	-1	k_{cat}
PFL	12.8	1	k_{cat}
ANS	64.03322	1	k_{cat}
FRD2	79.6	1	k_{cat}
FRD3	79.6	1	k_{cat}
POX	167	1	k_{cat}
CHORM	147457.6	1	k_{cat}
PTAr	1341.326	1	k_{cat}
PTAr	1419.466	-1	k_{cat}
ACKr	632.6667	1	k_{cat}

ACKr	942.75	-1	k_{cat}
LEUTAi	290	1	k_{cat}
TMDPP	984	1	k_{cat}
FBA	10.85	1	k_{cat}
HCO3E	17000	1	k_{cat}
PPS	1160.137	1	k_{cat}
PGI	1340.56	-1	k_{cat}
PGK	529.8	1	k_{cat}
EDA	72.15063	1	k_{cat}
NTPTP1	4000	1	k_{cat}
RPE	1180.013	1	k_{cat}
RPE	1300	-1	k_{cat}
TALA	5.67	-1	k_{cat}
PUNP1	38.785	1	k_{cat}
PUNP1	110	-1	k_{cat}
PUNP2	38.785	1	k_{cat}
PUNP2	110	-1	k_{cat}
PUNP3	15	1	k_{cat}
PUNP3	100.1667	-1	k_{cat}
TKT1	21.92635	1	k_{cat}
PUNP5	50.945	1	k_{cat}
PUNP5	111.6667	-1	k_{cat}
PUNP6	50.945	1	k_{cat}
PUNP6	111.6667	-1	k_{cat}
PUNP7	50.61333	1	k_{cat}
PUNP7	110	-1	k_{cat}
G6PDA	1800	1	k_{cat}
ALCD2x	24.37023	1	k_{cat}
ALCD2x	11.08	-1	k_{cat}
RNDR3	8.115	1	k_{cat}
ALDD2y	0.04	1	k_{cat}
RMI	68	1	k_{cat}
RMI	1717	-1	k_{cat}
TMDK1	114.104	1	k_{cat}
NADH10	0.077	1	k_{cat}
MDH	1035	1	k_{cat}
MDH	525	-1	k_{cat}
ABTA	3.34559	1	k_{cat}
FUM	290.0556	1	k_{cat}
FUM	40.10714	-1	k_{cat}
PYDXNK	0.3	1	k_{cat}
PYAM5PO	14.86	1	k_{cat}
SSALx	31.7	1	k_{cat}
ASPT	180	1	k_{cat}
SERD_L	490	1	k_{cat}
THRD	135	1	k_{cat}
NMNAT	0.00089	1	k_{cat}
KARA1	0.00417	1	k_{cat}
HMPK1	0.00012	1	k_{cat}
ACCOAC	65.25	1	k_{cat}
GLXCL	18.9	1	k_{cat}
GLCS1	284	1	k_{cat}
GLCP	50.5	1	k_{cat}
RBK_L1	40.42	1	k_{cat}
RBP4E	9.27384	1	k_{cat}
NTD2	368.25	1	k_{cat}
GLCRAL	27.3	1	k_{cat}
NTD4	368.25	1	k_{cat}
NTD5	294.632	1	k_{cat}
KAS14	3	1	k_{cat}
AGMT	3	1	k_{cat}
NTD6	294.66	1	k_{cat}
PYDXPP	10.25	1	k_{cat}
ARGDC	5.02	1	k_{cat}
ECOAH1	1280	-1	k_{cat}
G1SAT	0.59	-1	k_{cat}
UGLT	340.75	1	k_{cat}
UGLT	283	-1	k_{cat}
OAADC	2502.333	1	k_{cat}
MNNH	5.88	1	k_{cat}
DDGLK	26.68667	1	k_{cat}
XYLI2	16.91052	1	k_{cat}
XYLI2	11.085	-1	k_{cat}
PMANM	4.15	1	k_{cat}
GLUR	43.33	1	k_{cat}
UAG2E	3.625	1	k_{cat}

HCYSMT	0.0375	1	k_{cat}
MACPD	3	1	k_{cat}
UDPG4E	18	1	k_{cat}
UDPG4E	750	-1	k_{cat}
LACZ	247.5	1	k_{cat}
XYLI1	68.93588	1	k_{cat}
XYLI1	7.7	-1	k_{cat}
PPC	540	1	k_{cat}
ALDD4	26.95	1	k_{cat}
G3PT	160	1	k_{cat}
PYDXK	2.15	1	k_{cat}
HEX7	31.385	1	k_{cat}
URIC	24.7	1	k_{cat}
RMPA	38.3	1	k_{cat}
TGBPA	2.1	1	k_{cat}
DMSOR1	79.9	1	k_{cat}
PGLYCP	94	1	k_{cat}
G1PTT	5.925	1	k_{cat}
TDPGDH	3.4	1	k_{cat}
ADCS	0.53	1	k_{cat}
UDPGALM	1.5	1	k_{cat}
UMPK	107.399	1	k_{cat}
DDPGALA	0.94	1	k_{cat}
ALLTN	310.85	1	k_{cat}
UPPRT	3.85333	1	k_{cat}
NTD9	368.25	1	k_{cat}
NTD1	294.646	1	k_{cat}
NTD7	422.6667	1	k_{cat}
NDPK7	106	1	k_{cat}
TRPS3	2.09	1	k_{cat}
NTD10	368.25	1	k_{cat}
ADPT	9.33	1	k_{cat}
GTPDPK	2.4825	1	k_{cat}
ADK1	585.4	1	k_{cat}
ADK1	783	-1	k_{cat}
NDPK4	80.15	1	k_{cat}
ADA	18.5	1	k_{cat}
DADA	6.8	1	k_{cat}
ADD	5.5	1	k_{cat}
AGDC	95.875	1	k_{cat}
ASNN	800.0064	1	k_{cat}
ASPCT	417	1	k_{cat}
MCOATA	1580	1	k_{cat}
DAPE	75.5	-1	k_{cat}
GSPMDA	2.1	1	k_{cat}
GSPMDS	4.2	1	k_{cat}
GUAD	649	1	k_{cat}
TMAOR1	114	1	k_{cat}
DGK1	70.5	1	k_{cat}
NDPK1	38	1	k_{cat}
TRDR	16.955	1	k_{cat}
NNAM	0.93	1	k_{cat}
P5CD	19.15	1	k_{cat}
PGMT	92.5	1	k_{cat}
SPMDAT1	2.958	1	k_{cat}
KG6PDC	51	1	k_{cat}
SPMDAT2	2.958	1	k_{cat}
TRE6PP	14.3	1	k_{cat}
BETALDHy	276	1	k_{cat}
ADPRDP	117	1	k_{cat}
ACPS1	0.7	1	k_{cat}
ARAI	39.36357	1	k_{cat}
M1PD	8.5	1	k_{cat}
M1PD	125	-1	k_{cat}
G3PD2	291	1	k_{cat}
GALS3	118.25	1	k_{cat}
AMPN	61	1	k_{cat}
ACCOAL	79.57143	1	k_{cat}
CYANST	245	1	k_{cat}
GMAND	5	1	k_{cat}
CYTD	4.8	1	k_{cat}
DCTPD	1.24	1	k_{cat}
UAGAAT	9.16667	1	k_{cat}
CSND	125.4667	1	k_{cat}
GLUN	1270	1	k_{cat}
SERAT	115.217	-1	k_{cat}

CYSS	24	1	k_{cat}
PAPSR	3.5	1	k_{cat}
ASPK	29.26818	1	k_{cat}
ASAD	199.932	1	k_{cat}
OCBT	2300.433	1	k_{cat}
OCBT	90	-1	k_{cat}
GPDDA1	3.2	1	k_{cat}
GPDDA2	1.1	1	k_{cat}
ACBIPGT	0.058	1	k_{cat}
TRPS1	3.85906	1	k_{cat}
HPPPNDO	18.11	1	k_{cat}
GLYCK2	350	1	k_{cat}
ACOTA	1.55	1	k_{cat}
CYTDH	11.6	1	k_{cat}
ETHAAL	30	1	k_{cat}
AP4AH	250	1	k_{cat}
ADNCYC	1.67	1	k_{cat}
MI1PP	3.79667	1	k_{cat}
PPAKr	657.4	1	k_{cat}
PPAKr	618.5	-1	k_{cat}
TAUDO	2.34	1	k_{cat}
NTP3	422.9569	1	k_{cat}
PPM2	173	1	k_{cat}
ME2	66.6	1	k_{cat}
CBPS	3.15755	1	k_{cat}
ACOATA	0.30284	1	k_{cat}
ACOATA	0.30284	-1	k_{cat}
MLTG1	0.24	1	k_{cat}
AMALT3	94.275	1	k_{cat}
ACNML	7.7	1	k_{cat}
FTHFLi	3.6	1	k_{cat}
UDPGD	1.95425	1	k_{cat}
GLCRD	9.66583	1	k_{cat}
TDPAGTA	5.66	1	k_{cat}
GLUDC	4.74727	1	k_{cat}
HSDy	8.367	1	k_{cat}
CYSTL	1.5	1	k_{cat}
AMALT1	112.825	1	k_{cat}
MLTP2	4.1	1	k_{cat}
MLTP2	52	-1	k_{cat}
PACCOAL	30.85	1	k_{cat}
ACGAMT	1.2	1	k_{cat}
H2Otex	112	-1	k_{cat}
FACOAL160t2p	0.029	1	k_{cat}
FACOAL180t2p	0.029	1	k_{cat}
NO3R1bpp	392.5	1	k_{cat}
NO3R2pp	2.5	1	k_{cat}
NTP3pp	422.9569	1	k_{cat}
NO3R2bpp	392.5	1	k_{cat}
TMAOR1pp	258	1	k_{cat}
TRE6PS	5.2	1	k_{cat}
DRPA	294.5	1	k_{cat}
TKT2	20.43874	1	k_{cat}
UDCPDP	9	1	k_{cat}
TREH	199	1	k_{cat}
ATPS4rpp	270	1	k_{cat}
ATPS4rpp	217	-1	k_{cat}
CYTK2	41.66667	1	k_{cat}
O2tex	491	1	k_{cat}
NTD8pp	246.8733	1	k_{cat}
CSNt2pp	165	1	k_{cat}
TREHpp	199	1	k_{cat}
ACGAMK	0.03167	1	k_{cat}
METS	0.12	1	k_{cat}
ASPO5	5.55	1	k_{cat}
ASPO4	2.6	1	k_{cat}
FDMO4	0.86	1	k_{cat}
L_LACD3	178.7143	1	k_{cat}
L_LACD2	178.7143	1	k_{cat}
CYANSTpp	245	1	k_{cat}
MCPST	12.5	1	k_{cat}
UDCPDPpp	9	1	k_{cat}
NODOy	670	1	k_{cat}
GALM2pp	18400	1	k_{cat}
NODOx	670	1	k_{cat}
NHFRBO	14.9	1	k_{cat}

42A12BOOXpp	33.1032	1	k_{cat}
TYROXDApp	28.89711	1	k_{cat}
RNDR3b	2.5	1	k_{cat}
FEROpp	0.7125	1	k_{cat}
LACZpp	33.8	1	k_{cat}
GPDDA1pp	3.2	1	k_{cat}
GPDDA5pp	1.8	1	k_{cat}
FLVR	47.87692	1	k_{cat}
PSP_Lpp	26.8	1	k_{cat}
TYRPpp	62.4	1	k_{cat}
PAPSR2	3.5	1	k_{cat}
FACOAL80t2pp	0.029	1	k_{cat}
PPGPPDP	3	1	k_{cat}
GUACYC	28.7	1	k_{cat}
23PDE9pp	1.97	1	k_{cat}
SARCOX	37.43333	1	k_{cat}
FACOAL60t2pp	0.029	1	k_{cat}
ASNNpp	800.0064	1	k_{cat}
GLUNpp	1270	1	k_{cat}
NTD12	294.648	1	k_{cat}
NTD10pp	368.25	1	k_{cat}
NTD1pp	294.646	1	k_{cat}
NTD2pp	368.25	1	k_{cat}
NTD3pp	368.25	1	k_{cat}
NTD4pp	368.25	1	k_{cat}
NTD5pp	294.632	1	k_{cat}
NTD6pp	294.66	1	k_{cat}
NTD7pp	422.6667	1	k_{cat}
NTD9pp	368.25	1	k_{cat}
NH4tex	236	1	k_{cat}
PEAMNOpp	14.92857	1	k_{cat}
GAL1PPpp	0.0348	1	k_{cat}
NTPP9	0.16	1	k_{cat}
NTPP10	0.16	1	k_{cat}
ARGDCpp	5.02	1	k_{cat}
URIH	9.3	1	k_{cat}
INSH	0.043	1	k_{cat}
FACOAL100t2p	0.029	1	k_{cat}
GPDDA3pp	2.8	1	k_{cat}
GPDDA4pp	3.3	1	k_{cat}
GPDDA2pp	1.1	1	k_{cat}
FACOAL120t2p	0.029	1	k_{cat}
FACOAL140t2p	0.029	1	k_{cat}
HCYSMT2	0.38	1	k_{cat}
FALDH2	51.7	1	k_{cat}
CO2tex	380	1	k_{cat}
CBIAT	0.0775	1	k_{cat}
NTD12pp	294.648	1	k_{cat}
3NTD2pp	0.23	1	k_{cat}
3NTD9pp	8.04	1	k_{cat}
GLUDy	2136	1	k_{cat}
GLUDy	19466.67	-1	k_{cat}
23PDE7pp	7.63	1	k_{cat}
GLYCLTDy	7.48333	1	k_{cat}
LYSDC	405.5	1	k_{cat}
NTD3	368.25	1	k_{cat}
GTPDPDP	0.023	1	k_{cat}
DHFR	3.1513	1	k_{cat}
TYRTA	31.075	1	k_{cat}
TYRTA	64.16667	-1	k_{cat}
SHKK	35	1	k_{cat}
GLUSy	24.55	1	k_{cat}
GUAPRT	12.4865	1	k_{cat}
ABUTD	7.7	1	k_{cat}
E4PD	20	1	k_{cat}
DMSOR2pp	79.9	1	k_{cat}
DUTPDP	22.6625	1	k_{cat}
NO3R1pp	392.5	1	k_{cat}
NTP5	317.8481	1	k_{cat}
DPR	25	1	k_{cat}
NTD8	246.8733	1	k_{cat}
GALCTD	22	1	k_{cat}
PUNP4	50.61333	1	k_{cat}
PUNP4	100.1667	-1	k_{cat}
G6PP	700	1	k_{cat}
GLNS	6	1	k_{cat}

ACACT1r	15.8	1	k_{cat}
ACACT1r	159.5909	-1	k_{cat}
NTD11pp	368.25	1	k_{cat}
PDE1	63.4	1	k_{cat}
AMALT2	123.625	1	k_{cat}
MLTP1	5.4	1	k_{cat}
MLTP1	52	-1	k_{cat}
ALAALAr	15.13	1	k_{cat}
ADNK1	8.09733	1	k_{cat}
UDPGPpp	71	1	k_{cat}
PPPGO	3.35443	1	k_{cat}
LDH_D2	875	1	k_{cat}
SUCOAS	25.66667	1	k_{cat}
SUCOAS	21	-1	k_{cat}
CYSDDS	72	1	k_{cat}
NTP1	430.5998	1	k_{cat}
GPDDA4	3.3	1	k_{cat}
GALKr	15.66727	1	k_{cat}
G1PPpp	117	1	k_{cat}
LDH_D	320.5	-1	k_{cat}
AMALT4	84.05	1	k_{cat}
NTD11	368.25	1	k_{cat}
IPDDI	19.41364	1	k_{cat}
ORNDC	17.22986	1	k_{cat}
UPP3MT	38	1	k_{cat}
FACOAL181t2p	0.029	1	k_{cat}
EAR141y	130.2	1	k_{cat}
CFAS160G	0.39765	1	k_{cat}
FACOAL141t2p	0.029	1	k_{cat}
FACOAL161t2p	0.029	1	k_{cat}
SERD_D	13.1	1	k_{cat}
FLVRx	28.85	1	k_{cat}
FMNRx	6	1	k_{cat}
FORtex	272	-1	k_{cat}
PFK	83.6016	1	k_{cat}
PDX5POi	5.185	1	k_{cat}
PGM	255.2021	-1	k_{cat}
CYTBO3_4pp	814.4	1	k_{cat}
GLYCK	350	1	k_{cat}
GPDDA3	2.8	1	k_{cat}
TPI	9000	-1	k_{cat}
EAR181y	130.2	1	k_{cat}
DASYN140	8.92	1	k_{cat}
DASYN180	8.92	1	k_{cat}
DASYN141	8.92	1	k_{cat}
RPI	2100	1	k_{cat}
RPI	50	-1	k_{cat}
SFGTHi	17.505	1	k_{cat}
PYDAMK	0.55	1	k_{cat}
DHORDfum	7.8	1	k_{cat}
FACOAE120	50	1	k_{cat}
DSBCGT	25.2	1	k_{cat}
EAR180y	130.2	1	k_{cat}
ACONTb	44.8	1	k_{cat}
GLCP2	50.5	1	k_{cat}
SUCDi	167	1	k_{cat}
LALDO2x	266	1	k_{cat}
ASNS1	1.89833	1	k_{cat}
EAR80y	130.2	1	k_{cat}
EAR120y	130.2	1	k_{cat}
EAR121y	130.2	1	k_{cat}
EAR40y	130.2	1	k_{cat}
EAR161y	130.2	1	k_{cat}
3OAS140	3	1	k_{cat}
EAR160y	130.2	1	k_{cat}
PDE4	0.00096	1	k_{cat}
EAR100y	130.2	1	k_{cat}
ACONTa	5.3	1	k_{cat}
ACONTa	44.8	-1	k_{cat}
CFAS160E	0.39765	1	k_{cat}
CFAS180E	0.39765	1	k_{cat}
CFAS180G	0.39765	1	k_{cat}
DAGK140	2.8	1	k_{cat}
DAGK160	1.7	1	k_{cat}
3OAS60	3	1	k_{cat}
3OAS160	3	1	k_{cat}

EAR140y	130.2	1	k_{cat}
3OAS80	3	1	k_{cat}
3OAS100	3	1	k_{cat}
3OAS120	3	1	k_{cat}
LSERDHr	4.2	1	k_{cat}
DASYN120	8.92	1	k_{cat}
EAR60y	130.2	1	k_{cat}
DAGK181	2.8	1	k_{cat}
NTP11	0.22	1	k_{cat}
3OAR180	71	1	k_{cat}
3OAS180	3	1	k_{cat}
ACOAD6f	6	1	k_{cat}
NADH16pp	1517	1	k_{cat}
NADH18pp	1517	1	k_{cat}
NADH17pp	1517	1	k_{cat}
DTARTD	7.3	1	k_{cat}
OXCDC	88	1	k_{cat}
CTECOAI8	47	1	k_{cat}
CTECOAI8	12	-1	k_{cat}
CTECOAI6	63	1	k_{cat}
SELR	387	1	k_{cat}
FLDR2	273.5	1	k_{cat}
THFAT	18.4	1	k_{cat}
ALDD19xr	96.8	1	k_{cat}
MMM	48	1	k_{cat}
GTHPi	221.7	1	k_{cat}
ACOAD1f	9.6	1	k_{cat}
26DAHtex	65	1	transporter
26DAHtex	65	-1	transporter
HCINNMtex	65	1	transporter
HCINNMtex	65	-1	transporter
HPPPNtex	65	1	transporter
HPPPNtex	65	-1	transporter
ACtex	65	1	transporter
ACtex	65	-1	transporter
ACALDtex	65	1	transporter
ACALDtex	65	-1	transporter
ACMANAtex	65	1	transporter
ACMANAtex	65	-1	transporter
ACMUMtex	65	1	transporter
SUCCt2_2pp	65	1	transporter
SUCFUMtpp	65	1	transporter
SUCFUMtpp	65	-1	transporter
SUCptspp	65	1	transporter
SULabcpp	65	1	transporter
TSULabcpp	65	1	transporter
TARTRt7pp	65	1	transporter
TARTRt7pp	65	-1	transporter
TAURabcpp	65	1	transporter
THMDt2pp_co	65	1	transporter
NMNt7pp	65	1	transporter
GLUtex	65	1	transporter
GLUtex	65	-1	transporter
Kt2pp	65	1	transporter
GLYtex	65	1	transporter
GLYtex	65	-1	transporter
GLYALDtex	65	1	transporter
GLYALDtex	65	-1	transporter
AKGt2rpp	65	1	transporter
AKGt2rpp	65	-1	transporter
XYLt2pp	65	1	transporter
GLYBtex	65	1	transporter
GLYBtex	65	-1	transporter
GLYCtex	65	1	transporter
GLYCtex	65	-1	transporter
GLYC3Ptex	65	1	transporter
GLYC3Ptex	65	-1	transporter
GLYCLTtex	65	1	transporter
GLYCLTtex	65	-1	transporter
GTPtex	65	1	transporter
GTPtex	65	-1	transporter
GUAtex	65	1	transporter
GUAtex	65	-1	transporter
Htex	65	1	transporter
Htex	65	-1	transporter
H2tex	65	1	transporter
H2tex	65	-1	transporter
H2Otex	65	1	transporter
O2tpp	65	1	transporter
O2tpp	65	-1	transporter
PYRt2rpp	65	1	transporter
PYRt2rpp	65	-1	transporter
THMabcpp	65	1	transporter
THMDt2pp_co	65	1	transporter
THMDt2pp_co	65	-1	transporter
THRabcpp	65	1	transporter

TREptspp	65	1	transporter
XANtpp	65	1	transporter
XANtpp	65	-1	transporter
DAPtex	65	1	transporter
DAPtex	65	-1	transporter
TRPt2rpp	65	1	transporter
TRPt2rpp	65	-1	transporter
TYRt2rpp	65	1	transporter
TYRt2rpp	65	-1	transporter
URIt2pp_copy	65	1	transporter
URIt2pp_copy	65	1	transporter
URIt2pp_copy	65	-1	transporter
VALabcpp	65	1	transporter
12PPDStex	65	1	transporter
12PPDStex	65	-1	transporter
DDGLCNtex	65	1	transporter
DDGLCNtex	65	-1	transporter
XTSNt2rpp	65	1	transporter
XTSNt2rpp	65	-1	transporter
XYLabcpp	65	1	transporter
XYLUt2pp	65	1	transporter
THRt4pp	65	1	transporter
GALURtex	65	1	transporter
GALURtex	65	-1	transporter
FEOXAMabcpp	65	1	transporter
GALtex	65	1	transporter
GALtex	65	-1	transporter
CBL1abcpp	65	1	transporter
ACACt2pp	65	1	transporter
ACACt2pp	65	-1	transporter
ACGAptspp	65	1	transporter
ACMANAptspp	65	1	transporter
ACNAMt2pp	65	1	transporter
GALCTtex	65	1	transporter
GALCTtex	65	-1	transporter
GALCTNtex	65	1	transporter
GALCTNtex	65	-1	transporter
GALTtex	65	1	transporter
GALTtex	65	-1	transporter
FEENTERabcpp	65	1	transporter
FECRMabcpp	65	1	transporter
GAMtex	65	1	transporter
GAMtex	65	-1	transporter
GBBTNtex	65	1	transporter
GBBTNtex	65	-1	transporter
INDOLEtex	65	1	transporter
INDOLEtex	65	-1	transporter
ARBt2rpp	65	1	transporter
ARBt2rpp	65	-1	transporter
DAPabcpp	65	1	transporter
L_LACtex	65	1	transporter
L_LACtex	65	-1	transporter
ACt4pp	65	1	transporter
ADEt2rpp	65	1	transporter
ADEt2rpp	65	-1	transporter
CO2tpp	65	1	transporter
CO2tpp	65	-1	transporter
DHAtpp	65	1	transporter
DHAtpp	65	-1	transporter
ARGabcpp	65	1	transporter
ADNt2pp_copy	65	1	transporter
ADNt2pp_copy	65	1	transporter
ADNt2pp_copy	65	-1	transporter
ALAabcpp	65	1	transporter
ALLabcpp	65	1	transporter
ALLTNt2rpp	65	1	transporter
ALLTNt2rpp	65	-1	transporter
ARBabcpp	65	1	transporter
ARGAGMt7pp	65	1	transporter
ARGAGMt7pp	65	-1	transporter
ASCBptspp	65	1	transporter
ASNt2rpp	65	1	transporter
ASNt2rpp	65	-1	transporter
ASPabcpp	65	1	transporter
ASPt2pp	65	1	transporter
GUAtpp	65	1	transporter
GUAtpp	65	-1	transporter
ASPt2_2pp	65	1	transporter
HYXNtpp	65	1	transporter
HYXNtpp	65	-1	transporter
NACtpp	65	1	transporter
ASPt2_3pp	65	1	transporter
HIStex	65	1	transporter
HIStex	65	-1	transporter
FE3DCITabcpp	65	1	transporter
HXAtex	65	1	transporter
HXAtex	65	-1	transporter
HYXNtex	65	1	transporter
HYXNtex	65	-1	transporter
IDONtex	65	1	transporter
IDONtex	65	-1	transporter

ILEtex	65	1	transporter
ILEtex	65	-1	transporter
INSTtex	65	1	transporter
INSTtex	65	-1	transporter
INStex	65	1	transporter
INStex	65	-1	transporter
Ktex	65	1	transporter
Ktex	65	-1	transporter
CPGNtonex	65	1	transporter
GLCRtex	65	1	transporter
GLCRtex	65	-1	transporter
GLCNtex	65	1	transporter
GLCNtex	65	-1	transporter
GLCURtex	65	1	transporter
GLCURtex	65	-1	transporter
GLNtex	65	1	transporter
GLNtex	65	-1	transporter
O2tex	65	-1	transporter
OCTAtex	65	1	transporter
OCTAtex	65	-1	transporter
D_LACtex	65	1	transporter
D_LACtex	65	-1	transporter
ORNtex	65	1	transporter
ORNtex	65	-1	transporter
FRULYStex	65	1	transporter
FRULYStex	65	-1	transporter
MALTtexi	65	1	transporter
CITt7pp	65	1	transporter
CRNt7pp	65	1	transporter
GLCtex_copy1	65	1	transporter
CHLabcpp	65	1	transporter
DGSNt2pp	65	1	transporter
FE3DCITtonex	65	1	transporter
DINSt2pp	65	1	transporter
DURIt2pp	65	1	transporter
FE2abcpp	65	1	transporter
FRUptspp	65	1	transporter
FUCtpp	65	1	transporter
FUCtpp	65	-1	transporter
GALCTt2rpp	65	1	transporter
GALCTt2rpp	65	-1	transporter
GALt2pp	65	1	transporter
ASPtex	65	1	transporter
ASPtex	65	-1	transporter
ABUTt2pp	65	1	transporter
FUMt2_2pp	65	1	transporter
FUMt2_3pp	65	1	transporter
PHEtex	65	1	transporter
PHEtex	65	-1	transporter
FEENTERtonex	65	1	transporter
CYNTt2pp	65	1	transporter
CYSabcpp	65	1	transporter
SBTptspp	65	1	transporter
PItex	65	1	transporter
PItex	65	-1	transporter
CYTDt2pp_cop	65	1	transporter
CYTDt2pp_cop	65	1	transporter
CYTDt2pp_cop	65	-1	transporter
DADNt2pp	65	1	transporter
DCYTt2pp	65	1	transporter
ACALDtpp	65	1	transporter
ACALDtpp	65	-1	transporter
ACt2rpp	65	1	transporter
ACt2rpp	65	-1	transporter
DDGLCNt2rpp	65	1	transporter
DDGLCNt2rpp	65	-1	transporter
RMNtex	65	1	transporter
RMNtex	65	-1	transporter
MALTTRtexi	65	1	transporter
MALTTTRtexi	65	1	transporter
MALTPTtexi	65	1	transporter
LCTStex	65	1	transporter
LCTStex	65	-1	transporter
LEUtex	65	1	transporter
LEUtex	65	-1	transporter
LYStex	65	1	transporter
LYStex	65	-1	transporter
LYXtex	65	1	transporter
LYXtex	65	-1	transporter
MANtex	65	1	transporter
MANtex	65	-1	transporter
MAN6Ptex	65	1	transporter
MAN6Ptex	65	-1	transporter
CYSabc2pp	65	1	transporter
BUTSO3abcpp	65	1	transporter
ETHSO3abcpp	65	1	transporter
MELIBtex	65	1	transporter
MELIBtex	65	-1	transporter
DMSOtex	65	1	transporter
DMSOtex	65	-1	transporter
DURItex	65	1	transporter

DURItex	65	-1	transporter
FRUtex	65	1	transporter
FRUtex	65	-1	transporter
FUCtex	65	1	transporter
FUCtex	65	-1	transporter
FUMtex	65	1	transporter
FUMtex	65	-1	transporter
FE2tex	65	1	transporter
FE2tex	65	-1	transporter
NMNtex	65	1	transporter
NMNtex	65	-1	transporter
NO2tex	65	1	transporter
NO2tex	65	-1	transporter
FEOXAMUtex	65	1	transporter
ARBTNtpp	65	1	transporter
ARBTNtex	65	1	transporter
BUTt2rpp	65	1	transporter
BUTt2rpp	65	-1	transporter
CADVtpp	65	1	transporter
23CAMPtex	65	1	transporter
23CAMPtex	65	-1	transporter
23CCMPtex	65	1	transporter
23CCMPtex	65	-1	transporter
23CUMPtex	65	1	transporter
23CUMPtex	65	-1	transporter
23CGMPtex	65	1	transporter
23CGMPtex	65	-1	transporter
3AMPtex	65	1	transporter
3AMPtex	65	-1	transporter
3CMPtex	65	1	transporter
3CMPtex	65	-1	transporter
GDPtex	65	1	transporter
GDPtex	65	-1	transporter
FEENTERtpp	65	1	transporter
TMAtex	65	1	transporter
TMAtex	65	-1	transporter
TMAOtex	65	1	transporter
TMAOtex	65	-1	transporter
TREtex	65	1	transporter
TREtex	65	-1	transporter
FEENTERtex	65	1	transporter
FE3HOXUtpp	65	1	transporter
FEOXAMUtpp	65	1	transporter
FECRMUtpp	65	1	transporter
TRPtex	65	1	transporter
TRPtex	65	-1	transporter
PTRCt2pp	65	1	transporter
PNTOtex	65	1	transporter
PNTOtex	65	-1	transporter
TSULtex	65	1	transporter
TSULtex	65	-1	transporter
TYRtex	65	1	transporter
TYRtex	65	-1	transporter
URAtex	65	1	transporter
URAtex	65	-1	transporter
UREAtex	65	1	transporter
UREAtex	65	-1	transporter
URItex	65	1	transporter
URItex	65	-1	transporter
SULFACabcpp	65	1	transporter
ETHSO3tex	65	1	transporter
ETHSO3tex	65	-1	transporter
BUTSO3tex	65	1	transporter
BUTSO3tex	65	-1	transporter
LIPOtex	65	1	transporter
LIPOtex	65	-1	transporter
VALtex	65	1	transporter
VALtex	65	-1	transporter
XANtex	65	1	transporter
XANtex	65	-1	transporter
XYLtex	65	1	transporter
XYLtex	65	-1	transporter
PPPNtex	65	1	transporter
PPPNtex	65	-1	transporter
ABUTtex	65	1	transporter
ABUTtex	65	-1	transporter
FORtppi	65	1	transporter
PHEMEabcpp	65	1	transporter
METSOX1abcp	65	1	transporter
PPTtex	65	1	transporter
PPTtex	65	-1	transporter
GALBDtex	65	1	transporter
GALBDtex	65	-1	transporter
PTRCtex	65	1	transporter
PTRCtex	65	-1	transporter
PYRtex	65	1	transporter
PYRtex	65	-1	transporter
CU1abcpp	65	1	transporter
HG2abcpp	65	1	transporter
SULFACtex	65	1	transporter
SULFACtex	65	-1	transporter

DSERtex	65	1	transporter
DSERtex	65	-1	transporter
SERtex	65	1	transporter
SERtex	65	-1	transporter
NOtex	65	1	transporter
NOtex	65	-1	transporter
NOtpp	65	1	transporter
NOtpp	65	-1	transporter
N2Otpp	65	1	transporter
N2Otpp	65	-1	transporter
N2Otex	65	1	transporter
N2Otex	65	-1	transporter
CRNt8pp	65	1	transporter
CRNt2rpp	65	1	transporter
CRNt2rpp	65	-1	transporter
CRNDt2rpp	65	1	transporter
CRNDt2rpp	65	-1	transporter
CTBTt2rpp	65	1	transporter
CTBTt2rpp	65	-1	transporter
PACALDt2rpp	65	1	transporter
PACALDt2rpp	65	-1	transporter
PEAMNtex	65	1	transporter
PEAMNtex	65	-1	transporter
PACALDtex	65	1	transporter
PACALDtex	65	-1	transporter
LIPOt2pp	65	1	transporter
ACGAL1Ptex	65	1	transporter
ACGAL1Ptex	65	-1	transporter
ACGALtex	65	1	transporter
ACGALtex	65	-1	transporter
GLCUR1Ptex	65	1	transporter
GLCUR1Ptex	65	-1	transporter
UDPGLCURtex	65	1	transporter
UDPGLCURtex	65	-1	transporter
ISETACabcpp	65	1	transporter
ISETACtex	65	1	transporter
ISETACtex	65	-1	transporter
UDPGtex	65	1	transporter
UDPGtex	65	-1	transporter
UDPGALtex	65	1	transporter
UDPGALtex	65	-1	transporter
ACGAM1Ptex	65	1	transporter
ACGAM1Ptex	65	-1	transporter
UACGAMtex	65	1	transporter
UACGAMtex	65	-1	transporter
UDPACGALtex	65	1	transporter
UDPACGALtex	65	-1	transporter
INOSTt4pp	65	1	transporter
PHEMEtiex	65	1	transporter
CYANtex	65	1	transporter
CYANtex	65	-1	transporter
SO3tex	65	1	transporter
SO3tex	65	-1	transporter
TCYNTtex	65	1	transporter
TCYNTtex	65	-1	transporter
METSOX2abcp	65	1	transporter
METSOX1tex	65	1	transporter
METSOX1tex	65	-1	transporter
METSOX2tex	65	1	transporter
METSOX2tex	65	-1	transporter
3UMPtex	65	1	transporter
3UMPtex	65	-1	transporter
MSO3abcpp	65	1	transporter
MSO3tex	65	1	transporter
MSO3tex	65	-1	transporter
3GMPtex	65	1	transporter
3GMPtex	65	-1	transporter
FALDtpp	65	1	transporter
FALDtpp	65	-1	transporter
SO4tex	65	1	transporter
SO4tex	65	-1	transporter
DOPAtex	65	1	transporter
DOPAtex	65	-1	transporter
4HOXPACDtex	65	1	transporter
4HOXPACDtex	65	-1	transporter
SPMDtex	65	1	transporter
SPMDtex	65	-1	transporter
ETHAtex	65	1	transporter
ETHAtex	65	-1	transporter
G3PStex	65	1	transporter
G3PStex	65	-1	transporter
SUCCtex	65	1	transporter
SUCCtex	65	-1	transporter
SUCRtex	65	1	transporter
SUCRtex	65	-1	transporter
CD2t3pp	65	1	transporter
ZNabcpp	65	1	transporter
SKMt2pp	65	1	transporter
FE2t2pp	65	1	transporter
GLYC2Ptex	65	1	transporter
GLYC2Ptex	65	-1	transporter

PSERtex	65	1	transporter
PSERtex	65	-1	transporter
TARTRtex	65	1	transporter
TARTRtex	65	-1	transporter
R5Ptex	65	1	transporter
R5Ptex	65	-1	transporter
THRPtex	65	1	transporter
THRPtex	65	-1	transporter
TYRPtex	65	1	transporter
TYRPtex	65	-1	transporter
Kabcpp	65	1	transporter
TAURtex	65	1	transporter
TAURtex	65	-1	transporter
LEUabcpp	65	1	transporter
L_LACt2rpp	65	1	transporter
L_LACt2rpp	65	-1	transporter
MALt2_2pp	65	1	transporter
THRtex	65	1	transporter
THRtex	65	-1	transporter
CPGNUtpp	65	1	transporter
CPGNUtex	65	1	transporter
MALt2_3pp	65	1	transporter
MALTabcpp	65	1	transporter
MALTHXabcpp	65	1	transporter
MALTPTabcpp	65	1	transporter
MALTTRabcpp	65	1	transporter
THMDtex	65	1	transporter
THMDtex	65	-1	transporter
MALTTTRabcp	65	1	transporter
MAN6Pt6_2pp	65	1	transporter
CPGNabcpp	65	1	transporter
MANGLYCptsp	65	1	transporter
MELIBt2pp	65	1	transporter
CBIuabcpp	65	1	transporter
METabcpp	65	1	transporter
CITtex	65	1	transporter
CITtex	65	-1	transporter
ACGAtex	65	1	transporter
ACGAtex	65	-1	transporter
DDCAtexi	65	1	transporter
TTDCAtexi	65	1	transporter
HDCAtexi	65	1	transporter
RMNtpp	65	1	transporter
SERt4pp	65	1	transporter
METDtex	65	1	transporter
METDtex	65	-1	transporter
SUCCt2_3pp	65	1	transporter
MNLptspp	65	1	transporter
NAt3_1p5pp	65	1	transporter
NAt3_2pp	65	1	transporter
NH4tpp	65	-1	transporter
METtex	65	1	transporter
METtex	65	-1	transporter
NMNPtpp	65	1	transporter
NO2t2rpp	65	1	transporter
NO2t2rpp	65	-1	transporter
NO3t7pp	65	1	transporter
ORNabcpp	65	1	transporter
PIuabcpp	65	1	transporter
PNTOt4pp	65	1	transporter
METDabcpp	65	1	transporter
IDONt2rpp	65	1	transporter
IDONt2rpp	65	-1	transporter
ILEabcpp	65	1	transporter
INDOLEt2pp	65	1	transporter
INSt2pp	65	1	transporter
PROabcpp	65	1	transporter
PROt2rpp	65	1	transporter
PROt2rpp	65	-1	transporter
MNLtex	65	1	transporter
MNLtex	65	-1	transporter
PROt4pp	65	1	transporter
PTRCabcpp	65	1	transporter
LYXt2pp	65	1	transporter
OCDCAtexi	65	1	transporter
OCDCEAtexi	65	1	transporter
G6Ptex	65	1	transporter
G6Ptex	65	-1	transporter
TUNGSabcpp	65	1	transporter
NAtex	65	1	transporter
NAtex	65	-1	transporter
MOBDabcpp	65	1	transporter
MOBDtex	65	1	transporter
MOBDtex	65	-1	transporter
NI2uabcpp	65	1	transporter
FECRMtonex	65	1	transporter
ARBTNtonex	65	1	transporter
ADOCBLabcpp	65	1	transporter
NI2tex	65	1	transporter
NI2tex	65	-1	transporter
NACtex	65	1	transporter

NACtex	65	-1	transporter
CRNabcpp	65	1	transporter
CRNDabcpp	65	1	transporter
CTBTabcpp	65	1	transporter
NH4tex	65	-1	transporter
H2O2tex	65	1	transporter
H2O2tex	65	-1	transporter
ADEtex	65	1	transporter
ADEtex	65	-1	transporter
ADNtex	65	1	transporter
ADNtex	65	-1	transporter
ASO3tex	65	1	transporter
ASO3tex	65	-1	transporter
AGMtex	65	1	transporter
AGMtex	65	-1	transporter
ALAtex	65	1	transporter
ALAtex	65	-1	transporter
BALAtex	65	1	transporter
BALAtex	65	-1	transporter
DALAtex	65	1	transporter
DALAtex	65	-1	transporter
MANGLYCtex	65	1	transporter
MANGLYCtex	65	-1	transporter
AMPtex	65	1	transporter
AMPtex	65	-1	transporter
FRUURt2rpp	65	1	transporter
FRUURt2rpp	65	-1	transporter
FRUURtex	65	1	transporter
FRUURtex	65	-1	transporter
ARBtex	65	1	transporter
ARBtex	65	-1	transporter
ARGtex	65	1	transporter
ARGtex	65	-1	transporter
ASCBtex	65	1	transporter
ASCBtex	65	-1	transporter
RIBabcpp	65	1	transporter
GLYCAt2rpp	65	1	transporter
GLYCAt2rpp	65	-1	transporter
GLYCAtex	65	1	transporter
GLYCAtex	65	-1	transporter
BUTtex	65	1	transporter
BUTtex	65	-1	transporter
FE3HOXtonex	65	1	transporter
CU2abcpp	65	1	transporter
COBALT2abcp	65	1	transporter
NI2abcpp	65	1	transporter
MG2uabcpp	65	1	transporter
CHLtex	65	1	transporter
CHLtex	65	-1	transporter
GLYBabcpp	65	1	transporter
GLYC3Pt6pp	65	1	transporter
MMETtex	65	1	transporter
MMETtex	65	-1	transporter
FE3tex	65	1	transporter
FE3tex	65	-1	transporter
MG2tex	65	1	transporter
MG2tex	65	-1	transporter
Zn2tex	65	1	transporter
Zn2tex	65	-1	transporter
MMETt2pp	65	1	transporter
CD2tex	65	1	transporter
CD2tex	65	-1	transporter
GLYCtpp	65	1	transporter
GLYCtpp	65	-1	transporter
H2Otpp	65	1	transporter
H2Otpp	65	-1	transporter
GLNabcpp	65	1	transporter
CO2tex	65	-1	transporter
GLUabcpp	65	1	transporter
GLUABUTt7pp	65	1	transporter
GLUABUTt7pp	65	-1	transporter
GLUt2rpp	65	1	transporter
GLUt2rpp	65	-1	transporter
HCINNMt2rpp	65	1	transporter
HCINNMt2rpp	65	-1	transporter
HISabcpp	65	1	transporter
HISt2rpp	65	1	transporter
HISt2rpp	65	-1	transporter
GLUt4pp	65	1	transporter
GLYALDtpp	65	1	transporter
GLYALDtpp	65	-1	transporter
CRNtex	65	1	transporter
CRNtex	65	-1	transporter
LYSabcpp	65	1	transporter
COBALT2tex	65	1	transporter
COBALT2tex	65	-1	transporter
LCTSt3ipp	65	1	transporter
ARBt3ipp	65	1	transporter
14GLUCANabc	65	1	transporter
14GLUCANtex	65	1	transporter
DINStex	65	1	transporter

DINStex	65	-1	transporter
DIMPtex	65	1	transporter
DIMPtex	65	-1	transporter
DMStex	65	1	transporter
DMStex	65	-1	transporter
GALTptspp	65	1	transporter
GALURt2rpp	65	1	transporter
GALURt2rpp	65	-1	transporter
ZN2abcpp	65	1	transporter
CSNtex	65	1	transporter
CSNtex	65	-1	transporter
GLCNt2rpp	65	1	transporter
GLCNt2rpp	65	-1	transporter
GLCptspp	65	1	transporter
GLCRt2rpp	65	1	transporter
GLCRt2rpp	65	-1	transporter
UREAtpp	65	1	transporter
UREAtpp	65	-1	transporter
XANt2pp	65	1	transporter
GLYC3Pabcpp	65	1	transporter
PPPNt2rpp	65	1	transporter
PPPNt2rpp	65	-1	transporter
PTRCORNt7pp	65	1	transporter
PTRCORNt7pp	65	-1	transporter
GLCURt2rpp	65	1	transporter
GLCURt2rpp	65	-1	transporter
ACMUMptspp	65	1	transporter
SPMDabcpp	65	1	transporter
CYNTtex	65	1	transporter
CYNTtex	65	-1	transporter
HG2tex	65	1	transporter
HG2tex	65	-1	transporter
XTSNtex	65	1	transporter
XTSNtex	65	-1	transporter
CYStex	65	1	transporter
CYStex	65	-1	transporter
CYTDtex	65	1	transporter
CYTDtex	65	-1	transporter
DADNtex	65	1	transporter
DADNtex	65	-1	transporter
4PEPTtex	65	1	transporter
4PEPTtex	65	-1	transporter
DHAtex	65	1	transporter
DHAtex	65	-1	transporter
ETOHtex	65	1	transporter
ETOHtex	65	-1	transporter
GALabcpp	65	1	transporter
GLYCLTt2rpp	65	1	transporter
GLYCLTt2rpp	65	-1	transporter
GUAt2pp	65	1	transporter
HPPPNt2rpp	65	1	transporter
HPPPNt2rpp	65	-1	transporter
DCAtex	65	1	transporter
DCAtex	65	-1	transporter
DCYTtex	65	1	transporter
DCYTtex	65	-1	transporter
ACACtex	65	1	transporter
ACACtex	65	-1	transporter
ACNAMtex	65	1	transporter
ACNAMtex	65	-1	transporter
ARBTNabcpp	65	1	transporter
DGSNtex	65	1	transporter
DGSNtex	65	-1	transporter
ASO3t8pp	65	1	transporter
H2Stex	65	1	transporter
H2Stex	65	-1	transporter
H2St1pp	65	1	transporter
CELBpts	65	1	transporter
FORt2pp	65	1	transporter
MANptspp	65	1	transporter
TYMtex	65	1	transporter
TYMtex	65	-1	transporter
ARGORNt7pp	65	1	transporter
ARGORNt7pp	65	-1	transporter
CMPtex	65	1	transporter
CMPtex	65	-1	transporter
DUMPtex	65	1	transporter
DUMPtex	65	-1	transporter
XMPtex	65	1	transporter
XMPtex	65	-1	transporter
3PEPTtex	65	1	transporter
3PEPTtex	65	-1	transporter
ANHGMtex	65	1	transporter
ANHGMtex	65	-1	transporter
MALTptspp	65	1	transporter
FALDtex	65	1	transporter
FALDtex	65	-1	transporter
CU2tex	65	1	transporter
CU2tex	65	-1	transporter
FECRMUtex	65	1	transporter
CLtex	65	1	transporter

CLtex	65	-1	transporter
CYSDtex	65	1	transporter
CYSDtex	65	-1	transporter
CYSDabcpp	65	1	transporter
GMPtex	65	1	transporter
GMPtex	65	-1	transporter
IMPtex	65	1	transporter
IMPtex	65	-1	transporter
G3PItex	65	1	transporter
G3PItex	65	-1	transporter
CD2abcpp	65	1	transporter
G3PGtex	65	1	transporter
G3PGtex	65	-1	transporter
G3PEtex	65	1	transporter
G3PEtex	65	-1	transporter
G3PCtex	65	1	transporter
G3PCtex	65	-1	transporter
UMPtex	65	1	transporter
UMPtex	65	-1	transporter
G1Ptex	65	1	transporter
G1Ptex	65	-1	transporter
CUt3	65	1	transporter
ZN2t3pp	65	1	transporter
SKMtex	65	1	transporter
SKMtex	65	-1	transporter
MNt2pp	65	1	transporter
GLCabcpp	65	1	transporter
MNtex	65	1	transporter
MNtex	65	-1	transporter
GAL1Ptex	65	1	transporter
GAL1Ptex	65	-1	transporter
O2Stex	65	1	transporter
O2Stex	65	-1	transporter
Kt3pp	65	1	transporter
HOMt2pp	65	1	transporter
HOMtex	65	1	transporter
HOMtex	65	-1	transporter
SO2tex	65	1	transporter
SO2tex	65	-1	transporter
SO2tpp	65	1	transporter
SO2tpp	65	-1	transporter
CYStpp	65	1	transporter
DAMPtex	65	1	transporter
DAMPtex	65	-1	transporter
DCMPtex	65	1	transporter
DCMPtex	65	-1	transporter
DGMPtex	65	1	transporter
DGMPtex	65	-1	transporter
DTMPtex	65	1	transporter
DTMPtex	65	-1	transporter
ACSERtpp	65	1	transporter
ACSERtex	65	1	transporter
ACSERtex	65	-1	transporter
2AGPE120tipp	65	1	transporter
G3PGabcpp	65	1	transporter
G3PIabcpp	65	1	transporter
G3PSabcpp	65	1	transporter
2AGPA120tipp	65	1	transporter
2AGPG180tipp	65	1	transporter
ALAALAtex	65	1	transporter
ALAALAtex	65	-1	transporter
2AGPG141tipp	65	1	transporter
2AGPG161tipp	65	1	transporter
2AGPG181tipp	65	1	transporter
PGP120abcpp	65	1	transporter
MALtex	65	1	transporter
MALtex	65	-1	transporter
NO3tex	65	1	transporter
NO3tex	65	-1	transporter
PGP161abcpp	65	1	transporter
PGP181abcpp	65	1	transporter
PA120abcpp	65	1	transporter
URAt2pp	65	1	transporter
TTDCEAtexi	65	1	transporter
HDCEAtexi	65	1	transporter
PA160abcpp	65	1	transporter
ALAALAabcpp	65	1	transporter
HEXt2rpp	65	1	transporter
HEXt2rpp	65	-1	transporter
G3PEabcpp	65	1	transporter
G3PCabcpp	65	1	transporter
PA181abcpp	65	1	transporter
12DGR141tipp	65	1	transporter
PROGLYabcpp	65	1	transporter
GTHRDabcpp	65	1	transporter
2AGPG140tipp	65	1	transporter
2AGPG160tipp	65	1	transporter
D_LACt2pp	65	1	transporter
D_LACt2pp	65	-1	transporter
RIBtex	65	1	transporter
RIBtex	65	-1	transporter

THMtex	65	1	transporter
THMtex	65	-1	transporter
XYLUtex	65	1	transporter
XYLUtex	65	-1	transporter
ALLtex	65	1	transporter
ALLtex	65	-1	transporter
FRUpts2pp	65	1	transporter
PGP140abcpp	65	1	transporter
34dhpactex	65	1	transporter
34dhpactex	65	-1	transporter
G6Pt6_2pp	65	1	transporter
MALTHXtexi	65	1	transporter
12PPDStpp	65	1	transporter
12PPDStpp	65	-1	transporter
PROtex	65	1	transporter
PROtex	65	-1	transporter
FE3HOXUtex	65	1	transporter
FE3DHBZSton	65	1	transporter
H2tpp	65	1	transporter
H2tpp	65	-1	transporter
23DAPPAtex	65	1	transporter
23DAPPAtex	65	-1	transporter
FEOXAMtonex	65	1	transporter
GAMptspp	65	1	transporter
GSNt2pp	65	1	transporter
GSNtex	65	1	transporter
GSNtex	65	-1	transporter
SBTtex	65	1	transporter
SBTtex	65	-1	transporter
FORtex	65	1	transporter
PHEt2rpp	65	1	transporter
PHEt2rpp	65	-1	transporter
HG2t3pp	65	1	transporter
MELIBt3ipp	65	1	transporter
GLCtex_copy2	65	1	transporter
GLCtex_copy2	65	-1	transporter
NAt3pp	65	1	transporter
MINOHPtexi	65	1	transporter
12PPDRtpp	65	1	transporter
12PPDRtpp	65	-1	transporter
K2L4Aabcpp	65	1	transporter
AKGtex	65	1	transporter
AKGtex	65	-1	transporter
ALLTNtex	65	1	transporter
ALLTNtex	65	-1	transporter
ASNtex	65	1	transporter
ASNtex	65	-1	transporter
F6Pt6_2pp	65	1	transporter
PPALtpp	65	1	transporter
PPALtpp	65	-1	transporter
GAM6Pt6_2pp	65	1	transporter
FE3HOXabcpp	65	1	transporter
PPALtex	65	1	transporter
PPALtex	65	-1	transporter
CA2tex	65	1	transporter
CA2tex	65	-1	transporter
CUtex	65	1	transporter
CUtex	65	-1	transporter
12PPDRtex	65	1	transporter
12PPDRtex	65	-1	transporter
PROGLYtex	65	1	transporter
PROGLYtex	65	-1	transporter
F6Ptex	65	1	transporter
F6Ptex	65	-1	transporter
GAMAN6Ptex	65	1	transporter
GAMAN6Ptex	65	-1	transporter
PE141abcpp	65	1	transporter
PG140abcpp	65	1	transporter
PG141abcpp	65	1	transporter
12DGR140tipp	65	1	transporter
PG180abcpp	65	1	transporter
12DGR160tipp	65	1	transporter
PGP141abcpp	65	1	transporter
PGP160abcpp	65	1	transporter
12DGR180tipp	65	1	transporter
PGP180abcpp	65	1	transporter
PA140abcpp	65	1	transporter
12DGR120tipp	65	1	transporter
PG120abcpp	65	1	transporter
PE140abcpp	65	1	transporter
2AGPE140tipp	65	1	transporter
2AGPE160tipp	65	1	transporter
2AGPE180tipp	65	1	transporter
2AGPE141tipp	65	1	transporter
2AGPE161tipp	65	1	transporter
2AGPA161tipp	65	1	transporter
2AGPA181tipp	65	1	transporter
2AGPG120tipp	65	1	transporter
PA141abcpp	65	1	transporter
PA161abcpp	65	1	transporter
PA180abcpp	65	1	transporter

PE120abcpp	65	1	transporter
PE180abcpp	65	1	transporter
CGLYtex	65	1	transporter
CGLYtex	65	-1	transporter
GTHOXtex	65	1	transporter
GTHOXtex	65	-1	transporter
TDSR1	65	1	transporter
CGLYabcpp	65	1	transporter
2AGPE181tipp	65	1	transporter
2AGPA140tipp	65	1	transporter
2AGPA160tipp	65	1	transporter
2AGPA180tipp	65	1	transporter
2AGPA141tipp	65	1	transporter
12DGR161tipp	65	1	transporter
12DGR181tipp	65	1	transporter
TDSR2	65	1	transporter
GTHRDtex	65	1	transporter
GTHRDtex	65	-1	transporter
GTHRDabc2pp	65	1	transporter
NI2tpp	65	1	transporter
COBALT2t3pp	65	1	transporter
23DAPPAt2pp	65	1	transporter
CAt6pp	65	1	transporter
GLYBt2pp	65	1	transporter
5DGLCNt2rpp	65	1	transporter
5DGLCNt2rpp	65	-1	transporter
GALCTNt2pp	65	1	transporter
GALCTNLtex	65	1	transporter
GALCTNLtex	65	-1	transporter
DSERt2pp	65	1	transporter
ALAt4pp	65	1	transporter
LYSt3pp	65	1	transporter
CA2t3pp	65	1	transporter
THRt2pp	65	1	transporter
ARGt3pp	65	1	transporter
MALDtex	65	1	transporter
MALDtex	65	-1	transporter
OROTtex	65	1	transporter
OROTtex	65	-1	transporter
PPAt4pp	65	1	transporter
OROTt2_2pp	65	1	transporter
MALDt2_2pp	65	1	transporter
5DGLCNtex	65	1	transporter
5DGLCNtex	65	-1	transporter
CHLt2pp	65	1	transporter
GLYt4pp	65	1	transporter
FRULYSt2pp	65	1	transporter
TUNGStex	65	1	transporter
TUNGStex	65	-1	transporter
PSCLYSt2pp	65	1	transporter
LYSt2pp	65	1	transporter
COBALT2tpp	65	1	transporter
GLYt2pp	65	1	transporter
ALAt2pp	65	1	transporter
BALAt2pp	65	1	transporter
MG2tpp	65	1	transporter
5MTRtex	65	1	transporter
5MTRtex	65	-1	transporter
NI2t3pp	65	1	transporter
PPAtex	65	1	transporter
PPAtex	65	-1	transporter
GALCTNLt2pp	65	1	transporter
THYMt3pp	65	1	transporter
CLt3_2pp	65	1	transporter
PSCLYStex	65	1	transporter
PSCLYStex	65	-1	transporter
THYMtex	65	1	transporter
THYMtex	65	-1	transporter
FE2t3pp	65	1	transporter
MN2t3pp	65	1	transporter
MG2t3_2pp	65	1	transporter
MG2t3_2pp	65	-1	transporter
CD2tpp	65	1	transporter
ETHAt2pp	65	1	transporter
FE3abcpp	65	1	transporter
LALADGLUtex	65	1	transporter
LALADGLUtex	65	-1	transporter
LALALGLUtpp	65	1	transporter
3HPPtex	65	1	transporter
3HPPtex	65	-1	transporter
CMtpp	65	1	transporter
FUSAtpp	65	1	transporter
RFAMPtex	65	1	transporter
RFAMPtex	65	-1	transporter
MINCYCtex	65	1	transporter
MINCYCtex	65	-1	transporter
MINCYCtpp	65	1	transporter
MEOHtrpp	65	1	transporter
MEOHtrpp	65	-1	transporter
DOXRBCNtex	65	1	transporter
DOXRBCNtex	65	-1	transporter

DOXRBCNtpp	65	1	transporter
SELtex	65	1	transporter
SELtex	65	-1	transporter
ETOHtrpp	65	1	transporter
ETOHtrpp	65	-1	transporter
MEOHtex	65	1	transporter
MEOHtex	65	-1	transporter
5MTRtpp	65	1	transporter
TTRCYCtex	65	1	transporter
TTRCYCtex	65	-1	transporter
NOVBCNtpp	65	1	transporter
CMtex	65	1	transporter
CMtex	65	-1	transporter
TTRCYCtpp	65	1	transporter
LALALGLUtex	65	1	transporter
LALALGLUtex	65	-1	transporter
LALADGLUtpp	65	1	transporter
SLNTtex	65	1	transporter
SLNTtex	65	-1	transporter
3HPPtpp	65	1	transporter
RFAMPtpp	65	1	transporter
ALAtpp	65	1	transporter
CH4tex	65	1	transporter
CH4tex	65	-1	transporter
TARTRDtex	65	1	transporter
TARTRDtex	65	-1	transporter
BTNt2ipp	65	1	transporter
BTNtex	65	1	transporter
BTNtex	65	-1	transporter
DHBSZ3FEabcp	65	1	transporter
DXYLONtpp	65	1	transporter
DXYLONtpp	65	-1	transporter
2DGLCptspp	65	1	transporter
ARBTptspp	65	1	transporter
ARBTtex	65	1	transporter
DMSOtpp	65	1	transporter
DMSOtpp	65	-1	transporter
PYDXtex	65	1	transporter
PYDXtex	65	-1	transporter
PYDXtpp	65	1	transporter
SUCASPtpp	65	1	transporter
SUCASPtpp	65	-1	transporter
23DOGULNt4p	65	1	transporter
SUCMALtpp	65	1	transporter
SUCMALtpp	65	-1	transporter
PYDXNtex	65	1	transporter
PYDXNtex	65	-1	transporter
PYDXNtpp	65	1	transporter
DXYLONtex	65	1	transporter
DXYLONtex	65	-1	transporter
PYDAMtex	65	1	transporter
PYDAMtex	65	-1	transporter
PYDAMtpp	65	1	transporter
PSURItex	65	1	transporter
PSURItex	65	-1	transporter
CHLt3pp	65	1	transporter
TARTt2_3pp	65	1	transporter
SUCTARTtpp	65	1	transporter
SUCTARTtpp	65	-1	transporter
CHTBSptspp	65	1	transporter
GLYBt3pp	65	1	transporter
AI2tpp	65	1	transporter
Ftpp	65	1	transporter
CHTBStex	65	1	transporter
CHTBStex	65	-1	transporter
TRPtipp	65	1	transporter
TYRtipp	65	1	transporter
DHPStr	65	1	transporter
SQtr	65	1	transporter
DHPStex	65	1	transporter
DHPStex	65	-1	transporter
SPMDt3pp	65	1	transporter
FMNtex	65	1	transporter
FMNtex	65	-1	transporter
PHEtipp	65	1	transporter
MALt3pp	65	1	transporter
CITt3pp	65	1	transporter
QUIN2tpp	65	1	transporter
QUIN2tpp	65	-1	transporter
GLYC2Pabcpp	65	1	transporter
CRNDtex	65	1	transporter
CRNDtex	65	-1	transporter
CLBtex	65	1	transporter
CLBtex	65	-1	transporter
CHOLATEtpp	65	1	transporter
4ABZGLUtr	65	1	transporter
4ABZGLUtr	65	-1	transporter
SELabcpp	65	1	transporter
CYSItpp	65	1	transporter
FADt	65	1	transporter
PUACGAMtr	65	1	transporter

PIt2rpp	65	1	transporter
FADtex	65	1	transporter
FADtex	65	-1	transporter
AI2abcpp	65	1	transporter
4HBZt3pp	65	1	transporter
LEUt2rpp	65	1	transporter
LCTStpp	65	1	transporter
ILEt3pp	65	1	transporter
METt3pp	65	1	transporter
GSNt3pp	65	1	transporter
CLtipp	65	1	transporter
2DGLCtex	65	1	transporter
2DGLCtex	65	-1	transporter
PItpp	65	1	transporter
LEUt3pp	65	1	transporter
INSt3pp	65	1	transporter
INDOLEt2rpp	65	1	transporter
SQtex	65	1	transporter
SQtex	65	-1	transporter
ILEt2rpp	65	1	transporter
GLYtpp	65	1	transporter
GLYtpp	65	-1	transporter
URATEtex	65	1	transporter
URATEtex	65	-1	transporter
CBL1tonex	65	1	transporter
CBItonex	65	1	transporter
PSURItpp	65	1	transporter
CH4t2pp	65	1	transporter
METGLCURt2p	65	1	transporter
METGLCURtex	65	1	transporter
METGLCURtex	65	-1	transporter
FMNt	65	1	transporter
AI2tex	65	1	transporter
AI2tex	65	-1	transporter
CUt2pp	65	1	transporter
Ftex	65	1	transporter
Ftex	65	-1	transporter
CYSItex	65	1	transporter
CYSItex	65	-1	transporter
SERtpp	65	1	transporter
THRt2rpp	65	1	transporter
SERt2rpp	65	1	transporter
URATEtpp	65	1	transporter
CS1tex	65	1	transporter
CS1tex	65	-1	transporter
ADOCBLtonex	65	1	transporter
URAtpp	65	1	transporter
ASPtpp	65	1	transporter
ASPtpp	65	-1	transporter
FUMt1pp	65	1	transporter
FUMt1pp	65	-1	transporter
SUCCt1pp	65	1	transporter
SUCCt1pp	65	-1	transporter
FUSAtex	65	1	transporter
FUSAtex	65	-1	transporter
MEPNtex	65	1	transporter
MEPNtex	65	-1	transporter
MEPNabcpp	65	1	transporter
NOVBCNtex	65	1	transporter
NOVBCNtex	65	-1	transporter
4ABZGLUtex	65	1	transporter
4ABZGLUtex	65	-1	transporter
QUIN2tex	65	1	transporter
QUIN2tex	65	-1	transporter
SLNTabcpp	65	1	transporter
VALt2rpp	65	1	transporter
VALt3pp	65	1	transporter
MN2tipp	65	1	transporter

Table S3: Experimental and predicted protein expression levels

Predicted protein expression (mass fraction in proteome)

gene locus	Chemostat glucose-limited (μ=0.12 per h)	Chemostat glucose-limited (μ=0.2 per h)	Galactose (μ=0.26 per h)	Acetate (μ=0.3 per h)	Chemostat glucose-limited (μ=0.35 per h)	Pyruvate (μ=0.4 per h)	Fumarate (μ=0.42 per h)	Succinate (μ=0.44 per h)	Glucosamine (μ=0.46 per h)	Glycerol (μ=0.47 per h)	Mannose (μ=0.47 per h)	Chemostat glucose-limited (μ=0.5 per h)	Osmotic-stress glucose (μ=0.55 per h)	Xylose (μ=0.55 per h)	Glucose (μ=0.58 per h)	pH6 glucose (μ=0.63 per h)	Fructose (μ=0.65 per h)	42°C glucose (μ=0.66 per h)
b0002	0	0	0	0	0	0	0	0	0	0	0	0	0	0	0	0	0	0
b0003	8.02E-06	1.26E-05	1.60E-05	1.69E-05	2.11E-05	2.39E-05	2.51E-05	2.63E-05	2.74E-05	2.75E-05	2.79E-05	2.97E-05	3.26E-05	3.25E-05	3.43E-05	3.71E-05	3.82E-05	3.89E-05
b0004	5.76E-05	9.03E-05	0.000114566	0.000121208	0.000151551	0.000170949	0.000179927	0.000188339	0.000196262	0.000196961	0.000200184	0.000212823	0.000233249	0.000232548	0.000245502	0.000265924	0.000273717	0.000278176
b0007	2.28E-07	3.61E-07	4.62E-07	0	6.18E-07	7.01E-07	7.40E-07	7.76E-07	8.11E-07	0	8.28E-07	8.84E-07	9.74E-07	0	1.03E-06	0	1.16E-06	1.18E-06
b0008	0	0	0	4.56E-05	0	0.000460022	6.77E-05	7.09E-05	0	0	0	0	0	8.90E-05	0	0	0	0
b0009	0	0	0	0	0	0	0	0	0	0	0	0	0	0	0	0	0	0
b0010	0.000204067	0.000326323	0.000427482	0.001876471	0.000567975	0.002554645	0.001090899	0.000790513	0.00071333	0	0.000733677	0.000825673	0.000915132	0.000935409	0.000969642	0.001061891	0.001041994	0.001118109
b0019	0	0	0	0	0	0	0	0	0	0	0	0	0	0	0	0	0	0
b0025	9.88E-06	1.57E-05	2.00E-05	2.13E-05	2.68E-05	3.04E-05	3.21E-05	3.37E-05	3.52E-05	3.54E-05	3.59E-05	3.83E-05	4.23E-05	4.21E-05	4.47E-05	4.87E-05	5.02E-05	5.11E-05
b0029	5.15E-06	8.16E-06	1.04E-05	1.11E-05	1.40E-05	1.59E-05	1.67E-05	1.76E-05	1.83E-05	1.84E-05	1.87E-05	2.00E-05	2.20E-05	2.20E-05	2.33E-05	2.54E-05	2.62E-05	2.66E-05
b0030	0	0	0	0	0	2.55E-33	0	0	0	0	0	0	0	0	0	0	0	0
b0031	7.81E-05	0.000122296	0.000155124	0.00016405	0.000205011	0.000231127	0.000243212	0.000254525	0.000265173	0.000266087	0.000270441	0.000287417	0.000314815	0.00031387	0.000331234	0.000358563	0.000368979	0.000374942
b0032	0	0	0	0	0	0	0	0	0	0	0	0	0	0	0	0	0	0
b0033	0	0	0	0	0	0	0	0	0	0	0	0	0	0	0	0	0	0
b0036	0	0	0	0	0	0	0	0	0	0	0	0	0	0	0	0	0	0
b0037	0	0	0	0	0	0	0	0	0	0	0	0	0	0	0	0	0	0
b0038	0	0	0	0	0	0	0	0	0	0	0	0	0	0	0	0	0	0
b0040	0	0	0	0	0	0	0	0	0	0	0	0	0	0	0	0	0	0
b0046	0	0	0	0	0	0	0	0	0	0	0	0	0	0	0	0	0	0
b0047	0	0	0	0	0	0	0	0	0	0	0	0	0	0	0	0	0	0
b0048	8.44E-06	1.34E-05	1.71E-05	1.82E-05	2.29E-05	2.60E-05	2.74E-05	2.88E-05	3.00E-05	3.02E-05	3.07E-05	3.27E-05	3.61E-05	3.60E-05	3.81E-05	4.16E-05	4.29E-05	4.36E-05
b0049	0	0	0	0	0	0	0	0	0	0	0	0	0	0	0	0	0	0
b0052	4.71E-06	7.46E-06	9.55E-06	1.02E-05	1.28E-05	1.45E-05	1.53E-05	1.61E-05	1.68E-05	1.69E-05	1.71E-05	1.83E-05	2.02E-05	2.01E-05	2.13E-05	2.32E-05	2.39E-05	2.44E-05
b0054	6.49E-06	1.01E-05	1.27E-05	1.34E-05	1.66E-05	1.86E-05	1.95E-05	2.04E-05	2.12E-05	2.12E-05	2.16E-05	2.28E-05	2.48E-05	2.48E-05	2.60E-05	2.80E-05	2.87E-05	2.91E-05
b0061	0	0	0	0	0	0	0	0	0	0	0	0	0	0	0	0	0	0
b0062	0	0	0	0	0	0	0	0	0	0	0	0	0	0	0	0	0	0
b0063	0	0	0	0	0	0	0	0	0	0	0	0	0	0	0	0	0	0
b0066	0	0	0	0	0	0	0	0	0	0	0	0	0	0	0	0	0	0
b0067	0	0	0	0	0	0	0	0	0	0	0	0	0	0	0	0	0	0
b0068	0	0	0	0	0	0	0	0	0	0	0	0	0	0	0	0	0	0
b0070	0	0	0	0	0	0	0	0	0	0	0	0	0	0	0	0	0	0
b0071	0.000155664	0.000244025	0.000309713	0.000327668	0.000409697	0.000462136	0.000486408	0.000509148	0.000530568	0.000532457	0.00054117	0.000575339	0.000630556	0.000628662	0.000663681	0.000718889	0.000739955	0.000752011
b0072	0.000345295	0.000541299	0.000687007	0.000726837	0.000908793	0.001025114	0.001078954	0.001129397	0.001176911	0.0011811	0.001200427	0.001276221	0.001398705	0.001394504	0.001472182	0.001594646	0.001641374	0.001668116
b0073	0.000118768	0.000186186	0.000236304	0.000250003	0.000312589	0.000352599	0.000371118	0.000388468	0.000404811	0.000406252	0.0004129	0.00043897	0.0004811	0.000479655	0.000506373	0.000548496	0.000564569	0.000573767
b0074	0.000192951	0.000302478	0.0003839	0.000406157	0.000507834	0.000572834	0.00060292	0.000631107	0.000657658	0.000659999	0.000670799	0.000713153	0.000781597	0.000779249	0.000822656	0.000891089	0.000917201	0.000932144
b0077	0	0	0	0	0	0	0	0	0	0	0	0	0	0	0	0	0	0
b0078	0	0	0	0	0	0	0	0	0	0	0	0	0	0	0	0	0	0
b0084	3.52E-05	5.47E-05	6.89E-05	7.25E-05	9.01E-05	0.000100955	0.000105962	0.000110603	0.000114926	0.000115168	0.000117053	0.000123896	0.000134766	0.000134361	0.000141186	0.000151706	0.000155643	0.000157918
b0085	2.53E-05	3.93E-05	4.96E-05	5.22E-05	6.48E-05	7.26E-05	7.62E-05	7.96E-05	8.27E-05	8.29E-05	8.42E-05	8.91E-05	9.70E-05	9.67E-05	0.000101576	0.000109145	0.000111977	0.000113614
b0086	1.08E-05	1.68E-05	2.11E-05	2.22E-05	2.76E-05	3.09E-05	3.25E-05	3.39E-05	3.52E-05	3.53E-05	3.59E-05	3.80E-05	4.13E-05	4.12E-05	4.33E-05	4.65E-05	4.77E-05	4.84E-05
b0087	5.05E-06	7.85E-06	9.89E-06	1.04E-05	1.29E-05	1.45E-05	1.52E-05	1.59E-05	1.65E-05	1.65E-05	1.68E-05	1.78E-05	1.93E-05	1.93E-05	2.03E-05	2.18E-05	2.23E-05	2.27E-05
b0088	2.05E-05	3.19E-05	4.01E-05	4.23E-05	5.25E-05	5.88E-05	6.17E-05	6.44E-05	6.70E-05	6.71E-05	6.82E-05	7.22E-05	7.85E-05	7.83E-05	8.23E-05	8.84E-05	9.07E-05	9.20E-05
b0089	2.41E-05	3.75E-05	4.73E-05	4.97E-05	6.18E-05	6.92E-05	7.27E-05	7.58E-05	7.88E-05	7.90E-05	8.03E-05	8.50E-05	9.24E-05	9.21E-05	9.68E-05	0.000104035	0.000106735	0.000108295
b0090	1.21E-05	1.89E-05	2.38E-05	2.50E-05	3.11E-05	3.48E-05	3.66E-05	3.82E-05	3.96E-05	3.97E-05	4.04E-05	4.27E-05	4.65E-05	4.64E-05	4.87E-05	5.23E-05	5.37E-05	5.45E-05

Experimental protein expression (mass fraction in proteome)

gene locus	Chemostat glucose-limited (μ=0.12 per h)	Chemostat glucose-limited (μ=0.2 per h)	Galactose (μ=0.26 per h)	Acetate (μ=0.3 per h)	Chemostat glucose-limited (μ=0.35 per h)	Pyruvate (μ=0.4 per h)	Fumarate (μ=0.42 per h)	Succinate (μ=0.44 per h)	Glucosamine (μ=0.46 per h)	Glycerol (μ=0.47 per h)	Mannose (μ=0.47 per h)	Chemostat glucose-limited (μ=0.5 per h)	Osmotic-stress glucose (μ=0.55 per h)	Xylose (μ=0.55 per h)	Glucose (μ=0.58 per h)	pH6 glucose (μ=0.63 per h)	Fructose (μ=0.65 per h)	42°C glucose (μ=0.66 per h)
b0002	0.001330153	0.001696786	0.001621815	0.001587718	0.00235766	0.007039137	0.002329197	0.002558154	0.003420385	0.00306842	0.002884373	0.005094867	0.00398188	0.005786607	0.003467609	0.003117212	0.00563769	0.002615613
b0003	5.19E-05	5.51E-05	9.00E-05	0.000108021	5.66E-05	0.000219379	0.000116419	0.000101271	0.000170586	0.000109359	5.73E-05	7.90E-05	0.000356044	0.00027495	0.000136231	0.000185734	0.000157073	0.000127418
b0004	0.001436201	0.001501431	0.001933138	0.002229307	0.001938167	0.00717573	0.003189494	0.003585301	0.003124749	0.003850336	0.003176074	0.0029748	0.005746759	0.00558062	0.004694725	0.002976603	0.004483625	0.002284368
b0007	0	0	0	0	0	0	0	0	0	0	0	0	0	0	0	0	0	0
b0008	0.001354387	0.001599488	0.002386251	0.001999765	0.001977179	0.003065044	0.002604548	0.002546542	0.002546971	0.002926467	0.00236803	0.002527816	0.003328298	0.003283536	0.002553143	0.003079019	0.002890375	0.004449264
b0009	4.42E-06	4.84E-06	2.48E-06	8.63E-06	4.62E-06	7.97E-06	1.10E-05	2.87E-06	9.96E-06	6.81E-06	3.54E-06	5.16E-06	1.50E-06	1.07E-06	6.83E-06	1.80E-06	2.60E-06	1.81E-06
b0010	0	0	0	0	0	0	0	0	0	0	0	0	0	0	0	0	0	0
b0019	0	0	0	0	0	0	0	0	0	0	0	0	0	0	0	0	0	0
b0025	3.12E-05	4.12E-05	3.49E-05	2.92E-05	3.18E-05	3.80E-05	3.46E-05	3.03E-05	3.74E-05	3.94E-05	2.88E-05	4.51E-05	2.55E-05	3.62E-05	3.79E-05	3.46E-05	3.87E-05	4.03E-05
b0029	2.18E-05	2.74E-05	2.81E-05	3.49E-05	3.46E-05	4.85E-05	4.15E-05	3.52E-05	3.44E-05	5.29E-05	3.69E-05	3.59E-05	2.52E-05	3.72E-05	5.32E-05	3.22E-05	4.33E-05	3.38E-05
b0030	9.39E-05	9.93E-05	3.92E-05	5.81E-05	0.000100306	6.43E-05	9.10E-05	6.32E-05	3.68E-05	5.08E-05	7.55E-05	6.84E-05	1.44E-05	1.41E-05	9.40E-06	6.11E-06	1.83E-05	1.09E-05
b0031	0.000249544	0.000261383	0.000269561	0.000393316	0.000252662	0.00036888	0.000268524	0.000241184	0.000349621	0.000370753	0.000328464	0.000303579	0.00053349	0.000408169	0.00060435	0.000750229	0.000430406	0.000762236
b0032	0.000632985	0.000716529	0.001253781	0.001036416	0.00089725	0.001283555	0.001566841	0.001725536	0.001429966	0.001774227	0.001282428	0.001448268	0.001683734	0.001728718	0.002615714	0.001980656	0.002044887	0.002298085
b0033	0.001341653	0.001546277	0.001732042	0.001507946	0.001782574	0.001688998	0.002250628	0.00231119	0.001932498	0.002473369	0.00221369	0.002546162	0.00184518	0.0022101	0.003573554	0.002269328	0.002857322	0.00275546
b0036	3.08E-06	9.30E-06	2.28E-06	2.81E-06	8.13E-06	2.10E-06	3.46E-06	4.68E-06	2.22E-06	2.43E-06	0	5.71E-06	1.95E-07	0	1.14E-05	3.49E-06	0	1.39E-06
b0037	0	0	0	0	0	0	0	0	0	0	0	0	0	0	0	0	0	0
b0038	0	0	0	0	0	0	0	0	0	0	0	0	0	0	0	0	0	0
b0040	0	0	0	0	0	0	0	0	0	0	0	0	0	0	0	0	0	0
b0046	1.23E-06	2.69E-06	2.72E-06	3.65E-06	2.27E-06	1.67E-06	9.44E-07	4.81E-06	3.82E-06	4.92E-06	0	3.09E-06	1.90E-06	0	6.24E-06	1.01E-06	0	1.30E-06
b0047	7.12E-06	5.87E-06	1.44E-06	2.00E-06	5.59E-06	1.38E-06	2.37E-06	2.24E-06	3.87E-06	1.96E-06	2.02E-06	5.15E-06	5.72E-07	6.00E-07	1.03E-06	1.50E-06	1.49E-06	1.23E-06
b0048	3.12E-05	2.59E-05	2.75E-05	2.69E-05	2.59E-05	3.96E-05	4.33E-05	3.12E-05	3.23E-05	3.93E-05	3.07E-05	2.52E-05	2.79E-05	2.74E-05	3.62E-05	2.96E-05	3.12E-05	2.70E-05
b0049	1.30E-05	1.21E-05	9.87E-06	1.33E-05	1.00E-05	1.10E-05	1.08E-05	9.40E-06	9.46E-06	9.40E-06	9.29E-06	8.48E-06	1.37E-05	9.15E-06	1.22E-05	1.24E-05	6.36E-06	1.22E-05
b0052	2.88E-05	3.17E-05	3.98E-05	4.21E-05	3.02E-05	4.75E-05	4.10E-05	3.75E-05	4.56E-05	4.29E-05	3.45E-05	2.90E-05	6.50E-05	4.67E-05	4.41E-05	5.51E-05	3.66E-05	4.22E-05
b0054	0.000281323	0.000283027	0.000293053	0.000223609	0.000261613	0.000247536	0.000241513	0.000227914	0.000343253	0.000239751	0.000228396	0.000241425	0.000364115	0.00027003	0.000320233	0.000360365	0.000209414	0.000313058
b0061	0	0	0	0	0	0	0	0	0	0	0	0	0	0	0	0	0	0
b0062	0	0	0	0	0	0	0	0	0	0	0	0	0	0	0	0	0	0
b0063	0	0	0	0	0	0	0	0	0	0	0	0	0	0	0	0	0	0
b0066	0	0	0	0	0	0	0	0	0	0	0	0	0	0	0	0	0	0
b0067	0	0	0	0	0	0	0	0	0	0	0	0	0	0	0	0	0	0
b0068	2.08E-06	1.99E-06	3.69E-06	5.82E-06	2.30E-06	9.19E-06	4.89E-06	7.37E-06	5.86E-06	7.21E-06	5.51E-06	4.85E-06	3.12E-06	4.37E-06	4.56E-06	6.04E-07	5.34E-06	3.00E-08
b0070	0	0	0	0	0	0	0	0	0	0	0	0	0	0	0	0	0	0
b0071	0.00154297	0.001138126	0.001030572	0.001263052	0.001015545	0.000636608	0.000838447	0.001064388	0.001019784	0.000858533	0.000905837	0.000653093	0.000603259	0.000885755	0.000784819	0.000250811	0.000811079	0.000373487
b0072	0.001836117	0.001434768	0.000997454	0.001496609	0.001247966	0.000816616	0.001119736	0.001051642	0.001322104	0.001071418	0.000762512	0.000785266	0.000559913	0.000794068	0.000940284	0.000366712	0.000843002	0.000390466
b0073	0.002780745	0.002315349	0.002663336	0.002418549	0.001973756	0.00091491	0.001344713	0.00130862	0.001242657	0.001462607	0.001247526	0.001552059	0.003362103	0.001906781	0.001236605	0.001224931	0.001302454	0.001614974
b0074	0.002938219	0.002022082	0.002148367	0.002054025	0.001771217	0.000930152	0.001276026	0.001494856	0.001336872	0.001425227	0.001408565	0.001526623	0.002517452	0.002101748	0.00105973	0.000837238	0.001432046	0.001763378
b0077	0.00018207	0.000210593	0.000153828	0.000163579	0.000210292	0.000225742	0.000165049	0.00014452	0.000188402	0.000218568	0.000168575	0.000205047	0.000161714	0.00019706	0.000189348	0.000176694	0.00018323	0.000165903
b0078	0.000130911	0.000138152	0.000202853	0.000136872	0.000124088	0.00018606	0.000131444	0.000189356	0.000191827	0.000164739	0.000210833	0.000139398	0.000175417	0.000228506	0.000187843	0.000189681	0.000227614	0.000167654
b0084	3.00E-06	5.52E-06	5.00E-06	8.02E-06	5.17E-06	1.24E-05	5.91E-06	7.05E-06	8.72E-06	6.45E-06	9.60E-06	7.35E-06	3.29E-06	7.38E-06	5.00E-06	5.17E-06	5.92E-06	6.73E-06
b0085	0.000110128	0.000110028	0.000141259	0.000229444	0.000119557	0.000207939	0.000161118	0.000140939	0.000134906	0.000147397	0.000155956	0.000129545	0.000153235	0.000171822	0.000186279	0.000206454	0.000152118	0.000207979
b0086	9.23E-05	0.000102402	9.90E-05	0.000204014	0.000104104	0.000182473	0.00017645	9.38E-05	0.000117359	0.000165663	0.000117775	0.000142502	0.000146986	0.000141037	0.000171692	0.000174878	9.67E-05	0.000161807
b0087	7.87E-06	8.20E-06	5.16E-06	6.20E-06	8.14E-06	1.03E-05	7.22E-06	3.10E-06	6.78E-06	7.20E-06	3.29E-06	9.82E-06	5.11E-06	6.74E-06	5.55E-06	7.94E-06	5.59E-06	1.03E-05
b0088	1.22E-05	1.24E-05	1.96E-05	2.73E-05	1.31E-05	3.00E-05	2.48E-05	2.14E-05	3.32E-05	2.38E-05	2.10E-05	1.76E-05	1.57E-05	1.90E-05	2.60E-05	5.67E-05	2.26E-05	1.58E-05
b0089	0	0	0	0	0	0	0	0	0	0	0	0	0	0	0	0	0	0
b0090	2.01E-05	1.67E-05	2.34E-05	2.36E-05	1.89E-05	3.20E-05	1.75E-05	2.77E-05	2.12E-05	2.53E-05	2.19E-05	2.41E-05	1.85E-05	2.83E-05	2.37E-05	2.48E-05	2.88E-05	4.24E-05

b0651	0	0	0	0	0	0	0	0	0	0	0	0	0	0	0	0	0	0	5.04E-05	5.09E-05	4.26E-05	6.19E-05	6.23E-05	5.89E-05	7.93E-05	7.86E-05	7.21E-05	6.67E-05	6.84E-05	7.98E-05	2.29E-05	2.95E-05	3.31E-05	1.80E-05	5.32E-05	1.90E-05
b0652	0	0	0	0	0	0	0	0	0	0	0	0	0	0	0	0	0	0	2.26E-05	2.40E-05	1.79E-05	1.10E-05	2.17E-05	2.12E-05	2.19E-05	2.10E-05	1.98E-05	1.94E-05	2.27E-05	1.79E-05	1.57E-05	1.74E-05	1.50E-05	2.03E-05	1.99E-05	1.34E-05
b0653	0	0	0	0	0	0	0	0	0	0	0	0	0	0	0	0	0	0	0	0	0	0	0	0	0	0	0	0	0	0	0	0	0	0	0	0
b0654	0	0	0	0	0	0	0	0	0	0	0	0	0	0	0	0	0	0	0	0	0	0	0	0	0	0	0	0	0	0	0	0	0	0	0	0
b0655	0	0	0	0	0	0	0	0	0	0	0	0	0	0	0	0	0	0	0.001436238	0.001489343	0.00154888	0.001292015	0.001312104	0.001486491	0.001787019	0.001301402	0.001361696	0.001455299	0.001482687	0.001182808	0.001345095	0.000892483	0.0011251	0.001651259	0.000998333	0.000965786
b0657	0	0	0	0	0	0	0	0	0	0	0	0	0	0	0	0	0	0	1.48E-05	1.68E-05	1.03E-05	8.90E-06	1.72E-05	1.26E-05	1.25E-05	8.87E-06	1.95E-05	1.26E-05	1.39E-05	1.54E-05	8.18E-06	1.32E-05	1.84E-05	2.32E-05	1.61E-05	1.64E-05
b0662	0	0	0	0	0	0	0	0	0	0	0	0	0	0	0	0	0	0	1.66E-05	1.38E-05	1.09E-05	8.73E-06	1.09E-05	6.29E-06	9.07E-06	9.28E-06	1.22E-05	8.16E-06	9.03E-06	1.32E-05	4.06E-06	8.51E-06	9.09E-06	1.13E-05	8.55E-06	9.92E-06
b0674	0	0	0	0	0	0	0	0	0	0	0	0	0	0	0	0	0	0	0.000378883	0.000457508	0.000378982	0.000514548	0.000492455	0.000634052	0.000420329	0.000367566	0.000452781	0.000549391	0.000512459	0.000470932	0.000364485	0.000533763	0.000493259	0.000474951	0.000633911	0.000793418
b0675	0	0	0	0	0	0	0	0	0	0	0	0	0	0	0	0	0	0	0.000154126	0.000169843	0.000212292	0.00016166	0.000156627	0.00022018	0.000178292	0.000218496	0.000188394	0.000205332	0.000219775	0.000167048	0.000178311	0.000177849	0.000234797	0.000170507	0.000224143	0.000241606
b0677	0	0	0	0	0	0	0	0	0	0	0	0	0	0	0	0	0	0	0.000187364	0.000176269	0.000572905	0.000131525	0.000136436	0.000125097	0.000133323	0.000144564	0.000192156	0.000122434	0.000168749	0.00010447	0.000153322	0.000109223	7.88E-05	0.000121392	0.000111453	0.000113832
b0678	0	0	0	0	0	0	0	0	4.98E-05	0	0	0	0	0	0	0	0	0	0.000226914	0.000232994	0.000672963	0.000177026	0.000220276	0.00013005	0.00018966	0.000148105	0.000310291	0.000176037	0.000213883	0.000193537	0.000111154	0.000137613	0.000111751	0.000208489	0.00014134	0.000147989
b0679	0	0	0	0	0	0	0	0	0	0	0	0	0	0	0	0	0	0	0.000997615	0.000857896	0.000683563	0.000223611	0.000895841	0.000352383	0.000303017	0.000293115	0.000920774	0.000274248	0.000467154	0.000613691	4.68E-05	0.00013573	0.000124098	7.13E-05	0.000224997	0.000131826
b0681	0	0	0	0	0	0	0	0	0	0	0	0	0	0	0	0	0	0	0	0	0	0	0	0	0	0	0	0	0	0	0	0	0	0	0	0
b0684	0	0	0	0	0	0	0	0	0	0	0	0	0	0	0	0	0	0	0.000905084	0.000743298	0.001235534	0.000925669	0.000730006	0.001059927	0.000979491	0.001158215	0.001410466	0.001159326	0.001174204	0.000807335	0.00184288	0.001216904	0.001164069	0.0018689	0.001235018	0.001733085
b0688	0	0	0	0	0	0	0	0	0	0	0	0	0	0	0	0	0	0	0.000837635	0.000743042	0.000508409	0.000662876	0.000737346	0.000737144	0.000634255	0.000508695	0.000613308	0.000648753	0.000575449	0.000640035	0.000498191	0.00051342	0.0005352	0.000487223	0.000489143	0.000579924
b0692	0	0	0	0	0	0	0	0	0	0	0	0	0	0	0	0	0	0	0	0	0	0	0	0	0	0	0	0	0	0	0	0	0	0	0	0
b0693	0	0	0	0	0	0	0	0	0	0	0	0	0	0	0	0	0	0	0	0	0	0	0	0	0	0	0	0	0	0	0	0	0	0	0	0
b0696	0	0	0	0	0	0	0	0	0	0	0	0	0	0	0	0	0	0	0	0	0	0	0	0	0	0	0	0	0	0	0	0	0	0	0	0
b0697	0	0	0	0	0	0	0	0	0	0	0	0	0	0	0	0	0	0	0	0	0	0	0	0	0	0	0	0	0	0	0	0	0	0	0	0
b0698	0	0	0	0	0	0	0	0	0	0	0	0	0	0	0	0	0	0	0	0	0	0	0	0	0	0	0	0	0	0	0	0	0	0	0	0
b0709	0	0	0	0	0	0	0	0	0	0	0	0	0	0	0	0	0	0	0	0	0	0	0	0	0	0	0	0	0	0	0	0	0	0	0	0
b0720	0.00010569	0.000165647	0.000210198	0.006461264	0.000277977	0.000313505	0.000329948	0.00034535	0.000359854	0.000361122	0.000367031	0.000390164	0.000427531	0.000426247	0.00044994	0.000487273	0.000501514	0.000509663	0.012266108	0.011858383	0.007638998	0.013796839	0.010344216	0.01383748	0.006667393	0.007007673	0.007845672	0.006217087	0.009995387	0.010947068	0.003787436	0.005583367	0.003167493	0.003243098	0.006320759	0.004812557
b0721	6.99E-07	1.21E-06	1.66E-06	0.000352192	2.41E-06	2.86E-06	3.07E-06	0.000488748	3.48E-06	3.53E-06	3.58E-06	3.92E-06	4.48E-06	4.47E-06	4.84E-06	5.46E-06	5.71E-06	5.85E-06	0.000242901	0.000252735	0.000101611	0.000247487	0.000274726	0.000219496	0.000151529	0.00021109	0.000204774	0.000110626	0.000189105	0.000267541	2.98E-05	9.82E-05	8.38E-05	5.38E-05	0.000188026	4.94E-05
b0722	6.29E-07	1.09E-06	1.49E-06	0.000316931	2.17E-06	2.58E-06	2.77E-06	0.000439815	3.13E-06	3.17E-06	3.23E-06	3.52E-06	4.03E-06	4.02E-06	4.36E-06	4.92E-06	5.14E-06	5.27E-06	4.76E-06	3.89E-06	1.84E-06	3.61E-06	4.05E-06	4.77E-06	3.63E-06	3.96E-06	3.35E-06	2.83E-06	3.27E-06	3.38E-06	1.77E-06	3.11E-06	2.57E-06	1.07E-06	4.12E-06	3.51E-06
b0723	3.15E-06	5.47E-06	7.46E-06	0.00158673	1.09E-05	1.29E-05	1.38E-05	0.002201955	1.57E-05	1.59E-05	1.61E-05	1.76E-05	2.02E-05	2.01E-05	2.18E-05	2.46E-05	2.57E-05	2.64E-05	0.0032187	0.003147053	0.001545813	0.00346168	0.003038948	0.002490753	0.002039877	0.002021928	0.002136615	0.001477777	0.001910029	0.002941689	0.000764314	0.001430288	0.000915224	0.000843576	0.001424882	0.001006648
b0724	1.31E-06	2.27E-06	3.10E-06	0.000659351	4.52E-06	5.36E-06	5.75E-06	0.000915002	6.52E-06	6.60E-06	6.71E-06	7.33E-06	8.39E-06	8.37E-06	9.06E-06	1.02E-05	1.07E-05	1.10E-05	0.000954993	0.000890197	0.000722499	0.001606614	0.000911714	0.001084482	0.000891333	0.001180606	0.000911085	0.000655996	0.001013636	0.000759032	0.000334137	0.000554976	0.00045297	0.000391286	0.000725356	0.000297773
b0726	0	0	0	0.007007119	0	0	0	0	0	0	0	0	0	0	0	0	0	0	0.002301536	0.002272424	0.001503477	0.003723929	0.002390527	0.003420019	0.002113821	0.002701802	0.002571701	0.002127684	0.002341479	0.002754265	0.001302908	0.002350068	0.001222759	0.001669336	0.002385431	0.001333736
b0727	0	0	0	0.002935352	0	0	0	0	0	0	0	0	0	0	0	0	0	0	0.001720398	0.001670675	0.001333983	0.003826525	0.001805475	0.0033957	0.002330121	0.002382356	0.002447326	0.001799405	0.002107716	0.001932147	0.001675399	0.002225385	0.001576658	0.001725313	0.001817693	0.001749612
b0728	5.48E-05	8.58E-05	0.000108853	0.005233848	0.0001439	0.000162257	0.000170752	0.000178707	0.000186195	0.000186843	0.000189901	0.000201842	0.000221122	0.000220457	0.000232679	0.000251924	0.000259261	0.000263461	0.003987023	0.004017326	0.002809612	0.006869948	0.004298016	0.005917249	0.004459204	0.005291239	0.004592219	0.00355418	0.004654104	0.004616026	0.00133761	0.003382655	0.001754198	0.00140318	0.003142813	0.001602092
[illegible]																																				

b1387	0	0	0	0	0	0	0	0	0	0	0	[illegible]
b1388	0	0	0	0	0	0	0	0	0	0	0	[illegible]
b1389	0	0	0	0	0	0	0	0	0	0	0	[illegible]
b1390	0	0	0	0	0	0	0	0	0	0	0	[illegible]
b1391	0	0	0	0	0	0	0	0	0	0	0	[illegible]
b1392	0	0	0	0	0	0	0	0	0	0	0	[illegible]
b1393	0	0	0	0	0	0	0	0	0	0	0	[illegible]
b1394	0	0	0	0	0	0	0	0	0	0	0	[illegible]
b1395	0	0	0	0	0	0	0	0	0	0	0	[illegible]
b1396	0	0	0	0	0	0	0	0	0	0	0	[illegible]
b1397	0	0	0	0	0	0	0	0	0	0	0	[illegible]
b1398	0	0	0	0	0	0	0	0	0	0	0	[illegible]
b1400	0	0	0	0	0	0	0	0	0	0	0	[illegible]
b1415	0	0	0	0	0	0	0	0	0	0	0	[illegible]
b1430	0	0	0	0	0	0	0	0	0	0	0	[illegible]
b1440	0	0	0	0	0	0	0	0	0	0	0	[illegible]
b1441	0	0	0	0	0	0	0	0	0	0	0	[illegible]
b1442	0	0	0	0	0	0	0	0	0	0	0	[illegible]
b1443	0	0	0	0	0	0	0	0	0	0	0	[illegible]
b1444	0	0	0	0	0	0	0	0	0	0	0	[illegible]
b1448	0	0	0	0	0	0	0	0	0	0	0	[illegible]
b1449	0	0	0	0	0	0	0	0	0	0	0	[illegible]
b1453	0	0	0	0	0	0	0	0	0	0	0	[illegible]
b1463	0	0	0	0	0	0	0	0	0	0	0	[illegible]
b1465	0	0	0	0	0	0	0	0	0	0	0	[illegible]
b1466	0	0	0	0	0	0	0	0	0	0	0	[illegible]
b1467	0	0	0	0	0	0	0	0	0	0	0	[illegible]
b1468	0	0	0	0	0	0	0	0	0	0	0	[illegible]
b1469	0	0	0	0	0	0	0	0	0	0	0	[illegible]
[illegible]	[illegible]	[illegible]	[illegible]	[illegible]	[illegible]	[illegible]	[illegible]	[illegible]	[illegible]	[illegible]	[illegible]	[illegible]

b2173	0	0	0	0	0	0	0	0	0	0	0	0	0	0	0	0	0	0	3.48E-06	3.39E-06	5.20E-06	6.44E-06	6.00E-06	6.72E-06	6.75E-06	7.71E-06	8.19E-06	7.00E-06	6.01E-06	5.36E-06	9.97E-06	7.46E-06	1.34E-05	8.35E-06	1.05E-05	1.18E-05
b2174	0	0	0	0	0	0	0	0	0	0	0	0	0	0	0	0	0	0	6.38E-07	1.79E-06	9.73E-07	2.85E-07	1.96E-06	8.32E-07	1.52E-06	1.50E-06	3.98E-07	7.27E-07	1.42E-06	2.59E-06	1.48E-06	3.24E-06	3.12E-06	1.22E-06	4.27E-06	3.73E-06
[illegible]																																				

b2867	0	0	0	0	0	0	0	0	0	0	0	0	0	0	0	0	0	0	7.91E-07	2.02E-05	5.71E-07	2.46E-07	3.94E-06	8.50E-07	2.14E-06	1.18E-06	1.18E-06	6.02E-07	0	1.43E-06	3.50E-08	0	1.95E-06	9.57E-07	0	4.08E-08
b2868	0	0	0	0	0	0	0	0	0	0	0	0	0	0	0	0	0	0	7.48E-07	4.62E-07	1.23E-07	2.83E-08	2.77E-08	1.95E-07	9.82E-07	2.11E-07	2.23E-07	1.61E-06	0	4.84E-08	5.08E-09	0	2.29E-07	1.50E-07	0	2.52E-07
b2871	0	0	0	0	0	0	0	0	0	0	0	0	0	0	0	0	0	0	0	0	0	0	0	0	0	0	0	0	0	0	0	0	0	0	0	0
b2873	0	0	0	0	0	0	0	0	0	0	0	0	0	0	0	0	0	0	0	0	0	0	0	0	0	0	0	0	0	0	0	0	0	0	0	0
b2877	0	0	0	0	0	0	0	0	0	0	0	0	0	0	0	0	0	0	4.66E-06	1.08E-06	2.03E-06	2.30E-06	7.07E-06	1.36E-06	2.42E-06	1.28E-06	1.54E-06	1.63E-06	0	2.85E-06	5.15E-08	0	6.48E-06	1.94E-07	0	1.52E-06
b2882	0	0	0	0	0	0	0	0	0	0	0	0	0	0	0	0	0	0	0	0	0	0	0	0	0	0	0	0	0	0	0	0	0	0	0	0
b2883	0	0	0	0	0	0	0	0	0	0	0	0	0	0	0	0	0	0	1.07E-06	1.01E-06	1.51E-06	7.17E-07	3.62E-06	3.97E-07	2.69E-06	9.36E-07	1.72E-06	2.25E-06	0	1.29E-06	1.09E-06	0	1.84E-06	1.14E-06	0	9.72E-07
b2888	0	0	0	0	0	0	0	0	0	0	0	0	0	0	0	0	0	0	0	0	0	0	0	0	0	0	0	0	0	0	0	0	0	0	0	0
b2889	1.62E-08	2.56E-08	3.28E-08	3.48E-08	4.38E-08	4.98E-08	5.25E-08	5.51E-08	5.76E-08	5.78E-08	5.88E-08	6.27E-08	6.92E-08	6.90E-08	7.31E-08	7.96E-08	8.22E-08	8.36E-08	9.82E-06	8.40E-06	1.11E-05	5.91E-06	4.77E-06	1.62E-06	3.52E-06	2.47E-06	2.03E-06	2.79E-06	1.74E-06	4.10E-06	4.76E-06	1.17E-06	2.34E-06	4.36E-06	2.13E-06	3.25E-06
b2893	0	0	0	0	0	0	0	0	0	0	0	0	0	0	0	0	0	0	5.04E-05	5.61E-05	8.18E-05	0.000102764	5.65E-05	0.000212904	0.000138943	7.64E-05	9.51E-05	0.000117925	8.91E-05	7.45E-05	0.000124634	8.32E-05	0.000248215	0.000124033	0.000107019	0.000113344
b2895	4.03E-05	6.38E-05	8.16E-05	0.000100017	0.000109187	0.000123941	0.000130776	0.000137231	0.000143358	0.000142886	0.000146402	0.000156219	0.000172253	0.000171736	0.000181956	0.000198267	0.000204559	0.000208135	1.49E-05	1.46E-05	2.56E-05	3.24E-05	2.49E-05	4.13E-05	3.67E-05	4.84E-05	5.53E-05	3.95E-05	5.45E-05	2.78E-05	6.40E-05	6.39E-05	9.27E-05	5.11E-05	5.70E-05	4.34E-05
b2901	0	0	0	0	0	0	0	0	0	0	0	0	0	0	0	0	0	0	0.000102904	0.000110268	0.000143945	0.000129185	0.000102182	0.000171406	0.000149288	0.000120089	0.000160069	0.000150507	0.000113654	0.000109635	0.000130025	0.000145902	0.000139265	0.000138593	0.00011247	0.000155207
b2903	0	0	0	0	0	0	0	0	0	0	0	0	0	0	0	0	0	0	0.000208585	0.000222792	0.000202378	0.000186226	0.000199213	0.000174911	0.000255433	0.000324694	0.000232821	0.000274191	0.000285457	0.000335501	0.000166918	0.000243035	0.000239985	0.000222872	0.000342476	0.000265105
b2904	0	0	0	0	0	0	0	0	0	0	0	0	0	0	0	0	0	0	2.62E-05	2.31E-05	3.32E-05	3.73E-05	2.95E-05	4.27E-05	4.67E-05	4.29E-05	4.07E-05	5.21E-05	4.86E-05	4.21E-05	4.93E-05	5.67E-05	6.10E-05	4.89E-05	5.28E-05	7.41E-05
b2905	0	0	0	0	0	0	0	0	0	0	0	0	0	0	0	0	0	0	5.52E-05	6.34E-05	9.00E-05	8.53E-05	6.49E-05	9.48E-05	0.000130519	0.000126957	0.000166447	0.000145303	0.000110814	0.00011704	0.000129022	0.000110589	0.000181297	0.000149022	0.000148184	0.000151535
b2906	0	0	0	0	0	0	0	0	0	0	0	0	0	0	0	0	0	0	6.04E-06	7.07E-06	4.01E-06	4.70E-06	6.82E-06	6.71E-06	6.27E-06	6.15E-06	7.37E-06	6.33E-06	5.52E-06	6.89E-06	3.53E-06	5.75E-06	5.05E-06	5.61E-06	6.21E-06	6.02E-06
b2907	0	0	0	0	0	0	0	0	0	0	0	0	0	0	0	0	0	0	6.97E-06	1.10E-05	1.12E-05	1.38E-05	1.55E-05	1.82E-05	1.76E-05	1.94E-05	2.18E-05	1.52E-05	0	1.71E-05	1.82E-05	0	1.82E-05	1.90E-05	0	2.01E-05
b2912	0	0	0	0	0	0	0	0	0	0	0	0	0	0	0	0	0	0	5.96E-07	1.26E-06	1.14E-06	1.05E-06	1.66E-06	1.86E-06	2.57E-06	2.28E-06	4.06E-06	1.66E-06	0	1.63E-06	9.69E-07	0	5.08E-06	1.24E-06	0	1.20E-06
b2913	0.000109802	0.000174249	0.000223161	0.000237511	0.000299169	0.000339937	0.00035883	0.000376692	0.000393671	0.00039564	0.000402114	0.000429339	0.000473891	0.000472468	0.000500895	0.000546359	0.000563931	0.000573909	0.001507415	0.001700303	0.001591044	0.001739949	0.002173895	0.002089595	0.001642661	0.001436986	0.002451658	0.00197105	0.000980251	0.003081779	0.003373079	0.003670138	0.003420979	0.004126201	0.002226149	0.002957467
b2914	0	0	0	0	0	0	0	0	0	0	0	0	0	0	0	0	0	0	0.000372541	0.000405154	0.000392237	0.000456958	0.000409021	0.000456846	0.000469417	0.000373871	0.000393543	0.000411208	0.000354189	0.000456487	0.000582967	0.000483251	0.000429929	0.000452681	0.000341665	0.000570285
b2917	0	0	0	0	0	0	0	0	0	0	0	0	0	0	0	0	0	0	0	0	0	0	0	0	0	0	0	0	0	0	0	0	0	0	0	0
b2918	0	0	0	0	0	0	0	0	0	0	0	0	0	0	0	0	0	0	0	0	0	0	0	0	0	0	0	0	0	0	0	0	0	0	0	0
b2919	0	0	0	0	0	0	0	0	0	0	0	0	0	0	0	0	0	0	0	0	0	0	0	0	0	0	0	0	0	0	0	0	0	0	0	0
b2920	0	0	0	0	0	0	0	0	0	0	0	0	0	0	0	0	0	0	0	0	0	0	0	0	0	0	0	0	0	0	0	0	0	0	0	0
b2923	0	0	0	0	0	0	0	0	0	0	0	0	0	0	0	0	0	0	0	0	0	0	0	0	0	0	0	0	0	0	0	0	0	0	0	0
b2924	0	0	0	0	0	0	0	0	0	0	0	0	0	0	0	0	0	0	0.000447506	0.000494248	0.000489528	0.000427907	0.000508619	0.00070623	0.000528985	0.000726082	0.000893876	0.000691082	0.000464714	0.000750639	0.00054571	0.000608479	0.000601297	0.00080262	0.000693489	0.000852898
b2925	0.000476508	0.000743703	0.000940648	0	0.001237641	0	0	0	0.001591741	0.003133967	0.001622499	0.001721564	0.001880518	0.005612495	0.001975291	0.002132225	0.002191635	0.002225762	0.002818795	0.003020726	0.003880796	0.00375398	0.002965592	0.004233252	0.004136518	0.003684348	0.004111641	0.004499012	0.003090833	0.00383768	0.006465358	0.004754201	0.005353195	0.006072904	0.006294658	0.004172817
b2926	0.000525802	0.000831754	0.00107109	2.29E-05	0.001419571	6.82E-05	3.54E-05	3.73E-05	0.001812016	0.000986373	0.001855242	0.002025242	0.002231029	0.002251091	0.002355395	0.002564155	0.002579722	0.002690334	0.008954482	0.00816669	0.010797715	0.010351494	0.008953221	0.008977413	0.009682598	0.008601254	0.009315896	0.010384689	0.007641258	0.009156978	0.012765488	0.011725065	0.011229236	0.011222587	0.012424126	0.010360946
b2927	0	0	0	0	0	0	0	0	0	0	0	0	0	0	0	0	0	0	1.15E-05	1.36E-05	9.12E-06	1.75E-05	1.62E-05	2.37E-05	2.29E-05	1.31E-05	1.75E-05	3.03E-05	8.71E-06	2.26E-05	1.24E-05	1.68E-05	2.85E-05	1.06E-05	1.72E-05	2.08E-05
b2930	0	0	0	0	0	0	0	0	0	0	0	0	0	0	0	0	0	0	0	0	0	0	0	0	0	0	0	0	0	0	0	0	0	0	0	0
b2933	0	0	0	0	0	0	0	0	0	0	0	0	0	0	0	0	0	0	0	0	0	0	0	0	0	0	0	0	0	0	0	0	0	0	0	0
b2934	0	0	0	0	0	0	0	0	0	0	0	0	0	0	0	0	0	0	0	0	0	0	0	0	0	0	0	0	0	0	0	0	0	0	0	0
b2935	0.001129526	0.001762289	0.002228375	0.00016991	0.002930719	0.003294833	0.000252234	0.000264028	0.003767178	0.0077021	0.003839779	0.004073594	0.004448543	0.014661447	0.004671985	0.005041782	0.005181681	0.00526207	0.001109907	0.001318454	0.001029957	0.001623603	0.001646804	0.002058374	0.002019179	0.002021228	0.001706472	0.001802227	0.001699843	0.001761589	0.00125012	0.001988305	0.001912992	0.001521336	0.001738374	0.001583249
b2937	0	0	0	0	0	0	0	0	0	0	0	0	0	0	0	0	0	0	0.000331809	0.000247544	0.000243606	0.000254961	0.000206012	0.000238771	0.000284855	0.000201107	0.000223159	0.000547215	0.000192679	0.000226572	0.000197153	0.000181242	0.000293689	0.000220747	0.000205139	0.000148897
b2938	0	0	0	0	0	0	0	0	0	0	0	0	0	0	0	0	0	0	0.000437545	0.000457422	0.00048107	0.000397598	0.000398409	0.000564489	0.000465125	0.000401114	0.000484317	0.000600741	0.000445815	0.000480632	0.000297939	0.000451697	0.000652736	0.000518188	0.000534729	0.000399484
b2942	3.82E-05	6.05E-05	7.74E-05	8.23E-05	0.000103601	0.0001176	0.000124085	0.00013021	0.000136023	0.000136676	0.000138912	0.000148226	0.00016344	0.000162949	0.000172647	0.000188123	0.000194093	0.000197486	0.001562	0.00168543	0.001813121	0.001256563	0.001774107	0.002207082	0.001666532	0.001905192	0.002300244	0.002105353	0.001994198	0.002322319	0.003798126	0.003366179	0.003576509	0.006477608	0.002348888	0.00430158
b2943	0.000167192	0.000264569	0.001397065	0	0.000451822	0	0	0	0	0	0	0.000644958	0.000710618	0	0.000750309	0.000816949	0	0.000857234	0	0	0	0	0	0	0	0	0	0	0	0	0	0	0	0	0	0
b2947	0	0	0	0	0	0	0	0	0	0	0	0	0	0	0	0	0	0	0.000304929	0.000288106	0.000337834	0.0003352	0.000275156	0.000295966	0.000283164	0.000297815	0.000277309	0.000301889	0.00030346	0.000261367	0.000314244	0.000322717	0.000310182	0.00032678	0.000296885	0.00023446
b2954	0	0	0	0	0	0	0	0	0	0	0	0	0	0	0	0	0	0	4.37E-05	5.01E-05	5.07E-05	5.70E-05	4.96E-05	7.43E-05	6.54E-05	7.02E-05	7.30E-05	7.29E-05	7.46E-05	5.86E-05	6.20E-05	5.52E-05	9.71E-05	4.27E-05	6.44E-05	4.19E-05
b2957	0	0	0	0	0	0	0	0	0	0	0	0	0	0	0	0	0	0	3.29E-07	4.17E-07	8.91E-07	4.68E-07	5.97E-07	9.84E-07	6.34E-07	2.76E-07	4.99E-07	8.83E-07	4.10E-07	3.45E-07	1.56E-06	6.62E-07	1.18E-06	1.26E-06	2.91E-07	2.79E-07
b2963	0	0	0	0	0	0	0	0	0	0	0	0	0	0	0	0	0	0	1.36E-06	4.50E-06	1.02E-05	2.85E-07	5.09E-06	2.40E-07	2.50E-07	1.01E-05	3.99E-07	4.20E-07	8.91E-06	1.73E-06	1.09E-05	9.10E-06	9.94E-08	1.02E-05	1.03E-05	1.26E-05
b2964	0	0	0	0	0	0	0	0	0	0	0	0	0	0	0	0	0	0	0	0	0	0	0	0	0	0	0	0	0	0	0	0	0	0	0	0
b2965	0	0	0	0	0	0	0	0	0	0	0	0	0	0	0	0	0	0	2.21E-05	2.06E-05	2.34E-05	2.29E-05	2.34E-05	6.33E-05	3.80E-05	2.74E-05	3.03E-05	5.42E-05	2.49E-05	2.58E-05	1.90E-05	3.69E-05	4.64E-05	3.47E-05	3.22E-05	2.99E-05
b2975	0	0	0	0	-1.98E-36	0	0	0	0	0	0	0	-2.41E-37	0	0	0	0	0	7.44E-05	6.37E-05	1.41E-05	2.00E-05	3.48E-05	8.10E-07	1.02E-05	3.18E-06	1.29E-05	3.18E-06	8.33E-06	6.37E-06	1.54E-08	1.36E-06	4.07E-07	6.24E-07	3.49E-07	5.44E-07
b2976	0	0	0	0	0	0	0	0	0	0	0	0	0	0	0	0	0	0	0.001120113	0.001305117	0.000906013	0.001616288	0.001010681	0.000180343	0.000608156	0.000545045	0.000636123	0.000454031	0.000693567	0.000484123	0.000158157	0.000244952	0.000155027	0.000169808	0.000234154	0.00019884
b2979	0	0	0	0	0	0	0	0	0	0	0	0	0	0	0	0	0	0	8.99E-06	6.54E-06	1.08E-05	2.17E-05	6.94E-06	4.93E-06	1.00E-05	1.59E-05	6.04E-06	9.18E-06	1.64E-05	7.15E-06	5.36E-06	1.42E-05	1.23E-05	1.16E-05	1.66E-05	7.94E-06
b2987	0	0	0	0	0	0	0	0	0	0	0	0	0	0	0	0	0	0	0	0	0	0	0	0	0	0	0	0	0	0	0	0	0	0	0	0
b2988	0	0	0	0	0	0	0	0	0	0	0	0	0	0	0	0	0	0	3.28E-05	2.65E-05	4.01E-05	4.03E-05	3.04E-05	3.73E-05	3.60E-05	3.17E-05	3.77E-05	4.31E-05	4.24E-05	3.95E-05	9.71E-05	9.94E-05	8.83E-05	0.000215158	7.55E-05	0.000162725
b2989	0	0	0	0	0	0	0	0	0	0	0	0	0	0	0	0	0	0	0.000355434	0.000268342	0.000369043	0.000272264	0.000262041	0.000228738	0.000277174	0.000234027	0.000224376	0.000247739	0.000229319	0.000203403	0.000222722	0.000188667	0.00016995	0.0002301	0.000152616	0.000136026
b2994	0	0	0	0	0	0	0	0	0	0	0	0	0	0	0	0	0	0	1.64E-05	2.36E-05	1.19E-06	2.36E-06	2.49E-05	1.80E-06	4.00E-06	4.76E-06	1.98E-06	2.33E-06	2.95E-06	3.05E-06	9.04E-07	2.05E-06	2.31E-06	5.81E-07	1.03E-06	3.52E-06
b2995	0	0	0	0	0	0	0	0	0	0	0	0	0	0	0	0	0	0	0	0	0	0	0	0	0	0	0	0	0	0	0	0	0	0	0	0
b2996	0	0	0	0	0	0	0	0	0	0	0	0	0	0	0	0	0	0	2.55E-06	1.45E-06	3.59E-08	3.84E-05	3.61E-06	1.44E-06	1.15E-05	3.67E-06	2.35E-05	8.94E-06	0	1.18E-05	7.26E-06	0	3.63E-05	5.74E-06	0	7.85E-06
b2997	0	0	0	0	0	0	0	0	0	0	0	0	0	0	0	0	0	0	0	0	0	0	0	0	0	0	0	0	0	0	0	0	0	0	0	0
b3001	0	0	0	0	0	0	0	0	0	0	0	0	0	0	0	0	0	0	0.000314433	0.000223298	0.000196811	0.000272239	0.000197059	0.000169963	0.000179338	0.000166277	0.000108119	9.51E-05	0.000136252	0.000145408	3.66E-05	6.37E-05	4.43E-05	4.20E-05	6.48E-05	4.94E-05
b3003	0	0	0	0	0	0	0	0	0	0	0	0	0	0	0	0	0	0	9.43E-05	6.73E-05	0.000100795	4.96E-05	3.16E-05	2.26E-05	3.47E-05	2.37E-05	4.59E-05	2.46E-05	3.82E-05	2.45E-05	4.64E-05	1.28E-05	3.21E-05	5.36E-05	1.10E-05	3.51E-05
b3005	0	0	0	0	0	0	0	0	0	0	0	0	0	0	0	0	0	0	9.27E-06	1.33E-05	2.14E-05	1.86E-05	1.44E-05	2.87E-05	2.62E-05	3.05E-05	2.46E-05	3.22E-05	2.21E-05	2.35E-05	9.93E-05	6.16E-05	3.90E-05	6.14E-05	3.45E-05	4.58E-05
b3006	0	0	0	0	0	0	0	0	0	0	0	0	0	0	0	0	0	0	6.05E-05	8.06E-05	8.55E-05	0.000101227	0.000100556	0.000149738	0.000128748	0.000170376	0.000134979	0.000134741	0.00012337	0.000154085	0.000255979	0.000251302	0.000146775	0.000208763	0.00023225	0.0002674
b3008	0.000285182	0.000447072	0.000567421	0.000600322	0.00075061	0.000846693	0.000891165	0.000932828	0.000972077	0.000975542	0.000991505	0.001054111	0.001155287	0.001151818	0.001215985	0.001317144	0.001355751	0.001377832	0.000342476	0.000372068	0.000239806	0.000277533	0.000354875	0.000349591	0.000311793	0.000208994	0.000254211	0.000319742	0.000319442	0.000362456	0.000173271	0.000310181	0.000627167	0.00030405	0.000248771	0.00019366
b3011	0	0	0	0	0	0	0	0	0	0	0	0	0	0	0	0	0	0	0.000138344	0.000137878	0.000129879	0.000160923	0.000121512	0.000149371	0.000140214	8.41E-05	0.000141532	0.000502314	0.000158673	0.000202951	0.000423403	0.000520551	0.000302015	0.000183936	0.000741024	0.000920822
b3012	0	0	0	0	0	0	0	0	0	0	0	0	0	0	0	0	0	0	0.000130665	0.000103865	0.000125734	8.44E-05	8.17E-05	6.35E-05	8.43E-05	4.75E-05	0.000101946	0.000107161	4.75E-05	0.00010462	0.000187882	9.08E-05	6.95E-05	0.000133549	7.99E-05	0.000130539
b3018	1.04E-05	1.62E-05	2.04E-05	2.15E-05	2.67E-05	2.99E-05	3.14E-05	3.28E-05	3.41E-05	3.41E-05	3.47E-05	3.67E-05	3.99E-05	3.98E-05	4.18E-05	4.50E-05	4.61E-05	4.68E-05	1.06E-05	1.31E-05	7.19E-06	5.69E-06	1.56E-05	9.71E-06	5.48E-06	6.07E-06	1.17E-05	8.10E-06	7.42E-06	1.67E-05	6.70E-06	1.25E-05	8.00E-06	7.65E-06	1.20E-05	8.17E-06
b3028	0	0	0	0	0	0	0	0	0	0	0	0	0	0	0	0	0	0	5.50E-05	7.11E-05	6.02E-05	8.17E-05	7.65E-05	7.53E-05	7.03E-05	8.69E-05	9.10E-05	9.32E-05	8.70E-05	8.84E-05	0.000189012	0.000118599	6.68E-05	0.000155009	0.000112631	0.000161676
b3029	0	0	0	0	0	0	0	0	0	0	0	0	0	0	0	0	0	0	3.37E-05	3.54E-05	4.00E-05	5.57E-05	3.60E-05	4.28E-05	4.47E-05	4.31E-05	5.20E-05	4.49E-05	4.15E-05	3.78E-05	9.45E-05	5.94E-05	4.79E-05	0.000131987	4.34E-05	9.28E-05
b3032	0	0	0	0	0	0	0	0	0	0	0	0	0	0	0	0	0	0	8.54E-05	5.94E-05	6.47E-05	4.46E-05	4.21E-05	5.12E-05	4.68E-05	4.55E-05	5.88E-05	4.87E-05	4.77E-05	3.81E-05	6.25E-05	5.29E-05	3.59E-05	5.41E-05	4.45E-05	4.52E-05
b3034	0	0	0	0	0	0	0	0	0	0	0	0	0	0	0	0	0	0	1.82E-05	2.38E-05	2.76E-05	2.13E-05	1.82E-05	2.14E-05	2.22E-05	1.70E-05	2.02E-05	2.17E-05	3.07E-05	1.46E-05	2.17E-05	1.73E-05	2.24E-05	1.51E-05	1.89E-05	2.34E-05
b3035	0	0	0	0	0	0	0	0	0	0	0	0	0	0	0	0	0	0	0.001369911	0.001297492	0.002062755	0.001566009	0.001250588	0.001557867	0.001721791	0.001871005	0.001703991	0.001895959	0.001914778	0.001268775	0.00196045	0.001954614	0.001790591	0.001895361	0.001895999	0.001844875
b3040	1.15E-06	1.83E-06	2.34E-06	2.48E-06	3.13E-06	3.55E-06	3.74E-06	3.93E-06	4.10E-06	4.12E-06	4.19E-06	4.47E-06	4.93E-06	4.92E-06	5.21E-06	5.68E-06	5.86E-06	5.96E-06	0	0	0	0	0	0	0	0	0	0	0	0	0	0	0	0	0	0
b3041	1.26E-05	1.99E-05	2.55E-05	2.71E-05	3.41E-05	3.87E-05	4.08E-05	4.28E-05	4.47E-05	4.49E-05	4.57E-05	4.87E-05	5.37E-05	5.36E-05	5.68E-05	6.18E-05	6.38E-05	6.49E-05	2.33E-05	3.79E-05	5.41E-05	4.55E-05	2.86E-05	8.43E-05	6.26E-05	3.92E-05	7.71E-05	0.000108326	4.13E-05	4.46E-05	6.36E-05	5.41E-05	5.90E-05	6.53E-05	4.27E-05	4.02E-05
b3052	0	0	0	0	0	0	0	0	0	0	0	0	0	0	0	0	0	0	0.000216993	0.000223254	0.000277559	0.000182855	0.000215771	0.000280153	0.000251359	0.000256	0.000232195	0.000278273	0.000282562	0.000211643	0.000191515	0.000299054	0.000279171	0.000195848	0.000275933	0.000310099
b3057	0	0	0	0	0	0	0	0	0	0	0	0	0	0	0	0	0	0	0	0	0	0	0	0	0	0	0	0	0	0	0	0	0	0	0	0
b3058	1.72E-07	2.72E-07	3.48E-07	3.71E-07	4.66E-07	5.29E-07	5.58E-07	5.86E-07	6.12E-07	6.15E-07	6.25E-07	6.67E-07	7.36E-07	7.33E-07	7.77E-07	8.47E-07	8.73E-07	8.89E-07	7.56E-06	1.24E-05	9.04E-06	1.03E-05	1.24E-05	6.93E-06	1.05E-05	7.22E-06	3.56E-06	1.62E-05	1.15E-06	1.18E-05	1.33E-05	1.10E-05	1.69E-06	5.84E-06	1.02E-05	4.34E-06
b3059	5.07E-05	7.88E-05	9.93E-05	0.000104482	0.000129786	0.000145409	0.000152619	0.000159304	0.000165531	0.00016588	0.000168594	0.00017845	0.000194104	0.000193521	0.000203352	0.000218517	0.000224187	0.000227462	4.76E-06	3.42E-06	4.12E-07	1.64E-07	1.96E-06	1.19E-06	3.51E-07	6.39E-07	2.31E-07	1.06E-06	7.90E-07	1.72E-06	2.32E-07	2.37E-07	3.61E-08	8.01E-07	9.30E-07	5.26E-07
b3061	0	0	0	0	0	0	0	0	0	0	0	0	0	0	0	0	0	0	0	0	0	0	0	0	0	0	0	0	0	0	0	0	0	0	0	0
b3062	0	0	0	0	0	0	0	0	0	0	0	0	0	0	0	0	0	0	0	0	0	0	0	0	0	0	0	0	0	0	0	0	0	0	0	0
b3063	0	0	0	0	0	0	0	0	0	0	0	0	0	0	0	0	0	0	0	0	0	0	0	0	0	0	0	0	0	0	0	0	0	0	0	0
b3070	0	0	0	0	0	0	0	0	0	0	0	0	0	0	0	0	0	0	6.67E-05	6.88E-05	8.80E-05	0.00010091	6.96E-05	8.61E-05	0.000101002	9.53E-05	9.04E-05	8.94E-05	7.39E-05	9.81E-05	0.00014605	9.87E-05	8.56E-05	0.000119274	9.22E-05	0.000127773
b3073	0	0	0	0	0	0	0	0	0	0	0	0	0	0	0	0	0	0	8.02E-06	1.04E-05	1.11E-05	1.47E-05	1.23E-05	8.86E-06	1.74E-05	1.03E-05	1.25E-05	2.05E-05	1.10E-05	1.36E-05	8.12E-06	1.48E-05	1.16E-05	6.29E-06	1.11E-05	4.34E-06
b3089	0	0	0	0	0	0	0	0	0	0	0	0	0	0	0	0	0	0	0.000127923	0.000113769	3.97E-05	4.64E-05	9.91E-05	3.00E-05	3.13E-05	4.72E-05	4.46E-05	2.99E-05	2.26E-05	0.000119578	3.37E-05	5.38E-05	1.71E-05	4.09E-05	4.94E-05	2.16E-05
b3091	0	0	0	0	0	0	0	0	0	0	0	0	0	0	0	0	0	0	0	0	0	0	0	0	0	0	0	0	0	0	0	0	0	0	0	0
b3092	0	0	0	0	0	0	0	0	0	0	0	0	0	0	0	0	0	0	0.000118026	0.000114808	8.97E-05	7.64E-05	8.82E-05	7.53E-05	9.50E-05	7.18E-05	9.83E-05	9.07E-05	9.25E-05	8.35E-05	4.60E-05	6.02E-05	5.26E-05	5.15E-05	0.000114483	7.90E-05
b3093	0	0	0	0	0	0	0	0	0	0	0	0	0	0	0	0	0	0	0	0	0	0	0	0	0	0	0	0	0	0	0	0	0	0	0	0
b3114	0	0	0	0	0	0	0	0	0	0	0	0	0	0	0	0	0	0	2.09E-08	2.39E-07	1.07E-05	1.35E-08	3.36E-07	2.41E-08	4.33E-07	6.95E-07	2.95E-06	6.79E-07	0	4.50E-06	1.68E-06	0	1.79E-05	1.09E-06	0	8.25E-07
b3115	0	0	0	0	0	0	0	0	0	0	0	0	0	0	0	0	0	0	0	0	0	0	0	0	0	0	0	0	0	0	0	0	0	0	0	0
b3116	0	0	0	0	0	0	0	0	0	0	0	0	0	0	0	0	0	0	0	0	0	0	0	0	0	0	0	0	0	0	0	0	0	0	0	0
b3117	2.36E-05	3.70E-05	4.69E-05	4.97E-05	6.21E-05	7.00E-05	7.37E-05	7.71E-05	8.04E-05	8.07E-05	8.20E-05	8.72E-05	9.55E-05	9.53E-05	0.000100565	0.00010893	0.000112122	0.000113949	0	0	0	0	0	0	0	0	0	0	0	0	0	0	0	0	0	0
b3124	0	0	0	0	0	0	0	0	0	0	0	0	0	0	0	0	0	0	0	0	0	0	0	0	0	0	0	0	0	0	0	0	0	0	0	0
b3125	0	0	0	0	0	0	0	0	0	0	0	0	0	0	0	0	0	0	1.43E-05	8.27E-06	5.76E-06	6.34E-06	8.77E-06	5.48E-06	1.03E-05	6.97E-06	7.61E-06	7.68E-06	6.82E-06	6.45E-06	4.63E-06	4.83E-06	5.53E-06	3.43E-06	4.22E-06	7.15E-06
b3126	0	0	0	0	0	0	0	0	0	0	0	0	0	0	0	0	0	0	5.32E-06	3.00E-06	9.83E-07	1.82E-06	2.78E-06	8.48E-07	4.08E-07	1.60E-06	9.88E-07	2.21E-06	1.10E-06	2.56E-06	3.27E-07	2.48E-07	6.16E-07	2.24E-06	1.46E-06	1.30E-06
b3127	0	0	0	0	0	0	0	0	0	0	0	0	0	0	0	0	0	0	0	0	0	0	0	0	0	0	0	0	0	0	0	0	0	0	0	0
b3128	0	0	0	0	0	0	0	0	0	0	0	0	0	0	0	0	0	0	2.08E-05	1.24E-05	3.54E-06	4.86E-06	8.49E-06	4.42E-06	7.73E-06	3.79E-06	2.95E-06	3.53E-06	3.29E-06	3.50E-06	1.97E-06	2.81E-06	3.44E-06	1.61E-06	1.44E-06	2.85E-06
b3132	0	0	0	0	0	0	0	0	0	0	0	0	0	0	0	0	0	0	3.36E-07	1.44E-06	4.87E-07	2.81E-07	3.04E-06	9.14E-07	7.45E-06	1.71E-06	9.67E-07	2.23E-06	0	1.18E-06	7.17E-07	0	1.88E-06	8.60E-07	0	1.40E-06
b3137	0	0	0	0	0	0	0	0	0	0	0	0	0	0	0	0	0	0	0	0	0	0	0	0	0	0	0	0	0	0	0	0	0	0	0	0
b3161	0	0	0	0	0	0	0	0	0	0	0	0	0	0	0	0	0	0	0	0	0	0	0	0	0	0	0	0	0	0	0	0	0	0	0	0
b3172	0.000541209	0.000848421	0.001076799	0.00113923	0.001424424	0.001606744	0.001691128	0.001770194	0.001844665	0.001851232	0.001881523	0.002000321	0.0021923	0.002185716	0.002307469	0.002499414	0.00257266	0.002614565	0.001139039	0.001104391	0.001261123	0.001008689	0.001003982	0.001293268	0.001208426	0.001436243	0.00149893	0.001576721	0.001458324	0.001542749	0.001668133	0.001697135	0.002833539	0.00206141	0.001936155	0.002674811
b3176	0.00014199	0.000220561	0.000277902	0.000292531	0.000363382	0.000407124	0.000427317	0.000446027	0.000463463	0.000464445	0.000472045	0.000499642	0.000543466	0.000541834	0.000569366	0.000611797	0.000627675	0.000636845	0.000548503	0.00052834	0.000388532	0.000292647	0.00048112	0.000411043	0.000364655	0.000340307	0.000434231	0.000386185	0.000419299	0.000494099	0.000369193	0.000437734	0.000352659	0.00042614	0.000446624	0.000496061
b3177	3.04E-06	4.81E-06	6.16E-06	6.55E-06	8.24E-06	9.35E-06	9.87E-06	1.04E-05	1.08E-05	1.09E-05	1.10E-05	1.18E-05	1.30E-05	1.30E-05	1.37E-05	1.50E-05	1.54E-05	1.57E-05	1.68E-05	2.16E-05	2.77E-05	3.79E-05	2.86E-05	3.62E-05	4.05E-05	4.35E-05	4.30E-05	3.75E-05	3.00E-05	4.01E-05	5.45E-05	4.35E-05	4.66E-05	4.47E-05	3.54E-05	4.25E-05
b3182	0	0	0	0	0	0	0	0	0	0	0	0	0	0	0	0	0	0	1.06E-06	1.57E-06	1.02E-06	1.64E-06	1.93E-06	2.21E-06	1.75E-06	2.50E-06	2.07E-06	1.84E-06	1.51E-06	1.84E-06	1.74E-06	2.24E-06	2.95E-06	1.89E-06	1.99E-06	4.25E-06
b3187	3.36E-07	5.32E-07	6.80E-07	7.24E-07	9.11E-07	1.03E-06	1.09E-06	1.14E-06	1.20E-06	1.20E-06	1.22E-06	1.30E-06	1.44E-06	1.43E-06	1.52E-06	1.65E-06	1.71E-06	1.74E-06	1.80E-05	1.71E-05	9.97E-06	1.93E-05	1.60E-05	2.04E-05	2.07E-05	1.29E-05	2.22E-05	1.54E-05	1.39E-05	1.38E-05	8.11E-06	1.25E-05	1.97E-05	1.05E-05	1.28E-05	9.33E-06
b3189	6.33E-05	9.84E-05	0.00012397	0.000130497	0.000162104	0.000181616	0.000190624	0.000198973	0.00020675	0.000207186	0.000210576	0.000222888	0.000242442	0.000241713	0.000253992	0.000272918	0.00028	0.000284092	0.000142098	0.00014621	0.000138982	0.000133912	0.000157259	0.000185767	0.000139725	0.000138423	0.000197178	0.000211813	0.000122635	0.000168975	0.000171004	0.000172225	0.000198783	0.000125577	0.00015839	0.000139338
b3196	9.41E-06	1.49E-05	1.91E-05	2.03E-05	2.55E-05	2.90E-05	3.06E-05	3.21E-05	3.35E-05	3.37E-05	3.42E-05	3.65E-05	4.03E-05	4.01E-05	4.25E-05	4.63E-05	4.78E-05	4.86E-05	0	0	0	0	0	0	0	0	0	0	0	0	0	0	0	0	0	0
b3197	0	0	0	0	0	0	0	0	0	0	0	0	0	0	0	0	0	0	1.85E-05	2.19E-05	1.37E-05	1.92E-05	2.20E-05	2.46E-05	2.03E-05	1.54E-05	1.68E-05	2.40E-05	1.63E-05	2.64E-05	8.60E-06	1.48E-05	2.01E-05	2.18E-05	1.36E-05	2.53E-05
b3198	2.95E-06	4.59E-06	5.78E-06	6.09E-06	7.56E-06	8.47E-06	8.89E-06	9.28E-06	9.64E-06	9.66E-06	9.82E-06	1.04E-05	1.13E-05	1.13E-05	1.18E-05	1.27E-05	1.31E-05	1.33E-05	2.51E-05	2.93E-05	4.88E-05	3.00E-05	2.40E-05	2.61E-05	2.72E-05	4.14E-05	2.33E-05	2.79E-05	2.59E-05	3.05E-05	4.80E-05	4.61E-05	2.77E-05	4.08E-05	3.50E-05	2.68E-05
b3199	1.57E-06	2.44E-06	3.08E-06	3.24E-06	4.02E-06	4.51E-06	4.73E-06	4.94E-06	5.13E-06	5.14E-06	5.22E-06	5.53E-06	6.01E-06	6.00E-06	6.30E-06	6.77E-06	6.95E-06	7.05E-06	1.75E-05	1.18E-05	1.56E-05	1.05E-05	1.05E-05	9.15E-06	1.38E-05	1.24E-05	9.76E-06	9.19E-06	1.24E-05	9.58E-06	1.03E-05	9.52E-06	8.58E-06	6.32E-06	1.12E-05	6.17E-06
b3200	1.46E-06	2.26E-06	2.85E-06	3.00E-06	3.73E-06	4.18E-06	4.39E-06	4.58E-06	4.76E-06	4.77E-06	4.84E-06	5.13E-06	5.58E-06	5.56E-06	5.84E-06	6.28E-06	6.44E-06	6.54E-06	0.000142521	0.000134729	0.000207523	0.000133175	0.000122586	0.000162735	0.00015665	0.000150688	0.000167317	0.000160867	0.000147874	0.000111054	0.000202928	0.000159057	0.000148602	0.000187678	0.000141911	0.0001756
b3201	1.94E-06	3.01E-06	3.80E-06	4.00E-06	4.97E-06	5.56E-06	5.84E-06	6.10E-06	6.33E-06	6.35E-06	6.45E-06	6.83E-06	7.43E-06	7.40E-06	7.78E-06	8.36E-06	8.58E-06	8.70E-06	0.000240329	0.000237216	0.000237585	0.000207556	0.000209922	0.000293962	0.000285119	0.000224958	0.000229073	0.00027963	0.000215596	0.00022071	0.000210443	0.000213751	0.000298833	0.000214047	0.000229945	0.000211959
b3208	1.44E-05	2.23E-05	2.81E-05	2.96E-05	3.67E-05	4.12E-05	4.32E-05	4.51E-05	4.69E-05	4.70E-05	4.77E-05	5.05E-05	5.49E-05	5.48E-05	5.76E-05	6.19E-05	6.35E-05	6.44E-05	2.74E-08	0	7.10E-08	4.02E-08	0	0	8.60E-08	2.28E-07	7.79E-08	2.89E-07	0	3.06E-08	8.50E-08	0	3.64E-08	4.31E-08	0	2.16E-08
b3212	0	0	0	0	0	0	0	0	0	0	0	0	0	0	0	0	0	0	0.002295296	0.002693308	0.002004045	0.00188026	0.002584953	0.002142107	0.001597071	0.001907911	0.002376639	0.002648216	0.002503543	0.002730272	0.000947823	0.0028451	0.003185331	0.002945022	0.004134224	0.003164931
b3213	0	0	0	0	0	0	0	0	0	0	0	0	0	0	0	0	0	0	0.000503119	0.000601118	0.000753002	0.000568208	0.000540385	0.000626521	0.000596277	0.000545388	0.000641181	0.000890322	0.000567713	0.000534826	0.000395124	0.000844767	0.0010521	0.001015523	0.001026439	0.000766375
b3222	0	0	0	0	0	0	0	0	0	0	0	0	0	0	0	0	0	0	5.67E-06	3.58E-06	1.71E-06	2.32E-06	3.93E-06	3.65E-06	3.49E-06	2.61E-06	2.00E-06	2.66E-06	4.24E-06	3.63E-06	1.42E-06	1.05E-06	1.35E-06	8.17E-07	4.69E-06	9.95E-07
b3223	0	0	0	0	0	0	0	0	0	0	0	0	0	0	0	0	0	0	8.57E-06	9.11E-06	1.03E-05	5.40E-06	8.65E-06	8.86E-06	9.70E-06	8.11E-06	8.32E-06	9.10E-06	1.14E-05	4.91E-06	2.39E-06	3.18E-06	3.64E-06	2.59E-06	4.02E-06	2.68E-06
b3224	0	0	0	0	0	0	0	0	0	0	0	0	0	0	0	0	0	0	0	0	0	0	0	0	0	0	0	0	0	0	0	0	0	0	0	0
b3225	0	0	0	0	0	0	0	0	0	0	0	0	0	0	0	0	0	0	2.00E-05	2.86E-05	1.15E-05	2.56E-05	3.78E-05	3.10E-05	4.18E-05	3.69E-05	3.75E-05	4.17E-05	4.70E-05	3.09E-05	5.46E-06	1.58E-05	1.81E-05	2.84E-06	1.78E-05	9.54E-06
b3236	1.26E-06	2.06E-06	2.70E-06	0.000173415	3.74E-06	4.32E-06	0.00011953	8.98E-05	5.10E-06	5.15E-06	5.23E-06	5.64E-06	6.32E-06	6.30E-06	6.74E-06	7.46E-06	7.75E-06	7.91E-06	0.013936246	0.014760032	0.014144358	0.032716514	0.015783846	0.032374593	0.017118418	0.01740435	0.015936691	0.015692628	0.021641604	0.016635864	0.014167093	0.015941442	0.011718917	0.007666485	0.016069486	0.013195524
b3240	0	0	0	0	0	0	0	0	0	0	0	0	0	0	0	0	0	0	0	0	0	0	0	0	0	0	0	0	0	0	0	0	0	0	0	0
b3241	0	0	0	0	0	0	0	0	0	0	0	0	0	0	0	0	0	0	0	0	0	0	0	0	0	0	0	0	0	0	0	0	0	0	0	0
b3248	0	0	0	0	0	0	0	0	0	0	0	0	0	0	0	0	0	0	6.01E-06	7.56E-06	9.91E-06	1.42E-05	8.66E-06	1.52E-05	1.54E-05	1.18E-05	1.59E-05	1.77E-05	1.14E-05	1.13E-05	2.40E-05	1.49E-05	3.00E-05	1.88E-05	1.38E-05	5.70E-05
b3253	0	0	0	0	0	0	0	0	0	0	0	0	0	0	0	0	0	0	0.000427166	0.000404311	0.000430648	0.000296787	0.000346096	0.000360561	0.000364754	0.000300378	0.000324237	0.000354907	0.000356142	0.000269049	0.000299283	0.000293968	0.000259683	0.000327503	0.000259485	0.000302938
b3255	4.39E-05	6.82E-05	8.59E-05	9.04E-05	0.00011229	0.000125807	0.000132046	0.000137829	0.000143216	0.000143519	0.000145867	0.000154394	0.000167937	0.000167433	0.000175939	0.000189059	0.000193965	0.000196798	0.000186161	0.000193981	0.000242586	0.000194432	0.0001983	0.000261588	0.00024866	0.000282995	0.000248289	0.000270046	0.0002547	0.000226978	0.000380561	0.000268523	0.00029274	0.00029679	0.000260443	0.000408438
b3256	0.000129684	0.000201445	0.000253815	0.000267176	0.000331884	0.000371834	0.000390274	0.000407366	0.000423289	0.000424184	0.000431125	0.000456328	0.000496355	0.000494865	0.000520006	0.000558782	0.000573282	0.000581657	0.000386431	0.000398268	0.000452194	0.000435837	0.00043422	0.000526421	0.000590625	0.000581353	0.000483664	0.000575416	0.000508055	0.000452744	0.000331072	0.000499446	0.000609028	0.000526416	0.000575378	0.000700586
b3258	0	0	0	0	0	0	0	0	0	0	0	0	0	0	0	0	0	0	0	0	0	0	0	0	0	0	0	0	0	0	0	0	0	0	0	0
b3265	0	0	0	0	0	0	0	0	0	0	0	0	0	0	0	0	0	0	0	0	0	0	0	0	0	0	0	0	0	0	0	0	0	0	0	0
b3266	0	0	0	0	0	0	0	0	0	0	0	0	0	0	0	0	0	0	0	0	0	0	0	0	0	0	0	0	0	0	0	0	0	0	0	0
b3281	2.30E-05	3.60E-05	4.57E-05	4.84E-05	6.05E-05	6.83E-05	7.18E-05	7.52E-05	7.84E-05	7.86E-05	7.99E-05	8.50E-05	9.31E-05	9.28E-05	9.80E-05	0.000106178	0.00010929	0.000111071	2.19E-05	2.28E-05	2.74E-05	3.11E-05	2.12E-05	2.63E-05	2.90E-05	2.23E-05	2.40E-05	3.37E-05	1.97E-05	2.17E-05	4.78E-05	3.31E-05	2.43E-05	4.21E-05	3.17E-05	2.49E-05
b3290	0	0	0	0	0	0	0	0	0	0	0	0	0	0	0	0	0	0	4.33E-05	3.92E-05	1.21E-05	2.15E-05	2.74E-05	1.42E-05	1.67E-05	9.65E-06	1.92E-05	1.61E-05	1.13E-05	2.44E-05	6.19E-06	9.75E-06	1.46E-05	9.69E-06	1.07E-05	2.14E-05
b3291	0	0	0	0	0	0	0	0	0	0	0	0	0	0	0	0	0	0	0.000284988	0.000266398	0.000222607	0.000137554	0.000202384	0.000126144	0.000149253	0.000122122	0.000262122	0.000170211	0.000159279	0.000193784	0.000188968	0.000199148	0.000143615	0.000193845	0.000131285	0.00015568
b3336	0	0	0	0	0	0	0	0	0	0	0	0	0	0	0	0	0	0	0.002354167	0.001590774	0.001580148	0.001285024	0.001146588	0.000424271	0.001141889	0.000667108	0.000938809	0.000768408	0.001018079	0.00067294	0.000204236	0.000280987	0.000430319	0.000536313	0.000357229	0.000454082
b3350	0	0	0	0	0	0	0	0	0	0	0	0	0	0	0	0	0	0	0	0	0	0	0	0	0	0	0	0	0	0	0	0	0	0	0	0
b3359	0	0	0	0	0	0	0	0	0	0	0	0	0	0	0	0	0	0	0.001186919	0.001078075	0.001107668	0.000572681	0.000814456	0.000842534	0.000593558	0.000666058	0.000840141	0.000735908	0.000642939	0.000869697	0.00066958	0.000636613	0.001169982	0.000966898	0.00084055	0.000969309
b3360	1.44E-06	2.29E-06	2.93E-06	3.11E-06	3.91E-06	4.44E-06	4.69E-06	4.92E-06	5.14E-06	5.16E-06	5.25E-06	5.60E-06	6.18E-06	6.16E-06	6.52E-06	7.11E-06	7.33E-06	7.46E-06	5.04E-06	9.43E-06	6.38E-06	5.34E-06	1.21E-05	1.73E-06	1.29E-05	2.97E-06	1.01E-05	1.62E-05	0	5.48E-06	1.60E-05	0	2.15E-05	1.45E-05	0	1.62E-05
b3365	0	0	0	0	0	0	0	0	0	0	0	0	0	0	0	0	0	0	2.08E-06	1.68E-06	1.90E-06	2.64E-06	1.72E-06	2.38E-06	1.99E-06	2.25E-06	2.80E-06	2.30E-06	3.15E-06	2.09E-06	2.68E-06	1.39E-06	1.93E-06	2.37E-06	3.11E-06	1.25E-05
b3366	0	0	0	0	0	0	0	0	0	0	0	0	0	0	0	0	0	0	3.63E-07	5.24E-07	5.18E-07	4.94E-07	2.80E-07	4.46E-07	9.53E-08	8.31E-07	2.84E-07	3.55E-07	1.39E-07	6.24E-07	3.91E-07	7.95E-07	8.22E-07	3.60E-07	1.29E-07	9.97E-07
b3367	0	0	0	0	0	0	0	0	0	0	0	0	0	0	0	0	0	0	0	0	0	0	0	0	0	0	0	0	0	0	0	0	0	0	0	0
b3368	9.32E-06	1.48E-05	1.89E-05	2.01E-05	2.53E-05	2.87E-05	3.03E-05	3.18E-05	3.32E-05	3.33E-05	3.39E-05	3.62E-05	3.99E-05	3.98E-05	4.21E-05	4.59E-05	4.74E-05	4.82E-05	1.60E-05	1.42E-05	1.69E-05	1.20E-05	1.24E-05	1.56E-05	1.66E-05	1.66E-05	1.57E-05	1.46E-05	1.64E-05	1.62E-05	1.51E-05	1.89E-05	1.01E-05	1.98E-05	1.74E-05	1.85E-05
b3370	0	0	0	0	0	0	0	0	0	0	0	0	0	0	0	0	0	0	0	0	0	0	0	0	0	0	0	0	0	0	0	0	0	0	0	0
b3371	0	0	0	0	0	0	0	0	0	0	0	0	0	0	0	0	0	0	1.12E-06	0	3.61E-08	3.20E-07	9.72E-08	6.83E-08	3.13E-08	1.92E-08	1.60E-07	4.98E-07	0	1.96E-06	0	0	2.18E-07	4.46E-06	0	1.11E-07
b3374	0	0	0	0	0	0	0	0	0	0	0	0	0	0	0	0	0	0	0	0	0	0	0	0	0	0	0	0	0	0	0	0	0	0	0	0
b3385	0	0	0	0	0	0	0	0	0	0	0	0	0	0	0	0	0	0	6.69E-05	8.03E-05	7.37E-05	9.58E-05	8.13E-05	0.000109984	0.000118919	8.97E-05	0.000104976	0.000116466	9.45E-05	9.25E-05	0.000140563	0.000109061	0.000137884	0.000128801	8.26E-05	0.000140092
b3386	6.90E-06	1.08E-05	1.36E-05	1.07E-06	1.79E-05	2.01E-05	1.59E-06	1.67E-06	2.30E-05	4.70E-05	2.35E-05	2.49E-05	2.72E-05	3.69E-06	2.86E-05	3.08E-05	3.17E-05	3.22E-05	1.71E-05	1.66E-05	2.85E-05	3.05E-05	2.12E-05	4.95E-05	4.35E-05	4.75E-05	7.14E-05	4.40E-05	0	3.20E-05	4.21E-05	0	8.77E-05	5.24E-05	0	7.27E-05
b3389	2.50E-05	3.92E-05	4.97E-05	5.26E-05	6.58E-05	7.42E-05	7.81E-05	8.18E-05	8.52E-05	8.55E-05	8.69E-05	9.24E-05	0.000101259	0.000100954	0.000106579	0.000115447	0.000118831	0.000120767	6.86E-05	7.76E-05	8.32E-05	6.98E-05	7.10E-05	8.17E-05	6.56E-05	7.23E-05	6.80E-05	7.16E-05	7.39E-05	5.88E-05	8.86E-05	7.49E-05	8.09E-05	8.54E-05	6.63E-05	0.000101872
b3390	0	0	0	0	0	0	0	0	0	0	0	0	0	0	0	0	0	0	9.30E-05	0.000107455	0.000138673	0.00014641	0.000121	0.000160318	0.000180112	0.000191013	0.000187351	0.000175561	0.000179591	0.000149591	0.000234427	0.000205833	0.000281268	0.000198136	0.000199156	0.000255195
b3396	5.16E-05	8.02E-05	0.000101015	0.000106333	0.000132087	0.000147987	0.000155327	0.00016213	0.000168467	0.000168822	0.000171585	0.000181616	0.000197549	0.000196956	0.000206961	0.000222382	0.000228153	0.000231488	2.84E-05	2.60E-05	1.69E-05	1.92E-05	2.43E-05	2.42E-05	2.06E-05	4.93E-05	2.72E-05	1.29E-05	3.80E-05	2.44E-05	5.94E-05	3.32E-05	1.97E-05	1.19E-05	5.79E-05	3.85E-05
b3397	0	0	0	0	0	0	0	0	0	0	0	0	0	0	0	0	0	0	4.47E-05	5.36E-05	6.44E-05	5.76E-05	5.75E-05	8.99E-05	7.58E-05	8.65E-05	7.42E-05	6.96E-05	7.53E-05	6.11E-05	8.90E-05	8.16E-05	8.08E-05	6.95E-05	6.73E-05	6.49E-05
b3399	0	0	0	0	0	0	0	0	0	0	0	0	0	0	0	0	0	0	4.14E-06	2.57E-06	4.48E-06	3.20E-06	2.33E-06	5.61E-06	1.41E-05	6.69E-06	1.24E-05	8.07E-06	0	4.53E-06	3.22E-06	0	1.07E-05	2.88E-06	0	5.55E-06
b3403	0	0	0	0.000559256	0	0	0.000851822	0.000895402	0	0	0	0	0	0	0	0	0	0	0.003345626	0.003512258	0.002825222	0.003576138	0.003796227	0.003983986	0.003801969	0.003754098	0.002635516	0.002321926	0.002938644	0.002384892	0.000494949	0.001185499	0.000466768	0.000399762	0.000947767	0.000436904

[illegible]

b3654	0	0	0	0	0	0	0	0	0	0	0	0	0	0	0	0	0	0	0
b3656	0	0	0	0	0	0	0	0	0	0	0	0	0	0	0	0	0	0	7.79E-07
b3662	0	0	0	0	0	0	0	0	0	0	0	0	0	0	0	0	0	0	0
b3664	0	0	0	0	0	0	0	0	0	0	0	0	0	0	0	0	0	0	0
b3665	0	0	0	0	0	0	0	0	0	0	0	0	0	0	0	0	0	0	7.25E-06
b3666	0	0	0	0	0	0	0	0	0	0	0	0	0	0	0	0	0	0	1.29E-05
b3670	4.90E-05	7.69E-05	9.75E-05	0.000103205	0.000129041	0.000145558	0.000153203	0.000160366	0.000167112	0.000167707	0.000170451	0.000181214	0.000198606	0.000198009	0.00020904	0.000226429	0.000233064	0.000236862	0.000273117
b3671	0.000266827	0.000418291	0.000530889	0.000561668	0.000702277	0.000792167	0.000833772	0.000872753	0.00090947	0.000912708	0.000927643	0.000986215	0.001080867	0.001077621	0.00113765	0.001232289	0.0012684	0.001289065	0.000676008
b3679	0	0	0	0	0	0	0	0	0	0	0	0	0	0	0	0	0	0	0
b3691	0	0	0	0	0	0	0	0	0	0	0	0	0	0	0	0	0	0	0
b3693	0	0	0	0	0	0	0	0	0	0	0	0	0	0	0	0	0	0	2.40E-06
b3697	0	0	0	0.00131566	0	0.006104246	0.002041565	0.002152412	0	0	0	0	0	0	0	0	0	0	2.07E-05
b3708	0	0	0	0	0	0	0	0	0	0	0	0	0	0	0	0	0	0	0.002012967
b3709	0	0	0	0	0	0	0	0	0	0	0	0	0	0	0	0	0	0	2.90E-05
b3713	0	0	0	0	0	0	0	0	0	0	0	0	0	0	0	0	0	0	0.000388719
b3714	0	0	0	0	0	1.69E-33	0	0	0	0	0	0	0	0	0	0	0	0	3.78E-05
b3715	0	0	0	0	0	0	0	0	0	0	0	0	0	0	0	0	0	0	5.53E-07
b3721	0	0	0	0	0	0	0	0	0	0	0	0	0	0	0	0	0	0	0
b3722	0	0	0	0	0	0	0	0	0	0	0	0	0	0	0	0	0	0	0
b3725	0	0	0	0	0	0	0	0	0	0	0	0	0	0	0	0	0	0	1.51E-05
b3726	0	0	0	0	0	0	0	0	0	0	0	0	0	0	0	0	0	0	0
b3727	0	0	0	0	0	0	0	0	0	0	0	0	0	0	0	0	0	0	0
b3728	0	0	0	0	0	0	0	0	0	0	0	0	0	0	0	0	0	0	7.53E-05
b3729	0.000222248	0.00034523	0.000434982	0.00045788	0.000568779	0.000637245	0.000668852	0.000698137	0	0.000726967	0.000738862	0.000782058	0.000850653	0.000848098	0.000891193	0.000957607	0.000982461	0.000996813	0.000951764
b3730	8.82E-05	0.000137033	0.000172658	0.000181747	0.000225766	0.000252942	0.000265488	0.000277112	0.000287945	0.000288555	0.000293277	0.000310423	0.00033765	0.000336636	0.000353742	0.000380104	0.000389969	0.000395666	0.000104776
b3731	0.000234714	0.000372025	0.000480418	0.001102244	0.000637219	0.000839991	0.000965164	0.001073224	0.000854157	0.001165534	0.000875098	0.000912198	0.001005994	0.001006585	0.001062765	0.00115822	0.001221263	0.001215969	0.000633684
b3732	0.000783903	0.0012425	0.001604512	0.003681304	0.002128202	0.002805425	0.003223479	0.003584382	0.002852737	0.003892683	0.002922675	0.003046583	0.003359845	0.003361819	0.00354945	0.003868255	0.004078807	0.004061126	0.005576809
b3733	0.000491872	0.000779626	0.001006775	0.00230989	0.001335373	0.001760306	0.002022621	0.002249075	0.001789993	0.002442523	0.001833877	0.001911624	0.002108186	0.002109424	0.002227156	0.002427195	0.002559309	0.002548215	0.00034573
b3734	0.000860177	0.001363395	0.00176063	0.004039493	0.002335276	0.003078391	0.003537122	0.003933141	0.003130307	0.004271439	0.00320705	0.003343014	0.003686757	0.003688923	0.003894809	0.004244634	0.004475673	0.004456272	0.004691145
b3735	0.000301132	0.0004773	0.000616365	0.001414153	0.000817538	0.001077689	0.001238282	0.001376921	0.001095864	0.001495353	0.00112273	0.001170328	0.001290667	0.001291425	0.001363502	0.001485969	0.001566852	0.00156006	0.000592189
b3736	0.000268915	0.000426235	0.000550422	0.001262857	0.000730072	0.000962391	0.001105803	0.001229609	0.000978621	0.00133537	0.001002613	0.001045119	0.001152582	0.00115326	0.001217625	0.00132699	0.001399219	0.001393154	0.000610273
b3737	0.000128603	0.000203838	0.000263228	0.000603936	0.000349142	0.000460244	0.000528828	0.000588036	0.000468006	0.000638614	0.00047948	0.000499807	0.0005512	0.000551523	0.000582305	0.000634607	0.000669149	0.000666248	1.16E-05
b3738	0.000472024	0.000748166	0.00096615	0.002216681	0.001281488	0.001689275	0.001941004	0.002158321	0.001717764	0.002343963	0.001759877	0.001834487	0.002023117	0.002024305	0.002137286	0.002329253	0.002456036	0.00244539	0.000402082
b3739	0	0	0	0	0	0	0	0	0	0	0	0	0	0	0	0	0	0	0
b3744	6.33E-05	9.93E-05	0.000126032	0.000133339	0.000166718	0.000188058	0.000197935	0.000207189	0.000215905	0.000216674	0.000220219	0.000234124	0.000256593	0.000255822	0.000270073	0.000292538	0.000301112	0.000306017	6.93E-05
b3747	0	0	0	0	0	0	0	0	0	0	0	0	0	0	0	0	0	0	0
b3748	0	0	0	0	0	0	0	0	0	0	0	0	0	0	0	0	0	0	0.000201615
b3749	0	0	0	0	0	0	0	0	0	0	0	0	0	0	0	0	0	0	9.71E-05
b3750	0	0	0	0	0	0	0	0	0	0	0	0	0	0	0	0	0	0	2.79E-05
b3751	0	0	0	0	0	0	0	0	0	0	0	0	0	0	0	0	0	0	0.006444498
b3752	0	0	0	0	0	0	0	0	0	0	0	0	0	0	0	0	0	0	4.58E-05
b3770	0.000236821	0.000371098	0.000470837	0.000494381	0.000622517	0.000701987	0.000738766	0.000773209	0.000805637	0.000808454	0.000821683	0.000873397	0.00095691	0.000954036	0.001006979	0.001090372	0.00112217	0.001140373	0.000276033
b3771	0.000191878	0.000300797	0.000381767	0.0004039	0.000505014	0.000569655	0.000599574	0.000627605	0.000654009	0.000656337	0.000667077	0.000709197	0.000777262	0.000774928	0.000818096	0.000886151	0.000912119	0.00092698	0.000185215
b3772	0	0	0	0	0	0	0	0	0	0	0	0	0	0	0	0	0	0	3.39E-05
b3774	0.001605902	0.002517492	0.003195162	0.003380406	0.004226665	0.004767673	0.005018077	0.005252682	0.005473668	0.005493153	0.005583038	0.005935557	0.006505227	0.00648569	0.006846978	0.007416563	0.0076339	0.007758278	0.003745521
b3779	0	0	0	0	0	0	0	0	0	0	0	0	0	0	0	0	0	0	1.14E-05
b3781	3.20E-06	5.07E-06	6.48E-06	6.90E-06	8.68E-06	9.85E-06	1.04E-05	1.09E-05	1.14E-05	1.14E-05	1.16E-05	1.24E-05	1.37E-05	1.36E-05	1.45E-05	1.58E-05	1.63E-05	1.65E-05	0.001235218
b3784	0	0	0	0	0	0	0	0	0	0	0	0	0	0	0	0	0	0	1.07E-06
b3785	0	0	0	0	0	0	0	0	0	0	0	0	0	0	0	0	0	0	4.41E-05
b3786	0	0	0	0	0	0	0	0	0	0	0	0	0	0	0	0	0	0	6.78E-06
b3787	0	0	0	0	0	0	0	0	0	0	0	0	0	0	0	0	0	0	7.04E-06
b3788	0	0	0	0	0	0	0	0	0	0	0	0	0	0	0	0	0	0	1.66E-06
b3789	0	0	0	0	0	0	0	0	0	0	0	0	0	0	0	0	0	0	2.79E-06
b3790	0	0	0	0	0	0	0	0	0	0	0	0	0	0	0	0	0	0	8.62E-07
b3791	0	0	0	0	0	0	0	0	0	0	0	0	0	0	0	0	0	0	1.18E-05
b3792	0	0	0	0	0	0	0	0	0	0	0	0	0	0	0	0	0	0	0
b3793	0	0	0	0	0	0	0	0	0	0	0	0	0	0	0	0	0	0	0
b3794	0	0	0	0	0	0	0	0	0	0	0	0	0	0	0	0	0	0	1.39E-06
b3804	7.48E-07	1.18E-06	1.52E-06	1.61E-06	2.03E-06	2.30E-06	2.43E-06	2.55E-06	2.66E-06	2.68E-06	2.72E-06	2.90E-06	3.20E-06	3.19E-06	3.38E-06	3.68E-06	3.80E-06	3.87E-06	3.96E-06
b3805	4.27E-06	6.76E-06	8.65E-06	9.20E-06	1.16E-05	1.31E-05	1.39E-05	1.46E-05	1.52E-05	1.53E-05	1.55E-05	1.66E-05	1.83E-05	1.82E-05	1.93E-05	2.10E-05	2.17E-05	2.21E-05	4.08E-05
b3806	0	0	0	0	0	0	0	0	0	0	0	0	0	0	0	0	0	0	9.28E-07
b3809	1.31E-05	2.05E-05	2.60E-05	2.75E-05	3.44E-05	3.87E-05	4.08E-05	4.26E-05	4.44E-05	4.46E-05	4.53E-05	4.82E-05	5.28E-05	5.26E-05	5.55E-05	6.01E-05	6.18E-05	6.28E-05	3.90E-05
b3812	3.53E-06	5.59E-06	7.15E-06	7.60E-06	9.56E-06	1.09E-05	1.15E-05	1.20E-05	1.26E-05	1.26E-05	1.28E-05	1.37E-05	1.51E-05	1.50E-05	1.59E-05	1.74E-05	1.79E-05	1.82E-05	1.57E-05
b3816	1.00E-08	1.59E-08	2.03E-08	2.16E-08	2.71E-08	3.08E-08	3.25E-08	3.41E-08	3.56E-08	3.58E-08	3.64E-08	3.88E-08	4.28E-08	4.27E-08	4.52E-08	4.93E-08	5.09E-08	5.18E-08	0.000244862
b3821	0	0	0	0	0	0	0	0	0	0	0	0	0	0	0	0	0	0	4.07E-06
b3823	0	0	0	0	0	0	0	0	0	0	0	0	0	0	0	0	0	-6.88E-35	0
b3824	0	0	0	0	0	0	0	0	0	0	0	0	0	0	0	0	0	0	0
b3825	0	0	0	0	0	0	0	0	0	0	0	0	0	0	0	0	0	0	0
b3826	0	0	0	0	0	0	0	0	0	0	0	0	0	0	0	0	0	0	0.000118963
b3829	0.007002487	0.010977879	0.013933341	0.014741429	0.018432194	0.020791968	0.021884183	0.022907448	0.023871454	0.023956609	0.024348611	0.02588629	0.028371374	0.028286168	0.029862254	0.032347049	0.033295379	0.033837761	0.003662041
b3831	5.64E-06	8.94E-06	1.14E-05	0	1.53E-05	1.74E-05	1.83E-05	1.92E-05	2.01E-05	2.02E-05	2.05E-05	2.19E-05	2.41E-05	2.41E-05	2.55E-05	2.78E-05	2.87E-05	2.92E-05	0.001714698
b3833	0	0	0	0	0	0	0	0	0	0	0	0	0	0	0	0	0	0	0.000153723
b3843	4.48E-07	7.10E-07	9.09E-07	9.66E-07	1.22E-06	1.38E-06	1.46E-06	1.53E-06	1.60E-06	1.60E-06	1.63E-06	1.74E-06	1.92E-06	1.91E-06	2.03E-06	2.21E-06	2.28E-06	2.32E-06	0.000271279
b3844	0	0	0	0	0	0	0	0	0	0	0	0	0	0	0	0	0	0	0.000137756
b3845	0	0	0	0	0	0	0	0	0	0	0	0	0	0	0	0	0	0	0.000535043
b3846	0	0	0	0	0	0	0	0	0	0	0	0	0	0	0	0	0	0	0.001622377
b3849	0	0	0	0	0	0	0	0	0	0	0	0	0	0	0	0	0	0	0
b3850	7.77E-08	1.23E-07	1.57E-07	1.67E-07	2.11E-07	2.39E-07	2.52E-07	2.65E-07	2.77E-07	2.78E-07	2.83E-07	3.01E-07	3.32E-07	3.31E-07	3.51E-07	3.83E-07	3.95E-07	4.02E-07	1.39E-05
b3856	0	0	0	0	0	0	0	0	0	0	0	0	0	0	0	0	0	0	8.06E-07
b3857	0	0	0	0	0	0	0	0	0	0	0	0	0	0	0	0	0	0	2.34E-06
b3860	0	0	0	0	0	0	0	0	0	0	0	0	0	0	0	0	0	0	0.000282931
b3867	0	0	0	0	0	0	0	0	0	0	0	0	0	0	0	0	0	0	2.23E-05
b3870	0.000502833	0.000826714	0.001085797	0.001174499	0.001508742	0.001747178	0.001858008	0.001964822	0.001878599	0.002086057	0.002120191	0.00228784	0.002569074	0.002561359	0.002742951	0.00304131	0.003159323	0.003225454	0.003701168
b3875	0	0	0	0	0	0	0	0	0	0	0	0	0	0	0	0	0	0	0
b3876	0	0	0	0	0	0	0	0	0	0	0	0	0	0	0	0	0	0	0
b3877	0	0	0	0	0	0	0	0	0	0	0	0	0	0	0	0	0	0	0

b4287	0	0	0	0	0	0	0	0	0	0	0	0
b4288	0	0	0	0	0	0	0	0	0	0	0	0
b4289	0	0	0	0	0	0	0	0	0	0	0	0
b4290	0	0	0	0	0	0	0	0	0	0	0	0
b4291	0	0	0	0	0	0	0	0	0	0	0	0
b4297	0	0	0	0	0	0	0	0	0	0	0	0
b4298	0	0	0	0	0	0	0	0	0	0	0	0
b4311	0	0	0	0	0	0	0	0	0	0	0	0
b4321	0	0	0	0	0	0	0	0	0	0	0	0
b4322	0	0	0	0	0	0	0	0	0	0	0	0
b4323	0	0	0	0	0	0	0	0	0	0	0	0
b4337	0	0	0	0	0	0	0	0	0	0	0	0
b4352	0	0	0	0	0	0	0	0	0	0	0	0
b4356	0	0	0	0	0	0	0	0	0	0	0	0
b4358	0	0	0	0	0	0	0	0	0	0	0	0
b4367	0	0	0	0	0	5.69E-33	0	0	0	0	0	0
b4374	2.97E-07	4.70E-07	6.02E-07	0	8.05E-07	9.14E-07	9.64E-07	1.01E-06	1.06E-06	1.06E-06	1.08E-06	1.15E-06
b4381	0	0	0	0	0	0	0	0	0	0	0	0
b4382	0	0	0	0	0	0	0	0	0	0	0	0
b4383	7.27E-06	1.22E-05	1.64E-05	1.79E-05	2.32E-05	2.72E-05	2.91E-05	3.09E-05	3.26E-05	3.30E-05	3.35E-05	3.64E-05
b4384	1.57E-06	2.49E-06	3.18E-06	1.88E-08	4.26E-06	4.83E-06	5.10E-06	5.35E-06	5.59E-06	5.62E-06	5.71E-06	6.09E-06
b4386	0	0	0	0	0	0	0	0	0	0	0	0
b4388	1.21E-06	1.92E-06	2.46E-06	2.62E-06	3.30E-06	3.75E-06	3.96E-06	4.16E-06	4.34E-06	4.37E-06	4.44E-06	4.74E-06
b4390	0	0	0	0	0	0	0	0	0	0	0	0
b4392	0	0	0	0	0	0	0	0	0	0	0	0
b4394	0	0	0	0	0	0	0	0	0	0	0	0
b4407	2.39E-06	3.79E-06	4.85E-06	5.16E-06	6.49E-06	7.37E-06	7.78E-06	8.16E-06	8.53E-06	8.57E-06	8.71E-06	9.29E-06
b4460	0	0	0	0	0	0	0	0	0	0	0	0
b4464	0	0	0	0	0	0	0	0	0	0	0	0
b4467	0	0	0	0	0	0	0	0	0	0	0	0
b4468	0	0	0	0	0	0	0	0	0	0	0	0
b4471	0	0	0	0	0	0	0	0	0	0	0	0
b4474	0	0	0	0	0	0	0	0	0	0	0	0
b4476	0	0	0	0	0	0	0	0	0	0	0	0
b4477	0	0	0	0	0	0	0	0	0	0	0	0
b4478	0	0	0	0	0	0	0	0	0	0	0	0
b4481	0	0	0	0	0	0	0	0	0	0	0	0
b4485	0	0	0	0	0	0	0	0	0	0	0	0
b4513	0	0	0	0	0	0	0	0	0	0	0	0
b4515	0	0	0	0	0	0	0	0	0	0	0	0
b4544	0	0	0	0	0	0	0	0	0	0	0	0

b4287	0	0	0	0	0	0	6.85E-07	1.20E-06	3.45E-07	4.15E-07	2.82E-07	1.05E-06
b4288	0	0	0	0	0	0	0	0	0	0	0	0
b4289	0	0	0	0	0	0	0	0	0	0	0	0
b4290	0	0	0	0	0	0	2.61E-06	2.68E-06	7.83E-07	1.15E-06	1.69E-06	1.80E-06
b4291	0	0	0	0	0	0	9.04E-07	8.68E-07	2.42E-06	2.96E-06	1.44E-06	2.66E-06
b4297	0	0	0	0	0	0	0	0	0	0	0	0
b4298	0	0	0	0	0	0	4.28E-06	4.23E-06	7.03E-07	4.39E-06	1.10E-05	4.19E-06
b4311	0	0	0	0	0	0	0	0	0	0	0	0
b4321	0	0	0	0	0	0	0	0	0	0	0	0
b4322	0	0	0	0	0	0	0.000236872	0.000241616	0.000164213	0.000131613	0.00017404	0.000115501
b4323	0	0	0	0	0	0	2.04E-05	2.75E-05	2.12E-05	1.41E-05	2.11E-05	1.20E-05
b4337	0	0	0	0	0	0	0	0	0	0	0	0
b4352	0	0	0	0	0	0	3.70E-05	4.85E-05	5.14E-05	6.30E-05	4.82E-05	0.000149417
b4356	0	0	0	0	0	0	0	0	0	0	0	0
b4358	0	0	0	0	0	0	0	0	0	0	0	0
b4367	0	0	0	0	0	0	2.99E-07	2.00E-07	2.23E-06	1.64E-07	1.02E-07	4.21E-07
b4374	1.27E-06	1.27E-06	1.34E-06	1.46E-06	1.51E-06	1.54E-06	1.80E-06	2.23E-06	4.12E-06	1.91E-06	2.54E-06	5.84E-06
b4381	0	0	0	0	0	0	0.000252696	0.000208768	0.000212355	0.000200315	0.00017885	0.000167867
b4382	0	0	0	0	0	0	6.50E-05	5.19E-05	5.09E-05	3.79E-05	4.43E-05	4.47E-05
b4383	4.13E-05	4.11E-05	4.43E-05	4.96E-05	5.17E-05	5.28E-05	0.000647238	0.000591184	0.000668795	0.000497306	0.000505381	0.000353342
b4384	6.72E-06	6.70E-06	7.10E-06	7.73E-06	7.98E-06	8.12E-06	0.000571298	0.000609263	0.000624047	0.000491277	0.000564168	0.000440066
b4386	0	0	0	0	0	0	1.21E-05	1.40E-05	2.16E-05	1.50E-05	1.70E-05	2.21E-05
b4388	5.23E-06	5.21E-06	5.53E-06	6.03E-06	6.22E-06	6.33E-06	1.48E-05	2.23E-05	2.14E-05	1.35E-05	2.23E-05	2.46E-05
b4390	0	0	0	0	0	0	1.05E-05	9.11E-06	8.93E-06	3.51E-06	7.66E-06	6.02E-06
b4392	0	0	0	0	0	0	4.96E-05	3.74E-05	4.82E-05	4.48E-05	3.36E-05	5.88E-05
b4394	0	0	0	0	0	0	2.55E-05	2.66E-05	3.42E-05	3.61E-05	3.38E-05	2.90E-05
b4407	1.02E-05	1.02E-05	1.08E-05	1.18E-05	1.22E-05	1.24E-05	0	0	0	0	0	0
b4460	0	0	0	0	0	0	0	0	0	0	0	0
b4464	0	0	0	0	0	0	0	0	0	0	0	0
b4467	0	0	0	0	0	0	5.79E-06	3.50E-06	6.05E-07	1.22E-05	3.12E-06	2.70E-09
b4468	0	0	0	0	0	0	2.17E-09	2.20E-09	2.21E-09	7.33E-06	2.22E-09	2.22E-09
b4471	0	0	0	0	0	0	0	0	0	0	0	0
b4474	0	0	0	0	0	0	0	0	0	0	0	0
b4476	0	0	0	0	0	0	0	0	0	0	0	0
b4477	0	0	0	0	0	0	2.74E-09	2.79E-09	1.51E-05	2.83E-09	2.77E-09	2.79E-09
b4478	0	0	0	0	0	0	4.34E-06	6.03E-06	0.000251994	1.42E-06	2.70E-06	1.12E-06
b4481	0	0	0	0	0	0	1.58E-06	1.32E-06	1.61E-06	1.08E-06	1.10E-06	1.52E-06
b4485	0	0	0	0	0	0	7.29E-06	1.86E-06	1.97E-07	6.08E-07	9.89E-07	2.86E-07
b4513	0	0	0	0	0	0	0	0	0	0	0	0
b4515	0	0	0	0	0	0	0	0	0	0	0	0
b4544	0	0	0	0	0	0	0	0	0	0	0	0

b4287	1.37E-06	9.74E-07	1.58E-06	1.26E-06	0	1.08E-06	6.35E-07	0	2.65E-06	1.88E-06	0	6.68E-06
b4288	0	0	0	0	0	0	0	0	0	0	0	0
b4289	0	0	0	0	0	0	0	0	0	0	0	0
b4290	1.27E-06	9.00E-07	2.74E-06	6.71E-07	0	1.22E-06	1.52E-06	0	2.47E-06	2.15E-06	0	3.27E-06
b4291	1.25E-06	2.39E-06	1.10E-06	1.22E-06	2.85E-06	7.58E-07	7.02E-06	3.77E-06	9.11E-07	8.42E-06	6.71E-07	7.96E-06
b4297	0	0	0	0	0	0	0	0	0	0	0	0
b4298	2.11E-07	4.19E-06	2.80E-06	3.07E-06	0	4.36E-06	6.21E-07	0	5.86E-06	1.28E-07	0	1.09E-07
b4311	0	0	0	0	0	0	0	0	0	0	0	0
b4321	0	0	0	0	0	0	0	0	0	0	0	0
b4322	0.000173742	0.000128589	0.000147736	0.000162867	0.000191885	0.000134638	7.73E-05	8.95E-05	0.000120745	9.39E-05	0.000105774	8.56E-05
b4323	1.97E-05	1.91E-05	1.62E-05	1.59E-05	3.50E-05	1.27E-05	4.19E-06	6.06E-06	7.13E-06	3.18E-06	9.53E-06	8.71E-06
b4337	0	0	0	0	0	0	0	0	0	0	0	0
b4352	5.68E-05	6.19E-05	6.63E-05	7.06E-05	6.06E-05	4.96E-05	4.27E-05	5.06E-05	4.01E-05	4.49E-05	5.09E-05	5.16E-05
b4356	0	0	0	0	0	0	0	0	0	0	0	0
b4358	0	0	0	0	0	0	0	0	0	0	0	0
b4367	5.14E-07	3.29E-06	5.99E-07	1.38E-06	1.99E-06	6.49E-07	1.61E-05	9.27E-06	1.09E-07	1.05E-05	2.62E-06	4.81E-06
b4374	6.08E-06	4.92E-06	3.88E-06	6.16E-06	4.40E-06	5.69E-06	3.97E-06	5.60E-06	1.13E-05	3.85E-06	5.79E-06	9.43E-06
b4381	0.000174099	0.000146585	0.000221375	0.000170647	0.000185363	0.00017682	0.000436802	0.000281849	0.000219791	0.000505347	0.000167552	0.000365002
b4382	4.58E-05	4.79E-05	5.26E-05	3.22E-05	5.03E-05	3.53E-05	9.53E-05	6.03E-05	4.35E-05	8.55E-05	3.82E-05	6.51E-05
b4383	0.000464473	0.000420227	0.000402396	0.000387051	0.000450592	0.000423963	0.000722321	0.000442204	0.000306346	0.000854008	0.000353791	0.000593845
b4384	0.000613777	0.000636	0.000514474	0.000647136	0.000595182	0.000568545	0.000519096	0.000521836	0.000489024	0.000498658	0.000549412	0.000532385
b4386	1.90E-05	2.32E-05	2.57E-05	2.07E-05	1.85E-05	1.79E-05	2.64E-05	2.44E-05	1.61E-05	1.24E-05	1.91E-05	1.60E-05
b4388	2.09E-05	2.27E-05	3.01E-05	3.10E-05	2.64E-05	2.18E-05	2.89E-05	3.45E-05	4.50E-05	2.50E-05	3.31E-05	1.43E-05
b4390	4.99E-06	5.03E-06	6.78E-06	6.11E-06	5.71E-06	7.93E-06	3.63E-06	5.29E-06	5.07E-06	4.42E-06	5.71E-06	4.60E-06
b4392	5.42E-05	4.10E-05	3.26E-05	5.91E-05	4.72E-05	3.10E-05	4.92E-05	4.29E-05	4.01E-05	3.02E-05	3.49E-05	3.95E-05
b4394	4.54E-05	3.89E-05	5.40E-05	3.83E-05	0	3.64E-05	3.63E-05	0	5.16E-05	5.43E-05	0	4.54E-05
b4407	0	0	0	0	0	0	0	0	0	0	0	0
b4460	0	0	0	0	0	0	0	0	0	0	0	0
b4464	0	0	0	0	0	0	0	0	0	0	0	0
b4467	2.20E-06	6.22E-07	1.41E-06	1.16E-06	3.20E-07	5.20E-07	1.95E-09	1.97E-09	8.20E-08	2.31E-08	1.04E-07	1.55E-08
b4468	2.06E-07	2.24E-09	1.96E-08	2.23E-09	2.26E-09	2.25E-09	2.24E-09	2.25E-09	2.24E-09	2.22E-09	2.22E-09	2.22E-09
b4471	0	0	0	0	0	0	0	0	0	0	0	0
b4474	0	0	0	0	0	0	0	0	0	0	0	0
b4476	0	0	0	0	0	0	0	0	0	0	0	0
b4477	4.89E-08	2.84E-09	2.79E-09	2.90E-09	2.86E-09	2.80E-09	2.82E-09	2.84E-09	2.82E-09	2.79E-09	2.80E-09	2.82E-09
b4478	1.61E-06	3.72E-06	2.34E-06	1.76E-06	4.66E-06	1.41E-06	9.77E-07	2.44E-06	2.71E-06	1.89E-06	3.34E-06	1.98E-06
b4481	1.39E-06	1.76E-06	1.80E-06	2.05E-06	1.78E-06	1.56E-06	1.51E-06	2.79E-06	1.37E-06	1.76E-06	2.58E-06	3.71E-06
b4485	9.22E-07	2.38E-07	1.73E-07	7.50E-07	1.37E-08	7.69E-07	2.11E-09	2.62E-08	1.27E-07	4.90E-07	1.67E-08	1.50E-07
b4513	0	0	0	0	0	0	0	0	0	0	0	0
b4515	0	0	0	0	0	0	0	0	0	0	0	0
b4544	0	0	0	0	0	0	0	0	0	0	0	0

Table S4. Proteome efficiency of pathways on all carbon (glycolytic and gluconeogenic) sources.

Pathway		Pathway expression (for *shared* proteins) (n = 18)[a]			*measured-only* fraction[b] (median across 18 conditions) ($f_{measured\text{-}only}$)	individual *shared* proteins; median across 18 conditions[c]		
		$r_{pathway}^2$	p	$GMFE_{pathway}$		$r_{individual}^2$	p	n[d]
Measures (1)-(4)		(1)	(1)	(2)	(3)	(4)	(4)	
**Transporters**		**(-) 0.49**[e]	**1.2×10^{-03}**	**3.306**	**0.997**	**0.1**	**0.688**	**4**
**Central metabolism**		**0.50**	9.6×10^{-04}	**2.394**	**0.306**	**0.15**	**3.2×10^{-03}**	**56**
	Glycolysis	0.75	4.0×10^{-06}	3.437	0.081	0.3	0.084	11
	Pentose phosphate pathway	0.19	0.074	1.922	0.384	0.3	0.338	5
	TCA cycle	0.35	0.01	5.795	0.096	0.38	0.033	12
	Glyoxylate shunt	1	-	nan	1	1	1	2
	Energy generation	(-) 0.004[e]	0.801	1.571	0.052	0.13	0.058	28
	Central metabolism others	0.68	2.5×10^{-05}	1.506	0.549	0.99	0.058	3
**Biosynthesis**		**0.78**	**1.2×10^{-06}**	**1.769**	**0.265**	**0.44**	**1.7×10^{-30}**	**229**
	Amino acid	0.76	2.9×10^{-06}	1.386	0.31	0.45	8.6×10^{-11}	72
	Nucleotide	0.58	2.6×10^{-04}	3.422	0.231	0.15	0.045	28
	Cell envelope	0.38	6.3×10^{-03}	1.809	0.138	0.38	2.3×10^{-05}	40
	Cofactor	0.84	1.2×10^{-07}	1.232	0.114	0.62	5.5×10^{-16}	70
	Biosynthesis others	0.12	0.155	3.877	0.255	0.44	8.5×10^{-05}	29
**Translation**		**0.87**	**1.2×10^{-03}**	**1.35**	**0**	**0.99**	**0.063**	**3**
**Others (other metabolic enzymes)**		**0.005**	**0.781**	**1.619**	**0.917**	**0.14**	**0.045**	**30**
**Total metabolism**		**0.50**	**9.6×10^{-04}**	**1.807**	**0.52**	**0.35**	**3.4×10^{-30}**	**308**

[a] Values reflect the local optimality of complete pathways across conditions. n = 18 indicates the number of all carbon (glycolytic and gluconeogenic) sources analyzed.

[b] Mass fraction of *measured-only* (un-predicted but observed) proteins relative to all proteins in the pathway.

[c] These columns reflect the local optimality compared across individual proteins within each pathway at a given growth condition; values are medians across the n = 18 growth conditions.

[d] Number of proteins in each pathway or pathway set.

[e] Negative correlation coefficient $r_{pathway}$.

Table S5: Data source for pathway annotation

Pathway		Source or notes
**Biosynthesis**		Ecocyc pathway ontology: "Biosynthesis" in the first hierarchy
	Amino acid	Ecocyc pathway ontology: "Amino Acid Biosynthesis" in the secondary hierarchy
	Nucleotide	Ecocyc pathway ontology: "Nucleoside and Nucleotide Biosynthesis" in the secondary hierarchy excluding the "Purine Nucleotide Salvage" and "Pyrimidine Nucleotide Salvage" in the fourth hierarchy
	Cell envelope	Ecocyc pathway ontology: "Fatty Acid and Lipid Biosynthesis" and "Cell Structure Biosynthesis" in the secondary hierarchy
	Cofactor	Ecocyc pathway ontology: "Cofactor, Carrier, and Vitamin Biosynthesis" in the secondary hierarchy
	Biosynthesis_others	Genes in ecocyc pathway ontology: "Biosynthesis" in the first hierarchy but are not in the mentioned 4 pathways
**Precursors and energy generation**		Ecocyc pathway ontology: "Generation of Precursor Metabolites and Energy" in the first hierarchy
	Glycolysis	Ecocyc pathway ontology: "Glycolysis" in the secondary hierarchy
	Pentose phosphate pathway	Ecocyc pathway ontology: "Pentose Phosphate Pathways" in the secondary hierarchy
	TCA cycle	Ecocyc pathway ontology: "TCA cycle" in the secondary hierarchy
	Glyoxylate shunt	Gene aceA, aceB, and glcB (added manually)
	Energy	Ecocyc pathway ontology: "Electron Transfer Chains" and "ATP biosynthesis" in the secondary hierarchy
	Precursors_energy_others	Genes in ecocyc pathway ontology: "Generation of Precursor Metabolites and Energy" in the first hierarchy but are not in the mentioned 5 pathways
**Transport**		iML1515 annotation (genes that transport metabolites across both inner and outer membrane)
**Others**		Genes in iML1515 model but are not in all the mentioned pathways

Table S6: Pathway membership of genes

Gene locus	Biosynthesis					Central metabolism						Transporters	Others (other metabolic enzymes)
	Amino acid	Nucleotide	Cell envelope	Cofactor	Biosynthesis others	Glycolysis	Pentose phosphate pathway	TCA cycle	Glyoxylate shunt	Energy generation	Central metabolism others		
b0002	Yes	No	No	No	No	No	No	No	No	No	No	No	No
b0003	Yes	No	No	No	No	No	No	No	No	No	No	No	No
b0004	Yes	No	No	No	No	No	No	No	No	No	No	No	No
b0007	No	No	No	No	No	No	No	No	No	No	No	Yes	No
b0008	No	No	No	No	No	No	Yes	No	No	No	No	No	No
b0009	No	No	No	Yes	No	No	No	No	No	No	No	No	No
b0010	No	No	No	No	No	No	No	No	No	No	No	Yes	No
b0019	No	No	No	No	No	No	No	No	No	No	No	Yes	No
b0025	No	No	No	Yes	No	No	No	No	No	No	No	No	No
b0029	No	No	No	No	Yes	No	No	No	No	No	No	No	No
b0030	No	No	No	No	Yes	No	No	No	No	No	No	No	No
b0031	Yes	No	No	No	No	No	No	No	No	No	No	No	No
b0032	Yes	Yes	No	No	No	No	No	No	No	No	No	No	No
b0033	Yes	Yes	No	No	No	No	No	No	No	No	No	No	No
b0036	No	No	No	No	No	No	No	No	No	No	No	No	Yes
b0037	No	No	No	No	No	No	No	No	No	No	No	No	Yes
b0038	No	No	No	No	No	No	No	No	No	No	No	No	Yes
b0040	No	No	No	No	No	No	No	No	No	No	No	Yes	No
b0046	No	No	No	No	No	No	No	No	No	No	No	No	Yes
b0047	No	No	No	No	No	No	No	No	No	No	No	Yes	No
b0048	No	No	No	Yes	No	No	No	No	No	No	No	No	No
b0049	No	No	No	No	No	No	No	No	No	No	No	No	Yes
b0052	No	No	No	Yes	No	No	No	No	No	No	No	No	No
b0054	No	No	No	No	No	No	No	No	No	No	No	No	Yes
b0061	No	No	No	No	No	No	No	No	No	No	No	No	Yes
b0062	No	No	No	No	No	No	No	No	No	No	No	No	Yes
b0063	No	No	No	No	No	No	No	No	No	No	No	No	Yes
b0066	No	No	No	No	No	No	No	No	No	No	No	Yes	No
b0067	No	No	No	No	No	No	No	No	No	No	No	Yes	No
b0068	No	No	No	No	No	No	No	No	No	No	No	Yes	No
b0070	No	No	No	No	No	No	No	No	No	No	No	Yes	No
b0071	Yes	No	No	No	No	No	No	No	No	No	No	No	No
b0072	Yes	No	No	No	No	No	No	No	No	No	No	No	No
b0073	Yes	No	No	No	No	No	No	No	No	No	No	No	No
b0074	Yes	No	No	No	No	No	No	No	No	No	No	No	No
b0077	Yes	No	No	No	No	No	No	No	No	No	No	No	No
b0078	Yes	No	No	No	No	No	No	No	No	No	No	No	No
b0084	No	No	Yes	No	No	No	No	No	No	No	No	No	No
b0085	No	No	Yes	No	No	No	No	No	No	No	No	No	No
b0086	No	No	Yes	No	No	No	No	No	No	No	No	No	No
b0087	No	No	Yes	No	No	No	No	No	No	No	No	No	No
b0088	No	No	Yes	No	No	No	No	No	No	No	No	No	No
b0089	No	No	No	No	No	No	No	No	No	No	No	No	Yes
b0090	No	No	Yes	No	No	No	No	No	No	No	No	No	No
b0091	No	No	Yes	No	No	No	No	No	No	No	No	No	No
b0092	No	No	Yes	No	No	No	No	No	No	No	No	No	No
b0096	No	No	Yes	No	No	No	No	No	No	No	No	No	No
b0099	No	No	No	No	No	No	No	No	No	No	No	No	Yes
b0103	No	No	No	Yes	No	No	No	No	No	No	No	No	No
b0104	No	No	No	No	No	No	No	No	No	No	No	No	Yes
b0109	No	No	No	Yes	No	No	No	No	No	No	No	No	No
b0110	No	No	No	No	No	No	No	No	No	No	No	No	Yes
b0112	No	No	No	No	No	No	No	No	No	No	No	Yes	No
b0114	No	No	No	No	No	No	No	No	No	No	Yes	No	No
b0115	No	No	No	No	No	No	No	No	No	No	Yes	No	No
b0116	No	No	No	Yes	No	No	No	Yes	No	No	No	No	No
b0118	No	No	No	No	No	No	No	Yes	No	No	No	No	No
b0120	No	No	No	No	Yes	No	No	No	No	No	No	No	No
b0121	No	No	No	No	Yes	No	No	No	No	No	No	No	No
b0123	No	No	No	No	No	No	No	No	No	No	No	Yes	No
b0124	No	No	No	No	No	No	No	No	No	No	No	No	Yes
b0125	No	No	No	No	Yes	No	No	No	No	No	No	No	No
b0126	No	No	No	No	No	No	No	No	No	No	No	No	Yes
b0131	Yes	No	No	Yes	No	No	No	No	No	No	No	No	No
b0133	No	No	No	Yes	No	No	No	No	No	No	No	No	No
b0134	No	No	No	Yes	No	No	No	No	No	No	No	No	No
b0142	No	No	No	Yes	No	No	No	No	No	No	No	No	No
b0149	No	No	Yes	No	No	No	No	No	No	No	No	No	No
b0150	No	No	No	No	No	No	No	No	No	No	No	Yes	No
b0151	No	No	No	No	No	No	No	No	No	No	No	Yes	No
b0152	No	No	No	No	No	No	No	No	No	No	No	Yes	No
b0153	No	No	No	No	No	No	No	No	No	No	No	Yes	No
b0154	No	No	No	No	Yes	No	No	No	No	No	No	No	No
b0155	No	No	No	No	No	No	No	No	No	No	No	Yes	No
b0158	No	No	No	No	No	No	No	No	No	No	No	Yes	No
b0159	No	No	No	Yes	No	No	No	No	No	No	No	No	No
b0160	No	No	No	No	No	No	No	No	No	No	No	No	Yes
b0166	Yes	No	No	No	No	No	No	No	No	No	No	No	No
b0171	No	Yes	No	No	No	No	No	No	No	No	No	No	No
b0173	No	No	No	No	Yes	No	No	No	No	No	No	No	No
b0174	No	No	No	Yes	No	No	No	No	No	No	No	No	No
b0175	No	No	Yes	No	No	No	No	No	No	No	No	No	No
b0179	No	No	Yes	No	No	No	No	No	No	No	No	No	No
b0180	No	No	Yes	Yes	No	No	No	No	No	No	No	No	No
b0181	No	No	Yes	No	No	No	No	No	No	No	No	No	No

b0182	No	No	Yes	No	No	No	No	No	No	No	No	No	No
b0185	No	No	Yes	No	No	No	No	No	No	No	No	No	No
b0186	No	No	No	No	Yes	No	No	No	No	No	No	No	No
b0197	No	No	No	No	No	No	No	No	No	No	No	Yes	No
b0198	No	No	No	No	No	No	No	No	No	No	No	Yes	No
b0199	No	No	No	No	No	No	No	No	No	No	No	Yes	No
b0200	No	No	No	No	Yes	No	No	No	No	No	No	No	No
b0207	No	No	No	No	No	No	No	No	No	No	No	No	Yes
b0211	No	No	No	No	No	No	No	No	No	No	No	No	Yes
b0212	No	No	No	No	No	No	No	No	No	No	No	No	Yes
b0221	No	No	No	No	No	No	No	No	No	No	No	No	Yes
b0222	No	No	No	No	Yes	No	No	No	No	No	No	No	No
b0237	No	No	No	No	No	No	No	No	No	No	No	No	Yes
b0238	No	No	No	No	Yes	No	No	No	No	No	No	No	No
b0241	No	No	No	No	No	No	No	No	No	No	No	Yes	No
b0242	Yes	No	No	No	No	No	No	No	No	No	No	No	No
b0243	Yes	No	No	No	No	No	No	No	No	No	No	No	No
b0260	No	No	No	No	No	No	No	No	No	No	No	Yes	No
b0261	No	No	No	No	No	No	No	No	No	No	No	No	Yes
b0268	No	No	No	No	No	No	No	No	No	No	No	No	Yes
b0269	No	No	No	No	No	No	No	No	No	No	No	No	Yes
b0273	Yes	No	No	No	No	No	No	No	No	No	No	No	No
b0306	No	No	No	No	No	No	No	No	No	No	No	No	Yes
b0307	No	No	No	No	No	No	No	No	No	No	No	No	Yes
b0308	No	No	No	No	No	No	No	No	No	No	No	No	Yes
b0311	No	No	No	No	Yes	No	No	No	No	No	No	No	No
b0312	No	No	No	No	Yes	No	No	No	No	No	No	No	No
b0314	No	No	No	No	No	No	No	No	No	No	No	Yes	No
b0315	No	No	No	No	No	No	No	No	No	No	No	No	Yes
b0325	No	No	No	No	No	No	No	No	No	No	No	No	Yes
b0331	No	No	No	No	No	No	No	No	No	No	No	No	Yes
b0333	No	No	No	No	No	No	No	No	No	No	No	No	Yes
b0334	No	No	No	No	No	No	No	No	No	No	No	No	Yes
b0335	No	No	No	No	No	No	No	No	No	No	No	No	Yes
b0336	No	No	No	No	No	No	No	No	No	No	No	Yes	No
b0337	No	No	No	No	Yes	No	No	No	No	No	No	No	No
b0339	No	No	No	No	No	No	No	No	No	No	No	No	Yes
b0340	No	No	No	No	No	No	No	No	No	No	No	No	Yes
b0341	No	No	No	No	No	No	No	No	No	No	No	Yes	No
b0343	No	No	No	No	No	No	No	No	No	No	No	Yes	No
b0344	No	No	No	No	No	No	No	No	No	No	No	No	Yes
b0347	No	No	No	No	No	No	No	No	No	No	No	No	Yes
b0348	No	No	No	No	No	No	No	No	No	No	No	No	Yes
b0349	No	No	No	No	No	No	No	No	No	No	No	No	Yes
b0350	No	No	No	No	No	No	No	No	No	No	No	No	Yes
b0351	No	No	No	No	No	No	No	No	No	No	No	No	Yes
b0352	No	No	No	No	No	No	No	No	No	No	No	No	Yes
b0353	No	No	No	No	No	No	No	No	No	No	No	Yes	No
b0355	No	No	No	No	No	No	No	No	No	No	No	No	Yes
b0356	No	No	No	No	No	No	No	No	No	No	No	No	Yes
b0365	No	No	No	No	No	No	No	No	No	No	No	Yes	No
b0366	No	No	No	No	No	No	No	No	No	No	No	Yes	No
b0367	No	No	No	No	No	No	No	No	No	No	No	Yes	No
b0368	No	No	No	No	No	No	No	No	No	No	No	No	Yes
b0369	No	No	No	No	Yes	No	No	No	No	No	No	No	No
b0376	No	No	Yes	No	No	No	No	No	No	No	No	No	No
b0381	No	No	Yes	No	No	No	No	No	No	No	No	No	No
b0383	No	No	No	No	No	Yes	No	No	No	No	No	No	No
b0386	Yes	No	No	No	No	No	No	No	No	No	No	No	No
b0388	Yes	No	No	No	No	No	No	No	No	No	No	No	No
b0394	No	No	No	No	No	No	No	No	No	No	No	No	Yes
b0401	No	No	No	No	No	No	No	No	No	No	No	Yes	No
b0402	No	No	No	No	No	No	No	No	No	No	No	Yes	No
b0403	No	No	No	No	No	No	No	No	No	No	No	No	Yes
b0404	No	No	No	Yes	No	No	No	No	No	No	No	No	No
b0411	No	No	No	No	No	No	No	No	No	No	No	Yes	No
b0414	No	No	No	Yes	No	No	No	No	No	No	No	No	No
b0415	No	No	No	Yes	No	No	No	No	No	No	No	No	No
b0417	No	No	No	Yes	No	No	No	No	No	No	No	No	No
b0418	No	No	Yes	No	No	No	No	No	No	No	No	No	No
b0420	No	No	No	Yes	No	No	No	No	No	No	No	No	No
b0421	No	No	No	Yes	No	No	No	No	No	No	No	No	No
b0423	No	No	No	Yes	No	No	No	No	No	No	No	No	No
b0425	No	No	No	Yes	No	No	No	No	No	No	No	No	No
b0428	No	No	No	No	No	No	No	No	No	No	No	No	Yes
b0429	No	No	No	No	No	No	No	No	No	Yes	No	No	No
b0430	No	No	No	No	No	No	No	No	No	Yes	No	No	No
b0431	No	No	No	No	No	No	No	No	No	Yes	No	No	No
b0432	No	No	No	No	No	No	No	No	No	Yes	No	No	No
b0433	No	No	No	No	No	No	No	No	No	No	No	No	Yes
b0444	No	No	No	No	Yes	No	No	No	No	No	No	No	No
b0446	No	No	No	No	No	No	No	No	No	No	No	No	Yes
b0451	No	No	No	No	No	No	No	No	No	No	No	Yes	No
b0452	No	No	No	No	No	No	No	No	No	No	No	No	Yes
b0459	No	No	No	No	No	No	No	No	No	No	No	No	Yes
b0462	No	No	No	No	No	No	No	No	No	No	No	Yes	No
b0463	No	No	No	No	No	No	No	No	No	No	No	Yes	No
b0465	No	No	No	No	No	No	No	No	No	No	No	Yes	No
b0469	No	No	No	No	Yes	No	No	No	No	No	No	No	No
b0474	No	Yes	No	No	No	No	No	No	No	No	No	No	No
b0475	No	No	No	Yes	No	No	No	No	No	No	No	No	No

b0477	No	No	No	No	Yes	No	No	No	No	No	No	No	No
b0480	No	No	No	No	No	No	No	No	No	No	No	No	Yes
b0484	No	No	No	No	No	No	No	No	No	No	No	Yes	No
b0485	No	No	No	No	No	No	No	No	No	No	No	No	Yes
b0493	No	No	No	No	No	No	No	No	No	No	No	No	Yes
b0494	No	No	Yes	No	No	No	No	No	No	No	No	No	No
b0505	No	No	No	No	No	No	No	No	No	No	No	No	Yes
b0507	No	No	No	No	No	No	No	No	No	No	No	No	Yes
b0508	No	No	No	No	No	No	No	No	No	No	No	No	Yes
b0509	No	No	No	No	No	No	No	No	No	No	No	No	Yes
b0511	No	No	No	No	No	No	No	No	No	No	No	Yes	No
b0512	No	No	No	No	No	No	No	No	No	No	No	No	Yes
b0514	No	No	No	No	No	No	No	No	No	No	No	No	Yes
b0515	No	No	No	No	No	No	No	No	No	No	No	No	Yes
b0516	No	No	No	No	No	No	No	No	No	No	No	No	Yes
b0517	No	No	No	No	No	No	No	No	No	No	No	No	Yes
b0521	No	No	No	No	No	No	No	No	No	No	No	No	Yes
b0522	No	Yes	No	No	No	No	No	No	No	No	No	No	No
b0523	No	Yes	No	No	No	No	No	No	No	No	No	No	No
b0524	No	No	Yes	No	No	No	No	No	No	No	No	No	No
b0529	No	No	No	Yes	No	No	No	No	No	No	No	No	No
b0543	No	No	No	No	No	No	No	No	No	No	No	Yes	No
b0572	No	No	No	No	No	No	No	No	No	No	No	Yes	No
b0573	No	No	No	No	No	No	No	No	No	No	No	Yes	No
b0574	No	No	No	No	No	No	No	No	No	No	No	Yes	No
b0575	No	No	No	No	No	No	No	No	No	No	No	Yes	No
b0576	No	No	No	No	No	No	No	No	No	No	No	Yes	No
b0577	No	No	No	No	No	No	No	No	No	No	No	Yes	No
b0578	No	No	No	No	No	No	No	No	No	No	No	No	Yes
b0583	No	No	No	No	Yes	No	No	No	No	No	No	No	No
b0584	No	No	No	No	No	No	No	No	No	No	No	Yes	No
b0585	No	No	No	No	No	No	No	No	No	No	No	No	Yes
b0586	No	No	No	Yes	No	No	No	No	No	No	No	No	No
b0588	No	No	No	No	No	No	No	No	No	No	No	Yes	No
b0589	No	No	No	No	No	No	No	No	No	No	No	Yes	No
b0590	No	No	No	No	No	No	No	No	No	No	No	Yes	No
b0591	No	No	No	No	No	No	No	No	No	No	No	Yes	No
b0592	No	No	No	No	No	No	No	No	No	No	No	Yes	No
b0593	No	No	No	No	Yes	No	No	No	No	No	No	No	No
b0594	No	No	No	No	Yes	No	No	No	No	No	No	No	No
b0595	No	No	No	Yes	No	No	No	No	No	No	No	No	No
b0596	No	No	No	No	Yes	No	No	No	No	No	No	No	No
b0597	No	No	No	No	No	No	No	No	No	No	No	No	Yes
b0604	No	No	No	No	No	No	No	No	No	No	No	Yes	No
b0605	No	No	No	No	No	No	No	No	No	No	No	No	Yes
b0606	No	No	No	No	No	No	No	No	No	No	No	No	Yes
b0612	No	No	No	No	No	No	No	No	No	No	No	Yes	No
b0613	No	No	No	Yes	No	No	No	No	No	No	No	No	No
b0614	No	No	No	Yes	No	No	No	No	No	No	No	No	No
b0615	No	No	No	Yes	No	No	No	No	No	No	No	No	No
b0616	No	No	No	Yes	No	No	No	No	No	No	No	No	No
b0617	No	No	No	Yes	No	No	No	No	No	No	No	No	No
b0621	No	No	No	No	No	No	No	No	No	No	No	Yes	No
b0622	No	No	No	No	No	No	No	No	No	No	No	No	Yes
b0624	No	No	No	No	No	No	No	No	No	No	No	Yes	No
b0628	No	No	No	Yes	No	No	No	No	No	No	No	No	No
b0630	No	No	No	Yes	No	No	No	No	No	No	No	No	No
b0632	No	No	Yes	No	No	No	No	No	No	No	No	No	No
b0635	No	No	Yes	No	No	No	No	No	No	No	No	No	No
b0638	No	No	No	Yes	No	No	No	No	No	No	No	No	No
b0639	No	No	No	Yes	No	No	No	No	No	No	No	No	No
b0641	No	No	No	No	No	No	No	No	No	No	No	No	Yes
b0650	No	No	No	No	No	No	No	No	No	No	No	No	Yes
b0651	No	No	No	No	Yes	No	No	No	No	No	No	No	No
b0652	No	No	No	No	No	No	No	No	No	No	No	Yes	No
b0653	No	No	No	No	No	No	No	No	No	No	No	Yes	No
b0654	No	No	No	No	No	No	No	No	No	No	No	Yes	No
b0655	No	No	No	No	No	No	No	No	No	No	No	Yes	No
b0657	No	No	No	No	No	No	No	No	No	No	No	No	Yes
b0662	No	No	No	Yes	No	No	No	No	No	No	No	No	No
b0674	Yes	No	No	No	No	No	No	No	No	No	No	No	No
b0675	No	No	No	No	No	No	No	No	No	No	No	No	Yes
b0677	No	No	No	No	No	No	No	No	No	No	No	No	Yes
b0678	No	No	No	No	No	No	No	No	No	No	No	No	Yes
b0679	No	No	No	No	No	No	No	No	No	No	No	Yes	No
b0681	No	No	No	No	No	No	No	No	No	No	No	Yes	No
b0684	No	No	No	No	Yes	No	No	No	No	No	No	No	No
b0688	No	No	No	No	Yes	No	No	No	No	No	No	No	No
b0692	No	No	No	No	No	No	No	No	No	No	No	Yes	No
b0693	No	No	No	No	No	No	No	No	No	No	No	No	Yes
b0696	No	No	No	No	No	No	No	No	No	No	No	Yes	No
b0697	No	No	No	No	No	No	No	No	No	No	No	Yes	No
b0698	No	No	No	No	No	No	No	No	No	No	No	Yes	No
b0709	No	No	No	No	No	No	No	No	No	No	No	Yes	No
b0720	No	No	No	No	No	No	No	Yes	No	No	No	No	No
b0721	No	No	No	No	No	No	No	Yes	No	Yes	No	No	No
b0722	No	No	No	No	No	No	No	Yes	No	Yes	No	No	No
b0723	No	No	No	No	No	No	No	Yes	No	Yes	No	No	No
b0724	No	No	No	No	No	No	No	Yes	No	Yes	No	No	No
b0726	No	No	No	No	No	No	No	Yes	No	No	No	No	No
b0727	No	No	No	No	No	No	No	Yes	No	No	No	No	No

b0728	No	No	No	No	No	No	No	Yes	No	No	No	No	No
b0729	No	No	No	No	No	No	No	Yes	No	No	No	No	No
b0731	No	No	No	No	No	No	No	No	No	No	No	Yes	No
b0732	No	No	No	No	No	No	No	No	No	No	No	No	Yes
b0733	No	No	No	No	No	No	No	No	No	Yes	No	No	No
b0734	No	No	No	No	No	No	No	No	No	Yes	No	No	No
b0750	No	No	No	Yes	No	No	No	No	No	No	No	No	No
b0751	No	No	No	No	No	No	No	No	No	No	No	Yes	No
b0752	No	No	No	No	No	No	No	No	No	No	No	Yes	No
b0754	Yes	No	No	No	No	No	No	No	No	No	No	No	No
b0755	No	No	No	No	No	Yes	No	No	No	No	No	No	No
b0756	No	No	No	No	Yes	No	No	No	No	No	No	No	No
b0757	No	No	No	No	Yes	No	No	No	No	No	No	No	No
b0758	No	No	No	No	Yes	No	No	No	No	No	No	No	No
b0759	No	No	No	No	Yes	No	No	No	No	No	No	No	No
b0763	No	No	No	Yes	No	No	No	No	No	No	No	Yes	No
b0764	No	No	No	Yes	No	No	No	No	No	No	No	Yes	No
b0765	No	No	No	Yes	No	No	No	No	No	No	No	Yes	No
b0766	No	No	No	No	Yes	Yes	No	No	No	No	No	No	No
b0767	No	No	No	No	No	No	Yes	No	No	No	No	No	No
b0774	No	No	No	Yes	No	No	No	No	No	No	No	No	No
b0775	No	No	No	Yes	No	No	No	No	No	No	No	No	No
b0776	No	No	No	Yes	No	No	No	No	No	No	No	No	No
b0777	No	No	No	Yes	No	No	No	No	No	No	No	No	No
b0778	No	No	No	Yes	No	No	No	No	No	No	No	No	No
b0781	No	No	No	Yes	No	No	No	No	No	No	No	No	No
b0783	No	No	No	Yes	No	No	No	No	No	No	No	No	No
b0784	No	No	No	Yes	No	No	No	No	No	No	No	No	No
b0785	No	No	No	Yes	No	No	No	No	No	No	No	No	No
b0789	No	No	Yes	No	No	No	No	No	No	No	No	No	No
b0805	No	No	No	No	No	No	No	No	No	No	No	Yes	No
b0808	No	No	No	No	No	No	No	No	No	No	No	Yes	No
b0809	No	No	No	No	No	No	No	No	No	No	No	Yes	No
b0810	No	No	No	No	No	No	No	No	No	No	No	Yes	No
b0811	No	No	No	No	No	No	No	No	No	No	No	Yes	No
b0813	No	No	No	No	No	No	No	No	No	No	No	Yes	No
b0819	No	No	No	No	No	No	No	No	No	No	No	No	Yes
b0822	No	No	No	No	No	No	No	No	No	No	No	No	Yes
b0825	No	No	No	No	No	No	No	No	No	No	No	No	Yes
b0826	No	No	No	Yes	No	No	No	No	No	No	No	No	No
b0827	No	No	No	Yes	No	No	No	No	No	No	No	No	No
b0828	Yes	No	No	No	No	No	No	No	No	No	No	No	No
b0829	No	No	No	No	No	No	No	No	No	No	No	Yes	No
b0830	No	No	No	No	No	No	No	No	No	No	No	Yes	No
b0831	No	No	No	No	No	No	No	No	No	No	No	Yes	No
b0832	No	No	No	No	No	No	No	No	No	No	No	Yes	No
b0837	No	No	No	No	No	No	No	No	No	No	No	No	Yes
b0839	No	No	Yes	No	No	No	No	No	No	No	No	No	No
b0841	No	No	No	Yes	No	No	No	No	No	No	No	No	No
b0842	No	No	No	No	No	No	No	No	No	No	No	Yes	No
b0844	No	No	No	Yes	No	No	No	No	No	No	No	No	No
b0849	Yes	No	No	Yes	No	No	No	No	No	No	No	No	No
b0854	No	No	No	No	No	No	No	No	No	No	No	Yes	No
b0855	No	No	No	No	No	No	No	No	No	No	No	Yes	No
b0856	No	No	No	No	No	No	No	No	No	No	No	Yes	No
b0857	No	No	No	No	No	No	No	No	No	No	No	Yes	No
b0860	No	No	No	No	No	No	No	No	No	No	No	Yes	No
b0861	No	No	No	No	No	No	No	No	No	No	No	Yes	No
b0862	No	No	No	No	No	No	No	No	No	No	No	Yes	No
b0863	No	No	No	No	No	No	No	No	No	No	No	Yes	No
b0864	No	No	No	No	No	No	No	No	No	No	No	Yes	No
b0867	No	No	No	No	No	No	No	No	No	No	No	No	Yes
b0870	No	No	No	No	No	No	No	No	No	No	No	No	Yes
b0871	No	No	No	No	No	No	No	No	No	Yes	No	No	No
b0874	No	No	No	No	No	No	No	No	No	No	No	Yes	No
b0875	No	No	No	No	No	No	No	No	No	No	No	Yes	No
b0886	No	No	No	No	No	No	No	No	No	No	No	Yes	No
b0887	No	No	No	No	No	No	No	No	No	No	No	Yes	No
b0888	No	No	No	Yes	No	No	No	No	No	No	No	No	No
b0894	No	No	No	No	No	No	No	No	No	Yes	No	No	No
b0895	No	No	No	No	No	No	No	No	No	Yes	No	No	No
b0896	No	No	No	No	No	No	No	No	No	Yes	No	No	No
b0902	No	No	No	No	No	No	No	No	No	No	No	No	Yes
b0903	No	No	No	No	No	No	No	No	No	No	Yes	No	No
b0904	No	No	No	No	No	No	No	No	No	No	No	Yes	No
b0905	No	No	No	No	No	No	No	No	No	No	No	No	Yes
b0907	Yes	No	No	Yes	No	No	No	No	No	No	No	No	No
b0908	Yes	No	No	No	No	No	No	No	No	No	No	No	No
b0910	No	No	No	No	Yes	No	No	No	No	No	No	No	No
b0914	No	No	No	No	No	No	No	No	No	No	No	Yes	No
b0915	No	No	Yes	No	No	No	No	No	No	No	No	No	No
b0918	No	No	Yes	No	No	No	No	No	No	No	No	No	No
b0925	No	No	Yes	No	No	No	No	No	No	No	No	No	No
b0928	Yes	No	No	No	No	No	No	No	No	No	No	No	No
b0929	No	No	No	No	No	No	No	No	No	No	No	Yes	No
b0931	No	No	No	Yes	No	No	No	No	No	No	No	No	No
b0932	No	No	No	No	No	No	No	No	No	No	No	No	Yes
b0933	No	No	No	No	No	No	No	No	No	No	No	Yes	No
b0934	No	No	No	No	No	No	No	No	No	No	No	Yes	No
b0935	No	No	No	No	No	No	No	No	No	No	No	No	Yes
b0936	No	No	No	No	No	No	No	No	No	No	No	Yes	No

b0937	No	No	No	No	No	No	No	No	No	No	No	No	Yes
b0945	No	Yes	No	No	No	No	No	No	No	No	No	No	No
b0954	No	No	Yes	No	No	No	No	No	No	No	No	No	No
b0957	No	No	No	No	No	No	No	No	No	No	No	Yes	No
b0963	No	No	No	No	No	No	No	No	No	No	No	No	Yes
b0968	No	No	No	No	No	No	No	No	No	No	No	No	Yes
b0972	No	No	No	No	No	No	No	No	No	Yes	No	No	No
b0973	No	No	No	No	No	No	No	No	No	Yes	No	No	No
b0974	No	No	No	No	No	No	No	No	No	Yes	No	No	No
b0978	No	No	No	No	No	No	No	No	No	No	No	No	Yes
b0979	No	No	No	No	No	No	No	No	No	No	No	No	Yes
b0980	No	No	No	No	No	No	No	No	No	No	No	No	Yes
b0996	No	No	No	Yes	No	No	No	No	No	Yes	No	No	No
b0997	No	No	No	No	No	No	No	No	No	Yes	No	No	No
b1002	No	No	No	No	No	No	No	No	No	No	No	No	Yes
b1004	No	No	No	No	No	No	No	No	No	No	No	No	Yes
b1006	No	No	No	No	No	No	No	No	No	No	No	Yes	No
b1007	No	No	No	No	No	No	No	No	No	No	No	No	Yes
b1008	No	No	No	No	No	No	No	No	No	No	No	No	Yes
b1009	No	No	No	No	No	No	No	No	No	No	No	No	Yes
b1010	No	No	No	No	No	No	No	No	No	No	No	No	Yes
b1011	No	No	No	No	No	No	No	No	No	No	No	No	Yes
b1012	No	No	No	No	No	No	No	No	No	No	No	No	Yes
b1014	No	No	No	No	No	No	No	No	No	Yes	No	No	No
b1015	No	No	No	No	No	No	No	No	No	No	No	Yes	No
b1019	No	No	No	No	No	No	No	No	No	No	No	No	Yes
b1021	No	No	No	No	No	No	No	No	No	No	No	Yes	No
b1022	No	No	No	No	No	No	No	No	No	No	No	Yes	No
b1025	No	No	No	No	No	No	No	No	No	No	No	No	Yes
b1033	No	No	No	No	No	No	No	No	No	No	No	No	Yes
b1054	No	No	Yes	No	No	No	No	No	No	No	No	No	No
b1059	No	No	No	No	No	No	No	No	No	No	No	No	Yes
b1062	No	Yes	No	No	No	No	No	No	No	No	No	No	No
b1064	Yes	No	No	Yes	No	No	No	No	No	No	No	No	No
b1069	No	No	No	No	No	No	No	No	No	No	No	No	Yes
b1090	No	No	No	No	No	No	No	No	No	No	No	No	Yes
b1091	No	No	Yes	No	No	No	No	No	No	No	No	No	No
b1092	No	No	Yes	No	No	No	No	No	No	No	No	No	No
b1093	No	No	Yes	Yes	No	No	No	No	No	No	No	No	No
b1094	No	No	Yes	Yes	No	No	No	No	No	No	No	No	No
b1095	No	No	Yes	Yes	No	No	No	No	No	No	No	No	No
b1096	No	No	No	Yes	No	No	No	No	No	No	No	No	No
b1097	No	No	No	No	No	No	No	No	No	No	No	No	Yes
b1098	No	Yes	No	No	No	No	No	No	No	No	No	No	No
b1101	No	No	No	No	No	No	No	No	No	No	No	Yes	No
b1102	No	No	No	No	No	No	No	No	No	No	No	Yes	No
b1103	No	No	No	No	No	No	No	No	No	No	No	No	Yes
b1106	No	No	No	No	No	No	No	No	No	No	No	No	Yes
b1107	No	No	No	No	No	No	No	No	No	No	No	No	Yes
b1109	No	No	No	No	No	No	No	No	No	Yes	No	No	No
b1113	No	No	No	No	No	No	No	No	No	No	No	No	Yes
b1119	No	No	No	No	No	No	No	No	No	No	No	No	Yes
b1123	No	No	No	No	No	No	No	No	No	No	No	Yes	No
b1124	No	No	No	No	No	No	No	No	No	No	No	Yes	No
b1125	No	No	No	No	No	No	No	No	No	No	No	Yes	No
b1126	No	No	No	No	No	No	No	No	No	No	No	Yes	No
b1131	No	Yes	No	No	No	No	No	No	No	No	No	No	No
b1134	No	No	No	Yes	No	No	No	No	No	No	No	No	No
b1136	No	No	No	No	No	No	No	Yes	No	No	No	No	No
b1185	No	No	No	No	No	No	No	No	No	No	No	No	Yes
b1186	No	No	No	No	No	No	No	No	No	No	No	Yes	No
b1189	No	No	No	No	No	No	No	No	No	No	No	No	Yes
b1190	Yes	No	No	No	No	No	No	No	No	No	No	No	No
b1192	No	No	No	No	No	No	No	No	No	No	No	No	Yes
b1193	No	No	No	No	No	No	No	No	No	No	No	No	Yes
b1197	No	No	No	No	No	No	No	No	No	No	No	No	Yes
b1198	No	No	No	No	No	No	No	No	No	No	No	No	Yes
b1199	No	No	No	No	No	No	No	No	No	No	No	No	Yes
b1200	No	No	No	No	No	No	No	No	No	No	No	No	Yes
b1206	No	No	No	No	No	No	No	No	No	No	No	Yes	No
b1207	No	No	No	No	Yes	No	No	No	No	No	No	No	No
b1208	No	No	No	No	Yes	No	No	No	No	No	No	No	No
b1210	No	No	No	No	Yes	No	No	No	No	No	No	No	No
b1215	No	No	Yes	No	No	No	No	No	No	No	No	No	No
b1216	No	No	No	No	No	No	No	No	No	No	No	Yes	No
b1223	No	No	No	No	No	No	No	No	No	No	No	Yes	No
b1224	No	No	No	No	No	No	No	No	No	Yes	No	No	No
b1225	No	No	No	No	No	No	No	No	No	Yes	No	No	No
b1226	No	No	No	No	No	No	No	No	No	No	No	No	Yes
b1227	No	No	No	No	No	No	No	No	No	Yes	No	No	No
b1232	No	No	No	No	No	No	No	No	No	No	No	No	Yes
b1236	No	No	No	No	Yes	No	No	No	No	No	No	No	No
b1238	No	No	No	No	Yes	No	No	No	No	No	No	No	No
b1241	No	No	No	No	No	No	No	No	No	No	Yes	No	No
b1243	No	No	No	No	No	No	No	No	No	No	No	No	Yes
b1244	No	No	No	No	No	No	No	No	No	No	No	No	Yes
b1245	No	No	No	No	No	No	No	No	No	No	No	No	Yes
b1246	No	No	No	No	No	No	No	No	No	No	No	No	Yes
b1247	No	No	No	No	No	No	No	No	No	No	No	No	Yes
b1249	No	No	Yes	No	No	No	No	No	No	No	No	No	No
b1250	No	No	No	No	No	No	No	No	No	No	No	Yes	No

b1252	No	No	No	No	No	No	No	No	No	No	No	Yes	No
b1260	Yes	No	No	No	No	No	No	No	No	No	No	No	No
b1261	Yes	No	No	No	No	No	No	No	No	No	No	No	No
b1262	Yes	No	No	No	No	No	No	No	No	No	No	No	No
b1263	Yes	No	No	No	No	No	No	No	No	No	No	No	No
b1264	Yes	No	No	No	No	No	No	No	No	No	No	No	No
b1270	No	No	No	Yes	No	No	No	No	No	No	No	No	No
b1276	No	No	No	No	No	No	No	Yes	No	No	No	No	No
b1277	No	No	No	Yes	No	No	No	No	No	No	No	No	No
b1278	No	No	Yes	Yes	No	No	No	No	No	No	No	No	No
b1281	No	Yes	No	No	No	No	No	No	No	No	No	No	No
b1285	No	No	No	No	No	No	No	No	No	No	No	No	Yes
b1288	No	No	Yes	Yes	No	No	No	No	No	No	No	No	No
b1291	No	No	No	No	No	No	No	No	No	No	No	Yes	No
b1296	No	No	No	No	No	No	No	No	No	No	No	Yes	No
b1297	No	No	No	No	No	No	No	No	No	No	No	No	Yes
b1298	No	No	No	No	No	No	No	No	No	No	No	No	Yes
b1300	No	No	No	No	No	No	No	No	No	No	No	No	Yes
b1301	No	No	No	No	No	No	No	No	No	No	No	No	Yes
b1302	No	No	No	No	No	No	No	No	No	No	No	No	Yes
b1308	No	No	No	No	No	No	No	No	No	No	No	Yes	No
b1317	No	No	No	No	No	No	No	No	No	No	No	No	Yes
b1319	No	No	No	No	No	No	No	No	No	No	No	Yes	No
b1325	No	No	No	No	No	No	No	No	No	No	No	No	Yes
b1326	No	No	No	No	No	No	No	No	No	No	No	No	Yes
b1329	No	No	No	No	No	No	No	No	No	No	No	No	Yes
b1330	No	No	No	No	No	No	No	No	No	No	No	Yes	No
b1336	No	No	No	No	No	No	No	No	No	No	No	Yes	No
b1337	No	No	No	No	No	No	No	No	No	No	No	No	Yes
b1338	No	No	No	No	No	No	No	No	No	No	No	No	Yes
b1341	No	No	No	No	No	No	No	No	No	No	No	No	Yes
b1363	No	No	No	No	No	No	No	No	No	No	No	Yes	No
b1377	No	No	No	No	No	No	No	No	No	No	No	Yes	No
b1378	No	No	No	No	No	No	No	No	No	No	No	No	Yes
b1380	No	No	No	No	No	No	No	No	No	No	Yes	No	No
b1385	No	No	No	No	No	No	No	No	No	No	No	No	Yes
b1386	No	No	No	No	No	No	No	No	No	No	No	No	Yes
b1387	No	No	No	No	No	No	No	No	No	No	No	No	Yes
b1388	No	No	No	No	No	No	No	No	No	No	No	No	Yes
b1389	No	No	No	No	No	No	No	No	No	No	No	No	Yes
b1390	No	No	No	No	No	No	No	No	No	No	No	No	Yes
b1391	No	No	No	No	No	No	No	No	No	No	No	No	Yes
b1392	No	No	No	No	No	No	No	No	No	No	No	No	Yes
b1393	No	No	No	No	No	No	No	No	No	No	No	No	Yes
b1394	No	No	No	No	No	No	No	No	No	No	No	No	Yes
b1395	No	No	No	No	No	No	No	No	No	No	No	No	Yes
b1396	No	No	No	No	No	No	No	No	No	No	No	No	Yes
b1397	No	No	No	No	No	No	No	No	No	No	No	No	Yes
b1398	No	No	No	No	No	No	No	No	No	No	No	No	Yes
b1400	No	No	No	No	No	No	No	No	No	No	No	No	Yes
b1415	No	No	No	No	No	No	No	No	No	No	No	No	Yes
b1430	No	No	No	No	No	No	No	No	No	No	No	No	Yes
b1440	No	No	No	No	No	No	No	No	No	No	No	Yes	No
b1441	No	No	No	No	No	No	No	No	No	No	No	Yes	No
b1442	No	No	No	No	No	No	No	No	No	No	No	Yes	No
b1443	No	No	No	No	No	No	No	No	No	No	No	Yes	No
b1444	No	No	No	No	No	No	No	No	No	No	No	No	Yes
b1448	No	No	No	No	No	No	No	No	No	No	No	No	Yes
b1449	No	No	No	No	No	No	No	No	No	No	No	No	Yes
b1453	No	No	No	No	No	No	No	No	No	No	No	Yes	No
b1463	No	No	No	No	No	No	No	No	No	No	No	No	Yes
b1465	No	No	No	No	No	No	No	No	No	Yes	No	No	No
b1466	No	No	No	No	No	No	No	No	No	No	No	No	Yes
b1467	No	No	No	No	No	No	No	No	No	Yes	No	No	No
b1468	No	No	No	No	No	No	No	No	No	Yes	No	No	No
b1469	No	No	No	No	No	No	No	No	No	No	No	Yes	No
b1473	No	No	No	No	No	No	No	No	No	No	No	Yes	No
b1474	No	No	No	No	No	No	No	No	No	Yes	No	No	No
b1475	No	No	No	No	No	No	No	No	No	Yes	No	No	No
b1476	No	No	No	No	No	No	No	No	No	Yes	No	No	No
b1478	No	No	No	No	No	No	No	No	No	No	Yes	No	No
b1479	No	No	No	No	Yes	No	No	Yes	No	No	No	No	No
b1483	No	No	No	No	No	No	No	No	No	No	No	Yes	No
b1484	No	No	No	No	No	No	No	No	No	No	No	Yes	No
b1485	No	No	No	No	No	No	No	No	No	No	No	Yes	No
b1486	No	No	No	No	No	No	No	No	No	No	No	Yes	No
b1487	No	No	No	No	No	No	No	No	No	No	No	Yes	No
b1488	No	No	No	No	No	No	No	No	No	No	No	No	Yes
b1489	No	No	No	No	No	No	No	No	No	No	No	No	Yes
b1490	No	No	No	No	No	No	No	No	No	No	No	No	Yes
b1492	No	No	No	No	No	No	No	No	No	No	No	Yes	No
b1493	No	No	No	No	No	No	No	No	No	No	No	No	Yes
b1511	No	No	No	No	No	No	No	No	No	No	No	No	Yes
b1513	No	No	No	No	No	No	No	No	No	No	No	Yes	No
b1514	No	No	No	No	No	No	No	No	No	No	No	Yes	No
b1515	No	No	No	No	No	No	No	No	No	No	No	Yes	No
b1516	No	No	No	No	No	No	No	No	No	No	No	Yes	No
b1517	No	No	No	No	No	No	No	No	No	No	No	No	Yes
b1518	No	No	No	No	No	No	No	No	No	No	No	No	Yes
b1519	No	No	No	No	No	No	No	No	No	No	No	No	Yes
b1521	No	No	No	No	No	No	No	No	No	No	No	No	Yes

b1524	No	No	No	No	No	No	No	No	No	No	No	No	Yes
b1525	No	No	No	No	No	No	No	No	No	No	No	No	Yes
b1528	No	No	No	No	No	No	No	No	No	No	No	Yes	No
b1533	No	No	No	No	No	No	No	No	No	No	No	Yes	No
b1535	No	No	No	No	No	No	No	No	No	No	No	No	Yes
b1539	No	No	No	No	No	No	No	No	No	No	No	No	Yes
b1584	No	No	No	No	No	No	No	No	No	No	No	No	Yes
b1587	No	No	No	No	No	No	No	No	No	No	No	No	Yes
b1588	No	No	No	No	No	No	No	No	No	No	No	No	Yes
b1589	No	No	No	No	No	No	No	No	No	No	No	No	Yes
b1590	No	No	No	No	No	No	No	No	No	No	No	No	Yes
b1592	No	No	No	No	No	No	No	No	No	No	No	Yes	No
b1599	No	No	No	No	No	No	No	No	No	No	No	Yes	No
b1600	No	No	No	No	No	No	No	No	No	No	No	Yes	No
b1601	No	No	No	No	No	No	No	No	No	No	No	Yes	No
b1602	No	No	No	Yes	No	No	No	No	No	No	No	No	No
b1603	No	No	No	Yes	No	No	No	No	No	No	No	No	No
b1605	No	No	No	No	No	No	No	No	No	No	No	Yes	No
b1606	No	No	No	Yes	No	No	No	No	No	No	No	No	No
b1611	No	No	No	No	No	No	No	Yes	No	No	No	No	No
b1612	No	No	No	No	No	No	No	Yes	No	No	No	No	No
b1613	No	No	No	No	Yes	No	No	No	No	No	No	No	No
b1616	No	No	No	No	No	No	No	No	No	No	No	Yes	No
b1617	No	No	No	No	No	No	No	No	No	No	No	No	Yes
b1619	No	No	No	No	No	No	No	No	No	No	No	No	Yes
b1621	No	No	No	No	No	No	No	No	No	No	No	Yes	No
b1622	Yes	No	No	No	No	No	No	No	No	No	No	No	No
b1623	No	No	No	No	Yes	No	No	No	No	No	No	No	No
b1634	No	No	No	No	No	No	No	No	No	No	No	Yes	No
b1636	No	No	No	Yes	No	No	No	No	No	No	No	No	No
b1638	No	No	No	Yes	No	No	No	No	No	No	No	No	No
b1640	No	No	No	No	No	No	No	No	No	No	No	No	Yes
b1646	No	No	No	No	No	No	No	No	No	No	No	No	Yes
b1651	No	No	No	No	No	No	No	No	No	No	No	No	Yes
b1654	No	No	No	No	No	No	No	No	No	No	No	No	Yes
b1655	No	No	No	No	No	No	No	No	No	No	No	No	Yes
b1656	No	No	No	No	No	No	No	No	No	No	No	No	Yes
b1661	No	No	Yes	No	No	No	No	No	No	No	No	No	No
b1662	No	No	No	Yes	No	No	No	No	No	No	No	No	No
b1676	No	No	No	No	No	Yes	No	No	No	No	No	No	No
b1677	No	No	No	No	No	No	No	No	No	No	No	No	Yes
b1678	No	No	Yes	No	No	No	No	No	No	No	No	No	No
b1679	No	No	No	No	No	No	No	No	No	No	No	No	Yes
b1680	Yes	No	No	Yes	No	No	No	No	No	No	No	No	No
b1681	No	No	No	No	No	No	No	No	No	No	No	No	Yes
b1682	No	No	No	No	No	No	No	No	No	No	No	No	Yes
b1683	No	No	No	No	No	No	No	No	No	No	No	No	Yes
b1684	No	No	No	No	No	No	No	No	No	No	No	No	Yes
b1686	No	No	No	Yes	No	No	No	No	No	No	No	No	No
b1692	No	No	No	No	No	No	No	No	No	No	No	No	Yes
b1693	Yes	No	No	No	No	No	No	No	No	No	No	No	No
b1694	No	No	No	No	No	No	No	No	No	No	No	No	Yes
b1695	No	No	No	No	No	No	No	No	No	No	No	No	Yes
b1701	No	No	No	No	No	No	No	No	No	No	No	No	Yes
b1702	No	No	No	No	Yes	Yes	No	No	No	No	No	No	No
b1704	Yes	No	No	No	No	No	No	No	No	No	No	No	No
b1709	No	No	No	No	No	No	No	No	No	No	No	Yes	No
b1710	No	No	No	No	No	No	No	No	No	No	No	No	Yes
b1711	No	No	No	No	No	No	No	No	No	No	No	Yes	No
b1723	No	No	No	No	No	Yes	No	No	No	No	No	No	No
b1727	No	No	No	No	No	No	No	No	No	No	No	No	Yes
b1732	No	No	No	No	No	No	No	No	No	No	No	No	Yes
b1733	No	No	No	No	No	No	No	No	No	No	No	No	Yes
b1734	No	No	No	No	No	No	No	No	No	No	No	No	Yes
b1736	No	No	No	No	No	No	No	No	No	No	No	Yes	No
b1737	No	No	No	No	No	No	No	No	No	No	No	Yes	No
b1738	No	No	No	No	No	No	No	No	No	No	No	Yes	No
b1740	No	No	No	Yes	No	No	No	No	No	No	No	No	No
b1744	No	No	No	No	No	No	No	No	No	No	No	No	Yes
b1745	No	No	No	No	No	No	No	No	No	No	No	No	Yes
b1746	No	No	No	No	No	No	No	No	No	No	No	No	Yes
b1747	No	No	No	No	No	No	No	No	No	No	No	No	Yes
b1748	No	No	No	No	No	No	No	No	No	No	No	No	Yes
b1759	No	No	No	No	No	No	No	No	No	No	No	No	Yes
b1761	Yes	No	No	No	No	No	No	No	No	No	No	No	No
b1764	Yes	No	No	No	No	No	No	No	No	No	No	No	No
b1767	Yes	No	No	No	No	No	No	No	No	No	No	No	No
b1768	No	No	No	Yes	No	No	No	No	No	No	No	No	No
b1771	No	No	No	No	No	No	No	No	No	No	No	No	Yes
b1778	No	No	No	No	No	No	No	No	No	No	No	No	Yes
b1779	No	No	No	No	Yes	Yes	No	No	No	No	No	No	No
b1781	No	No	No	No	No	No	No	No	No	No	No	No	Yes
b1794	No	No	No	No	No	No	No	No	No	No	No	No	Yes
b1798	No	No	No	No	No	No	No	No	No	No	No	Yes	No
b1800	Yes	No	No	No	No	No	No	No	No	No	No	No	No
b1801	No	No	No	No	No	No	No	No	No	No	No	Yes	No
b1805	No	No	No	No	No	No	No	No	No	No	No	No	Yes
b1812	No	No	No	Yes	No	No	No	No	No	No	No	No	No
b1813	No	No	No	No	No	No	No	No	No	No	No	No	Yes
b1814	No	No	No	No	No	No	No	No	No	No	No	No	Yes
b1817	No	No	No	No	No	No	No	No	No	No	No	Yes	No

b1818	No	No	No	No	No	No	No	No	No	No	No	Yes	No
b1819	No	No	No	No	No	No	No	No	No	No	No	Yes	No
b1821	No	No	No	No	No	No	No	No	No	No	No	Yes	No
b1832	No	No	No	No	No	No	No	No	No	No	No	No	Yes
b1849	No	Yes	No	No	No	No	No	No	No	No	No	No	No
b1850	No	No	No	No	No	Yes	No	No	No	No	No	No	No
b1851	No	No	No	No	No	Yes	No	No	No	No	No	No	No
b1852	No	No	No	No	No	No	Yes	No	No	No	No	No	No
b1854	No	No	No	No	No	Yes	No	No	No	No	No	No	No
b1855	No	No	Yes	No	No	No	No	No	No	No	No	No	No
b1856	No	No	No	No	No	No	No	No	No	No	No	No	Yes
b1857	No	No	No	No	No	No	No	No	No	No	No	Yes	No
b1858	No	No	No	No	No	No	No	No	No	No	No	Yes	No
b1859	No	No	No	No	No	No	No	No	No	No	No	Yes	No
b1865	No	No	No	Yes	No	No	No	No	No	No	No	No	No
b1870	No	No	No	No	No	No	No	No	No	No	No	No	Yes
b1872	No	No	No	No	No	No	No	No	No	No	No	No	Yes
b1873	No	No	No	Yes	No	No	No	No	No	No	No	No	No
b1896	No	No	No	No	Yes	No	No	No	No	No	No	No	No
b1897	No	No	No	No	Yes	No	No	No	No	No	No	No	No
b1900	No	No	No	No	No	No	No	No	No	No	No	Yes	No
b1901	No	No	No	No	No	No	No	No	No	No	No	Yes	No
b1907	No	No	No	No	No	No	No	No	No	No	No	Yes	No
b1912	No	No	Yes	No	No	No	No	No	No	No	No	No	No
b1917	No	No	No	No	No	No	No	No	No	No	No	Yes	No
b1918	No	No	No	No	No	No	No	No	No	No	No	Yes	No
b1919	No	No	No	No	No	No	No	No	No	No	No	No	Yes
b1920	No	No	No	No	No	No	No	No	No	No	No	Yes	No
b1927	No	No	No	No	No	No	No	No	No	No	No	No	Yes
b1967	No	No	No	No	No	No	No	No	No	No	No	No	Yes
b1981	No	No	No	No	No	No	No	No	No	No	No	Yes	No
b1982	No	No	No	No	No	No	No	No	No	No	No	No	Yes
b1985	No	No	No	No	No	No	No	No	No	No	No	Yes	No
b1990	No	No	No	No	No	No	No	No	No	No	No	No	Yes
b1991	No	No	No	Yes	No	No	No	No	No	No	No	No	No
b1992	No	No	No	Yes	No	No	No	No	No	No	No	No	No
b1993	No	No	No	Yes	No	No	No	No	No	No	No	No	No
b2010	No	No	Yes	No	No	No	No	No	No	No	No	No	No
b2014	No	No	No	No	No	No	No	No	No	No	No	Yes	No
b2019	Yes	No	No	No	No	No	No	No	No	No	No	No	No
b2020	Yes	No	No	No	No	No	No	No	No	No	No	No	No
b2021	Yes	No	No	No	No	No	No	No	No	No	No	No	No
b2022	Yes	No	No	No	No	No	No	No	No	No	No	No	No
b2023	Yes	No	No	No	No	No	No	No	No	No	No	No	No
b2024	Yes	No	No	No	No	No	No	No	No	No	No	No	No
b2025	Yes	No	No	No	No	No	No	No	No	No	No	No	No
b2026	Yes	No	No	No	No	No	No	No	No	No	No	No	No
b2027	No	No	No	No	No	No	No	No	No	No	No	No	Yes
b2028	No	No	No	No	Yes	No	No	No	No	No	No	No	No
b2029	No	No	No	No	No	No	Yes	No	No	No	No	No	No
b2032	No	No	No	No	No	No	No	No	No	No	No	No	Yes
b2033	No	No	No	No	No	No	No	No	No	No	No	No	Yes
b2034	No	No	No	No	No	No	No	No	No	No	No	No	Yes
b2035	No	No	No	No	No	No	No	No	No	No	No	No	Yes
b2036	No	No	No	No	Yes	No	No	No	No	No	No	No	No
b2037	No	No	No	No	No	No	No	No	No	No	No	No	Yes
b2038	No	No	No	No	Yes	No	No	No	No	No	No	No	No
b2039	No	No	Yes	No	No	No	No	No	No	No	No	No	No
b2040	No	No	No	No	Yes	No	No	No	No	No	No	No	No
b2041	No	No	No	No	Yes	No	No	No	No	No	No	No	No
b2047	No	No	No	No	No	No	No	No	No	No	No	No	Yes
b2048	No	No	No	No	Yes	No	No	No	No	No	No	No	No
b2049	No	No	No	No	Yes	No	No	No	No	No	No	No	No
b2051	No	No	No	No	No	No	No	No	No	No	No	No	Yes
b2052	No	No	No	No	Yes	No	No	No	No	No	No	No	No
b2053	No	No	No	No	Yes	No	No	No	No	No	No	No	No
b2065	No	Yes	No	No	No	No	No	No	No	No	No	No	No
b2066	No	No	No	No	Yes	No	No	No	No	No	No	No	No
b2091	No	No	No	No	No	No	No	No	No	No	No	No	Yes
b2092	No	No	No	No	No	No	No	No	No	No	No	Yes	No
b2093	No	No	No	No	No	No	No	No	No	No	No	Yes	No
b2094	No	No	No	No	No	No	No	No	No	No	No	Yes	No
b2095	No	No	No	No	No	No	No	No	No	No	No	No	Yes
b2096	No	No	No	No	No	No	No	No	No	No	No	No	Yes
b2097	No	No	No	No	Yes	Yes	No	No	No	No	No	No	No
b2103	No	No	No	Yes	No	No	No	No	No	No	No	No	No
b2104	No	No	No	Yes	No	No	No	No	No	No	No	No	No
b2128	No	No	No	No	No	No	No	No	No	No	No	Yes	No
b2129	No	No	No	No	No	No	No	No	No	No	No	Yes	No
b2130	No	No	No	No	No	No	No	No	No	No	No	Yes	No
b2131	No	No	No	No	No	No	No	No	No	No	No	Yes	No
b2132	No	No	No	No	No	No	No	No	No	No	No	No	Yes
b2133	No	No	No	No	No	No	No	No	No	Yes	No	No	No
b2134	No	No	No	No	No	No	No	No	No	No	No	No	Yes
b2143	No	No	No	No	Yes	No	No	No	No	No	No	No	No
b2146	No	No	No	No	No	No	No	No	No	No	No	No	Yes
b2147	No	No	No	No	No	No	No	No	No	No	No	No	Yes
b2148	No	No	No	No	No	No	No	No	No	No	No	Yes	No
b2149	No	No	No	No	No	No	No	No	No	No	No	Yes	No
b2150	No	No	No	No	No	No	No	No	No	No	No	Yes	No
b2153	No	No	No	Yes	No	No	No	No	No	No	No	No	No

b2154	No	No	No	No	No	No	No	No	No	No	No	No	Yes
b2155	No	No	No	No	No	No	No	No	No	No	No	Yes	No
b2156	No	No	No	No	No	No	No	No	No	No	No	Yes	No
b2162	No	No	No	No	Yes	No	No	No	No	No	No	No	No
b2164	No	No	No	No	No	No	No	No	No	No	No	Yes	No
b2165	No	No	No	No	No	No	No	No	No	No	No	No	Yes
b2166	No	No	No	No	No	No	No	No	No	No	No	No	Yes
b2167	No	No	No	No	No	No	No	No	No	No	No	Yes	No
b2168	No	No	No	No	No	No	No	No	No	No	No	No	Yes
b2169	No	No	No	No	No	No	No	No	No	No	No	Yes	No
b2170	No	No	No	No	No	No	No	No	No	No	No	Yes	No
b2173	No	No	No	No	No	No	No	No	No	No	No	No	Yes
b2174	No	No	No	No	No	No	No	No	No	No	No	No	Yes
b2175	No	No	No	No	No	No	No	No	No	No	No	No	Yes
b2177	No	No	No	No	No	No	No	No	No	No	No	No	Yes
b2178	No	No	No	No	No	No	No	No	No	No	No	No	Yes
b2179	No	No	No	No	No	No	No	No	No	No	No	No	Yes
b2180	No	No	No	No	No	No	No	No	No	No	No	No	Yes
b2197	No	No	No	Yes	No	No	No	No	No	No	No	Yes	No
b2198	No	No	No	Yes	No	No	No	No	No	No	No	Yes	No
b2199	No	No	No	Yes	No	No	No	No	No	No	No	Yes	No
b2200	No	No	No	Yes	No	No	No	No	No	No	No	Yes	No
b2201	No	No	No	Yes	No	No	No	No	No	No	No	Yes	No
b2202	No	No	No	Yes	No	No	No	No	No	No	No	No	No
b2203	No	No	No	No	No	No	No	No	No	Yes	No	No	No
b2204	No	No	No	No	No	No	No	No	No	Yes	No	No	No
b2205	No	No	No	No	No	No	No	No	No	Yes	No	No	No
b2206	No	No	No	No	No	No	No	No	No	Yes	No	No	No
b2210	No	No	No	No	No	No	No	Yes	No	No	No	No	No
b2215	No	No	No	No	No	No	No	No	No	No	No	Yes	No
b2221	No	No	No	No	No	No	No	No	No	No	No	No	Yes
b2222	No	No	No	No	No	No	No	No	No	No	No	No	Yes
b2223	No	No	No	No	No	No	No	No	No	No	No	Yes	No
b2224	No	No	No	No	No	No	No	No	No	No	No	No	Yes
b2232	No	No	No	Yes	No	No	No	No	No	No	No	No	No
b2234	No	Yes	No	No	No	No	No	No	No	No	No	No	No
b2235	No	Yes	No	No	No	No	No	No	No	No	No	No	No
b2239	No	No	No	No	No	No	No	No	No	No	No	No	Yes
b2240	No	No	No	No	No	No	No	No	No	No	No	Yes	No
b2241	No	No	No	No	No	No	No	No	No	Yes	No	No	No
b2242	No	No	No	No	No	No	No	No	No	Yes	No	No	No
b2243	No	No	No	No	No	No	No	No	No	Yes	No	No	No
b2245	No	No	No	No	No	No	No	No	No	No	No	No	Yes
b2247	No	No	No	No	No	No	No	No	No	No	No	No	Yes
b2251	No	Yes	No	No	No	No	No	No	No	No	No	No	No
b2253	No	No	Yes	No	No	No	No	No	No	No	No	No	No
b2254	No	No	Yes	No	No	No	No	No	No	No	No	No	No
b2255	No	No	Yes	No	No	No	No	No	No	No	No	No	No
b2256	No	No	Yes	No	No	No	No	No	No	No	No	No	No
b2257	No	No	Yes	No	No	No	No	No	No	No	No	No	No
b2258	No	No	No	No	No	No	No	No	No	No	No	No	Yes
b2260	No	No	No	Yes	No	No	No	No	No	No	No	No	No
b2261	No	No	No	Yes	No	No	No	No	No	No	No	No	No
b2262	No	No	No	Yes	No	No	No	No	No	No	No	No	No
b2263	No	No	No	Yes	No	No	No	No	No	No	No	No	No
b2264	No	No	No	Yes	No	No	No	No	No	No	No	No	No
b2265	No	No	No	Yes	No	No	No	No	No	No	No	No	No
b2276	No	No	No	No	No	No	No	No	No	Yes	No	No	No
b2277	No	No	No	No	No	No	No	No	No	Yes	No	No	No
b2278	No	No	No	No	No	No	No	No	No	Yes	No	No	No
b2279	No	No	No	No	No	No	No	No	No	Yes	No	No	No
b2280	No	No	No	No	No	No	No	No	No	Yes	No	No	No
b2281	No	No	No	No	No	No	No	No	No	Yes	No	No	No
b2282	No	No	No	No	No	No	No	No	No	Yes	No	No	No
b2283	No	No	No	No	No	No	No	No	No	Yes	No	No	No
b2284	No	No	No	No	No	No	No	No	No	Yes	No	No	No
b2285	No	No	No	No	No	No	No	No	No	Yes	No	No	No
b2286	No	No	No	No	No	No	No	No	No	Yes	No	No	No
b2287	No	No	No	No	No	No	No	No	No	Yes	No	No	No
b2288	No	No	No	No	No	No	No	No	No	Yes	No	No	No
b2290	Yes	No	No	No	No	No	No	No	No	No	No	No	No
b2291	No	No	No	No	No	No	No	No	No	No	No	No	Yes
b2293	No	No	No	No	No	No	No	No	No	No	No	No	Yes
b2296	No	No	No	No	No	No	No	No	No	No	Yes	No	No
b2297	No	No	No	No	No	No	No	No	No	No	Yes	No	No
b2302	No	No	No	No	No	No	No	No	No	No	No	No	Yes
b2303	No	No	No	Yes	No	No	No	No	No	No	No	No	No
b2306	No	No	No	No	No	No	No	No	No	No	No	Yes	No
b2307	No	No	No	No	No	No	No	No	No	No	No	Yes	No
b2308	No	No	No	No	No	No	No	No	No	No	No	Yes	No
b2309	No	No	No	No	No	No	No	No	No	No	No	Yes	No
b2310	No	No	No	No	No	No	No	No	No	No	No	Yes	No
b2312	No	Yes	No	No	No	No	No	No	No	No	No	No	No
b2315	No	No	No	Yes	No	No	No	No	No	No	No	No	No
b2316	No	No	Yes	No	No	No	No	No	No	No	No	No	No
b2320	No	No	No	Yes	No	No	No	No	No	No	No	No	No
b2323	No	No	Yes	Yes	No	No	No	No	No	No	No	No	No
b2328	No	No	No	No	No	No	No	No	No	No	No	No	Yes
b2329	Yes	No	No	No	No	No	No	No	No	No	No	No	No
b2341	No	No	No	No	No	No	No	No	No	No	No	No	Yes
b2342	No	No	No	No	No	No	No	No	No	No	No	No	Yes

b2344	No	No	No	No	No	No	No	No	No	No	No	Yes	No
b2361	No	No	No	No	No	No	No	No	No	No	No	No	Yes
b2366	No	No	No	No	No	No	No	No	No	No	No	No	Yes
b2371	No	No	No	No	No	No	No	No	No	No	No	No	Yes
b2373	No	No	No	No	No	No	No	No	No	No	No	No	Yes
b2374	No	No	No	No	No	No	No	No	No	No	No	No	Yes
b2378	No	No	Yes	No	No	No	No	No	No	No	No	No	No
b2379	Yes	No	No	No	No	No	No	No	No	No	No	No	No
b2388	No	No	No	No	No	No	No	No	No	No	No	No	Yes
b2392	No	No	No	No	No	No	No	No	No	No	No	Yes	No
b2393	No	No	No	No	No	No	No	No	No	No	No	Yes	No
b2400	No	No	No	No	Yes	No	No	No	No	No	No	No	No
b2406	No	No	No	No	No	No	No	No	No	No	No	Yes	No
b2407	No	No	No	No	Yes	No	No	No	No	No	No	No	No
b2411	No	No	No	No	No	No	No	No	No	No	No	No	Yes
b2413	No	No	No	No	No	No	No	No	No	No	No	Yes	No
b2414	Yes	No	No	No	No	No	No	No	No	No	No	No	No
b2415	No	No	No	No	No	No	No	No	No	No	No	Yes	No
b2416	No	No	No	No	No	No	No	No	No	No	No	Yes	No
b2417	No	No	No	No	No	No	No	No	No	No	No	Yes	No
b2418	No	No	No	Yes	No	No	No	No	No	No	No	No	No
b2421	Yes	No	No	No	No	No	No	No	No	No	No	No	No
b2422	No	No	No	Yes	No	No	No	No	No	No	No	Yes	No
b2423	No	No	No	Yes	No	No	No	No	No	No	No	Yes	No
b2424	No	No	No	Yes	No	No	No	No	No	No	No	Yes	No
b2425	No	No	No	Yes	No	No	No	No	No	No	No	Yes	No
b2428	No	No	No	No	No	No	No	No	No	No	No	No	Yes
b2429	No	No	No	No	No	No	No	No	No	No	No	Yes	No
b2430	No	No	Yes	No	No	No	No	No	No	No	No	No	No
b2431	No	No	No	No	No	No	No	No	No	No	No	No	Yes
b2435	No	No	No	No	No	No	No	No	No	No	No	No	Yes
b2436	No	No	No	Yes	No	No	No	No	No	No	No	No	No
b2440	No	No	No	No	No	No	No	No	No	No	No	No	Yes
b2441	No	No	No	No	No	No	No	No	No	No	No	No	Yes
b2458	No	No	No	No	No	No	No	No	No	No	Yes	No	No
b2463	No	No	No	No	Yes	No	No	No	No	No	No	No	No
b2464	No	No	No	No	No	No	Yes	No	No	No	No	No	No
b2465	No	No	No	No	No	No	Yes	No	No	No	No	No	No
b2467	No	No	No	No	No	No	No	No	No	No	No	No	Yes
b2470	No	No	No	No	No	No	No	No	No	No	No	Yes	No
b2472	Yes	No	No	No	No	No	No	No	No	No	No	No	No
b2476	No	Yes	No	No	No	No	No	No	No	No	No	No	No
b2478	Yes	No	No	No	No	No	No	No	No	No	No	No	No
b2480	No	No	No	No	No	No	No	No	No	No	No	No	Yes
b2481	No	No	No	No	No	No	No	No	No	No	No	No	Yes
b2482	No	No	No	No	No	No	No	No	No	No	No	No	Yes
b2483	No	No	No	No	No	No	No	No	No	No	No	No	Yes
b2484	No	No	No	No	No	No	No	No	No	No	No	No	Yes
b2485	No	No	No	No	No	No	No	No	No	No	No	No	Yes
b2486	No	No	No	No	No	No	No	No	No	No	No	No	Yes
b2487	No	No	No	No	No	No	No	No	No	No	No	No	Yes
b2488	No	No	No	No	No	No	No	No	No	No	No	No	Yes
b2489	No	No	No	No	No	No	No	No	No	No	No	No	Yes
b2490	No	No	No	No	No	No	No	No	No	No	No	No	Yes
b2492	No	No	No	No	No	No	No	No	No	No	No	Yes	No
b2497	No	No	No	No	No	No	No	No	No	No	No	Yes	No
b2498	No	No	No	No	Yes	No	No	No	No	No	No	No	No
b2499	No	Yes	No	No	No	No	No	No	No	No	No	No	No
b2500	No	Yes	No	No	No	No	No	No	No	No	No	No	No
b2501	No	No	No	No	No	No	No	No	No	No	No	No	Yes
b2502	No	No	No	No	No	No	No	No	No	No	No	No	Yes
b2503	No	No	No	No	No	No	No	No	No	No	No	No	Yes
b2507	No	Yes	No	No	No	No	No	No	No	No	No	No	No
b2508	No	Yes	No	No	No	No	No	No	No	No	No	No	No
b2515	No	No	No	No	Yes	No	No	No	No	No	No	No	No
b2518	No	Yes	No	No	No	No	No	No	No	No	No	No	No
b2519	No	No	Yes	No	No	No	No	No	No	No	No	No	No
b2521	No	No	No	No	No	No	No	No	No	No	No	No	Yes
b2523	No	No	No	No	No	No	No	No	No	No	No	No	Yes
b2528	No	No	No	No	No	No	No	No	No	No	No	No	Yes
b2529	No	No	No	No	No	No	No	No	No	No	No	No	Yes
b2530	Yes	No	No	Yes	No	No	No	No	No	No	No	No	No
b2533	No	No	No	No	No	No	No	No	No	No	No	No	Yes
b2536	No	No	No	No	No	No	No	No	No	No	No	Yes	No
b2538	No	No	No	No	No	No	No	No	No	No	No	No	Yes
b2539	No	No	No	No	No	No	No	No	No	No	No	No	Yes
b2540	No	No	No	No	No	No	No	No	No	No	No	No	Yes
b2541	No	No	No	No	No	No	No	No	No	No	No	No	Yes
b2542	No	No	No	No	No	No	No	No	No	No	No	No	Yes
b2551	Yes	No	No	Yes	No	No	No	No	No	No	No	No	No
b2552	No	No	No	No	No	No	No	No	No	No	No	No	Yes
b2557	No	Yes	No	No	No	No	No	No	No	No	No	No	No
b2558	No	No	No	No	No	No	No	No	No	No	No	No	Yes
b2563	No	No	No	Yes	No	No	No	No	No	No	No	No	No
b2564	No	No	No	Yes	No	No	No	No	No	No	No	No	No
b2574	No	No	No	Yes	No	No	No	No	No	No	No	No	No
b2578	No	No	No	No	No	No	No	No	No	No	No	Yes	No
b2579	No	No	No	No	No	No	No	No	No	No	No	No	Yes
b2582	No	Yes	No	Yes	No	No	No	No	No	No	No	No	No
b2585	No	No	Yes	No	No	No	No	No	No	No	No	No	No
b2587	No	No	No	No	No	No	No	No	No	No	No	Yes	No

b2599	Yes	No	No	No	No	No	No	No	No	No	No	No	No
b2600	Yes	No	No	No	No	No	No	No	No	No	No	No	No
b2601	Yes	No	No	No	No	No	No	No	No	No	No	No	No
b2615	No	No	No	Yes	No	No	No	No	No	No	No	No	No
b2660	No	No	No	No	No	No	No	No	No	No	No	No	Yes
b2661	No	No	No	No	No	No	No	No	No	No	No	No	Yes
b2662	Yes	No	No	No	No	No	No	No	No	No	No	No	No
b2663	No	No	No	No	No	No	No	No	No	No	No	Yes	No
b2668	No	No	No	No	No	No	No	No	No	No	No	No	Yes
b2670	No	No	No	No	No	No	No	No	No	No	No	Yes	No
b2675	No	Yes	No	No	No	No	No	No	No	No	No	No	No
b2676	No	Yes	No	No	No	No	No	No	No	No	No	No	No
b2677	No	No	No	No	No	No	No	No	No	No	No	Yes	No
b2678	No	No	No	No	No	No	No	No	No	No	No	Yes	No
b2679	No	No	No	No	No	No	No	No	No	No	No	Yes	No
b2682	No	No	No	No	No	No	No	No	No	No	No	Yes	No
b2683	No	No	No	No	No	No	No	No	No	No	No	Yes	No
b2687	No	No	No	Yes	No	No	No	No	No	No	No	No	No
b2688	No	No	No	Yes	No	No	No	No	No	No	No	No	No
b2690	No	No	No	No	No	No	No	No	No	No	No	No	Yes
b2700	No	No	No	Yes	No	No	No	No	No	No	No	No	No
b2701	No	No	No	No	No	No	No	No	No	No	No	No	Yes
b2702	No	No	No	No	No	No	No	No	No	No	No	Yes	No
b2703	No	No	No	No	No	No	No	No	No	No	No	Yes	No
b2704	No	No	No	No	No	No	No	No	No	No	No	Yes	No
b2705	No	No	No	No	No	No	No	No	No	No	No	No	Yes
b2708	No	No	Yes	No	No	No	No	No	No	No	No	No	No
b2710	No	No	No	No	No	No	No	No	No	No	No	No	Yes
b2711	No	No	No	No	No	No	No	No	No	No	No	No	Yes
b2715	No	No	No	No	No	No	No	No	No	No	No	Yes	No
b2716	No	No	No	No	No	No	No	No	No	No	No	No	Yes
b2719	No	No	No	No	No	No	No	No	No	No	Yes	No	No
b2720	No	No	No	No	No	No	No	No	No	No	Yes	No	No
b2721	No	No	No	No	No	No	No	No	No	No	Yes	No	No
b2722	No	No	No	No	No	No	No	No	No	No	Yes	No	No
b2723	No	No	No	No	No	No	No	No	No	No	Yes	No	No
b2724	No	No	No	No	No	No	No	No	No	No	Yes	No	No
b2727	No	No	No	No	No	No	No	No	No	No	No	No	Yes
b2738	No	No	No	No	No	No	No	No	No	No	No	No	Yes
b2744	No	No	No	No	No	No	No	No	No	No	No	No	Yes
b2746	No	No	No	No	Yes	No	No	No	No	No	No	No	No
b2747	No	No	No	No	Yes	No	No	No	No	No	No	No	No
b2750	No	No	No	No	No	No	No	No	No	No	No	No	Yes
b2751	No	No	No	No	No	No	No	No	No	No	No	No	Yes
b2752	No	No	No	No	No	No	No	No	No	No	No	No	Yes
b2762	No	No	No	No	No	No	No	No	No	No	No	No	Yes
b2763	No	No	No	No	No	No	No	No	No	No	No	No	Yes
b2764	No	No	No	No	No	No	No	No	No	No	No	No	Yes
b2765	No	No	No	No	Yes	No	No	No	No	No	No	No	No
b2779	No	No	No	No	Yes	Yes	No	No	No	No	No	No	No
b2780	No	Yes	No	No	No	No	No	No	No	No	No	No	No
b2781	No	Yes	No	No	No	No	No	No	No	No	No	No	No
b2784	No	No	No	No	Yes	No	No	No	No	No	No	No	No
b2787	No	No	No	No	No	No	No	No	No	No	No	No	Yes
b2788	No	No	No	No	No	No	No	No	No	No	No	No	Yes
b2789	No	No	No	No	No	No	No	No	No	No	No	Yes	No
b2794	No	No	No	No	No	No	No	No	No	No	No	No	Yes
b2796	No	No	No	No	No	No	No	No	No	No	No	Yes	No
b2797	No	No	No	No	No	No	No	No	No	No	No	No	Yes
b2799	No	No	No	No	No	No	No	No	No	No	No	No	Yes
b2800	No	No	No	No	No	No	No	No	No	No	No	No	Yes
b2801	No	No	No	No	No	No	No	No	No	No	No	Yes	No
b2802	No	No	No	No	No	No	No	No	No	No	No	No	Yes
b2803	No	No	No	No	No	No	No	No	No	No	No	No	Yes
b2810	No	No	No	No	No	No	No	No	No	No	No	No	Yes
b2813	No	No	No	No	No	No	No	No	No	No	No	No	Yes
b2817	No	No	No	No	No	No	No	No	No	No	No	No	Yes
b2818	Yes	No	No	No	No	No	No	No	No	No	No	No	No
b2827	No	Yes	No	Yes	No	No	No	No	No	No	No	No	No
b2835	No	No	No	No	No	No	No	No	No	No	No	Yes	No
b2836	No	No	No	No	No	No	No	No	No	No	No	No	Yes
b2838	Yes	No	No	No	No	No	No	No	No	No	No	No	No
b2841	No	No	No	No	No	No	No	No	No	No	No	Yes	No
b2842	No	No	No	No	No	No	No	No	No	No	No	No	Yes
b2843	No	No	No	No	No	No	No	No	No	No	No	No	Yes
b2866	No	No	No	No	No	No	No	No	No	No	No	No	Yes
b2867	No	No	No	No	No	No	No	No	No	No	No	No	Yes
b2868	No	No	No	No	No	No	No	No	No	No	No	No	Yes
b2871	No	No	No	No	No	No	No	No	No	No	No	No	Yes
b2873	No	No	No	No	No	No	No	No	No	No	No	No	Yes
b2877	No	No	No	Yes	No	No	No	No	No	No	No	No	No
b2882	No	No	No	No	No	No	No	No	No	No	No	Yes	No
b2883	No	No	No	No	No	No	No	No	No	No	No	No	Yes
b2888	No	No	No	No	No	No	No	No	No	No	No	Yes	No
b2889	No	No	No	Yes	No	No	No	No	No	No	No	No	No
b2893	No	No	No	No	No	No	No	No	No	No	No	Yes	No
b2895	No	No	No	No	Yes	No	No	No	No	No	No	No	No
b2901	No	No	No	No	No	No	No	No	No	No	No	No	Yes
b2903	No	No	No	Yes	No	No	No	No	No	No	No	No	No
b2904	No	No	No	Yes	No	No	No	No	No	No	No	No	No
b2905	No	No	No	Yes	No	No	No	No	No	No	No	No	No

b2906	No	No	No	Yes	No	No	No	No	No	No	No	No	No
b2907	No	No	No	Yes	No	No	No	No	No	No	No	No	No
b2912	No	No	No	Yes	No	No	No	No	No	No	No	No	No
b2913	Yes	No	No	No	No	No	No	No	No	No	No	No	No
b2914	No	No	No	No	No	No	Yes	No	No	No	No	No	No
b2917	No	No	No	No	No	No	No	No	No	No	No	No	Yes
b2918	No	No	No	No	No	No	No	No	No	No	No	No	Yes
b2919	No	No	No	No	No	No	No	No	No	No	No	No	Yes
b2920	No	No	No	No	No	No	No	No	No	No	No	No	Yes
b2923	No	No	No	No	No	No	No	No	No	No	No	Yes	No
b2924	No	No	No	No	No	No	No	No	No	No	No	Yes	No
b2925	No	No	No	No	Yes	Yes	No	No	No	No	No	No	No
b2926	No	No	No	No	Yes	Yes	No	No	No	No	No	No	No
b2927	No	No	No	Yes	No	No	No	No	No	No	No	No	No
b2930	No	No	No	No	Yes	Yes	No	No	No	No	No	No	No
b2933	No	No	No	No	No	No	No	No	No	No	No	Yes	No
b2934	No	No	No	No	No	No	No	No	No	No	No	Yes	No
b2935	No	No	No	No	No	No	Yes	No	No	No	No	No	No
b2937	No	No	No	No	Yes	No	No	No	No	No	No	No	No
b2938	No	No	No	No	Yes	No	No	No	No	No	No	No	No
b2942	No	No	No	Yes	No	No	No	No	No	No	No	No	No
b2943	No	No	No	No	No	No	No	No	No	No	No	Yes	No
b2947	No	No	No	Yes	No	No	No	No	No	No	No	No	No
b2954	No	No	No	No	No	No	No	No	No	No	No	No	Yes
b2957	Yes	No	No	No	No	No	No	No	No	No	No	No	No
b2963	No	No	No	No	No	No	No	No	No	No	No	No	Yes
b2964	No	No	No	No	No	No	No	No	No	No	No	Yes	No
b2965	No	No	No	No	Yes	No	No	No	No	No	No	No	No
b2975	No	No	No	No	No	No	No	No	No	No	No	Yes	No
b2976	No	No	No	No	No	No	No	No	Yes	No	No	No	No
b2979	No	No	No	No	No	No	No	No	No	No	No	No	Yes
b2987	No	No	No	No	No	No	No	No	No	No	No	Yes	No
b2988	No	No	No	Yes	No	No	No	No	No	No	No	No	No
b2989	No	No	No	No	No	No	No	No	No	No	No	No	Yes
b2994	No	No	No	No	No	No	No	No	No	Yes	No	No	No
b2995	No	No	No	No	No	No	No	No	No	Yes	No	No	No
b2996	No	No	No	No	No	No	No	No	No	Yes	No	No	No
b2997	No	No	No	No	No	No	No	No	No	Yes	No	No	No
b3001	No	No	No	No	No	No	No	No	No	No	No	No	Yes
b3003	No	No	No	No	No	No	No	No	No	No	No	No	Yes
b3005	No	No	No	No	No	No	No	No	No	No	No	Yes	No
b3006	No	No	No	No	No	No	No	No	No	No	No	Yes	No
b3008	Yes	No	No	No	No	No	No	No	No	No	No	No	No
b3011	No	No	No	No	No	No	No	No	No	No	No	No	Yes
b3012	No	No	No	No	No	No	No	No	No	No	No	No	Yes
b3018	No	No	Yes	No	No	No	No	No	No	No	No	No	No
b3028	No	No	No	No	No	No	No	No	No	No	No	No	Yes
b3029	No	No	No	No	No	No	No	No	No	No	No	No	Yes
b3032	No	No	No	No	No	No	No	No	No	No	No	No	Yes
b3034	No	No	No	Yes	No	No	No	No	No	No	No	No	No
b3035	No	No	No	No	No	No	No	No	No	No	No	Yes	No
b3040	No	No	No	No	No	No	No	No	No	No	No	Yes	No
b3041	No	No	No	Yes	No	No	No	No	No	No	No	No	No
b3052	No	No	No	No	Yes	No	No	No	No	No	No	No	No
b3057	No	No	No	Yes	No	No	No	No	No	No	No	No	No
b3058	No	No	No	Yes	No	No	No	No	No	No	No	No	No
b3059	No	No	No	No	No	No	No	No	No	No	No	No	Yes
b3061	No	No	No	No	No	No	No	No	No	No	No	No	Yes
b3062	No	No	No	No	No	No	No	No	No	No	No	No	Yes
b3063	No	No	No	No	No	No	No	No	No	No	No	Yes	No
b3070	No	No	No	No	No	No	No	No	No	No	No	No	Yes
b3073	No	No	No	No	No	No	No	No	No	No	No	No	Yes
b3089	No	No	No	No	No	No	No	No	No	No	No	Yes	No
b3091	No	No	No	No	No	No	No	No	No	No	No	No	Yes
b3092	No	No	No	No	No	No	No	No	No	No	No	No	Yes
b3093	No	No	No	No	No	No	No	No	No	No	No	Yes	No
b3114	No	No	No	No	No	No	No	No	No	No	Yes	No	No
b3115	No	No	No	No	No	No	No	No	No	No	No	No	Yes
b3116	No	No	No	No	No	No	No	No	No	No	No	Yes	No
b3117	No	No	No	No	No	No	No	No	No	No	No	No	Yes
b3124	No	No	No	No	No	No	No	No	No	No	No	No	Yes
b3125	No	No	No	No	No	No	No	No	No	No	No	No	Yes
b3126	No	No	No	No	No	No	No	No	No	No	No	No	Yes
b3127	No	No	No	No	No	No	No	No	No	No	No	Yes	No
b3128	No	No	No	No	No	No	No	No	No	No	No	No	Yes
b3132	No	No	No	No	No	No	No	No	No	No	No	No	Yes
b3137	No	No	No	No	No	No	No	No	No	No	No	No	Yes
b3161	No	No	No	No	No	No	No	No	No	No	No	Yes	No
b3172	Yes	No	No	No	No	No	No	No	No	No	No	No	No
b3176	No	No	No	No	Yes	No	No	No	No	No	No	No	No
b3177	No	No	No	Yes	No	No	No	No	No	No	No	No	No
b3182	No	No	Yes	No	No	No	No	No	No	No	No	No	No
b3187	No	No	No	Yes	No	No	No	No	No	No	No	No	No
b3189	No	No	Yes	No	No	No	No	No	No	No	No	No	No
b3196	No	No	No	No	No	No	No	No	No	No	No	Yes	No
b3197	No	No	Yes	No	No	No	No	No	No	No	No	No	No
b3198	No	No	Yes	No	No	No	No	No	No	No	No	No	No
b3199	No	No	No	No	No	No	No	No	No	No	No	No	Yes
b3200	No	No	No	No	No	No	No	No	No	No	No	No	Yes
b3201	No	No	No	No	No	No	No	No	No	No	No	No	Yes
b3208	No	No	Yes	No	No	No	No	No	No	No	No	No	No

b3212	Yes	No	No	No	No	No	No	No	No	No	No	No	No
b3213	Yes	No	No	No	No	No	No	No	No	No	No	No	No
b3222	No	No	No	No	No	No	No	No	No	No	No	No	Yes
b3223	No	No	No	No	No	No	No	No	No	No	No	No	Yes
b3224	No	No	No	No	No	No	No	No	No	No	No	Yes	No
b3225	No	No	No	No	No	No	No	No	No	No	No	No	Yes
b3236	No	No	No	No	Yes	No	No	Yes	No	No	No	No	No
b3240	No	No	No	No	No	No	No	No	No	No	No	Yes	No
b3241	No	No	No	No	No	No	No	No	No	No	No	Yes	No
b3248	No	No	No	No	No	No	No	No	No	No	No	No	Yes
b3253	No	No	No	No	No	No	No	No	No	No	No	No	Yes
b3255	No	No	No	No	No	No	No	No	No	No	No	No	Yes
b3256	No	No	Yes	No	No	No	No	No	No	No	No	No	No
b3258	No	No	No	No	No	No	No	No	No	No	No	Yes	No
b3265	No	No	No	No	No	No	No	No	No	No	No	Yes	No
b3266	No	No	No	No	No	No	No	No	No	No	No	Yes	No
b3281	Yes	No	No	No	No	No	No	No	No	No	No	No	No
b3290	No	No	No	No	No	No	No	No	No	No	No	Yes	No
b3291	No	No	No	No	No	No	No	No	No	No	No	Yes	No
b3336	No	No	No	No	No	No	No	No	No	No	No	Yes	No
b3350	No	No	No	No	No	No	No	No	No	No	No	Yes	No
b3359	Yes	No	No	No	No	No	No	No	No	No	No	No	No
b3360	No	No	No	Yes	No	No	No	No	No	No	No	No	No
b3365	No	No	No	No	No	No	No	No	No	No	No	No	Yes
b3366	No	No	No	No	No	No	No	No	No	No	No	No	Yes
b3367	No	No	No	No	No	No	No	No	No	No	No	Yes	No
b3368	No	No	No	No	Yes	No	No	No	No	No	No	No	No
b3370	No	No	No	No	No	No	No	No	No	No	No	Yes	No
b3371	No	No	No	No	No	No	No	No	No	No	No	No	Yes
b3374	No	No	No	No	No	No	No	No	No	No	No	No	Yes
b3385	No	No	No	No	No	No	No	No	No	No	No	No	Yes
b3386	No	No	No	No	No	No	Yes	No	No	No	No	No	No
b3389	Yes	No	No	No	No	No	No	No	No	No	No	No	No
b3390	Yes	No	No	No	No	No	No	No	No	No	No	No	No
b3396	No	No	Yes	No	No	No	No	No	No	No	No	No	No
b3397	No	No	No	No	No	No	No	No	No	No	No	No	Yes
b3399	No	No	No	No	No	No	No	No	No	No	No	No	Yes
b3403	No	No	No	No	Yes	No	No	No	No	No	No	No	No
b3409	No	No	No	No	No	No	No	No	No	No	No	Yes	No
b3412	No	No	No	Yes	No	No	No	No	No	No	No	No	No
b3415	No	No	No	No	No	No	No	No	No	No	No	Yes	No
b3416	No	No	No	No	No	No	No	No	No	No	No	No	Yes
b3417	No	No	No	No	No	No	No	No	No	No	No	No	Yes
b3425	No	No	No	No	No	No	No	No	No	No	No	No	Yes
b3426	No	No	No	No	No	No	No	No	No	Yes	No	No	No
b3428	No	No	No	No	No	No	No	No	No	No	No	No	Yes
b3429	No	No	No	No	Yes	No	No	No	No	No	No	No	No
b3430	No	No	No	No	Yes	No	No	No	No	No	No	No	No
b3431	No	No	No	No	No	No	No	No	No	No	No	No	Yes
b3432	No	No	No	No	Yes	No	No	No	No	No	No	No	No
b3433	Yes	No	No	No	No	No	No	No	No	No	No	No	No
b3437	No	No	No	No	No	No	No	No	No	No	No	No	Yes
b3447	No	No	No	No	No	No	No	No	No	No	No	No	Yes
b3449	No	No	No	No	No	No	No	No	No	No	No	No	Yes
b3450	No	No	No	No	No	No	No	No	No	No	No	Yes	No
b3451	No	No	No	No	No	No	No	No	No	No	No	Yes	No
b3452	No	No	No	No	No	No	No	No	No	No	No	Yes	No
b3453	No	No	No	No	No	No	No	No	No	No	No	Yes	No
b3454	No	No	No	No	No	No	No	No	No	No	No	Yes	No
b3455	No	No	No	No	No	No	No	No	No	No	No	Yes	No
b3456	No	No	No	No	No	No	No	No	No	No	No	Yes	No
b3457	No	No	No	No	No	No	No	No	No	No	No	Yes	No
b3458	No	No	No	No	No	No	No	No	No	No	No	Yes	No
b3460	No	No	No	No	No	No	No	No	No	No	No	Yes	No
b3469	No	No	No	No	No	No	No	No	No	No	No	Yes	No
b3475	No	No	No	Yes	No	No	No	No	No	No	No	No	No
b3476	No	No	No	No	No	No	No	No	No	No	No	Yes	No
b3477	No	No	No	No	No	No	No	No	No	No	No	Yes	No
b3478	No	No	No	No	No	No	No	No	No	No	No	Yes	No
b3479	No	No	No	No	No	No	No	No	No	No	No	Yes	No
b3480	No	No	No	No	No	No	No	No	No	No	No	Yes	No
b3488	No	No	No	No	No	No	No	No	No	No	No	No	Yes
b3493	No	No	No	No	No	No	No	No	No	No	No	Yes	No
b3496	No	No	No	No	No	No	No	No	No	No	No	Yes	No
b3500	No	No	No	No	No	No	No	No	No	No	No	No	Yes
b3502	No	No	No	No	No	No	No	No	No	No	No	Yes	No
b3503	No	No	No	No	No	No	No	No	No	No	No	No	Yes
b3517	No	No	No	No	No	No	No	No	No	No	No	No	Yes
b3519	No	No	No	No	No	No	No	No	No	No	No	No	Yes
b3525	No	No	No	No	No	No	No	No	No	No	No	No	Yes
b3526	No	No	No	No	No	No	No	No	No	No	No	No	Yes
b3528	No	No	No	No	No	No	No	No	No	No	No	Yes	No
b3540	No	No	No	No	No	No	No	No	No	No	No	Yes	No
b3541	No	No	No	No	No	No	No	No	No	No	No	Yes	No
b3542	No	No	No	No	No	No	No	No	No	No	No	Yes	No
b3543	No	No	No	No	No	No	No	No	No	No	No	Yes	No
b3544	No	No	No	No	No	No	No	No	No	No	No	Yes	No
b3546	No	No	No	No	No	No	No	No	No	No	No	No	Yes
b3551	No	No	No	No	No	No	No	No	No	No	No	No	Yes
b3553	No	No	No	No	No	No	No	No	No	No	No	No	Yes
b3564	No	No	No	No	No	No	No	No	No	No	No	No	Yes

b3565	No	No	No	No	No	No	No	No	No	No	No	No	Yes
b3566	No	No	No	No	No	No	No	No	No	No	No	Yes	No
b3567	No	No	No	No	No	No	No	No	No	No	No	Yes	No
b3568	No	No	No	No	No	No	No	No	No	No	No	Yes	No
b3571	No	No	No	No	No	No	No	No	No	No	No	No	Yes
b3572	Yes	No	No	No	No	No	No	No	No	No	No	No	No
b3575	No	No	No	No	No	No	No	No	No	No	No	No	Yes
b3577	No	No	No	No	No	No	No	No	No	No	No	Yes	No
b3578	No	No	No	No	No	No	No	No	No	No	No	Yes	No
b3579	No	No	No	No	No	No	No	No	No	No	No	Yes	No
b3580	No	No	No	No	No	No	No	No	No	No	No	No	Yes
b3581	No	No	No	No	No	No	No	No	No	No	No	No	Yes
b3582	No	No	No	No	No	No	No	No	No	No	No	No	Yes
b3583	No	No	No	No	No	No	No	No	No	No	No	No	Yes
b3588	No	No	No	No	No	No	No	No	No	No	No	No	Yes
b3589	No	No	No	No	No	No	No	No	No	No	No	No	Yes
b3590	No	No	No	No	No	No	No	No	No	No	No	No	Yes
b3599	No	No	No	No	No	No	No	No	No	No	No	Yes	No
b3600	No	No	No	No	No	No	No	No	No	No	No	No	Yes
b3603	No	No	No	No	No	No	No	No	No	No	No	Yes	No
b3605	No	No	No	No	No	No	No	No	No	No	No	No	Yes
b3607	Yes	No	No	No	No	No	No	No	No	No	No	No	No
b3608	No	No	Yes	No	No	No	No	No	No	No	No	No	No
b3610	Yes	No	No	Yes	No	No	No	No	No	No	No	No	No
b3612	No	No	No	No	Yes	Yes	No	No	No	No	No	No	No
b3614	No	No	No	No	No	No	No	No	No	No	No	No	Yes
b3616	No	No	No	No	No	No	No	No	No	No	No	No	Yes
b3617	No	No	No	No	No	No	No	No	No	No	No	No	Yes
b3619	No	No	No	No	Yes	No	No	No	No	No	No	No	No
b3620	No	No	Yes	No	No	No	No	No	No	No	No	No	No
b3621	No	No	Yes	No	No	No	No	No	No	No	No	No	No
b3622	No	No	No	No	No	No	No	No	No	No	No	No	Yes
b3623	No	No	Yes	No	No	No	No	No	No	No	No	No	No
b3624	No	No	No	No	No	No	No	No	No	No	No	No	Yes
b3625	No	No	Yes	No	No	No	No	No	No	No	No	No	No
b3626	No	No	Yes	No	No	No	No	No	No	No	No	No	No
b3627	No	No	Yes	No	No	No	No	No	No	No	No	No	No
b3628	No	No	Yes	No	No	No	No	No	No	No	No	No	No
b3629	No	No	No	No	No	No	No	No	No	No	No	No	Yes
b3630	No	No	Yes	No	No	No	No	No	No	No	No	No	No
b3631	No	No	Yes	No	No	No	No	No	No	No	No	No	No
b3632	No	No	Yes	No	No	No	No	No	No	No	No	No	No
b3633	No	No	Yes	No	No	No	No	No	No	No	No	No	No
b3634	No	No	No	Yes	No	No	No	No	No	No	No	No	No
b3639	No	No	No	Yes	No	No	No	No	No	No	No	No	No
b3640	No	Yes	No	No	No	No	No	No	No	No	No	No	No
b3642	No	Yes	No	No	No	No	No	No	No	No	No	No	No
b3648	No	Yes	No	No	No	No	No	No	No	No	No	No	No
b3650	No	No	No	No	Yes	No	No	No	No	No	No	No	No
b3653	No	No	No	No	No	No	No	No	No	No	No	Yes	No
b3654	No	No	No	No	No	No	No	No	No	No	No	Yes	No
b3656	No	No	No	No	No	No	No	No	No	No	No	No	Yes
b3662	No	No	No	No	No	No	No	No	No	No	No	Yes	No
b3664	No	No	No	No	No	No	No	No	No	No	No	Yes	No
b3665	No	No	No	No	No	No	No	No	No	No	No	No	Yes
b3666	No	No	No	No	No	No	No	No	No	No	No	Yes	No
b3670	Yes	No	No	No	No	No	No	No	No	No	No	No	No
b3671	Yes	No	No	No	No	No	No	No	No	No	No	No	No
b3679	No	No	No	No	No	No	No	No	No	No	No	Yes	No
b3691	No	No	No	No	No	No	No	No	No	No	No	Yes	No
b3693	No	No	No	No	No	No	No	No	No	No	No	No	Yes
b3697	No	No	No	No	No	No	No	No	No	No	No	No	Yes
b3708	No	No	No	No	No	No	No	No	No	No	No	No	Yes
b3709	No	No	No	No	No	No	No	No	No	No	No	Yes	No
b3713	No	No	No	No	No	No	No	No	No	No	No	No	Yes
b3714	No	No	No	No	No	No	No	No	No	No	No	Yes	No
b3715	No	No	No	No	No	No	No	No	No	No	No	No	Yes
b3721	No	No	No	No	No	No	No	No	No	No	No	No	Yes
b3722	No	No	No	No	No	No	No	No	No	No	No	Yes	No
b3725	No	No	No	No	No	No	No	No	No	No	No	Yes	No
b3726	No	No	No	No	No	No	No	No	No	No	No	Yes	No
b3727	No	No	No	No	No	No	No	No	No	No	No	Yes	No
b3728	No	No	No	No	No	No	No	No	No	No	No	Yes	No
b3729	No	No	No	No	Yes	No	No	No	No	No	No	No	No
b3730	No	No	No	No	Yes	No	No	No	No	No	No	No	No
b3731	No	No	No	No	No	No	No	No	No	Yes	No	No	No
b3732	No	No	No	No	No	No	No	No	No	Yes	No	No	No
b3733	No	No	No	No	No	No	No	No	No	Yes	No	No	No
b3734	No	No	No	No	No	No	No	No	No	Yes	No	No	No
b3735	No	No	No	No	No	No	No	No	No	Yes	No	No	No
b3736	No	No	No	No	No	No	No	No	No	Yes	No	No	No
b3737	No	No	No	No	No	No	No	No	No	Yes	No	No	No
b3738	No	No	No	No	No	No	No	No	No	Yes	No	No	No
b3739	No	No	No	No	No	No	No	No	No	No	No	No	Yes
b3744	Yes	No	No	No	No	No	No	No	No	No	No	No	No
b3747	No	No	No	No	No	No	No	No	No	No	No	Yes	No
b3748	No	No	No	No	No	No	No	No	No	No	No	Yes	No
b3749	No	No	No	No	No	No	No	No	No	No	No	Yes	No
b3750	No	No	No	No	No	No	No	No	No	No	No	Yes	No
b3751	No	No	No	No	No	No	No	No	No	No	No	Yes	No
b3752	No	No	No	No	No	No	No	No	No	No	No	No	Yes

b3770	Yes	No	No	No	No	No	No	No	No	No	No	No	No
b3771	Yes	No	No	No	No	No	No	No	No	No	No	No	No
b3772	Yes	No	No	No	No	No	No	No	No	No	No	No	No
b3774	Yes	No	No	Yes	No	No	No	No	No	No	No	No	No
b3779	No	No	No	No	Yes	No	No	No	No	No	No	No	No
b3781	No	Yes	No	Yes	No	No	No	No	No	No	No	No	No
b3784	No	No	Yes	No	No	No	No	No	No	No	No	No	No
b3785	No	No	No	No	No	No	No	No	No	No	No	No	Yes
b3786	No	No	Yes	No	No	No	No	No	No	No	No	No	No
b3787	No	No	Yes	No	No	No	No	No	No	No	No	No	No
b3788	No	No	Yes	No	No	No	No	No	No	No	No	No	No
b3789	No	No	Yes	No	No	No	No	No	No	No	No	No	No
b3790	No	No	Yes	No	No	No	No	No	No	No	No	No	No
b3791	No	No	Yes	No	No	No	No	No	No	No	No	No	No
b3792	No	No	Yes	No	No	No	No	No	No	No	No	No	No
b3793	No	No	No	No	No	No	No	No	No	No	No	No	Yes
b3794	No	No	Yes	No	No	No	No	No	No	No	No	No	No
b3804	No	No	No	No	Yes	No	No	No	No	No	No	No	No
b3805	No	No	No	No	Yes	No	No	No	No	No	No	No	No
b3806	No	No	No	No	No	No	No	No	No	No	No	No	Yes
b3809	Yes	No	No	No	No	No	No	No	No	No	No	No	No
b3812	No	No	No	Yes	No	No	No	No	No	No	No	No	No
b3816	No	No	No	No	No	No	No	No	No	No	No	Yes	No
b3821	No	No	No	No	No	No	No	No	No	No	No	No	Yes
b3823	No	No	No	No	No	No	No	No	No	No	No	Yes	No
b3824	No	No	No	No	No	No	No	No	No	No	No	Yes	No
b3825	No	No	No	No	No	No	No	No	No	No	No	No	Yes
b3826	No	No	No	No	No	No	No	No	No	No	No	No	Yes
b3829	Yes	No	No	Yes	No	No	No	No	No	No	No	No	No
b3831	No	No	No	No	Yes	No	No	No	No	No	No	No	No
b3833	No	No	No	Yes	No	No	No	No	No	No	No	No	No
b3843	No	No	No	Yes	No	No	No	No	No	No	No	No	No
b3844	No	No	No	No	No	No	No	No	No	No	No	No	Yes
b3845	No	No	No	No	No	No	No	No	No	No	No	No	Yes
b3846	No	No	No	No	No	No	No	No	No	No	No	No	Yes
b3849	No	No	No	No	No	No	No	No	No	No	No	Yes	No
b3850	No	No	No	Yes	No	No	No	No	No	No	No	No	No
b3856	No	No	No	No	No	No	No	No	No	No	No	No	Yes
b3857	No	No	No	Yes	No	No	No	No	No	No	No	No	No
b3860	No	No	No	Yes	No	No	No	No	No	No	No	No	No
b3867	No	No	No	Yes	No	No	No	No	No	No	No	No	No
b3870	Yes	No	No	No	No	No	No	No	No	No	No	No	No
b3875	No	No	No	No	No	No	No	No	No	No	No	Yes	No
b3876	No	No	No	No	No	No	No	No	No	No	No	Yes	No
b3877	No	No	No	No	No	No	No	No	No	No	No	Yes	No
b3878	No	No	No	No	No	No	No	No	No	No	No	No	Yes
b3880	No	No	No	No	No	No	No	No	No	No	No	No	Yes
b3881	No	No	No	No	No	No	No	No	No	No	No	No	Yes
b3882	No	No	No	No	No	No	No	No	No	No	No	No	Yes
b3883	No	No	No	No	No	No	No	No	No	No	No	No	Yes
b3885	No	No	No	No	No	No	No	No	No	No	No	No	Yes
b3892	No	No	No	No	No	No	No	No	No	Yes	No	No	No
b3893	No	No	No	No	No	No	No	No	No	Yes	No	No	No
b3894	No	No	No	No	No	No	No	No	No	Yes	No	No	No
b3902	No	No	No	No	No	No	No	No	No	No	No	No	Yes
b3903	No	No	No	No	No	No	No	No	No	No	No	No	Yes
b3904	No	No	No	No	No	No	No	No	No	No	No	No	Yes
b3907	No	No	No	No	No	No	No	No	No	No	No	Yes	No
b3908	No	No	No	No	No	No	No	No	No	No	No	No	Yes
b3909	No	No	No	No	No	No	No	No	No	No	No	Yes	No
b3915	No	No	No	No	No	No	No	No	No	No	No	Yes	No
b3916	No	No	No	No	No	Yes	No	No	No	No	No	No	No
b3917	No	No	No	Yes	No	No	No	No	No	No	No	Yes	No
b3918	No	No	No	No	No	No	No	No	No	No	No	No	Yes
b3919	No	No	No	No	Yes	Yes	No	No	No	No	No	No	No
b3924	No	No	No	No	No	No	No	No	No	No	No	No	Yes
b3925	No	No	No	No	Yes	Yes	No	No	No	No	No	No	No
b3926	No	No	No	No	No	No	No	No	No	No	No	No	Yes
b3927	No	No	No	No	No	No	No	No	No	No	No	Yes	No
b3930	No	No	No	Yes	No	No	No	No	No	No	No	No	No
b3939	Yes	No	No	No	No	No	No	No	No	No	No	No	No
b3940	Yes	No	No	No	No	No	No	No	No	No	No	No	No
b3941	No	No	No	Yes	No	No	No	No	No	No	No	No	No
b3942	No	No	No	No	No	No	No	No	No	No	No	No	Yes
b3943	No	No	No	No	No	No	No	No	No	No	No	Yes	No
b3945	No	No	No	No	No	No	No	No	No	No	No	No	Yes
b3946	No	No	No	No	No	No	No	No	No	No	No	No	Yes
b3951	No	No	No	No	No	No	No	No	No	No	No	No	Yes
b3952	No	No	No	No	No	No	No	No	No	No	No	No	Yes
b3956	No	No	No	No	No	No	No	No	No	No	Yes	No	No
b3957	Yes	No	No	No	No	No	No	No	No	No	No	No	No
b3958	Yes	No	No	No	No	No	No	No	No	No	No	No	No
b3959	Yes	No	No	No	No	No	No	No	No	No	No	No	No
b3960	Yes	No	No	No	No	No	No	No	No	No	No	No	No
b3962	No	No	No	No	No	No	No	No	No	No	No	No	Yes
b3966	No	No	No	No	No	No	No	No	No	No	No	Yes	No
b3967	No	No	Yes	No	No	No	No	No	No	No	No	No	No
b3972	No	No	Yes	No	No	No	No	No	No	No	No	No	No
b3974	No	No	No	Yes	No	No	No	No	No	No	No	No	No
b3990	No	No	No	Yes	No	No	No	No	No	No	No	No	No
b3991	No	No	No	Yes	No	No	No	No	No	No	No	No	No

b3992	No	No	No	Yes	No	No	No	No	No	No	No	No	No
b3993	No	No	No	Yes	No	No	No	No	No	No	No	No	No
b3994	No	No	No	Yes	No	No	No	No	No	No	No	No	No
b3996	No	No	No	No	No	No	No	No	No	No	No	No	Yes
b3997	No	No	No	Yes	No	No	No	No	No	No	No	No	No
b4005	No	Yes	No	No	No	No	No	No	No	No	No	No	No
b4006	No	Yes	No	No	No	No	No	No	No	No	No	No	No
b4013	Yes	No	No	No	No	No	No	No	No	No	No	No	No
b4014	No	No	No	No	No	No	No	No	Yes	No	No	No	No
b4015	No	No	No	No	No	No	No	No	Yes	No	No	No	No
b4019	Yes	No	No	Yes	No	No	No	No	No	No	No	No	No
b4020	No	No	No	No	No	No	No	No	No	No	No	Yes	No
b4024	Yes	No	No	No	No	No	No	No	No	No	No	No	No
b4025	No	No	No	No	Yes	Yes	No	No	No	No	No	No	No
b4031	No	No	No	No	No	No	No	No	No	No	No	Yes	No
b4032	No	No	No	No	No	No	No	No	No	No	No	Yes	No
b4033	No	No	No	No	No	No	No	No	No	No	No	Yes	No
b4034	No	No	No	No	No	No	No	No	No	No	No	Yes	No
b4035	No	No	No	No	No	No	No	No	No	No	No	Yes	No
b4036	No	No	No	No	No	No	No	No	No	No	No	Yes	No
b4039	No	No	No	Yes	No	No	No	No	No	No	No	No	No
b4040	No	No	No	Yes	No	No	No	No	No	No	No	No	No
b4041	No	No	Yes	No	No	No	No	No	No	No	No	No	No
b4042	No	No	No	No	No	No	No	No	No	No	No	No	Yes
b4053	Yes	No	No	No	No	No	No	No	No	No	No	No	No
b4054	Yes	No	No	No	No	No	No	No	No	No	No	No	No
b4055	No	No	No	No	No	No	No	No	No	No	No	No	Yes
b4064	No	No	No	No	No	No	No	No	No	No	No	Yes	No
b4067	No	No	No	No	No	No	No	No	No	No	No	Yes	No
b4069	No	No	No	No	No	No	No	No	No	No	No	No	Yes
b4070	No	No	No	Yes	No	No	No	No	No	Yes	No	No	No
b4071	No	No	No	Yes	No	No	No	No	No	No	No	No	No
b4072	No	No	No	No	No	No	No	No	No	Yes	No	No	No
b4073	No	No	No	No	No	No	No	No	No	Yes	No	No	No
b4077	No	No	No	No	No	No	No	No	No	No	No	Yes	No
b4079	No	No	No	No	No	No	No	No	No	No	Yes	No	No
b4084	No	No	No	No	No	No	No	No	No	No	No	No	Yes
b4085	No	No	No	No	No	No	No	No	No	No	No	No	Yes
b4086	No	No	No	No	No	No	No	No	No	No	No	Yes	No
b4087	No	No	No	No	No	No	No	No	No	No	No	Yes	No
b4088	No	No	No	No	No	No	No	No	No	No	No	Yes	No
b4090	No	No	No	No	No	No	Yes	No	No	No	No	No	No
b4092	No	No	No	No	No	No	No	No	No	No	No	No	Yes
b4093	No	No	No	No	No	No	No	No	No	No	No	No	Yes
b4094	No	No	No	No	No	No	No	No	No	No	No	No	Yes
b4095	No	No	No	No	No	No	No	No	No	No	No	No	Yes
b4096	No	No	No	No	No	No	No	No	No	No	No	No	Yes
b4098	No	No	No	No	No	No	No	No	No	No	No	No	Yes
b4099	No	No	No	No	No	No	No	No	No	No	No	No	Yes
b4100	No	No	No	No	No	No	No	No	No	No	No	No	Yes
b4101	No	No	No	No	No	No	No	No	No	No	No	No	Yes
b4104	No	No	No	No	No	No	No	No	No	No	No	Yes	No
b4105	No	No	No	No	No	No	No	No	No	No	No	Yes	No
b4106	No	No	No	No	No	No	No	No	No	No	No	Yes	No
b4111	No	No	No	No	No	No	No	No	No	No	No	Yes	No
b4115	No	No	No	No	No	No	No	No	No	No	No	Yes	No
b4117	No	No	No	No	No	No	No	No	No	No	No	No	Yes
b4119	No	No	No	No	No	No	No	No	No	No	No	No	Yes
b4120	No	No	No	No	No	No	No	No	No	No	No	Yes	No
b4122	No	No	No	No	No	No	No	Yes	No	No	No	No	No
b4123	No	No	No	No	No	No	No	No	No	No	No	Yes	No
b4129	No	No	No	No	Yes	No	No	No	No	No	No	No	No
b4130	No	No	No	No	No	No	No	No	No	No	No	Yes	No
b4131	No	No	No	No	Yes	No	No	No	No	No	No	No	No
b4132	No	No	No	No	No	No	No	No	No	No	No	Yes	No
b4136	No	No	No	Yes	No	No	No	No	No	No	No	Yes	No
b4138	No	No	No	No	No	No	No	No	No	No	No	Yes	No
b4139	Yes	No	No	No	No	No	No	No	No	No	No	No	No
b4141	No	No	No	No	No	No	No	No	No	No	No	Yes	No
b4146	No	No	No	No	No	No	No	No	No	No	No	No	Yes
b4151	No	No	No	No	No	No	No	No	No	Yes	No	No	No
b4152	No	No	No	No	No	No	No	No	No	Yes	No	No	No
b4153	No	No	No	No	No	No	No	No	No	Yes	No	No	No
b4154	No	No	No	No	No	No	No	No	No	Yes	No	No	No
b4159	No	No	No	No	No	No	No	No	No	No	No	Yes	No
b4160	No	No	Yes	No	No	No	No	No	No	No	No	No	No
b4161	No	No	No	No	No	No	No	No	No	No	No	No	Yes
b4167	No	No	No	No	No	No	No	No	No	No	No	No	Yes
b4169	No	No	No	No	No	No	No	No	No	No	No	No	Yes
b4173	No	No	No	No	No	No	No	No	No	No	No	No	Yes
b4177	No	Yes	No	No	No	No	No	No	No	No	No	No	No
b4192	No	No	No	No	No	No	No	No	No	No	No	No	Yes
b4193	No	No	No	No	No	No	No	No	No	No	No	Yes	No
b4194	No	No	No	No	No	No	No	No	No	No	No	Yes	No
b4195	No	No	No	No	No	No	No	No	No	No	No	Yes	No
b4196	No	No	No	No	No	No	No	No	No	No	No	No	Yes
b4197	No	No	No	No	No	No	No	No	No	No	No	No	Yes
b4198	No	No	No	No	No	No	No	No	No	No	No	No	Yes
b4208	No	No	No	No	No	No	No	No	No	No	No	Yes	No
b4209	No	No	No	No	No	No	No	No	No	No	No	No	Yes
b4213	No	No	No	No	No	No	No	No	No	No	No	No	Yes

b4214	No	No	No	No	No	No	No	No	No	No	No	No	Yes
b4219	No	No	No	No	No	No	No	No	No	No	No	No	Yes
b4226	No	No	No	No	No	No	No	No	No	No	No	No	Yes
b4227	No	No	No	No	No	No	No	No	No	No	No	Yes	No
b4230	No	No	No	No	No	No	No	No	No	No	No	Yes	No
b4231	No	No	No	No	No	No	No	No	No	No	No	Yes	No
b4232	No	No	No	No	Yes	Yes	No	No	No	No	No	No	No
b4233	No	No	No	No	No	No	No	No	No	No	No	No	Yes
b4237	No	No	No	No	No	No	No	No	No	No	No	No	Yes
b4238	No	Yes	No	No	No	No	No	No	No	No	No	No	No
b4239	No	No	No	No	No	No	No	No	No	No	No	No	Yes
b4240	No	No	No	No	No	No	No	No	No	No	No	Yes	No
b4242	No	No	No	No	No	No	No	No	No	No	No	Yes	No
b4244	No	Yes	No	No	No	No	No	No	No	No	No	No	No
b4245	No	Yes	No	No	No	No	No	No	No	No	No	No	No
b4254	Yes	No	No	No	No	No	No	No	No	No	No	No	No
b4260	No	No	No	No	No	No	No	No	No	No	No	No	Yes
b4261	No	No	No	No	No	No	No	No	No	No	No	No	Yes
b4262	No	No	No	No	No	No	No	No	No	No	No	No	Yes
b4265	No	No	No	No	No	No	No	No	No	No	No	Yes	No
b4266	No	No	No	No	No	No	No	No	No	No	No	No	Yes
b4267	No	No	No	No	No	No	No	No	No	No	No	No	Yes
b4268	No	No	No	No	No	No	No	No	No	No	No	No	Yes
b4269	No	No	No	No	No	No	No	No	No	No	No	No	Yes
b4287	No	No	No	No	No	No	No	No	No	No	No	Yes	No
b4288	No	No	No	No	No	No	No	No	No	No	No	Yes	No
b4289	No	No	No	No	No	No	No	No	No	No	No	Yes	No
b4290	No	No	No	No	No	No	No	No	No	No	No	Yes	No
b4291	No	No	No	No	No	No	No	No	No	No	No	Yes	No
b4297	No	No	No	No	No	No	No	No	No	No	No	No	Yes
b4298	No	No	No	No	No	No	No	No	No	No	No	No	Yes
b4311	No	No	No	No	No	No	No	No	No	No	No	Yes	No
b4321	No	No	No	No	No	No	No	No	No	No	No	Yes	No
b4322	No	No	No	No	No	No	No	No	No	No	No	No	Yes
b4323	No	No	No	No	No	No	No	No	No	No	No	No	Yes
b4337	No	No	No	No	No	No	No	No	No	No	No	Yes	No
b4352	No	No	No	No	No	No	No	No	No	No	No	No	Yes
b4356	No	No	No	No	No	No	No	No	No	No	No	Yes	No
b4358	No	No	No	No	No	No	No	No	No	No	No	No	Yes
b4367	No	No	No	No	No	No	No	No	No	No	No	No	Yes
b4374	No	No	No	No	No	No	No	No	No	No	No	No	Yes
b4381	No	No	No	No	No	No	No	No	No	No	No	No	Yes
b4382	No	No	No	No	Yes	No	No	No	No	No	No	No	No
b4383	No	No	No	No	No	No	No	No	No	No	No	No	Yes
b4384	No	No	No	No	Yes	No	No	No	No	No	No	No	No
b4386	No	No	No	Yes	No	No	No	No	No	No	No	No	No
b4388	Yes	No	No	No	No	No	No	No	No	No	No	No	No
b4390	No	No	No	Yes	No	No	No	No	No	No	No	No	No
b4392	No	No	No	No	No	No	No	No	No	No	No	No	Yes
b4394	No	No	No	No	No	No	No	No	No	No	No	No	Yes
b4407	No	No	No	Yes	No	No	No	No	No	No	No	No	No
b4460	No	No	No	No	No	No	No	No	No	No	No	Yes	No
b4464	No	No	No	No	No	No	No	No	No	No	No	Yes	No
b4467	No	No	No	No	No	No	No	No	No	No	No	No	Yes
b4468	No	No	No	No	No	No	No	No	No	No	No	No	Yes
b4471	No	No	No	No	No	No	No	No	No	No	No	No	Yes
b4474	No	No	No	No	No	No	No	No	No	No	No	No	Yes
b4476	No	No	No	No	No	No	No	No	No	No	No	Yes	No
b4477	No	No	No	No	No	No	No	No	No	No	No	No	Yes
b4478	No	No	No	No	No	No	No	No	No	No	No	No	Yes
b4481	No	No	Yes	No	No	No	No	No	No	No	No	No	No
b4485	No	No	No	No	No	No	No	No	No	No	No	Yes	No
b4513	No	No	No	No	No	No	No	No	No	No	No	Yes	No
b4515	No	No	No	No	No	No	No	No	No	Yes	No	No	No
b4544	No	No	No	No	No	No	No	No	No	No	No	No	Yes
